# Supplementary material for: Titanium catalyzed [2σ + 2π] cycloaddition of bicyclo[1.1.0]-butanes with 1,3-dienes for efficient synthesis of stilbene bioisosteres
Source: Nat Commun. 2024 May 23;15:4374. doi: 10.1038/s41467-024-48494-9 (PMC11116475; doi:10.1038/s41467-024-48494-9)
Supplement: Supplementary file 1 — Supplementary Information [file 41467_2024_48494_MOESM1_ESM.pdf]

Supplementary Information

Titanium Catalyzed [2σ+2π] Cycloaddition of Bicyclo[1.1.0]-butanes with 1,3-Dienes for Efficient Synthesis of Stilbene Bioisosteres

Yonghong Liu[1,2‡], Zhixian Wu[1,2‡], Jing-Ran Shan[3]*, Huaipu Yan[2], Er-Jun Hao[4]*, and Lei Shi[1,2,4]*

[1]Cancer Hospital of Dalian University of Technology, 116024, Dalian, China.

[2]School of Chemistry, Dalian University of Technology, 116024, Dalian, China.

[3]Department of Chemistry and Biochemistry, University of California Los Angeles, Los Angeles, California 90095, United States.

[4]Key Laboratory of Green Chemical Media and Reactions, Ministry of Education, School of Chemistry and Chemical Engineering, Henan Normal University, Xinxiang 453007, China.

[‡]These authors contributed equally: Yonghong Liu, Zhixian Wu.

*Corresponding Author(s): jrshan@chem.ucla.edu; hej@htu.edu.cn; shilei17@dlut.edu.cn

Table of Contents

1 Supplementary Notes 3

2 Supplementary Methods 4

2.1 Synthesis of Salen-titanium complexes 4

2.2 Procedures for the Preparation of bicyclo[1.1.0]-butane ketones 4

2.3 Procedures for the preparation of 1,3-diene substrates 7

2.4 Optimization of Reaction Conditions 11

2.5. Control Experiments 13

2.6 Radical Trapping Experiment 14

2.7 Gram-Scale Reaction 15

2.8 Prilezhaev epoxidation of 5b 15

2.9 Reduction of 5b 16

2.10 Wacker-type oxidation of 5b 16

2.11 Photocatalytic reaction of disulfide with 5b 17

2.12 Visible light promoted E→Z isomerization of alkene 3r 17

2.13 Amide conversion to ketone 18

2.14 The X-ray crystallographic data for compound 3a 19

2.15 Computational Details 22

2.16 General Procedure and Characterization Data 23

3 Supplementary Figures 45

4 Supplementary References 124

1 Supplementary Notes

Unless otherwise noted, all reactions of substrates preparation were conducted in flame – dried glassware under a nitrogen atmosphere using anhydrous solvent passed through an activated alumina column (Innovative Technology). Commercially available reagents were used without further purification. Thin layer chromatography (TLC) was performed using Huanghai TLC silica gel plates HSG F254 and visualized using UV light, anisaldehyde or potassium permanganate. ^{1}H and ^{13}C NMR spectra were recorded in $CDCl_3$ on a Bruker 400M spectrometer. Chemical shifts in ^{1}H NMR spectra were reported in parts per million (ppm) on the δ scale from an internal standard of residual $CDCl_3$ (7.26 ppm). Data for ^{1}H NMR were reported as follows: chemical shift, multiplicity (s = singlet, d = doublet, t = triplet, q = quartet, m = multiplet, br = broad), coupling constant in Hertz (Hz) and integration. Data for ^{13}C NMR spectra were reported in terms of chemical shift in ppm from the central peak of $CDCl_3$ (77.00 ppm). Data for mass spectra (HRMS) were obtained from an HPLC-Q-Tof mass spectrometer using acetonitrile as the mobile phase.

2 Supplementary Methods

2.1 Synthesis of Salen-titanium complexes

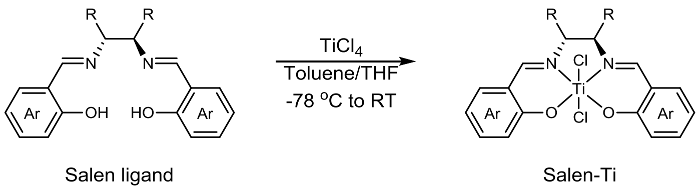

Supplementary Figure 1. Synthesis of Salen-titanium complexes

To a heat-dried Schlenk flask were added the salen ligand (5.0 mmol, 1.0 eq) and freshly distilled THF. The resulting yellow solution was cooled to -78 ℃ under N_2. Then $TiCl_4$ solution (1.0 M in toluene, 1.0 eq) was added dropwise into the above solution at the same temperature. The red suspension was then allowed to warm to r.t. and heated under reflux for 2 h. After the reaction was cooled to room temperature, the red solid was obtained by recrystallization with DCM and n-hexane. Then the red solid was washed with diethyl ether and *n*-hexane to afford Salen-Ti complex.

2.2 Procedures for the Preparation of bicyclo[1.1.0]-butane ketones

Procedure I:

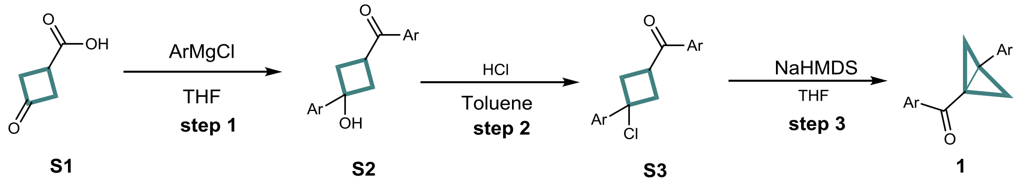

Supplementary Figure 2. Procedure I for the Preparation of bicyclo[1.1.0]-butane ketones

Step 1. To a solution of 3-oxocyclobutane-1-carboxylic acid **S1** (5.7 g, 50 mmol, 1.0 equiv) in dry THF (100 mL) was added ArMgCl (2.0 M, 80 mL, 160 mmol, 3.2 equiv) dropwise under argon at 0 ^{o}C. Move the reaction to room temperature, and the mixture stirred for 48 h at same conditions. Then, a saturated aq. solution of NH_4Cl (50 mL) and water (100 mL) were added dropwise to the solution. The resulting mixture was extracted with ethyl acetate for three times, and washed with saturated brine water. The combined organic layers were dried over magnesium sulfate, filtered and concentrated in vacuum. The resulting mixture was used in next step without further purification.

Step 2. To a solution of **S2** (50 mol, 1.0 equiv) in toluene (50 mL) was added HCl (50 mL) dropwise at room temperature. The resulted mixture was stirred at room temperature for 4 h. The organic phase was separated, and ethyl acetate (100 mL) to the resulted mixture, washed with water (100 mL), saturated $NaHCO_3$ (2 ×100 mL), brine (2 × 100 mL), dried over Na_2SO_4, filtered and concentrated under reduced pressure to give the **S3** as a colorless oil and used for next step without further purification.

Step 3. To a solution of NaHMDS (50 mol, 1 eqiuv) in THF (100 mL) was added a solution of **S3** (50 mol, 1.0 equiv) under argon at - 20 ℃. The resulted mixture was stirred for 3 h at the same temperature. Then, the resulting mixture was moved to room temperature and a sat. solution of NH_4Cl (50 mL) and water (100 mL) were added dropwise. The mixture was diluted with ethyl acetate (200 mL). The organic layer was separated, washed with brine (2 × 200 mL), dried over Na_2SO_4, filtered through SiO_2 and concentrated under reduced pressure. The resulting mixture was purified by flash column chromatography on silica gel (PE : EA = 15 : 1) to obtain bicyclo[1.1.0]-butane ketones yield.

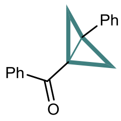

1a

Yield: 4.33 g, 37%, white solid. ^1H NMR (400 MHz, $CDCl_3$) δ 7.60 – 7.51 (m, 2H), 7.48 – 7.42 (m, 1H), 7.36 – 7.31 (m, 2H), 7.29 – 7.17 (m, 3H), 7.18 – 7.09 (m, 2H), 3.17 (s, 2H), 1.91 (s, 2H). ^{13}C NMR (101 MHz, $CDCl_3$) δ 196.47, 138.19, 132.71, 131.76, 128.29, 128.22, 127.93, 127.12, 125.92, 38.55, 37.61, 31.21. **HRMS**: calculated for $C_{17}H_{15}O$ $[M+H]^+$ 235.1117; found 235.1114.

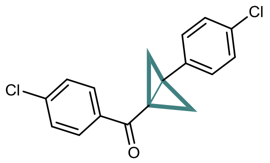

1b

Yield: 4.98 g, 33%, white solid. ^1H NMR (400 MHz, $CDCl_3$) δ 7.52 (d, J = 8.5 Hz, 2H), 7.33 (d, J = 8.6 Hz, 2H), 7.20 (d, J = 8.6 Hz, 2H), 7.06 (d, J = 8.6 Hz, 2H), 3.11 (s, 2H), 1.93 (s, 2H). ^{13}C NMR (101 MHz, $CDCl_3$) δ 194.74, 138.45, 136.24, 133.21, 131.31, 129.73, 128.64, 128.44, 127.21, 38.03, 37.85, 31.17. **HRMS**: calculated for $C_{17}H_{13}Cl_2O$ $[M+H]^+$ 303.0338; found 303.0351.

Procedure II:[2]

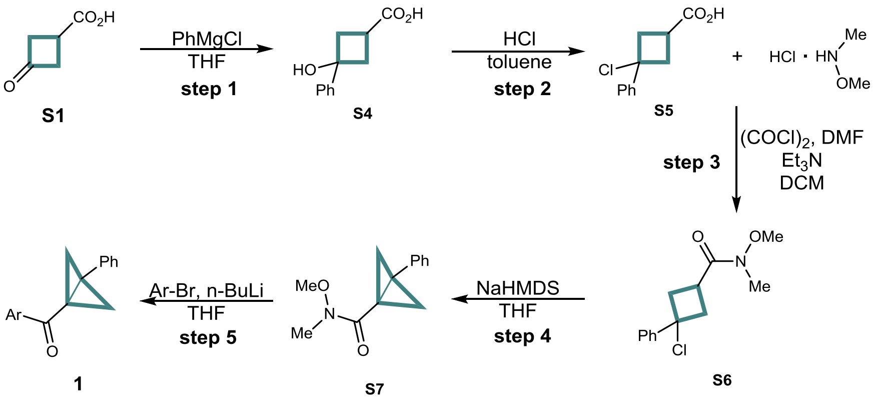

Supplementary Figure 3. Procedure II for the Preparation of bicyclo[1.1.0]-butane ketones

Step 1. To a solution of 3-oxocyclobutane-1-carboxylic acid **S1** (5.7 g, 50 mmol, 1.0 equiv) in dry THF (100 mL) was added ArMgCl (2.0 M, 2.1 equiv) dropwise under argon at 0 ^{o}C. Move the reaction to room temperature, and the mixture stirred for 3 h at same conditions. Then, HCl (1 M, 100 ml) was added to the solution. The resulting mixture was extracted with ethyl acetate for five times, and washed with saturated brine water. The combined organic layers were dried over magnessium sulfate, filtered and concentrated in vacuum. The resulting mixture was used in next step without further purification (After adding hydrochloric acid solution, it can be directly concentrated in vacuum to obtain crude product).

Step 2. To a solution of crude **S4** (50 mol, 1.0 equiv) in toluene (50 mL) was added HCl (50 mL) dropwise at room temperature. The resulted mixture was stirred at room temperature for 4 h. The organic phase was separated, and ethyl acetate (100 mL) to the resulted mixture, washed with water (100 mL), brine (2 × 100 mL), dried over Na_2SO_4, filtered and concentrated under reduced pressure to give the **S5** as a colorless oil and used for next step without further purification.

Step 3. A 500 mL round-bottomed flask immersed in ice bath was charged with crude acid **S5** (50.0 mmol, 1.00 eq.) and CH_2Cl_2 (100 mL) under N_2 atmosphere. To the stirred solution was added oxalyl chloride (1.1 equiv) dropwise over 30 minutes followed by addition of DMF (25 drops, catalytic amount) in one portion. After 2 hour, raw material N,O-dimethylhydroxylamine hydrochloride (50 mmol, 1 equiv) and Et_3N (150 mmol, 3 equiv) was added. The mixture was stirred for overnight at room temperature, quenched with H_2O (100 mL) and the organic layer was separated. Then the solvent was evapotated in vacuum. The crude amide **S6** was directly used in next reaction without further purification.

Step 4. To a solution of NaHMDS (50 mol, 1 eqiuv) in THF (100 mL) was added a solution of crude amide **S6** (50 mol, 1.0 equiv) under argon at - 20 ℃. The resulted mixture was stirred for 3 h at the same temperature. Then, the resulting mixture was moved to room temperature and a saturated solution of NH_4Cl (50 mL) and water (100 mL) were added dropwise. The mixture was diluted with ethyl acetate (200 mL). The organic layer was separated, washed with brine (2 × 200 mL), dried over Na_2SO_4, filtered through SiO_2 and concentrated under reduced pressure. The resulting mixture was purified by flash column chromatography on silica gel (PE : EA = 1 : 1) to obtain **S7** (4.6 g, 43%). ^{1}H NMR (400 MHz, $CDCl_3$) δ 7.36 – 7.24 (m, 4H), 7.23 – 7.14 (m, 1H), 3.61 (s, 3H), 3.08 (s, 3H), 2.98 (s, 2H), 1.57 (s, 2H). ^{13}C NMR (101 MHz, $CDCl_3$) δ 169.06, 133.95, 128.27, 126.51, 126.16, 61.13, 36.46, 33.46, 32.55, 22.52. **HRMS**: calculated for $C_{13}H_{16}NO_2$ [M+H] $^+$ 218.1176; found 218.1183.

Step 5. To a solution of aryl bromides (6 mmol, 2 equiv) in dry THF (15 mL) was added nBuLi (1.6 M, 3.7 mL, 6 mmol, 2 equiv) dropwise under argon at -78 ^{o}C. After stirred at the same temperature for 1 h, a solution of **S7** (3 mmol, 1.0 eq.) in THF (5 mL) was added. After 0.5 h, the resulting mixture was moved to room temperature for 1.5 h before quenched with saturated NH_4Cl solution (20 mL). The aqueous layer was extracted with EtOAc (3 x 20 mL), washed with brine (3 × 20 mL), dried over Na_2SO_4, filtered through SiO_2 and concentrated under reduced pressure. The resulting mixture was purified by flash column chromatography on silica gel (PE : EA = 10 : 1) to obtain

bicyclo[1.1.0]-butane ketones yield.

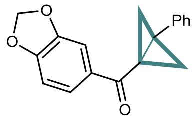

1c

^{1}H NMR (400 MHz, $CDCl_3$) δ 7.29 (dd, *J* = 8.1, 1.7 Hz, 1H), 7.27 – 7.13 (m, 5H), 7.05 (d, *J* = 1.7 Hz, 1H), 6.77 (d, *J* = 8.1 Hz, 1H), 5.98 (s, 2H), 3.16 (s, 2H), 1.88 (s, 2H). ^{13}C NMR (101 MHz, $CDCl_3$) δ 194.25, 150.89, 147.50, 132.90, 132.74, 128.34, 127.10, 126.01, 124.69, 108.28, 107.48, 101.58, 37.97, 37.68, 30.54. **HRMS**: calculated for $C_{18}H_{15}O_3$ [M+H]$^+$ 279.1016; found 279.1026.

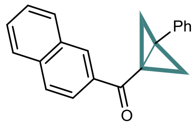

1d

^{1}H NMR (400 MHz, $CDCl_3$) δ 8.15 (d, *J* = 1.6 Hz, 1H), 7.89 (dd, *J* = 7.8, 1.6 Hz, 1H), 7.84 (dd, *J* = 7.9, 1.5 Hz, 1H), 7.77 (d, *J* = 8.5 Hz, 1H), 7.62 – 7.50 (m, 3H), 7.22 (dd, *J* = 5.3, 2.0 Hz, 3H), 7.15 (dt, *J* = 6.8, 2.2 Hz, 2H), 3.25 (s, 2H), 1.99 (s, 2H). ^{13}C NMR (101 MHz, $CDCl_3$) δ 196.32, 135.60, 134.93, 132.77, 132.16, 129.72, 129.22, 128.37, 127.90, 127.87, 127.71, 127.19, 126.54, 126.01, 124.40, 38.71, 37.81, 31.42. **HRMS**: calculated for $C_{21}H_{17}O$ [M+H]$^+$ 285.1274; found 285.1285.

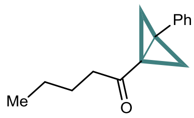

1e

^{1}H NMR (400 MHz, $CDCl_3$) δ 7.42 – 7.15 (m, 5H), 3.01 (s, 2H), 2.05 (t, *J* = 7.2 Hz, 2H), 1.62 (s, 2H), 1.38 – 1.25 (m, 2H), 1.04 (q, *J* = 7.5 Hz, 2H), 0.72 (t, *J* = 7.3 Hz, 3H). ^{13}C NMR (101 MHz, $CDCl_3$) δ 202.87, 133.13, 128.53, 127.05, 125.44, 38.98, 36.60, 35.30, 32.31, 25.48, 22.06, 13.67. **HRMS**: calculated for $C_{15}H_{19}O$ [M+H]$^+$ 215.1430; found 215.1438.

2.3 Procedures for the preparation of 1,3-diene substrates

Procedure I:[3]

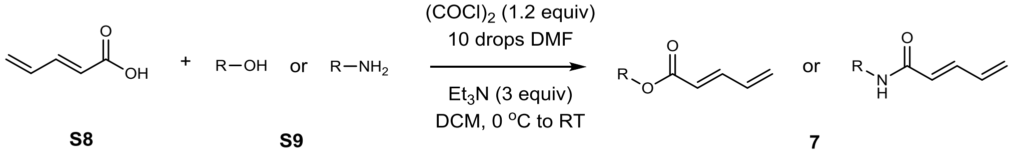

Supplementary Figure 4. Procedure I for the preparation of 1,3-diene substrates

A 50 mL round-bottomed flask immersed in ice bath was charged with (*E*)-penta-2,4-dienoic acid

(5 mmol, 1.0 equiv) and CH_2Cl_2 (20 mL) under N_2 atmosphere. To the stirred solution was added oxalyl chloride (5.5 mmol, 1.1 equiv) dropwise over 10 minutes followed by addition of DMF (10 drops, catalytic amount) in one portion. After 1 hour, raw material phenol/amine (5 mmol, 1 equiv) and Et_3N (15 mmol, 3 equiv) was added. The mixture was stirred for 3 h at room temperature, quenched with H_2O (20 mL) and the organic layer was separated. Then the solvent was evapotated in vacuum. The resulting mixture was purified by flash column chromatography on silica gel to obtain 1,3-diene substrates yield.

Procedure II:[4]

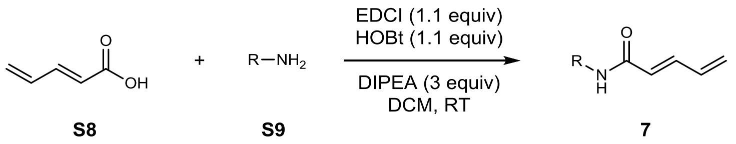

Supplementary Figure 5. Procedure II for the preparation of 1,3-diene substrates

A mixture of anilines **S8** (10 mmol, 1.0 equiv), (*E*)-penta-2,4-dienoic acid **S9** (10 mmol, 1 equiv), EDCI (11 mmol, 1.1 equiv), HOBt (11 mmol, 1.1 equiv), and DIPEA (30 mmol, 3.0 equiv) in anhydrous CH_2Cl_2 (25 mL) was stirred at room temperature overnight. Water was added and the mixture was extracted with CH_2Cl_2. The combined organic layers was washed with water and brine, dried over anhydrous Na_2SO_4, and concentrated in vacuo. The resulting residue was purified by silica gel flash chromatography to give the desired **7** products.

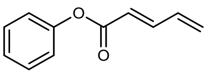

7a

According to the General Procedure I：^{1}H NMR (400 MHz, $CDCl_3$) δ 7.54 – 7.38 (m, 3H), 7.25 (ddt, *J* = 8.5, 7.0, 1.2 Hz, 1H), 7.19 – 7.09 (m, 2H), 6.62 – 6.47 (m, 1H), 6.13 (d, *J* = 15.4 Hz, 1H), 5.70 (dd, *J* = 16.9, 1.2 Hz, 1H), 5.59 (dd, *J* = 9.9, 1.2 Hz, 1H). ^{13}C NMR (101 MHz, $CDCl_3$) δ 165.13, 150.61, 146.48, 134.53, 129.34, 126.63, 125.69, 121.58, 121.52, 121.20. **HRMS**: calculated for $C_{11}H_{11}O_2$ $[M+H]^+$ 175.0754; found 175.0752.

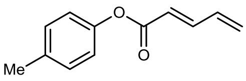

7b

According to the General Procedure I：^{1}H NMR (400 MHz, $CDCl_3$) δ 7.46 (dd, *J* = 15.4, 11.0 Hz, 1H), 7.19 (d, *J* = 8.2 Hz, 2H), 7.10 – 6.98 (m, 2H), 6.54 (ddd, *J* = 16.9, 10.9, 10.0 Hz, 1H), 6.11 (d, *J* = 15.4 Hz, 1H), 5.69 (dd, *J* = 16.9, 1.3 Hz, 1H), 5.58 (dd, *J* = 9.9, 1.3 Hz, 1H), 2.36 (s, 3H). ^{13}C NMR (101 MHz, $CDCl_3$) δ 165.36, 148.37, 146.32, 135.31, 134.57, 129.85, 126.49, 121.30, 121.25, 121.19, 20.81. **HRMS**: calculated for $C_{12}H_{13}O_2$ $[M+H]^+$ 189.0910; found 189.0909.

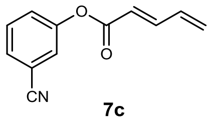

7c

According to the General Procedure I： ^{1}H NMR (400 MHz, $CDCl_3$) δ 7.57 – 7.48 (m, 2H), 7.48 – 7.42 (m, 2H), 7.40 (dt, *J* = 7.7, 2.0 Hz, 1H), 6.55 (dt, *J* = 16.9, 10.5 Hz, 1H), 6.09 (d, *J* = 15.4 Hz, 1H), 5.73 (dd, *J* = 16.9, 1.2 Hz, 1H), 5.62 (dd, *J* = 9.9, 1.1 Hz, 1H). ^{13}C NMR (101 MHz, $CDCl_3$) δ 164.41, 150.73, 147.62, 134.31, 130.30, 129.40, 127.58, 126.59, 125.34, 120.17, 117.83, 113.32. **HRMS**: calculated for $C_{12}H_{10}NO_2$ [M+H] $^+$ 200.0706; found 200.0704.

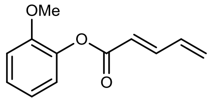

7d

According to the General Procedure I： ^{1}H NMR (400 MHz, $CDCl_3$) δ 7.47 (dd, *J* = 15.4, 11.0 Hz, 1H), 7.28 – 7.18 (m, 1H), 7.09 (dd, *J* = 7.9, 1.7 Hz, 1H), 7.04 – 6.92 (m, 1H), 6.87 (q, *J* = 3.5, 2.7 Hz, 1H), 6.64 – 6.47 (m, 1H), 6.16 (d, *J* = 15.4 Hz, 1H), 5.72 – 5.65 (m, 1H), 5.57 (d, *J* = 10.0 Hz, 1H), 3.83 (s, 3H). ^{13}C NMR (101 MHz, $CDCl_3$) δ 164.74, 151.17, 146.47, 134.65, 126.80, 122.83, 120.97, 120.71, 114.46, 112.37, 55.80. **HRMS**: calculated for $C_{12}H_{13}O_3$ [M+H] $^+$ 205.0859; found 205.0857.

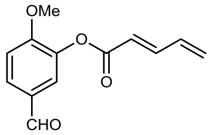

7e

According to the General Procedure I： ^{1}H NMR (400 MHz, $CDCl_3$) δ 9.84 (s, 1H), 7.74 (dd, *J* = 8.4, 1.9 Hz, 1H), 7.60 (d, *J* = 1.9 Hz, 1H), 7.44 (dd, *J* = 15.5, 11.0 Hz, 1H), 7.06 (d, *J* = 8.5 Hz, 1H), 6.52 (dt, *J* = 16.9, 10.5 Hz, 1H), 6.11 (d, *J* = 15.5 Hz, 1H), 5.68 (d, *J* = 16.9 Hz, 1H), 5.57 (d, *J* = 10.1 Hz, 1H), 3.87 (s, 3H). ^{13}C NMR (101 MHz, $CDCl_3$) δ 189.95, 164.24, 156.33, 147.01, 140.02, 134.42, 129.99, 129.79, 126.95, 123.39, 120.27, 111.91, 56.11.**HRMS**: calculated for $C_{13}H_{13}O_4$ [M+H] $^+$ 233.0808; found 233.0806.

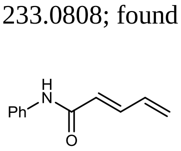

7f

According to the General Procedure II: ^{1}H NMR (400 MHz, $CDCl_3$) δ 8.85 (s, 1H), 7.66 (d, *J* = 7.4 Hz, 2H), 7.42 – 7.25 (m, 3H), 7.11 (t, *J* = 7.4 Hz, 1H), 6.35 (dt, *J* = 16.8, 10.4 Hz, 1H), 6.20 (dd, *J* = 15.5, 6.4 Hz, 1H), 5.59 – 5.18 (m, 2H). ^{13}C NMR (101 MHz, $CDCl_3$) δ 164.80, 141.91, 138.03, 134.56, 134.53, 129.14, 128.74, 124.68, 124.27, 120.41. **HRMS**: calculated for $C_{11}H_{12}NO$ [M+H] $^+$ 174.0913; found 174.0911.

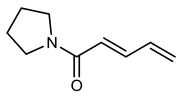

7g

According to the General Procedure II：^{1}H NMR (400 MHz, Chloroform-*d*) δ 7.35 – 7.15 (m, 1H), 6.43 (dt, *J* = 16.9, 10.5 Hz, 1H), 6.18 (d, *J* = 14.9 Hz, 1H), 5.53 (dd, *J* = 16.9, 1.4 Hz, 1H), 5.38 (dd, *J* = 10.0, 1.5 Hz, 1H), 3.50 (td, *J* = 6.9, 2.2 Hz, 4H), 1.94 (p, *J* = 6.7 Hz, 2H), 1.84 (p, *J* = 6.7 Hz, 2H). ^{13}C NMR (101 MHz, $CDCl_3$) δ 164.56, 141.68, 135.08, 123.97, 122.76, 46.39, 45.80, 25.99, 24.21. **HRMS**: calculated for $C_9H_{14}NO$ [M+H] $^+$ 152.1070; found 152.1074.

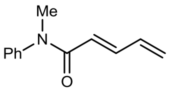

7h

According to the General Procedure II：^{1}H NMR (400 MHz, Chloroform-*d*) δ 7.40 – 7.33 (m, 2H), 7.32 – 7.26 (m, 1H), 7.21 (ddt, *J* = 14.9, 11.0, 0.9 Hz, 1H), 7.14 (q, *J* = 2.1, 1.6 Hz, 1H), 7.12 (t, *J* = 1.3 Hz, 1H), 6.32 – 6.06 (m, 1H), 5.78 (d, *J* = 14.9 Hz, 1H), 5.46 (ddt, *J* = 17.1, 1.7, 0.8 Hz, 1H), 5.27 (ddt, *J* = 10.1, 1.6, 0.8 Hz, 1H), 3.30 (s, 3H). ^{13}C NMR (101 MHz, $CDCl_3$) δ 165.86, 143.40, 141.82, 134.99, 129.42, 127.38, 127.15, 123.83, 122.44, 37.27. **HRMS**: calculated for $C_{12}H_{14}NO$ [M+H] $^+$ 188.1070; found 188.1068.

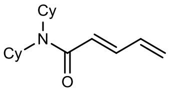

7i

According to the General Procedure II：^{1}H NMR (400 MHz, Chloroform-*d*) δ 7.12 (dd, *J* = 14.7, 11.0 Hz, 1H), 6.43 (dt, *J* = 16.0, 10.3 Hz, 1H), 6.29 (d, *J* = 14.8 Hz, 1H), 5.45 (d, *J* = 16.9 Hz, 1H), 5.32 (d, *J* = 10.0 Hz, 1H), 3.47 (s, 1H), 2.40 – 2.05 (m, 2H), 1.65 (dt, *J* = 67.4, 19.3 Hz, 13H), 1.38 – 0.94 (m, 6H). ^{13}C NMR (101 MHz, $CDCl_3$) δ 166.04, 141.02, 135.36, 124.54, 122.84, 57.37, 55.65, 31.67, 30.10, 26.33, 25.97, 25.23, 25.18. **HRMS**: calculated for $C_{17}H_{28}NO$ [M+H] $^+$ 262.2165; found 262.2163.

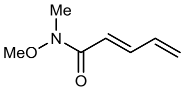

7j

According to the General Procedure I：^{1}H NMR (400 MHz, Chloroform-*d*) δ 7.44 – 7.11 (m, 1H), 6.58 – 6.40 (m, 2H), 5.57 (dt, *J* = 16.9, 1.0 Hz, 1H), 5.43 (dd, *J* = 10.2, 1.3 Hz, 1H), 3.68 (s, 3H), 3.23 (s, 3H). ^{13}C NMR (101 MHz, $CDCl_3$) δ 171.04, 143.42, 135.13, 124.77, 119.74, 61.72, 32.31.**HRMS**: calculated for $C_7H_{12}NO_2$ [M+H] $^+$ 142.0863; found 142.0860.

2.4 Optimization of Reaction Conditions

2.4.1. The Effect of Salen-Ti

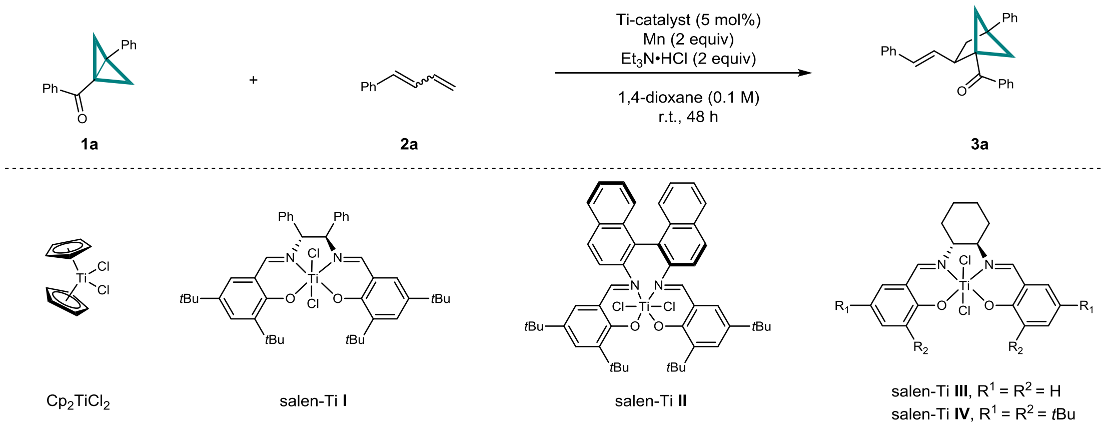

Entry [a]	[**Ti-catalyst**]	yield of **3a** (%)[b]
1	Cp_2TiCl_2	61
2	$CpTiCl_3$ instead of Cp_2TiCl_2	19
3	salen-Ti **I** instead of Cp_2TiCl_2	47
4	salen-Ti **II** instead of Cp_2TiCl_2	58
5	salen-Ti **III** instead of Cp_2TiCl_2	81
6	salen-Ti **IV** instead of Cp_2TiCl_2	91(89)[c]

[a] Reaction conditions: **1a** (0.2 mmol), **2a** (0.3 mmol) and Ti-catalyst (5 mol%) in 1,4-dioxane (0.1 M) reaction for 48 h. [b] Yields were determined by ^{1}H NMR analysis with 1,3,5-trimethoxybenzene as the internal standard.

Supplementary Table 1. The Effect of Salen-Ti

2.4.2 The Effect of Solvents

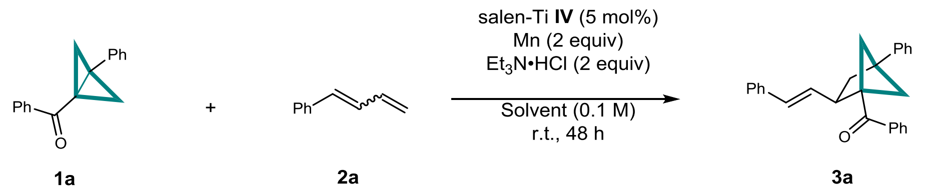

Entry [a]	[**Solvent**]	Yield of **3a** (%)[b]
1	CH_3CN	70
2	DCM	56
3	EtOAc	48
4	THF	83
5	Et_2O	39

[a] Reaction conditions: **1a** (0.2 mmol), **2a** (0.3 mmol) and salen-Ti **IV** (5 mol%) in solvents (0.1 M) reaction for 48 h. [b] Yields were determined by ^{1}H NMR analysis with 1,3,5-trimethoxybenzene as the internal standard.

Supplementary Table 2. The Effect of Solvents

2.5. Control Experiments

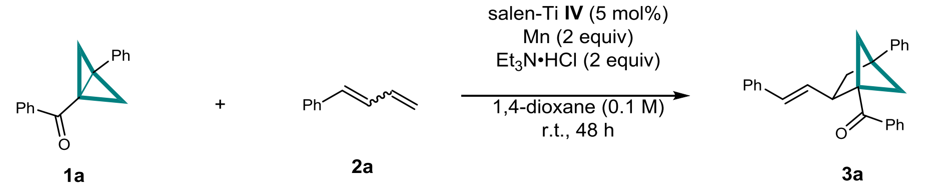

Entry[a]	[**variation from standard conditions**]	Yield of **3a** (%)[b]
1	none	91 (89)[c]
2	2,4,6-collidine•HCl in instead of Et_3N•HCl	26
3	Zn in instead of Mn	83
4	Mn (50 mol%)	44
5	Et_3N•HCl (50 mol%)	53
6	no Et_3N•HCl	17
7	no Mn	0
8	no catalyst	0

[a] Reaction conditions: **1a** (0.2 mmol), **2a** (0.3 mmol) and salen-Ti **IV** (5 mol%) in 1,4-dioxane (0.1 M) reaction for 48 h. [b] Yields were determined by ^{1}H NMR analysis with 1,3,5-trimethoxybenzene as the internal standard. [c] Isolated yield.

Supplementary Table 3. Control Experiments

2.6 Radical Trapping Experiment

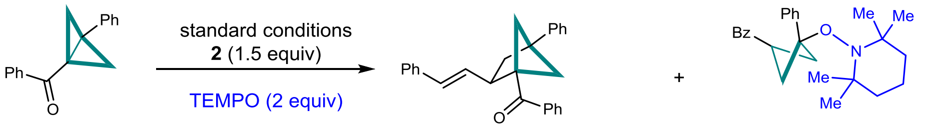

1a 3a 16

Not detected

detected by HRMS [M+H]$^+$
calculated for 392.2584; found 392.2588.

Supplementary Figure 6. Radical Trapping Experiment

In a flame-dried 10 mL reaction tube equipped with a magnetic stirrer bar was added with salen-Ti **IV** (0.05 equiv), Et_3N•HCl (2 equiv), Mn(2 equiv), **1a** (0.2 mmol, 1.0 equiv) and **2a** (1.5 equiv). The Schlenk flask was transferred to an argon-filled glovebox and followed by the addition of 1,4-dioxane (0.1 M). Then the mixture was added TEMPO (0.4 mmol, 2 equiv). Next, the resulting mixture was removed out the glovebox. At last, the reaction mixture was stirred at room temperature for 48 h. (The reaction solution was detected by ^{1}H NMR and HRMS analysis and the **3a** was not found, **16** was detected by HRMS)

HRMS of **16**: calculated for $C_{26}H_{34}NO_2$, [M+H]$^+$ 392.2584; found 392.2588.

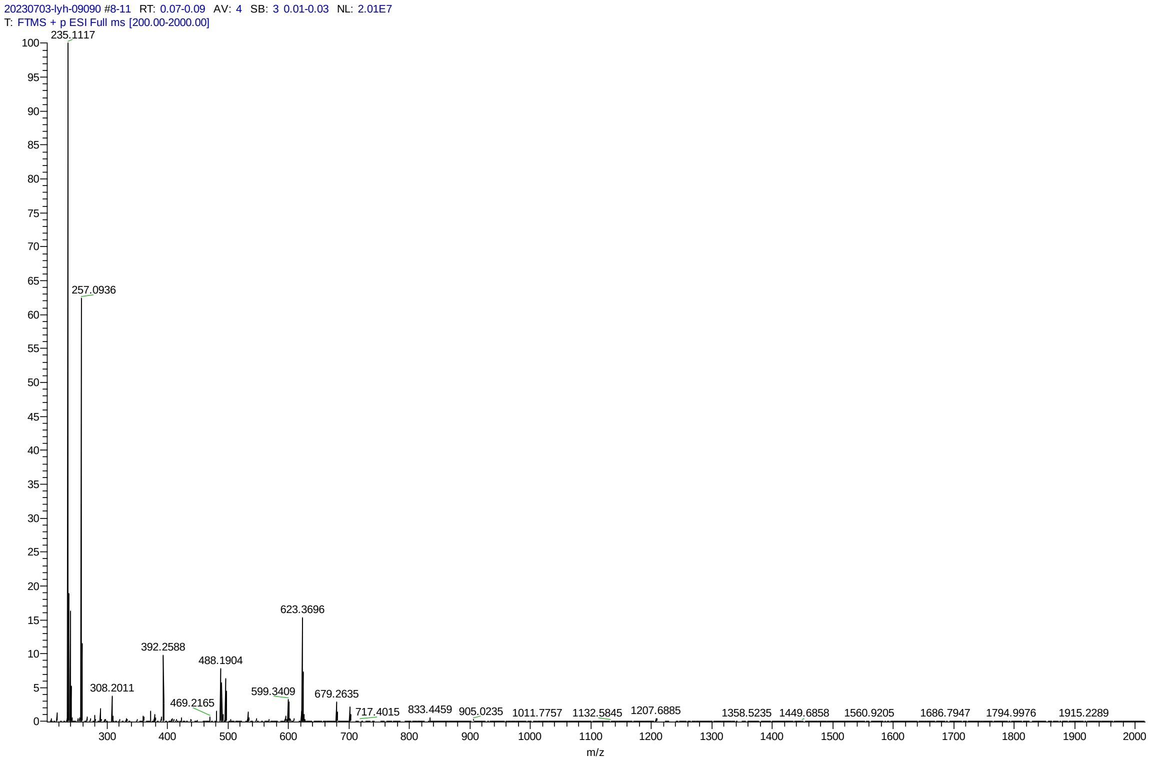

Supplementary Figure 6'. High Resolution Mass Spectrometry of Radical Trapping Experiment

2.7 Gram-Scale Reaction

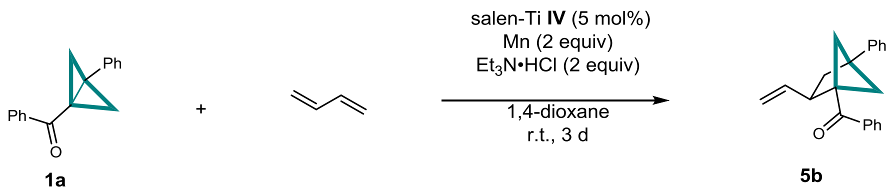

Supplementary Figure 7. Gram-Scale Reaction

In a flame-dried 100 mL reaction tube equipped with a magnetic stirrer bar was added salen-Ti **IV** (0.05 equiv), Et_3N•HCl (2 equiv), Mn (2 equiv) and **1a** (5.77 mmol, 1.35 g, 1 equiv). The Schlenk flask was transferred to an argon-filled glovebox and followed by the addition of 1,4-dioxane (50 mL). Then, to the resulting mixture were added 1,3-Butadiene (2 mol/L in hexane, 2 equiv). Next, the resulting mixture was stirred at room temperature for 3 d in argon-filled glovebox. After completion of the reaction, the resulting mixture was removed out the glovebox, the solvent was evaporated in vacuo and the crude material was purified by flash column chromatography to furnish the desired product **5b** (78%, 1.31 g).

2.8 Prilezhaev epoxidation of 5b

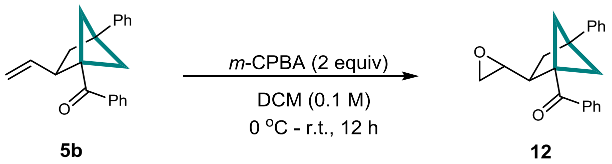

Supplementary Figure 8. Prilezhaev epoxidation of 5b

A solution of **56** (0.5 mmol, 1.0 equiv) in DCM (10 mL) was cooled by an ice bath and *m*-CPBA (1.5 mmol, 3 equiv) was added portion wise. The reaction mixture was allowed to warm to r.t. slowly and stirred vigorously overnight at room temperature, then the reaction was quenched with saturated $Na_2S_2O_3$ solution and extracted with DCM. The concentrated organic layer was dried over anhydrous Na_2SO_4. The organic layer was then concentrated under reduced pressure and the crude material was purified by flash column chromatography (using petroleum ether/EtOAc = 4:1) to provide the **12** as a white soild in 84% yield (128 mg). Rf = 0.2 (PE:EA = 9:1)**.** **^{1}H NMR** (400 MHz, $CDCl_3$) δ 8.06 – 7.84 (m, 2H), 7.59 – 7.51 (m, 1H), 7.50 – 7.43 (m, 2H), 7.39 – 7.32 (m, 2H), 7.31 – 7.21 (m, 3H), 3.08 (ddd, J = 8.2, 4.1, 2.7 Hz, 1H), 2.80 – 2.70 (m, 1H), 2.59 – 2.43 (m, 2H), 2.44 – 2.28 (m, 4H), 2.23 (dt, J = 6.8, 2.2 Hz, 1H), 1.93 (ddd, J = 11.2, 4.2, 1.9 Hz, 1H). **^{13}C NMR** (101 MHz, $CDCl_3$) δ 201.45, 141.72, 136.79, 132.70, 128.45, 128.36, 128.31, 126.60, 125.84, 58.76, 52.59, 50.21, 49.45, 48.82, 46.14, 42.30, 37.65. **HRMS**: calculated for $C_{21}H_{21}O_2$, $[M+H]^+$ 305.1536; found 305.1536.

2.9 Reduction of 5b

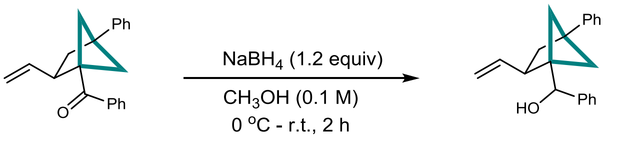

5b **13**, 98%, dr = 2:1

Supplementary Figure 9. Reduction of 5b

To a solution of **5b** (0.5 mmol, 1.0 equiv) in CH_3OH (5 mL) was added $NaBH_4$ (0.6 mmol, 1.2 equiv) at 0 °C. The reaction mixture was stirred for 2 h at room temperature. After completion of the reaction, the reaction was cooled to 0 °C and saturated NH_4Cl (5 mL) solution and H_2O (10 mL) were aedded. The layers were separated and the aqueous layer extracted with DCM (50 mL x 3). The combined organic layers were washed with brine (20 mL), dried with Na_2SO_4, and concentrated in *vacuo* to yield the crude material. The crude material was purified by flash column chromatography (using petroleum ether/EtOAc = 10:1) to provide the compound **13** as a colorless oil in 98% (dr = 2:1) yield (142 mg). Rf = 0.2 (PE:EA = 8:1). **^{1}H NMR** (400 MHz, $CDCl_3$) Main product: δ 7.45 – 7.25 (m, 8H), 7.21 (dd, *J* = 12.3, 7.2 Hz, 2H), 6.26 – 5.81 (m, 1H), 5.39 – 5.07 (m, 2H), 4.87 (d, *J* = 31.5 Hz, 1H), 3.02 (td, *J* = 9.0, 3.7 Hz, 1H), 2.32 – 2.12 (m, 2H), 2.07 – 1.97 (m, 1H), 1.91 (dd, *J* = 9.1, 6.9 Hz, 1H), 1.84 – 1.70 (m, 1H), 1.71 – 1.60 (m, 1H), 1.45 – 1.27 (m, 1H).**^{1}H NMR** (400 MHz, $CDCl_3$) Another diastereomeric: δ 2.43 (td, *J* = 9.2, 3.7 Hz, 1H). **^{13}C NMR** (101 MHz, $CDCl_3$) δ 143.43, 143.36, 142.66, 141.55, 140.56, 140.46, 128.11, 128.07, 127.97, 127.93, 127.52, 127.16, 126.69, 126.35, 126.02, 125.86, 125.63, 116.14, 74.26, 73.54, 56.39, 55.77, 50.32, 49.05, 46.49, 45.10, 43.33, 43.04, 42.43, 42.21, 40.54, 38.83. **HRMS**: calculated for $C_{21}H_{23}O$ $[M+H]^+$ 291.1743; found 291.1749.

2.10 Wacker-type oxidation of 5b

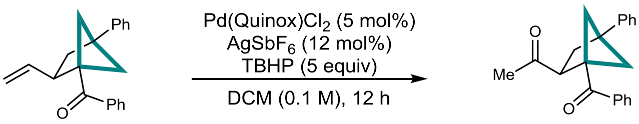

5b **14**, 68%

Supplementary Figure 10. Wacker-type oxidation of 5b

In a flame-dried 15 mL reaction tube equipped with a magnetic stirrer bar in DCM (2 mL) was added $AgSbF_6$ (0.12 equiv) and $Pd(Quinox)Cl_2$ (0.05 equiv) at room temperature stirred for 15 min under N_2 protection. Then, to the resulting mixture was added TBHP (5 equiv) and **5b** (0.2 mmol, 1 equiv). Next, the resulting mixture was stirred at room temperature for 12 h under N_2 protection. After completion of the reaction, the mixture was filtered by silicone gasket. The solvent was evaporated in vacuo and the crude material was purified by flash column chromatography to furnish the desired product **14** as a colorless oil in 68% yield (41 mg). Rf = 0.3 (PE:EA = 8:1). **^{1}H NMR** (400 MHz, $CDCl_3$) δ 7.94 – 7.77 (m, 2H), 7.64 – 7.51 (m, 1H), 7.51 – 7.41 (m, 2H), 7.39 – 7.29 (m, 2H), 7.29 – 7.12 (m, 3H), 3.78 (ddd, *J* = 9.4, 4.6, 1.5 Hz, 1H), 2.41 (ddd, *J* = 10.6, 9.2, 2.5 Hz, 1H), 2.34 – 2.25 (m, 5H), 2.07 (s, 3H). **^{13}C NMR** (101 MHz, $CDCl_3$) δ 208.41, 201.51, 141.47, 137.25, 132.61, 128.47, 128.39, 128.37, 126.69,

125.81, 57.93, 57.23, 50.66, 49.76, 42.88, 37.68, 29.67. **HRMS**: calculated for $C_{21}H_{21}O_2$, $[M+H]^+$ 305.1536; found 305.1532.

2.11 Photocatalytic reaction of disulfide with 5b

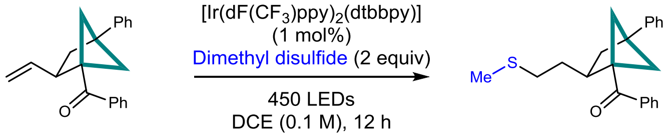

5b **15**, 60%

Supplementary Figure 11. Photocatalytic reaction of disulfide with 5b

In a flame-dried 15 mL reaction tube equipped with a magnetic stirrer bar in DCE (3 mL) was added **5b** (0.3 mmol, 1 equiv) and [Ir(dF(CF$_3$)ppy)$_2$(dtbbpy)] (1 mol%) at room temperature under N_2 protection. Then, to the resulting mixture was added dimethyl disulfide (2 equiv). Next, the reaction mixture was stirred at room temperature with irradiation of 5 W 450 nm LED for 12 h under N_2 protection. After completion of the reaction, the solvent was evaporated in vacuo and the crude material was purified by flash column chromatography to furnish the desired product **15** as a colorless oil in 60% yield (60 mg)**.** Rf = 0.6 (PE:EA = 10:1). **^{1}H NMR** (400 MHz, $CDCl_3$) δ 7.98 – 7.90 (m, 2H), 7.60 – 7.53 (m, 1H), 7.47 (dd, J = 8.4, 7.0 Hz, 2H), 7.38 – 7.30 (m, 2H), 7.29 – 7.20 (m, 3H), 2.88 (tt, J = 8.3, 4.6 Hz, 1H), 2.53 – 2.27 (m, 5H), 2.20 (dd, J = 9.0, 7.2 Hz, 1H), 2.13 (ddd, J = 7.3, 2.8, 1.4 Hz, 1H), 1.93 (s, 3H), 1.70 (tdd, J = 12.8, 6.2, 3.6 Hz, 3H). **^{13}C NMR** (101 MHz, $CDCl_3$) δ 202.05, 142.34, 136.61, 132.91, 128.58, 128.34, 128.29, 126.43, 125.82, 59.48, 50.92, 49.74, 44.66, 42.01, 41.26, 32.34, 31.62, 15.13. **HRMS**: calculated for $C_{22}H_{25}OS$, $[M+H]^+$ 337.1621; found 337.1616.

2.12 Visible light promoted E→Z isomerization of alkene 3r

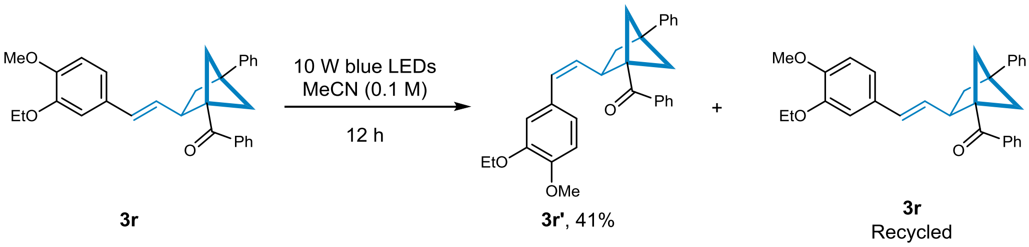

3r **3r'**, 41% **3r** Recycled

Supplementary Figure 12. Visible light promoted E→Z isomerization of alkene 3r

To a solution of CH_3CN (1 mL) was added **3r** (0.1 mmol, 1.0 equiv) under N_2 protection. The reaction mixture was stirred at room temperature with irradiation of 10 W 400 nm LEDs for 12 h. Then, the solvent was evaporated in vacuo and the crude material was purified by flash column chromatography to furnish the desired product **3r'** as a white solid in 41% yield (18 mg). Rf = 0.29 (PE:EA = 6:1). ^{1}H NMR (400 MHz, $CDCl_3$) δ 7.87 – 7.74 (m, 2H), 7.51 – 7.47 (m, 1H), 7.38 – 7.32 (m, 4H), 7.26 (ddd, J = 13.4, 7.9, 1.4 Hz, 3H), 6.70 (d, J = 8.2 Hz, 1H), 6.59 (dd, J = 8.3, 1.5 Hz, 1H), 6.55 (d, J = 1.6 Hz, 1H), 6.36 (d, J = 11.5 Hz, 1H), 5.75 (dd, J = 11.4, 9.9 Hz, 1H), 4.02 – 3.92 (m, 2H),3.89 – 3.85 (m, 4H), 2.53 (ddd, J = 11.2, 8.9, 2.7 Hz, 1H), 2.45 – 2.36 (m, 1H), 2.33 (dd, J = 8.0, 4.5 Hz, 2H), 2.23 – 2.14 (m, 1H), 2.01 – 1.89 (m, 1H), 1.44 (t, J = 7.0 Hz, 3H). ^{13}C NMR (101 MHz, $CDCl_3$) δ 201.72, 147.97,

147.62, 142.04, 136.57, 132.62, 131.39, 130.38, 129.91, 128.33, 128.29, 128.25, 126.49, 125.87, 120.78, 113.03, 110.89, 64.03, 60.08, 55.85, 49.81, 49.63, 43.81, 43.69, 42.67, 14.77. **HRMS**: calculated for $C_{30}H_{31}O_3$ $[M+H]^+$ 439.2268; found 439.2275.

2.13 Amide conversion to ketone

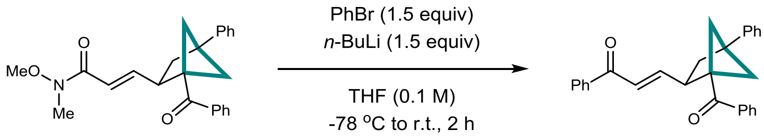

9j **9k**, 95%

Supplementary Figure 13. Amide conversion to ketone

To a solution of dry THF (2 mL) was added PhBr (0.3 mmol, 1.5 equiv) and *n*-BuLi (1.6 M in THF, 0.2 mL, 1.5 equiv) under argon at -78 °C. The mixture stirred for 3 h at same conditions. Then, **9j** (0.2 mmol, 1 equiv) was added and move the reaction to room temperature stirred for 1 h. Then, saturated ammonium chloride (2 ml) was added to the solution. The resulting mixture was extracted with ethyl acetate for 3 times, and washed with saturated brine water. The combined organic layers were dried over magnessium sulfate, filtered and concentrated in vacuum. The crude material was purified by flash column chromatography to furnish the desired product**. 9k**, Rf = 0.4 (PE:EA = 10:1). The crude material was purified by flash column chromatography (using petroleum ether/EtOAc = 10:1) to provide the title compound as a white solid in 95% yield (75 mg)**.** ^{1}**H NMR** (400 MHz, $CDCl_3$) δ 7.94 – 7.86 (m, 2H), 7.80 – 7.70 (m, 2H), 7.60 – 7.50 (m, 2H), 7.50 – 7.32 (m, 6H), 7.32 – 7.23 (m, 3H), 7.04 (dd, J = 15.4, 8.9 Hz, 1H), 6.69 (d, J = 15.4 Hz, 1H), 3.60 (td, J = 8.9, 3.8 Hz, 1H), 2.54 (ddd, J = 11.4, 8.6, 2.9 Hz, 1H), 2.48 – 2.33 (m, 3H), 2.28 (ddd, J = 7.4, 2.9, 1.6 Hz, 1H), 2.07 (ddd, J = 11.3, 3.9, 2.4 Hz, 1H). ^{13}**C NMR** (101 MHz, $CDCl_3$) δ 201.13, 190.66, 148.65, 141.56, 137.59, 136.88, 133.06, 132.77, 128.72, 128.62, 128.51, 128.37, 127.10, 126.79, 125.92, 60.26, 50.33, 50.30, 48.39, 42.45, 41.58. **HRMS**: calculated for $C_{28}H_{25}O_2$ $[M+H]^+$ 393.1849; found 393.1846.

2.14 The X-ray crystallographic data for compound 3a

Crystallization procedure: crystals suitable for X-ray crystallographic analysis were obtained by dissolving the white solids **3a** (25 mg) in a mixed solvents ($V_{toluene}$: $V_{n\text{-}hexane}$ = 2 mL : 6 mL). The solvent was allowed to slowly evaporate over 48 hours to obtain needle crystals.

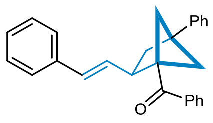

3a

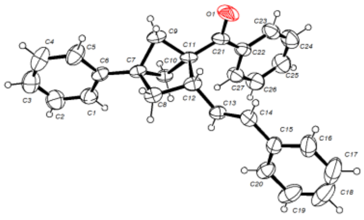

CCDC: 2286139

Supplementary Figure 14. The X-ray crystallographic data for compound 3a

Testing instrument: D8 Venture, Brock, Germany
Instrument type: D8 Venture
Instrument parameters:
Light source: Mo target　　X ray：Mo-Ka(=0.71073 Å)
Detector: CMOS surface detector　　Resolution：0.80Å
Current and voltage：50 kV，1.2 mA　　Exposure time：30 s
Distance from surface detector to sample：40 mm　　Test temperature：150(2)K

Structural analysis and finishing process:
After integral reduction of diffraction data by SAINT program, empirical absorption correction was performed by SADABS program. The single crystal structure was analyzed by direct method using SHELXT2014, and the structure was refined by least square method. The hydrogen atom was obtained by isotropic calculation during the finishing process, and the hydrogen atom on C-H was obtained by calculation and hydrogenation, and it was refined by riding model.

Supplementary Table 4. Crystal data and structure refinements for 3a

Empirical formula	$C_{27}H_{24}O$
Formula weight	364.46
Temperature [K]	293(2)
Crystal system	monoclinic
Space group	P21/n
a/Å	15.7223(5)
b/Å	6.3962(2)
c/Å	20.9604(6)
α/°	90
β/°	101.251(3)
γ/°	90
Volume/Å^3	2067.33(11)
Z	4
ρcalc/g cm^{-3}	1.171
μ/mm−1	0.532
F(000)	776.0
Crystal size/mm3	0.22 ×0.2 ×0.18
Radiation	Cu Kα (λ = 1.54184)

2Θ range for data collection/ °	7.81 to 142.54
Index ranges	-18 ≤ h ≤ 19,<br>-4 ≤ k ≤ 7,<br>-22 ≤ l ≤ 25
Reflections collected	8086
Independent reflections	3909<br>[Rint = 0.0205]
Data/restraints/parameters	3909/0/253
Goodness−of−fit on F2	1.180
Final R indexes [I>=2σ (I)]	R_1 = 0.0622,<br>wR_2 = 0.1534
Final R indexes [all data]	R_1 = 0.0774,<br>wR_2 = 0.1607
Largest diff. peak/hole / e $Å^{-3}$	0.14/-0.15
CCDC	2286139

Supplementary Table 5. Bond Lengths [Å] for 3a

O1-C21	1.214(3)	C12-C13	1.491(3)
C1-C2	1.379(4)	C13-C14	1.313(3)
C1-C6	1.378(4)	C14-C15	1.460(3)
C2-C3	1.361(5)	C15-C16	1.390(4)
C3-C4	1.361(5)	C15-C20	1.392(4)
C4-C5	1.387(4)	C16-C17	1.384(6)
C5-C6	1.378(3)	C17- C18	1.358(8)
C6-C7	1.494(3)	C18-C19	1.371(7)
C7-C8	1.539(3)	C19-C20	1.379(4)
C7-C9	1.550(3)	C21-C22	1.488(4)
C7-C10	1.546(3)	C22-C23	1.385(4)
C8-C12	1.554(3)	C22-C27	1.379(4)
C9-C11	1.547(3)	C23-C24	1.371(4)
C10-C11	1.549(3)	C24-C25	1.370(5)
C11-C12	1.559(3)	C25-C26	1.370(5)
C11-C21	1.497(3)	C26-C27	1.388(4)

Supplementary Table 6. Bond Angles [o] for 3a

C6-C1-C2	121.6(3)	C13-C12-C8	115.0(2)
C3-C2-C1	120.1(3)	C13-C12-C11	114.60(18)
C2-C3-C4	119.4(3)	C14-C13-C12	125.7(2)
C3-C4-C5	120.9(3)	C13-C14-C15	127.3(2)
C6-C5-C4	120.5(3)	C16-C15-C14	119.5(3)
C1-C6-C5	117.6(3)	C16-C15-C20	118.0(3)

C1-C6-C7	120.9(2)	C20-C15-C14	122.5(2)
C5-C6-C7	121.5(2)	C17-C16-C15	119.9(5)
C6-C7-C8	116.9(2)	C18-C17-C16	121.2(5)
C6-C7- C9	123.49(19)	C17-C18-C19	119.8(5)
C6-C7-C10	122.37(19)	C18- C19- C20	120.0(5)
C8-C7-C9	101.20(19)	C19-C20-C15	121.0(4)
C8-C7-C10	100.93(17)	O1-C21-C11	117.3(2)
C10-C7-C9	85.83(17)	O1-C21-C22	119.4(3)
C7-C8-C12	100.58(18)	C22-C21-C11	123.3(2)
C11-C9-C7	83.19(16)	C23-C22-C21	117.6(3)
C7-C10-C11	83.25(16)	C27-C22-C21	123.6(2)
C9-C11-C10	85.83(17)	C27-C22-C23	118.8(3)
C9-C11-C12	100.72(18)	C24-C23-C22	120.4(3)
C10-C11-C12	102.56(17)	C25-C24-C23	120.5(3)
C21-C11-C9	118.91(18)	C26-C25-C24	120.1(3)
C21-C11-C10	128.38(19)	C25-C26-C27	119.5(3)
C21-C11-C12	114.4(2)	C22-C27-C26	120.7(3)
C8-C12-C11	98.06(18)		

2.15 Computational Details

DFT calculations were performed with Gaussian 16, Revision C.01[5]. Geometries for intermediates and transition states were optimized in the gas phase using the ωB97XD[6] functional and with the def2-SVP[7] basis set. Frequencies were computed at the same level of theory to determine whether the structures are minima (with no imaginary frequency) or transition states (with only one imaginary frequency) and to obtain zero-point energy and thermal energy corrections under 298.15 K and 1 atm pressure. Single-point solvation energies were calculated using the ωB97XD functional and with the def2-TZVPP basis set and SMD solvation model[8] (solvent = 1,4-dioxane). Grimme's correction for entropy[9] were performed using Goodvibes v3.0.1[10].

Please note that the Cartesian Coordinate data in Supplementary Data file.

2.16 General Procedure and Characterization Data

In a flame-dried 10 mL reaction tube equipped with a magnetic stirrer bar was charged sequentially with salen-Ti **IV** (0.05 equiv), Mn (2.0 equiv), Et_3N•HCl (2.0 equiv). The Schlenk flask was transferred to an argon-filled glovebox and followed by the addition of 1,4-dioxane (2 mL). Then the mixture was stirred at room temperature for 30 min in argon-filled glovebox. Then, to the resulting mixture were added 1,3-dienes (0.3 mmol, 1.5 equiv) and BCB ketones **1** (0.2 mmol, 1.0 equiv). Next, the resulting mixture was removed out the glovebox and stirred at room temperature for 48 h. After completion of the reaction (TLC), the mixture was filtered by silicone gasket. The solvent was evaporated in vacuo and the crude material was purified by flash column chromatography to furnish the desired product.

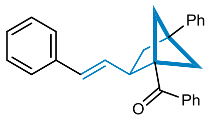

3a

3a, Rf = 0.5 (PE:EA = 7:1). The crude material was purified by flash column chromatography (using petroleum ether/EtOAc = 10:1) to provide the title compound as a white soild in 89% yield (65 mg). **^{1}H NMR** (400 MHz, $CDCl_3$) δ 7.94 – 7.87 (m, 2H), 7.58 – 7.53 (m, 1H), 7.46 (dd, *J* = 8.3, 6.9 Hz, 2H), 7.40 – 7.34 (m, 2H), 7.34 – 7.29 (m, 2H), 7.29 – 7.21 (m, 5H), 7.19 (ddd, *J* = 8.2, 4.5, 2.3 Hz, 1H), 6.35 – 6.17 (m, 2H), 3.53 (ddd, *J* = 8.7, 3.8, 1.5 Hz, 1H), 2.53 (ddd, *J* = 11.3, 8.6, 2.8 Hz, 1H), 2.46 – 2.34 (m, 3H), 2.23 (ddd, *J* = 7.3, 2.9, 1.6 Hz, 1H), 2.04 (ddd, *J* = 11.1, 3.8, 2.6 Hz, 1H). **^{13}C NMR** (101 MHz, $CDCl_3$) δ 201.73, 142.12, 137.01, 132.68, 131.39, 130.40, 128.48, 128.36, 128.30, 127.20, 126.52, 126.17, 125.91, 60.34, 50.25, 49.84, 48.81, 42.31, 41.95. **^{13}C NMR-DEPT 135** (101 MHz, $CDCl_3$) δ 132.76, 131.47, 130.48, 128.56, 128.44, 128.38, 127.28, 126.60, 126.24, 125.99, 50.33, 48.89, 42.39, 42.03. **HRMS**: calculated for $C_{27}H_{24}NaO$ $[M+Na]^+$ 387.1725; found 387.1723.

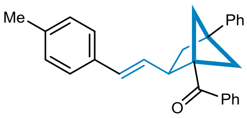

3b

3b, Rf = 0.3 (PE:EA = 10:1). The crude material was purified by flash column chromatography (using petroleum ether/EtOAc = 10:1) to provide the title compound as a white soild in 85% yield (64 mg). **^{1}H NMR** (400 MHz, $CDCl_3$) δ 7.95 – 7.88 (m, 2H), 7.60 – 7.52 (m, 1H), 7.46 (dd, *J* = 8.3, 6.8 Hz, 2H), 7.42 – 7.35 (m, 2H), 7.35 – 7.23 (m, 3H), 7.15 (d, *J* = 8.0 Hz, 2H), 7.07 (d, *J* = 7.9 Hz, 2H), 6.36 – 6.14 (m, 2H), 3.53 (dtt, *J* = 8.6, 4.2, 1.8 Hz, 1H), 2.53 (ddd, *J* = 11.3, 8.6, 2.8 Hz, 1H), 2.47 – 2.35 (m, 3H), 2.32 (s, 3H), 2.23 (ddd, *J* = 7.2, 2.9, 1.6 Hz, 1H), 2.04 (dt, *J* = 11.2, 3.4 Hz, 1H). **^{13}C NMR** (101 MHz, $CDCl_3$) δ 201.78, 142.17, 137.04, 136.95, 134.22, 132.62, 131.22, 129.31, 129.04, 128.44, 128.33, 128.28, 126.48, 126.05, 125.90, 60.33, 50.24, 49.81, 48.82, 42.28, 41.95, 21.10. **HRMS**: calculated for $C_{28}H_{27}O$ $[M+H]^+$ 379.2056; found 379.2062.

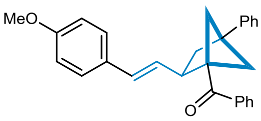

3c

3c, Rf = 0.4 (PE:EA = 7:1). The crude material was purified by flash column chromatography (using petroleum ether/EtOAc = 10:1) to provide the title compound as a white soild in 82% yield (65 mg). **^{1}H NMR** (400 MHz, $CDCl_3$) δ 7.97 – 7.84 (m, 2H), 7.60 – 7.51 (m, 1H), 7.45 (dd, *J* = 8.3, 6.9 Hz, 2H), 7.39 – 7.33 (m, 2H), 7.32 – 7.22 (m, 3H), 7.19 – 7.13 (m, 2H), 6.85 – 6.72 (m, 2H), 6.27 – 6.05 (m, 2H), 3.78 (s, 3H), 3.50 (tdd, *J* = 8.4, 3.9, 1.5 Hz, 1H), 2.50 (ddd, *J* = 11.3, 8.6, 2.8 Hz, 1H), 2.45 – 2.32 (m, 3H), 2.21 (ddd, *J* = 7.1, 2.9, 1.5 Hz, 1H), 2.01 (ddd, *J* = 11.1, 3.9, 2.7 Hz, 1H). **^{13}C NMR** (101 MHz, $CDCl_3$) δ201.86, 158.90, 142.22, 137.07, 132.64, 130.76, 129.85, 128.46, 128.35, 128.31, 128.19, 127.30, 126.49, 125.92, 113.77, 60.38, 55.24, 50.26, 49.82, 48.87, 42.31, 42.03. **HRMS**: calculated for $C_{28}H_{27}O_2$ [M+H] $^{+}$ 395.2006; found 395.2010.

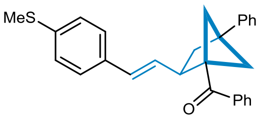

3d

3d, Rf = 0.35 (PE:EA = 4:1). The crude material was purified by flash column chromatography (using petroleum ether/EtOAc = 10:1) to provide the title compound as a white soild in 85% yield (70 mg). **^{1}H NMR** (400 MHz, $CDCl_3$) δ 8.03 – 7.81 (m, 2H), 7.58 – 7.52 (m, 1H), 7.48 – 7.41 (m, 2H), 7.39 – 7.33 (m, 2H), 7.33 – 7.22 (m, 3H), 7.18 – 7.10 (m, 4H), 6.35 – 6.13 (m, 2H), 3.65 – 3.38 (m, 1H), 2.51 (ddd, *J* = 11.3, 8.6, 2.8 Hz, 1H), 2.46 (s, 3H), 2.43 – 2.30 (m, 3H), 2.22 (ddd, *J* = 7.3, 3.0, 1.6 Hz, 1H), 2.02 (ddd, *J* = 11.1, 3.9, 2.6 Hz, 1H). **^{13}C NMR** (101 MHz, $CDCl_3$) δ 201.74, 142.10, 137.21, 137.02, 134.04, 132.68, 130.74, 129.89, 128.48, 128.36, 128.29, 126.58, 126.52, 125.90, 60.34, 50.22, 49.84, 48.84, 42.34, 41.95, 15.89. **HRMS**: calculated for $C_{28}H_{27}OS$ [M+H] $^{+}$ 411.1777; found 411.1784.

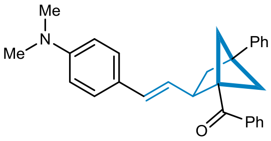

3e

3e, Rf = 0.4 (PE:EA = 6:1). The crude material was purified by flash column chromatography (using petroleum ether/EtOAc = 10:1) to provide the title compound as a white soild in 78% yield (64 mg). **^{1}H NMR** (400 MHz, $CDCl_3$) δ 7.93 – 7.87 (m, 2H), 7.58 – 7.50 (m, 1H), 7.49 – 7.42 (m, 2H), 7.41 – 7.35 (m, 2H), 7.34 – 7.30 (m, 2H), 7.29 – 7.23 (m, 1H), 7.18 – 7.11 (m, 2H), 6.77 – 6.53 (m, 2H), 6.19 (d, *J* = 15.7 Hz, 1H), 6.06 (dd, *J* = 15.7, 8.3 Hz, 1H), 3.61 – 3.45 (m, 1H), 2.93 (s, 6H), 2.51 (ddd, *J* = 11.3, 8.6, 2.8 Hz, 1H), 2.46 – 2.29 (m, 3H), 2.21 (ddd, *J* = 6.9, 2.9, 1.6 Hz, 1H), 2.03 (ddd, *J* = 11.1, 3.9, 2.7 Hz, 1H). **^{13}C NMR** (101 MHz, $CDCl_3$) δ 201.96, 149.79, 142.35, 137.12, 132.52, 131.14, 128.39, 128.31, 127.05, 126.41, 126.02, 125.91, 125.68, 112.35, 60.40, 50.30, 49.76, 48.97, 42.25, 42.11, 40.52. **HRMS**: calculated for $C_{29}H_{30}NO$ [M+H] $^{+}$ 408.2322; found 408.2323.

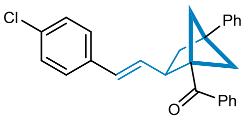

3f

3f, Rf = 0.3 (PE:EA = 10:1). The crude material was purified by flash column chromatography (using petroleum ether/EtOAc = 10:1) to provide the title compound as a white soild in 81% yield (64 mg). **^{1}H NMR** (400 MHz, $CDCl_3$) δ 7.98 – 7.83 (m, 2H), 7.60 – 7.52 (m, 1H), 7.46 (dd, J = 8.3, 6.9 Hz, 2H), 7.36 (d, J = 7.4 Hz, 2H), 7.34 – 7.24 (m, 3H), 7.21 (d, J = 8.5 Hz, 2H), 7.18 – 7.11 (m, 2H), 6.23 (d, J = 6.5 Hz, 2H), 3.63 – 3.40 (m, 1H), 2.52 (ddd, J = 11.3, 8.6, 2.8 Hz, 1H), 2.47 – 2.31 (m, 3H), 2.27 – 2.18 (m, 1H), 2.10 – 1.97 (m, 1H). **^{13}C NMR** (101 MHz, $CDCl_3$) δ 201.63, 141.99, 136.97, 135.48, 132.75, 131.15, 130.20, 128.49, 128.37, 128.27, 127.36, 126.56, 125.89, 60.31, 50.16, 49.86, 48.77, 42.37, 41.89. **HRMS**: calculated for $C_{27}H_{24}ClO$ $[M+H]^{+}$ 399.1510; found 399.1513.

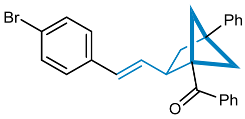

3g

3g, Rf = 0.5 (PE:EA = 8:1). The crude material was purified by flash column chromatography (using petroleum ether/EtOAc = 10:1) to provide the title compound as a white soild in 78% yield (69 mg). **^{1}H NMR** (400 MHz, $CDCl_3$) δ 7.99 – 7.81 (m, 2H), 7.61 – 7.50 (m, 1H), 7.45 (dd, J = 8.4, 6.9 Hz, 2H), 7.40 – 7.33 (m, 4H), 7.33 – 7.24 (m, 3H), 7.12 – 7.05 (m, 2H), 6.38 – 6.05 (m, 2H), 3.61 – 3.44 (m, 1H), 2.51 (ddd, J = 11.3, 8.6, 2.8 Hz, 1H), 2.46 – 2.29 (m, 3H), 2.22 (ddd, J = 7.2, 3.0, 1.6 Hz, 1H), 2.02 (ddd, J = 11.2, 3.9, 2.6 Hz, 1H). **^{13}C NMR** (101 MHz, $CDCl_3$) δ 201.63, 141.98, 136.96, 135.92, 132.76, 131.43, 131.29, 130.25, 128.51, 128.38, 128.27, 127.70, 126.56, 125.89, 120.91, 60.29, 50.16, 49.86, 48.78, 42.37, 41.87. **HRMS**: calculated for $C_{27}H_{24}BrO$ $[M+H]^{+}$ 443.1005; found 443.1010.

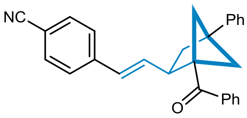

3h

3h, Rf = 0.3 (PE:EA = 7:1). The crude material was purified by flash column chromatography (using petroleum ether/EtOAc = 7:1) to provide the title compound as a white soild in 73% yield (57 mg). **^{1}H NMR** (400 MHz, $CDCl_3$) δ 7.93 – 7.84 (m, 2H), 7.61 – 7.49 (m, 3H), 7.46 (dd, J = 8.4, 6.9 Hz, 2H), 7.41 – 7.33 (m, 2H), 7.33 – 7.22 (m, 5H), 6.40 (dd, J = 15.8, 8.5 Hz, 1H), 6.26 (d, J = 15.8 Hz, 1H), 3.54 (td, J = 8.6, 3.6 Hz, 1H), 2.54 (ddd, J = 11.3, 8.6, 2.8 Hz, 1H), 2.47 – 2.38 (m, 2H), 2.35 (dd, J = 8.7, 7.3 Hz, 1H), 2.23 (ddd, J = 7.3, 2.9, 1.5 Hz, 1H), 2.04 (ddd, J = 11.2, 3.9, 2.6 Hz, 1H). **^{13}C NMR** (101 MHz, $CDCl_3$) δ 201.36, 141.71, 141.40, 136.81, 134.69, 132.87, 132.20, 129.90, 128.56, 128.39, 128.22, 126.63, 126.61, 125.84, 118.95, 110.34, 60.29, 50.08, 49.90, 48.75, 42.41, 41.81. **HRMS**: calculated for $C_{28}H_{24}NO$ $[M+H]^{+}$ 390.1852; found 390.1862.

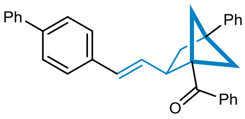

3i

3i, Rf = 0.3 (PE:EA = 10:1). The crude material was purified by flash column chromatography (using petroleum ether/EtOAc = 10:1) to provide the title compound as a white soild in 70% yield (62 mg). **^{1}H NMR** (400 MHz, $CDCl_3$) δ 7.99 – 7.85 (m, 2H), 7.63 – 7.54 (m, 3H), 7.54 – 7.42 (m, 6H), 7.42 – 7.23 (m, 8H), 6.42 – 6.19 (m, 2H), 3.56 (tdd, J = 5.7, 3.8, 1.9 Hz, 1H), 2.55 (ddd, J = 11.3, 8.6, 2.8 Hz, 1H), 2.50 – 2.35 (m, 3H), 2.25 (ddd, J = 7.2, 2.8, 1.6 Hz, 1H), 2.07 (ddd, J = 10.9, 3.9, 2.6 Hz, 1H). **^{13}C NMR** (101 MHz, $CDCl_3$) δ 201.72, 142.12, 140.69, 139.96, 137.03, 136.06, 132.70, 130.94,

130.56, 128.71, 128.50, 128.37, 128.31, 127.18, 127.07, 126.86, 126.59, 126.53, 125.92, 60.38, 50.26, 49.87, 48.89, 42.34, 41.99. **HRMS**: calculated for $C_{33}H_{29}O$ [M+H] $^{+}$ 441.2213; found 441.2219.

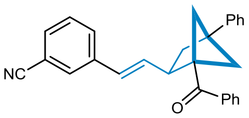

3j

3j, Rf = 0.4 (PE:EA = 6:1). The crude material was purified by flash column chromatography (using petroleum ether/EtOAc = 10:1) to provide the title compound as a white soild in 75% yield (58 mg). **^{1}H NMR** (400 MHz, $CDCl_3$) δ 7.89 (d, J = 7.6 Hz, 2H), 7.61 – 7.54 (m, 1H), 7.51 – 7.41 (m, 5H), 7.40 – 7.22 (m, 6H), 6.32 (dd, J = 15.7, 8.3 Hz, 1H), 6.22 (d, J = 15.7 Hz, 1H), 3.52 (td, J = 8.6, 3.7 Hz, 1H), 2.53 (ddd, J = 11.4, 8.6, 2.8 Hz, 1H), 2.47 – 2.32 (m, 3H), 2.24 (dt, J = 7.1, 2.0 Hz, 1H), 2.03 (dt, J = 11.3, 3.3 Hz, 1H). **^{13}C NMR** (101 MHz, $CDCl_3$) δ 201.46, 141.77, 138.17, 136.88, 133.47, 132.89, 130.46, 130.24, 129.67, 129.28, 129.16, 128.58, 128.39, 128.24, 126.62, 125.86, 118.72, 112.53, 60.30, 50.05, 49.92, 48.71, 42.43, 41.83. **HRMS**: calculated for $C_{28}H_{24}NO$ [M+H] $^{+}$ 390.1852; found 390.1861.

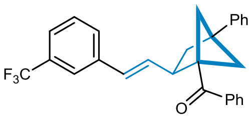

3k

3k, Rf = 0.3 (PE:EA = 10:1). The crude material was purified by flash column chromatography (using petroleum ether/EtOAc = 10:1) to provide the title compound as a white soild in 80% yield (69 mg). **^{1}H NMR** (400 MHz, $CDCl_3$) δ 8.00 – 7.84 (m, 2H), 7.60 – 7.53 (m, 1H), 7.50 – 7.40 (m, 4H), 7.41 – 7.34 (m, 4H), 7.34 – 7.24 (m, 3H), 6.47 – 6.13 (m, 2H), 3.63 – 3.37 (m, 1H), 2.54 (ddd, J = 11.3, 8.6, 2.8 Hz, 1H), 2.49 – 2.34 (m, 3H), 2.25 (ddd, J = 7.0, 2.9, 1.6 Hz, 1H), 2.05 (ddd, J = 11.2, 3.9, 2.6 Hz, 1H). **^{13}C NMR** (101 MHz, $CDCl_3$) δ 201.60, 141.92, 137.73, 136.97, 132.83, 132.54, 130.75 (q, $J_{C\text{-}F}$= 32.1 Hz), 130.09, 129.30, 128.81, 128.54, 128.40, 128.28, 126.60, 125.89, 124.07 (q, $J_{C\text{-}F}$ = 272.4 Hz), 123.74 (q, $J_{C\text{-}F}$ = 3.7 Hz), 122.81 (q, $J_{C\text{-}F}$ = 3.8 Hz), 60.34, 50.07, 49.92, 48.80, 42.47, 41.93. **^{19}F NMR** (377 MHz, $CDCl_3$) δ -62.67. **HRMS**: calculated for $C_{28}H_{24}F_3O$ [M+H] $^{+}$ 433.1774; found 433.1779.

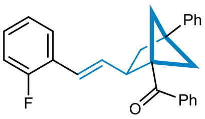

3l

3l, Rf = 0.3 (PE:EA = 10:1). The crude material was purified by flash column chromatography (using petroleum ether/EtOAc = 10:1) to provide the title compound as a white soild in 71% yield (54 mg). **^{1}H NMR** (400 MHz, $CDCl_3$) δ 8.02 – 7.82 (m, 2H), 7.59 – 7.52 (m, 1H), 7.46 (dd, J = 8.2, 6.8 Hz, 2H), 7.41 – 7.23 (m, 6H), 7.20 – 7.12 (m, 1H), 7.05 – 7.01 (m, 1H), 6.97 (ddd, J = 10.8, 8.2, 1.3 Hz, 1H), 6.44 (d, J = 15.9 Hz, 1H), 6.34 (dd, J = 15.9, 8.2 Hz, 1H), 3.55 (tdd, J = 8.5, 4.0, 1.4 Hz, 1H), 2.54 (ddd, J = 11.3, 8.6, 2.9 Hz, 1H), 2.50 – 2.30 (m, 3H), 2.23 (ddd, J = 7.3, 2.9, 1.6 Hz, 1H), 2.06 (ddd, J = 11.2, 3.9, 2.6 Hz, 1H). **^{13}C NMR** (101 MHz, $CDCl_3$) δ 201.69, 159.88 (d, $J_{C\text{-}F}$ = 248.7 Hz), 142.02, 136.98, 133.01 (d, $J_{C\text{-}F}$ = 4.3 Hz), , 132.71, 128.49, 128.40, 128.36, 128.26, 127.22, 127.18, 126.53, 125.90, 124.76 (d, $J_{C\text{-}F}$ = 12.3 Hz), 123.90 (d, $J_{C\text{-}F}$ = 3.6 Hz), 123.78 (d, $J_{C\text{-}F}$ = 3.6 Hz), 115.57, 115.35, 60.34, 50.16, 49.84, 49.13, 42.35, 41.88. **^{19}F NMR** (377 MHz, $CDCl_3$) δ -118.46. **HRMS**: calculated for $C_{27}H_{24}FO$ [M+H] $^{+}$ 383.1806; found 383.1812.

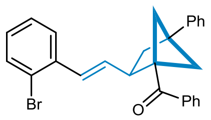

3m

3m, Rf = 0.3 (PE:EA = 10:1). The crude material was purified by flash column chromatography (using petroleum ether/EtOAc = 10:1) to provide the title compound as a white soild in 88% yield (78 mg). **^{1}H NMR** (400 MHz, $CDCl_3$) δ 7.97 – 7.87 (m, 2H), 7.60 – 7.52 (m, 1H), 7.47 (ddd, *J* = 8.1, 6.4, 1.3 Hz, 3H), 7.42 – 7.35 (m, 3H), 7.35 – 7.30 (m, 2H), 7.30 – 7.24 (m, 1H), 7.21 (td, *J* = 7.7, 1.3 Hz, 1H), 7.05 (td, *J* = 7.7, 1.7 Hz, 1H), 6.61 (d, *J* = 15.6 Hz, 1H), 6.19 (dd, *J* = 15.6, 8.5 Hz, 1H), 3.59 (td, *J* = 8.6, 3.7 Hz, 1H), 2.55 (ddd, *J* = 11.3, 8.6, 2.9 Hz, 1H), 2.50 – 2.32 (m, 3H), 2.24 (ddd, *J* = 7.1, 2.9, 1.6 Hz, 1H), 2.07 (ddd, *J* = 11.2, 3.9, 2.6 Hz, 1H). **^{13}C NMR** (101 MHz, $CDCl_3$) δ 201.62, 141.98, 136.95, 133.53, 132.73, 132.62, 130.36, 128.51, 128.36, 128.30, 127.31, 127.14, 126.54, 125.90, 123.18, 60.36, 50.18, 49.85, 48.71, 42.29, 41.74. **HRMS**: calculated for $C_{27}H_{24}BrO$ [M+H]$^+$ 443.1005; found 443.1013.

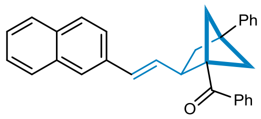

3n

3n, Rf = 0.3 (PE:EA = 10:1). The crude material was purified by flash column chromatography (using petroleum ether/EtOAc = 10:1) to provide the title compound as a white soild in 61% yield (51 mg). **^{1}H NMR** (400 MHz, $CDCl_3$) δ 8.00 – 7.86 (m, 2H), 7.81 – 7.70 (m, 3H), 7.61 – 7.53 (m, 2H), 7.53 – 7.31 (m, 9H), 7.33 – 7.24 (m, 1H), 6.50 – 6.32 (m, 2H), 3.59 (td, *J* = 7.7, 3.9 Hz, 1H), 2.56 (ddd, *J* = 11.3, 8.6, 2.8 Hz, 1H), 2.50 – 2.36 (m, 3H), 2.29 – 2.21 (m, 1H), 2.09 (ddd, *J* = 11.2, 3.9, 2.6 Hz, 1H). **^{13}C NMR** (101 MHz, $CDCl_3$) δ 201.79, 142.12, 137.05, 134.46, 133.48, 132.76, 132.70, 131.50, 130.85, 128.50, 128.37, 128.32, 127.96, 127.81, 127.56, 126.53, 126.10, 125.92, 125.80, 125.61, 123.60, 60.41, 50.22, 49.87, 48.96, 42.41, 42.02. **HRMS**: calculated for $C_{31}H_{27}O$ [M+H]$^+$ 415.2056; found 415.2063.

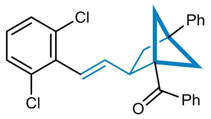

3o

3o, Rf = 0.5 (PE:EA = 7:1). The crude material was purified by flash column chromatography (using petroleum ether/EtOAc = 10:1) to provide the title compound as a white soild in 82% yield (71 mg). **^{1}H NMR** (400 MHz, $CDCl_3$) δ 8.04 – 7.87 (m, 2H), 7.63 – 7.52 (m, 1H), 7.46 (dd, *J* = 8.3, 6.8 Hz, 2H), 7.42 – 7.29 (m, 4H), 7.29 – 7.20 (m, 3H), 7.02 (t, *J* = 8.0 Hz, 1H), 6.37 – 6.13 (m, 2H), 3.69 – 3.55 (m, 1H), 2.56 (ddd, *J* = 11.3, 8.6, 2.8 Hz, 1H), 2.50 – 2.37 (m, 2H), 2.33 (dd, *J* = 9.0, 7.3 Hz, 1H), 2.26 (ddd, *J* = 7.4, 2.9, 1.6 Hz, 1H), 2.09 (ddd, *J* = 11.2, 3.9, 2.7 Hz, 1H). **^{13}C NMR** (101 MHz, $CDCl_3$) δ 201.36, 142.01, 139.01, 136.95, 134.49, 134.19, 132.67, 128.43, 128.41, 128.35, 128.15, 127.81, 126.53, 125.93, 124.91, 60.25, 50.51, 49.98, 49.25, 42.01, 41.50. **HRMS**: calculated for $C_{27}H_{23}Cl_2O$ [M+H]$^+$ 433.1120; found 433.1130.

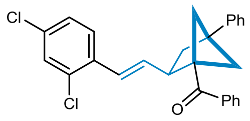

3p

3p, Rf = 0.3 (PE:EA = 10:1). The crude material was purified by flash column chromatography (using petroleum ether/EtOAc = 10:1) to provide the title compound as a white soild in 72% yield (62 mg). **^{1}H NMR** (400 MHz, $CDCl_3$) δ 8.05 – 7.81 (m, 2H), 7.55 (d, *J* = 7.3 Hz, 1H), 7.47 (d, *J* = 7.6 Hz, 2H), 7.36 (d, *J* = 7.4 Hz, 2H), 7.28 (ddd, *J* = 14.9, 8.0, 3.2 Hz, 5H), 7.13 (dd, *J* = 8.5, 2.2 Hz, 1H), 6.56 (d, *J* = 15.7 Hz, 1H), 6.21 (dd, *J* = 15.7, 8.6 Hz, 1H), 3.55 (td, *J* = 8.7, 3.7 Hz, 1H), 2.54 (ddd, *J* = 11.4, 8.6, 2.8 Hz, 1H), 2.42 (td, *J* = 8.8, 4.3 Hz, 2H), 2.34 (t, *J* = 8.0 Hz, 1H), 2.23 (dt, *J* = 7.4, 2.1 Hz, 1H), 2.04 (dt, *J* = 11.2, 3.2 Hz, 1H). **^{13}C NMR** (101 MHz, $CDCl_3$) δ 201.59, 141.87, 136.94, 134.02, 133.80, 133.16, 133.10, 132.80, 129.14, 128.55, 128.39, 128.27, 127.64, 127.03, 126.76, 126.59, 125.90, 60.37, 50.11, 49.89, 48.82, 42.36, 41.77. **HRMS**: calculated for $C_{27}H_{23}Cl_2O$ [M+H] $^+$ 433.1120; found 433.1128.

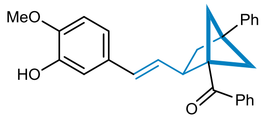

3q

3q, Rf = 0.2 (PE:EA = 6:1). The crude material was purified by flash column chromatography (using petroleum ether/EtOAc = 6:1) to provide the title compound as a white soild in 66% yield (54 mg). **^{1}H NMR** (400 MHz, $CDCl_3$) δ 7.88 (dd, *J* = 8.3, 1.3 Hz, 2H), 7.58 – 7.51 (m, 1H), 7.45 (dd, *J* = 8.3, 6.8 Hz, 2H), 7.39 – 7.33 (m, 2H), 7.33 – 7.28 (m, 2H), 7.28 – 7.21 (m, 1H), 6.86 (d, *J* = 1.9 Hz, 1H), 6.78 – 6.63 (m, 2H), 6.24 – 6.04 (m, 2H), 5.56 (s, 1H), 3.85 (s, 3H), 3.58 – 3.38 (m, 1H), 2.50 (ddd, *J* = 11.3, 8.6, 2.8 Hz, 1H), 2.44 – 2.31 (m, 3H), 2.24 – 2.18 (m, 1H), 2.06 – 1.96 (m, 1H). **^{13}C NMR** (101 MHz, $CDCl_3$) δ 201.85, 145.99, 145.47, 142.21, 137.08, 132.63, 130.83, 128.75, 128.46, 128.35, 128.30, 126.48, 125.91, 118.49, 111.82, 110.41, 60.35, 55.92, 50.27, 49.84, 48.78, 42.29, 42.00. **HRMS**: calculated for $C_{28}H_{27}O_3$ [M+H] $^+$ 411.1955; found 411.1961.

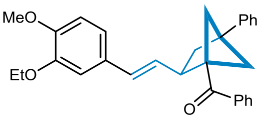

3r

3r, Rf = 0.3 (PE:EA = 6:1). The crude material was purified by flash column chromatography (using petroleum ether/EtOAc = 6:1) to provide the title compound as a white soild in 86% yield (75 mg). **^{1}H NMR** (400 MHz, $CDCl_3$) δ 8.00 – 7.79 (m, 2H), 7.58 – 7.51 (m, 1H), 7.45 (dd, *J* = 8.3, 6.8 Hz, 2H), 7.39 – 7.33 (m, 2H), 7.33 – 7.22 (m, 3H), 6.79 (d, *J* = 1.2 Hz, 1H), 6.75 (d, *J* = 1.2 Hz, 2H), 6.20 (d, *J* = 15.7 Hz, 1H), 6.10 (dd, *J* = 15.7, 8.1 Hz, 1H), 4.07 (q, *J* = 7.0 Hz, 2H), 3.84 (s, 3H), 3.51 (td, *J* = 8.5, 3.9 Hz, 1H), 2.51 (ddd, *J* = 11.3, 8.6, 2.8 Hz, 1H), 2.45 – 2.31 (m, 3H), 2.22 (ddd, *J* = 6.9, 2.9, 1.6 Hz, 1H), 2.09 – 1.97 (m, 1H), 1.46 (t, *J* = 7.0 Hz, 3H). **^{13}C NMR** (101 MHz, $CDCl_3$) δ 201.82, 148.77, 148.15, 142.14, 137.03, 132.62, 131.07, 130.07, 128.43, 128.32, 128.30, 128.26, 126.47, 125.87, 119.20, 111.18, 110.12, 64.20, 60.31, 55.88, 50.18, 49.78, 48.85, 42.35, 42.01, 14.76. **HRMS**: calculated for $C_{30}H_{31}O_3$ [M+H] $^+$ 439.2268; found 439.2275.

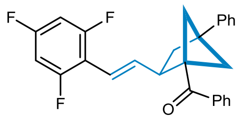

3s

3s, Rf = 0.4 (PE:EA = 10:1). The crude material was purified by flash column chromatography (using petroleum ether/EtOAc = 10:1) to provide the title compound as a colorless oil in 74% yield (62 mg). **^{1}H NMR** (400 MHz, $CDCl_3$) δ 7.99 – 7.75 (m, 2H), 7.59 – 7.52 (m, 1H), 7.46 (dd, *J* = 8.3, 6.8 Hz, 2H), 7.40 – 7.33 (m, 2H), 7.33 – 7.23 (m, 3H), 6.75 – 6.41 (m, 3H), 6.17 (d, *J* = 16.2 Hz, 1H), 3.50 (td, *J* = 8.8, 3.6 Hz, 1H), 2.53 (ddd, *J* = 11.3, 8.6, 2.8 Hz, 1H), 2.48 – 2.30 (m, 3H), 2.23 (ddd, *J* = 7.2, 2.9, 1.6 Hz, 1H), 2.10 – 1.97 (m, 1H). **^{13}C NMR** (101 MHz, $CDCl_3$) δ 201.61, 162.95 – 161.51 (m), 160.02 – 158.65 (m), 141.94, 137.29 (td, $J_{C\text{-}F}$ = 7.2, 1.9 Hz), 137.01, 132.69, 128.47, 128.36, 128.26, 126.55, 125.90, 116.91, 111.92 – 109.35 (m), 101.42 – 98.22 (m), 60.34, 50.16, 50.03, 49.94, 42.35, 41.91. **^{19}F NMR** (377 MHz, $CDCl_3$) δ (-109.99) – (-110.03) (m, 1F), (-110.23) – (-110.33) (m, 2F). **HRMS**: calculated for $C_{27}H_{22}F_3O$ $[M+H]^+$ 419.1617; found 419.1624.

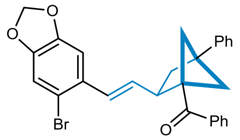

3t

3t, Rf = 0.3 (PE:EA = 10:1). The crude material was purified by flash column chromatography (using petroleum ether/EtOAc = 10:1) to provide the title compound as a white soild in 88% yield (84 mg). **^{1}H NMR** (400 MHz, $CDCl_3$) δ 8.04 – 7.85 (m, 2H), 7.59 – 7.52 (m, 1H), 7.46 (dd, *J* = 8.3, 6.8 Hz, 2H), 7.39 – 7.34 (m, 2H), 7.33 – 7.22 (m, 3H), 6.92 (s, 1H), 6.85 (s, 1H), 6.60 – 6.46 (m, 1H), 6.03 (dd, *J* = 15.6, 8.5 Hz, 1H), 5.93 (s, 2H), 3.54 (td, *J* = 8.7, 3.7 Hz, 1H), 2.53 (ddd, *J* = 11.3, 8.6, 2.8 Hz, 1H), 2.47 – 2.29 (m, 3H), 2.22 (ddd, *J* = 7.4, 2.9, 1.6 Hz, 1H), 2.03 (ddd, *J* = 11.2, 3.9, 2.7 Hz, 1H). **^{13}C NMR** (101 MHz, $CDCl_3$) δ 201.67, 147.60, 147.50, 142.02, 136.99, 132.73, 131.85, 130.32, 130.13, 128.52, 128.36, 128.30, 126.53, 125.90, 114.08, 112.35, 106.37, 101.63, 60.38, 50.14, 49.84, 48.68, 42.31, 41.82. **HRMS**: calculated for $C_{28}H_{24}BrO_3$ $[M+H]^+$ 487.0903; found 487.0913.

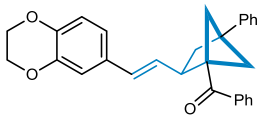

3u

3u, Rf = 0.3 (PE:EA = 5:1). The crude material was purified by flash column chromatography (using petroleum ether/EtOAc = 7:1) to provide the title compound as a white soild in 74% yield (62 mg). **^{1}H NMR** (400 MHz, $CDCl_3$) δ 7.96 – 7.84 (m, 2H), 7.62 – 7.50 (m, 1H), 7.45 (dd, *J* = 8.3, 6.9 Hz, 2H), 7.40 – 7.33 (m, 2H), 7.33 – 7.19 (m, 3H), 6.86 – 6.58 (m, 3H), 6.29 – 5.96 (m, 2H), 4.22 (s, 4H), 3.57 – 3.41 (m, 1H), 2.50 (ddd, *J* = 11.3, 8.6, 2.8 Hz, 1H), 2.45 – 2.27 (m, 3H), 2.21 (ddd, *J* = 7.2, 2.9, 1.6 Hz, 1H), 2.06 – 1.90 (m, 1H). **^{13}C NMR** (101 MHz, $CDCl_3$) δ 201.75, 143.31, 142.92, 142.18, 137.05, 132.62, 130.86, 130.65, 128.79, 128.45, 128.33, 128.27, 126.48, 125.90, 119.50, 117.06, 114.71, 64.35, 64.26, 60.34, 50.26, 49.81, 48.75, 42.24, 41.98. **HRMS**: calculated for $C_{29}H_{27}O_3$ $[M+H]^+$ 423.1955; found 423.1961.

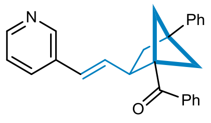

3v

3v, Rf = 0.2 (PE:EA = 2:1). The crude material was purified by flash column chromatography (using petroleum ether/EtOAc = 3:1) to provide the title compound as a white soild in 83% yield (61 mg). **^{1}H NMR** (400 MHz, $CDCl_3$) δ 8.41 (s, 2H), 7.96 – 7.80 (m, 2H), 7.62 – 7.51 (m, 2H), 7.45 (dd, *J* = 8.3, 6.9 Hz, 2H), 7.40 – 7.33 (m, 2H), 7.33 – 7.23 (m, 3H), 7.17 (dd, *J* = 8.0, 4.7 Hz, 1H), 6.32 (dd, *J* = 15.8, 8.2 Hz, 1H), 6.22 (d, *J* = 15.9 Hz, 1H), 3.53 (tdd, *J* = 8.4, 3.8, 1.4 Hz, 1H), 2.53 (ddd, *J* = 11.3, 8.6, 2.8 Hz, 1H), 2.47 – 2.32 (m, 3H), 2.23 (ddd, *J* = 7.2, 2.9, 1.6 Hz, 1H), 2.03 (ddd, *J* = 11.2, 3.8, 2.6 Hz, 1H). **^{13}C NMR** (101 MHz, $CDCl_3$) δ 201.52, 148.14, 147.97, 141.84, 136.91, 133.01, 132.83, 132.66, 132.60, 128.55, 128.38, 128.24, 127.81, 126.59, 125.87, 123.32, 60.30, 50.10, 49.90, 48.85, 42.39, 41.82. **HRMS**: calculated for $C_{26}H_{24}NO$, $[M+H]^+$ 366.1852; found 366.1854.

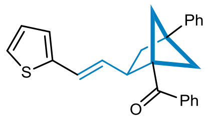

3w

3w, Rf = 0.4 (PE:EA = 9:1). The crude material was purified by flash column chromatography (using petroleum ether/EtOAc = 10:1) to provide the title compound as a colorless oil in 92% yield (68 mg). **^{1}H NMR** (400 MHz, $CDCl_3$) δ 7.99 – 7.69 (m, 2H), 7.67 – 7.50 (m, 1H), 7.45 (dd, *J* = 8.4, 6.9 Hz, 2H), 7.41 – 7.33 (m, 2H), 7.33 – 7.23 (m, 3H), 7.08 (d, *J* = 5.0 Hz, 1H), 6.89 (dd, *J* = 5.1, 3.5 Hz, 1H), 6.78 (dd, *J* = 3.6, 1.0 Hz, 1H), 6.39 (d, *J* = 15.6 Hz, 1H), 6.11 (dd, *J* = 15.6, 8.5 Hz, 1H), 3.48 (td, *J* = 8.6, 3.7 Hz, 1H), 2.50 (ddd, *J* = 11.3, 8.6, 2.8 Hz, 1H), 2.45 – 2.31 (m, 3H), 2.22 (ddd, *J* = 7.1, 2.9, 1.6 Hz, 1H), 2.01 (ddd, *J* = 11.1, 3.9, 2.7 Hz, 1H). **^{13}C NMR** (101 MHz, $CDCl_3$) δ 201.65, 142.08, 142.04, 136.97, 132.69, 130.12, 128.48, 128.35, 128.31, 127.11, 126.52, 125.89, 125.04, 124.51, 123.71, 60.26, 50.18, 49.84, 48.70, 42.36, 41.94. **HRMS**: calculated for $C_{25}H_{23}OS$ $[M+H]^+$ 371.1464; found 371.1470.

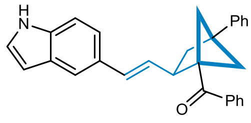

3x

3x, Rf = 0.2 (PE:EA = 4:1). The crude material was purified by flash column chromatography (using petroleum ether/EtOAc = 4:1) to provide the title compound as a colorless oil in 96% yield (77 mg). **^{1}H NMR** (400 MHz, $CDCl_3$) δ 8.29 (s, 1H), 7.97 – 7.85 (m, 2H), 7.59 – 7.53 (m, 1H), 7.50 – 7.43 (m, 3H), 7.41 – 7.35 (m, 2H), 7.35 – 7.31 (m, 2H), 7.30 – 7.27 (m, 1H), 7.23 (d, *J* = 8.5 Hz, 1H), 7.17 – 7.12 (m, 2H), 6.48 (t, *J* = 2.8 Hz, 1H), 6.38 (d, *J* = 15.6 Hz, 1H), 6.21 (dd, *J* = 15.7, 8.4 Hz, 1H), 3.57 (td, *J* = 8.6, 3.7 Hz, 1H), 2.54 (ddd, *J* = 11.3, 8.6, 2.8 Hz, 1H), 2.48 – 2.32 (m, 3H), 2.27 – 2.20 (m, 1H), 2.06 (ddd, *J* = 11.0, 4.2, 2.7 Hz, 1H). **^{13}C NMR** (101 MHz, $CDCl_3$) δ 202.24, 142.31, 137.09, 135.30, 132.63, 132.49, 129.10, 128.46, 128.33, 127.90, 127.37, 126.45, 125.93, 124.56, 120.30, 118.85, 110.99, 102.63, 60.48, 50.31, 49.81, 49.03, 42.29, 42.10. **HRMS**: calculated for $C_{29}H_{26}NO$ $[M+H]^+$ 404.2009; found 404.2013.

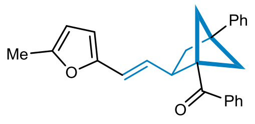

3y

3y, Rf = 0.3 (PE:EA = 9:1). The crude material was purified by flash column chromatography (using petroleum ether/EtOAc = 10:1) to provide the title compound as a colorless oil in 71% yield (52 mg)**.** ^{1}**H NMR** (400 MHz, $CDCl_3$) δ 7.98 – 7.86 (m, 2H), 7.58 – 7.51 (m, 1H), 7.46 (d, *J* = 7.7 Hz, 2H), 7.35 (d, *J* = 7.4 Hz, 2H), 7.32 – 7.22 (m, 3H), 6.15 (dd, *J* = 15.6, 8.6 Hz, 1H), 6.05 – 5.67 (m, 3H), 3.47 (td, *J* = 8.9, 3.7 Hz, 1H), 2.49 (ddd, *J* = 11.3, 8.6, 2.8 Hz, 1H), 2.45 – 2.33 (m, 3H), 2.27 (s, 3H), 2.19 (dt, *J* = 7.2, 1.9 Hz, 1H), 2.01 (dt, *J* = 11.1, 3.3 Hz, 1H). ^{13}**C NMR** (101 MHz, $CDCl_3$) δ 201.64, 151.57, 150.88, 142.20, 136.90, 132.64, 128.44, 128.34, 127.30, 126.47, 125.88, 119.88, 108.28, 107.08, 60.41, 50.32, 49.79, 48.73, 42.20, 13.62. **HRMS**: calculated for $C_{26}H_{25}O_2$ $[M+H]^+$ 369.1849; found 369.1856.

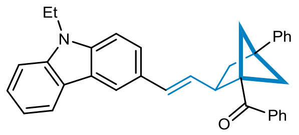

3z

3z, Rf = 0.2 (PE:EA = 10:1). The crude material was purified by flash column chromatography (using petroleum ether/EtOAc = 10:1) to provide the title compound as a white soild in 95% yield (91 mg)**.** ^{1}**H NMR** (400 MHz, $CDCl_3$) δ 8.09 (d, *J* = 7.7 Hz, 1H), 7.96 (dd, *J* = 4.4, 2.7 Hz, 3H), 7.60 – 7.53 (m, 1H), 7.52 – 7.44 (m, 3H), 7.44 – 7.33 (m, 6H), 7.32 – 7.28 (m, 1H), 7.28 – 7.22 (m, 2H), 6.48 (d, *J* = 15.6 Hz, 1H), 6.31 (dd, *J* = 15.7, 8.4 Hz, 1H), 4.33 (q, *J* = 7.2 Hz, 2H), 3.61 (td, *J* = 8.6, 3.6 Hz, 1H), 2.57 (ddd, *J* = 11.3, 8.6, 2.8 Hz, 1H), 2.52 – 2.37 (m, 3H), 2.32 – 2.24 (m, 1H), 2.11 (dt, *J* = 11.2, 3.3 Hz, 1H), 1.41 (t, *J* = 7.2 Hz, 3H). ^{13}**C NMR** (101 MHz, $CDCl_3$) δ 202.00, 142.30, 140.20, 139.37, 137.14, 132.61, 132.06, 128.45, 128.36, 128.34, 128.22, 127.57, 126.46, 125.93, 125.60, 124.15, 122.96, 122.88, 120.40, 118.78, 118.21, 108.47, 108.26, 60.46, 50.24, 49.82, 49.07, 42.40, 42.14, 37.49, 13.74. **HRMS**: calculated for $C_{35}H_{32}NO$ $[M+H]^+$ 482.2478; found 482.2485.

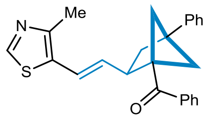

3aa

3aa, Rf = 0.5 (PE:EA = 5:1). The crude material was purified by flash column chromatography (using petroleum ether/EtOAc = 10:1) to provide the title compound as a white solid in 78% yield (60 mg)**.** ^{1}**H NMR** (400 MHz, $CDCl_3$) δ 8.46 (s, 1H), 8.01 – 7.80 (m, 2H), 7.59 – 7.52 (m, 1H), 7.45 (dd, *J* = 8.3, 6.9 Hz, 2H), 7.35 (d, *J* = 7.4 Hz, 2H), 7.32 – 7.22 (m, 3H), 6.29 (d, *J* = 15.5 Hz, 1H), 5.99 (dd, *J* = 15.5, 8.7 Hz, 1H), 3.48 (td, *J* = 8.7, 3.7 Hz, 1H), 2.51 (ddd, *J* = 11.3, 8.6, 2.8 Hz, 1H), 2.44 – 2.35 (m, 2H), 2.31 (t, *J* = 8.0 Hz, 1H), 2.28 (s, 3H), 2.23 (ddd, *J* = 7.3, 2.9, 1.6 Hz, 1H), 1.99 (ddd, *J* = 11.2, 3.9, 2.6 Hz, 1H). ^{13}**C NMR** (101 MHz, $CDCl_3$) δ 201.58, 149.13, 141.85, 137.03, 133.39, 132.78, 128.54, 128.38, 128.26, 126.60, 125.88, 121.12, 60.35, 50.11, 49.95, 48.99, 42.35, 42.00, 15.02. **HRMS**: calculated for $C_{25}H_{24}NOS$ $[M+H]^+$ 386.1573; found 386.1576.

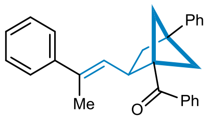

4a

4a, Rf = 0.4 (PE:EA = 9:1). The crude material was purified by flash column chromatography (using petroleum ether/EtOAc = 15:1) to provide the title compound as a colorless oil in 72% yield (54 mg). **^{1}H NMR** (400 MHz, $CDCl_3$) δ 7.95 – 7.91 (m, 2H), 7.58 – 7.52 (m, 1H), 7.46 (dd, *J* = 8.4, 6.9 Hz, 2H), 7.37 (t, *J* = 7.5 Hz, 2H), 7.33 – 7.17 (m, 8H), 5.87 (dd, *J* = 9.3, 1.6 Hz, 1H), 3.72 (td, *J* = 9.0, 3.9 Hz, 1H), 2.60 (ddd, *J* = 11.2, 8.6, 2.8 Hz, 1H), 2.47 (dd, *J* = 9.3, 6.5 Hz, 1H), 2.44 – 2.37 (m, 2H), 2.23 (ddd, *J* = 7.1, 2.9, 1.6 Hz, 1H), 1.92 (dt, *J* = 11.0, 3.2 Hz, 1H), 1.76 (d, *J* = 1.4 Hz, 3H). **^{13}C NMR** (101 MHz, $CDCl_3$) δ 201.97, 143.55, 142.22, 137.05, 136.99, 132.63, 128.92, 128.44, 128.34, 128.28, 128.05, 126.79, 126.46, 125.90, 125.77, 60.34, 50.11, 49.92, 44.47, 43.32, 42.52, 16.21. **HRMS**: calculated for $C_{28}H_{27}O$ $[M+H]^+$ 379.2056; found 379.2063.

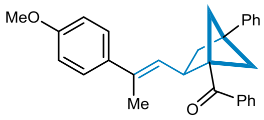

4b

4b, Rf = 0.2 (PE:EA = 10:1). The crude material was purified by flash column chromatography (using petroleum ether/EtOAc = 10:1) to provide the title compound as a white solid in 50% yield (41 mg). **^{1}H NMR** (400 MHz, $CDCl_3$) δ 7.96 – 7.86 (m, 2H), 7.57 – 7.51 (m, 1H), 7.48 – 7.41 (m, 2H), 7.40 – 7.33 (m, 2H), 7.32 – 7.28 (m, 2H), 7.28 – 7.21 (m, 1H), 7.20 – 7.13 (m, 2H), 6.85 – 6.77 (m, 2H), 5.79 (dq, *J* = 9.3, 1.4 Hz, 1H), 3.79 (s, 3H), 3.70 (td, *J* = 8.9, 3.8 Hz, 1H), 2.58 (ddd, *J* = 11.2, 8.6, 2.8 Hz, 1H), 2.45 (dd, *J* = 9.3, 6.5 Hz, 1H), 2.41 – 2.34 (m, 2H), 2.22 (ddd, *J* = 7.0, 2.9, 1.6 Hz, 1H), 1.90 (ddd, *J* = 10.9, 3.8, 2.7 Hz, 1H), 1.73 (d, *J* = 1.4 Hz, 3H). **^{13}C NMR** (101 MHz, $CDCl_3$) δ 202.06, 158.62, 142.29, 137.10, 136.30, 136.10, 132.59, 128.42, 128.33, 128.28, 127.43, 126.78, 126.44, 125.90, 113.41, 60.37, 55.25, 50.13, 49.90, 44.50, 43.43, 42.54, 16.24. **HRMS**: calculated for $C_{29}H_{29}O_2$ $[M+H]^+$ 409.2162; found 409.2170.

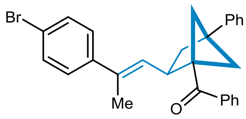

4c

4c, Rf = 0.3 (PE:EA = 10:1). The crude material was purified by flash column chromatography (using petroleum ether/EtOAc = 10:1) to provide the title compound as a white solid in 52% yield (47 mg). **^{1}H NMR** (400 MHz, $CDCl_3$) δ 7.95 – 7.85 (m, 2H), 7.58 – 7.52 (m, 1H), 7.48 – 7.41 (m, 2H), 7.40 – 7.32 (m, 4H), 7.32 – 7.22 (m, 3H), 7.13 – 6.99 (m, 2H), 5.84 (dq, *J* = 9.3, 1.4 Hz, 1H), 3.68 (td, *J* = 9.0, 3.9, 1.6 Hz, 1H), 2.58 (ddd, *J* = 11.2, 8.6, 2.8 Hz, 1H), 2.45 (dd, *J* = 9.2, 6.5 Hz, 1H), 2.41 – 2.34 (m, 2H), 2.22 (ddd, *J* = 7.2, 2.9, 1.6 Hz, 1H), 1.90 (ddd, *J* = 11.0, 3.8, 2.7 Hz, 1H), 1.72 (d, *J* = 1.4 Hz, 3H). **^{13}C NMR** (101 MHz, $CDCl_3$) δ 201.90, 142.39, 142.08, 137.02, 135.97, 132.71, 131.09, 129.55, 128.48, 128.36, 128.27, 127.42, 126.52, 125.89, 120.64, 60.29, 50.03, 49.95, 44.49, 43.24, 42.62, 16.10. **HRMS**: calculated for $C_{28}H_{26}BrO$ $[M+H]^+$ 457.1162; found 457.1170.

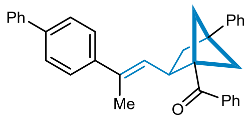

4d

4d, Rf = 0.4 (PE:EA = 10:1). The crude material was purified by flash column chromatography (using petroleum ether/EtOAc = 14:1) to provide the title compound as a white solid in 61% yield (55 mg). **^{1}H NMR** (400 MHz, $CDCl_3$) δ 8.00 – 7.85 (m, 2H), 7.64 – 7.40 (m, 9H), 7.39 – 7.33 (m, 3H), 7.33 – 7.28 (m, 4H), 7.29 – 7.21 (m, 1H), 5.93 (dq, *J* = 9.3, 1.4 Hz, 1H), 3.74 (dt, *J* = 8.8, 3.6 Hz, 1H), 2.60 (ddd, *J* = 11.2, 8.6, 2.8 Hz, 1H), 2.50 – 2.38 (m, 3H), 2.24 (ddd, *J* = 7.0, 2.9, 1.5 Hz, 1H), 1.93 (ddd, *J* = 11.0, 3.8, 2.7 Hz, 1H), 1.79 (d, *J* = 1.3 Hz, 3H). **^{13}C NMR** (101 MHz, $CDCl_3$) δ 201.99, 142.45, 142.23, 140.81, 139.66, 137.10, 136.51, 132.66, 129.03, 128.72, 128.48, 128.37, 128.31, 127.14, 126.94, 126.79, 126.50, 126.15, 125.92, 60.39, 50.14, 49.96, 44.55, 43.39, 42.59, 16.16. **HRMS**: calculated for $C_{34}H_{31}O$ $[M+H]^{+}$ 455.2369; found 455.2373.

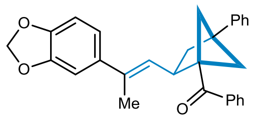

4e

4e, Rf = 0.2 (PE:EA = 10:1). The crude material was purified by flash column chromatography (using petroleum ether/EtOAc = 10:1) to provide the title compound as a white solid in 67% yield (57 mg). **^{1}H NMR** (400 MHz, $CDCl_3$) δ 8.00 – 7.82 (m, 2H), 7.58 – 7.51 (m, 1H), 7.48 – 7.42 (m, 2H), 7.39 – 7.33 (m, 2H), 7.32 – 7.22 (m, 3H), 6.71 (dt, *J* = 11.2, 1.2 Hz, 3H), 5.92 (s, 2H), 5.76 (dd, *J* = 9.3, 1.4 Hz, 1H), 3.68 (td, *J* = 8.9, 3.8 Hz, 1H), 2.57 (ddd, *J* = 11.2, 8.6, 2.8 Hz, 1H), 2.44 (dd, *J* = 9.3, 6.4 Hz, 1H), 2.37 (ddd, *J* = 8.0, 5.7, 3.6 Hz, 2H), 2.21 (ddd, *J* = 7.1, 2.9, 1.6 Hz, 1H), 1.89 (ddd, *J* = 10.9, 3.8, 2.7 Hz, 1H), 1.70 (d, *J* = 1.4 Hz, 3H). **^{13}C NMR** (101 MHz, $CDCl_3$) δ 201.99, 147.40, 146.45, 142.23, 138.02, 137.08, 136.49, 132.63, 128.44, 128.34, 128.27, 128.02, 126.46, 125.89, 119.12, 107.77, 106.43, 100.87, 60.33, 50.08, 49.92, 44.47, 43.36, 42.56, 16.46. **HRMS**: calculated for $C_{29}H_{27}O_3$ $[M+H]^{+}$ 423.1955; found 423.1954.

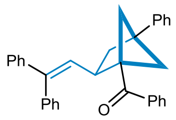

4f

4f, Rf = 0.4 (PE:EA = 15:1). The crude material was purified by flash column chromatography (using petroleum ether/EtOAc = 20:1) to provide the title compound as a white solid in 78% yield (69 mg). **^{1}H NMR** (400 MHz, $CDCl_3$) δ 7.79 – 7.73 (m, 2H), 7.56 – 7.50 (m, 1H), 7.39 – 7.32 (m, 4H), 7.32 – 7.14 (m, 9H), 7.14 – 7.09 (m, 2H), 6.82 – 6.72 (m, 2H), 6.20 (d, *J* = 10.4 Hz, 1H), 3.48 (tdd, *J* = 10.2, 3.9, 1.4 Hz, 1H), 2.52 – 2.39 (m, 2H), 2.33 (dd, *J* = 6.6, 2.6 Hz, 1H), 2.30 – 2.22 (m, 2H), 2.01 (ddd, *J* = 11.0, 4.0, 2.6 Hz, 1H). **^{13}C NMR** (101 MHz, $CDCl_3$) δ 201.65, 143.26, 142.09, 142.00, 139.66, 136.69, 132.56, 129.53, 129.41, 128.39, 128.32, 128.18, 127.99, 127.95, 127.23, 127.13, 126.73, 126.45, 125.87, 60.52, 49.84, 49.73, 45.32, 43.37, 42.41. **HRMS**: calculated for $C_{33}H_{29}O$ $[M+H]^{+}$ 441.2213; found 441.2205.

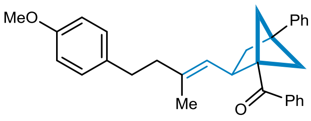

4g

4g, Rf = 0.2 (PE:EA = 9:1). The crude material was purified by flash column chromatography (using petroleum ether/EtOAc = 9:1) to provide the title compound as a white solid in 40% yield (35 mg). ^{1}**H NMR** (400 MHz, $CDCl_3$) δ 7.99 – 7.81 (m, 2H), 7.58 – 7.51 (m, 1H), 7.44 (dd, *J* = 8.2, 6.8 Hz, 2H), 7.37 – 7.30 (m, 2H), 7.30 – 7.20 (m, 3H), 7.05 – 6.98 (m, 2H), 6.82 – 6.75 (m, 2H), 5.27 (dd, *J* = 9.2, 1.5 Hz, 1H), 3.77 (s, 3H), 3.62 – 3.41 (m, 1H), 2.47 (tdd, *J* = 8.4, 5.6, 2.9 Hz, 3H), 2.37 (dd, *J* = 9.1, 6.5 Hz, 1H), 2.30 (dd, *J* = 6.4, 2.7 Hz, 1H), 2.25 (dd, *J* = 9.1, 7.1 Hz, 1H), 2.19 – 2.06 (m, 3H), 1.72 (dt, *J* = 10.9, 3.2 Hz, 1H), 1.59 (H_2O), 1.34 (d, *J* = 1.3 Hz, 3H). ^{13}**C NMR** (101 MHz, $CDCl_3$) δ 202.17, 157.63, 142.48, 137.15, 137.13, 134.27, 132.55, 129.16, 128.35, 128.31, 126.38, 125.90, 125.81, 113.62, 60.19, 55.22, 50.15, 49.81, 43.77, 43.36, 42.40, 41.81, 33.73, 16.46. **HRMS**: calculated for $C_{31}H_{33}O_2$ $[M+H]^+$ 437.2475; found 437.2481.

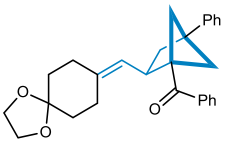

4h

4h, Rf = 0.5 (PE:EA = 5:1). The crude material was purified by flash column chromatography (using petroleum ether/EtOAc = 10:1) to provide the title compound as a white solid in 77% yield (64 mg). ^{1}**H NMR** (400 MHz, $CDCl_3$) δ 7.96 – 7.87 (m, 2H), 7.57 – 7.51 (m, 1H), 7.49 – 7.41 (m, 2H), 7.37 – 7.32 (m, 2H), 7.31 – 7.16 (m, 3H), 5.28 (d, *J* = 9.6 Hz, 1H), 4.04 – 3.75 (m, 4H), 3.58 (td, *J* = 9.3, 3.6 Hz, 1H), 2.48 (ddd, *J* = 11.1, 8.5, 2.8 Hz, 1H), 2.41 – 2.24 (m, 3H), 2.22 – 2.03 (m, 3H), 1.96 (tt, *J* = 14.4, 6.4 Hz, 2H), 1.80 (dt, *J* = 11.0, 3.2 Hz, 1H), 1.58 (dddd, *J* = 12.0, 7.0, 4.9, 1.9 Hz, 1H), 1.45 – 1.33 (m, 2H), 0.99 (ddd, *J* = 13.6, 9.1, 4.8 Hz, 1H). ^{13}**C NMR** (101 MHz, $CDCl_3$) δ 201.68, 142.35, 139.11, 136.70, 132.74, 128.39, 128.29, 126.38, 125.89, 123.60, 108.65, 64.15, 64.12, 60.40, 50.28, 49.85, 43.21, 42.19, 35.85, 34.99, 33.51, 25.44. **HRMS**: calculated for $C_{28}H_{31}O_3$ $[M+H]^+$ 415.2268; found 415.2258.

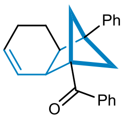

5a

5a, Rf = 0.5 (PE:EA = 15:1). The crude material was purified by flash column chromatography (using petroleum ether/EtOAc = 20:1) to provide the title compound as a yellow oil in 87% yield (54 mg). ^{1}**H NMR** (400 MHz, $CDCl_3$) δ 8.04 – 7.86 (m, 2H), 7.61 – 7.51 (m, 1H), 7.51 – 7.41 (m, 2H), 7.40 – 7.28 (m, 2H), 7.28 – 7.15 (m, 3H), 6.03 (ddt, *J* = 9.2, 5.8, 2.8 Hz, 1H), 5.54 (dt, *J* = 10.1, 2.5 Hz, 1H), 3.13 (dq, *J* = 8.7, 2.6 Hz, 1H), 2.73 (tdd, *J* = 9.2, 7.2, 1.6 Hz, 1H), 2.51 (dd, *J* = 9.3, 6.3 Hz, 1H), 2.31 (dd, *J* = 9.3, 7.3 Hz, 1H), 2.23 (d, *J* = 6.4 Hz, 1H), 2.11 – 2.00 (m, 2H), 1.86 (dddt, *J* = 19.1, 10.6, 5.6, 2.9 Hz, 1H), 1.67 – 1.55 (m, 1H), 1.28 – 1.10 (m, 1H). ^{13}**C NMR** (101 MHz, $CDCl_3$) δ 201.86, 141.84, 136.94, 132.78, 131.39, 128.53, 128.48, 128.20, 127.32, 126.28, 126.03, 58.65, 52.75, 51.30, 44.07, 43.88, 37.76, 23.45, 22.80. **HRMS**: calculated for $C_{23}H_{23}O$ $[M+H]^+$ 315.1743; found 315.1737.

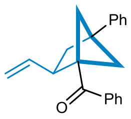

5b

5b, Rf = 0.6 (PE:EA = 8:1). The crude material was purified by flash column chromatography (using petroleum ether/EtOAc = 15:1) to provide the title compound as a colorless oil in 84% yield (48 mg). **^{1}H NMR** (400 MHz, $CDCl_3$) δ 8.01 – 7.76 (m, 2H), 7.60 – 7.53 (m, 1H), 7.51 – 7.43 (m, 2H), 7.39 – 7.32 (m, 2H), 7.32 – 7.21 (m, 3H), 5.89 (ddd, *J* = 17.0, 10.3, 8.2 Hz, 1H), 5.08 – 4.84 (m, 2H), 3.47 – 3.30 (m, 1H), 2.49 – 2.32 (m, 3H), 2.26 (dd, *J* = 9.0, 7.2 Hz, 1H), 2.16 (ddd, *J* = 7.3, 2.9, 1.6 Hz, 1H), 1.96 (ddd, *J* = 11.1, 3.9, 2.7 Hz, 1H). **^{13}C NMR** (101 MHz, $CDCl_3$) δ 201.64, 142.17, 138.51, 136.89, 132.70, 128.45, 128.32, 126.47, 125.88, 116.32, 60.03, 50.32, 49.74, 49.36, 42.03, 41.18. **HRMS**: calculated for $C_{21}H_{21}O$ [M+H] $^+$ 289.1587; found 289.1592.

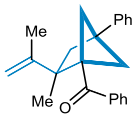

5c

5c, Rf = 0.5 (PE:EA = 10:1). The crude material was purified by flash column chromatography (using petroleum ether/EtOAc = 15:1) to provide the title compound as a colorless oil in 63% yield (40 mg). **^{1}H NMR** (400 MHz, $CDCl_3$) δ 7.85 – 7.81 (m, 2H), 7.55 – 7.48 (m, 1H), 7.46 – 7.38 (m, 2H), 7.36 – 7.28 (m, 2H), 7.27 – 7.16 (m, 3H), 5.01 (dd, *J* = 10.6, 5.0 Hz, 2H), 2.69 (dt, *J* = 9.6, 6.1 Hz, 1H), 2.64 – 2.57 (m, 1H), 2.56 – 2.50 (m, 1H), 2.20 (ddt, *J* = 17.3, 6.3, 3.1 Hz, 2H), 1.95 – 1.87 (m, 1H), 1.65 (d, *J* = 5.2 Hz, 3H), 1.48 (d, *J* = 5.5 Hz, 3H). **^{13}C NMR** (101 MHz, $CDCl_3$) δ 203.51, 149.51, 142.49, 138.50, 132.25, 128.75, 128.27, 128.18, 126.37, 125.77, 112.47, 60.81, 52.98, 48.45, 48.29, 47.16, 45.66, 24.57, 22.42. **HRMS**: calculated for $C_{23}H_{25}O$ [M+H] $^+$ 317.1900; found 317.1902.

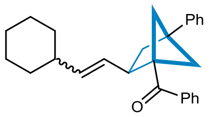

5d

5d, Rf = 0.6 (PE:EA = 10:1). The crude material was purified by flash column chromatography (using petroleum ether/EtOAc = 10:1) to provide the title compound as a white solid in 52% yield (38 mg). **^{1}H NMR** (400 MHz, $CDCl_3$) δ 7.94 (d, *J* = 7.7 Hz, 1H), 7.85 (d, *J* = 7.7 Hz, 1H), 7.54 (dd, *J* = 7.2, 3.8 Hz, 1H), 7.47 – 7.43 (m, 2H), 7.36 – 7.33 (m, 2H), 7.29 – 7.22 (m, 3H), 5.51 – 5.28 (m, 1H), 5.22 – 5.14 (m, 1H), 3.67 (td, *J* = 9.4, 3.6 Hz, 1H), 2.56 – 2.20 (m, 4H), 2.16 – 2.12 (m, 1H), 1.91 – 1.76 (m, 2H), 1.65 – 1.55 (m, 3H), 1.50 – 1.34 (m, 2H), 1.26 – 0.80 (m, 4H), 0.75 – 0.66 (m, 1H). **^{1}H NMR** (400 MHz, $CDCl_3$) Another configuration: δ 3.24 (td, *J* = 8.6, 3.7 Hz, 1H). **^{13}C NMR** (101 MHz, $CDCl_3$) δ 202.15, 201.28, 142.46, 142.36, 138.69, 138.08, 137.35, 136.58, 132.75, 132.48, 128.45, 128.41, 128.33, 128.30, 127.59, 127.55, 126.40, 125.93, 60.50, 60.38, 50.53, 50.22, 49.84, 49.78, 48.50, 43.25, 43.21, 42.07, 41.80, 41.73, 40.52, 36.69, 33.21, 32.92, 32.86, 26.06, 25.89, 25.80, 25.56. **HRMS**: calculated for $C_{27}H_{31}O$ [M+H] $^+$ 371.2369; found 371.2357.

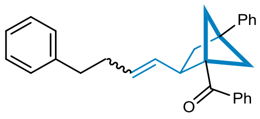

5e

5e, Rf = 0.4 (PE:EA = 12:1). The crude material was purified by flash column chromatography

(using petroleum ether/EtOAc = 15:1) to provide the title compound as a colorless oil in 70% yield (55 mg). **^{1}H NMR** (400 MHz, $CDCl_3$) δ 7.99 – 7.87 (m, 2H), 7.55 (dd, *J* = 7.5, 5.6 Hz, 1H), 7.51 – 7.42 (m, 2H), 7.37 – 7.34 (m, 2H), 7.31 – 7.19 (m, 5H), 7.18 – 7.13 (m, 2H), 7.11 – 7.04 (m, 1H), 7.00 – 6.92 (m, 1H), 5.62 – 5.48 (m, 1H), 5.44 – 5.32 (m, 1H), 3.62 (td, *J* = 9.2, 3.6 Hz, 1H), 2.59 – 2.47 (m, 1H), 2.46 – 1.96 (m, 8H), 1.88 (dt, *J* = 11.0, 3.2 Hz, 1H). **^{1}H NMR** (400 MHz, $CDCl_3$) Another configuration: δ 3.32 (td, *J* = 8.5, 3.7 Hz, 1H), 1.76 (dt, *J* = 11.0, 3.2 Hz, 1H). **^{13}C NMR** (101 MHz, $CDCl_3$) δ 201.94, 201.56, 142.34, 142.21, 141.77, 141.67, 137.11, 136.72, 132.76, 132.60, 131.65, 130.89, 130.82, 130.51, 128.49, 128.42, 128.37, 128.35, 128.31, 128.26, 128.16, 128.09, 126.42, 125.89, 125.68, 125.65, 60.21, 60.18, 50.36, 50.18, 49.84, 49.73, 48.43, 43.04, 42.91, 42.14, 42.09, 41.80, 35.88, 35.61, 34.25, 29.31. **HRMS**: calculated for $C_{29}H_{29}O$ $[M+H]^+$ 393.2213; found 393.2219.

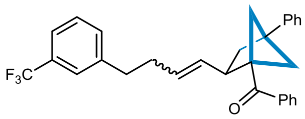

5f

5f, Rf = 0.3 (PE:EA = 10:1). The crude material was purified by flash column chromatography (using petroleum ether/EtOAc = 10:1) to provide the title compound as a colorless oil in 70% yield (64 mg). **^{1}H NMR** (400 MHz, $CDCl_3$) 7.95 (d, *J* = 7.6 Hz, 1H), 7.90 (d, *J* = 7.6 Hz, 1H), 7.59 – 7.53 (m, 1H), 7.52 – 7.45 (m, 2H), 7.43 – 7.41 (m, 1H), 7.39 – 7.21 (m, 7H), 7.21 – 7.09 (m, 1H), 5.64 – 5.50 (m, 1H), 5.45 – 5.24 (m, 1H), 3.60 (td, *J* = 9.4, 3.6 Hz, 1H), 2.64 – 2.51 (m, 1H), 2.50 – 2.20 (m, 6H), 2.18 – 2.14 (m, 1H), 2.13 – 1.95 (m, 1H), 1.87 (dt, *J* = 11.1, 3.2 Hz, 1H). **^{1}H NMR** (400 MHz, $CDCl_3$) Another configuration: δ 3.32 (td, *J* = 8.5, 3.8 Hz, 1H), 1.75 (dt, *J* = 11.1, 3.2 Hz, 1H). **^{13}C NMR** (101 MHz, $CDCl_3$) δ 201.82, 201.39, 142.55, 142.50, 142.24, 142.11, 137.06, 136.65, 132.87, 132.65, 131.78, 131.73, 131.54, 131.02, 130.88, 130.40 (q, $J_{C\text{-}F}$ = 31.9 Hz), 130.32 (q, $J_{C\text{-}F}$ = 32.0 Hz), 130.11, 128.55, 128.52, 128.47, 128.43, 128.35, 128.31, 125.88, 125.03 (q, $J_{C\text{-}F}$ = 3.9 Hz), 124.89 (q, $J_{C\text{-}F}$ = 3.7 Hz), 124.22 (d, $J_{C\text{-}F}$ = 272.4 Hz), 122.59 (q, $J_{C\text{-}F}$ = 3.9 Hz), 121.52 (d, $J_{C\text{-}F}$ = 270.9 Hz), 60.28, 60.18, 50.44, 50.16, 49.89, 49.75, 48.38, 43.00, 42.72, 42.15, 41.93, 41.76, 35.58, 35.37, 33.92, 29.00. **^{19}F NMR** (377 MHz, $CDCl_3$) δ -62.34, -62.43. **HRMS**: calculated for $C_{30}H_{28}F_3O$ $[M+H]^+$ 461.2087; found 461.2100.

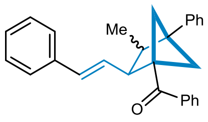

5g

5g, Rf = 0.6 (PE:EA = 10:1). The crude material was purified by flash column chromatography (using petroleum ether/EtOAc = 10:1) to provide the title compound as a white solid in 73% yield (55 mg). **^{1}H NMR** (400 MHz, $CDCl_3$) δ 7.97 – 7.85 (m, 4H), 7.60 – 7.53 (m, 2H), 7.50 – 7.44 (m, 4H), 7.40 – 7.33 (m, 4H), 7.32 – 7.12 (m, 16H), 6.39 – 6.28 (m, 2H), 6.27 – 6.14 (m, 2H), 3.62 – 3.49 (m, 1H), 2.95 (ddd, *J* = 5.7, 3.8, 1.8 Hz, 1H), 2.82 – 2.71 (m, 1H), 2.63 (dd, *J* = 9.3, 6.7 Hz, 1H), 2.59 – 2.48 (m, 3H), 2.39 – 2.28 (m, 3H), 2.26 – 2.15 (m, 2H), 1.09 (d, *J* = 6.8 Hz, 3H), 0.87 (d, *J* = 7.2 Hz, 3H). **^{13}C NMR** (101 MHz, $CDCl_3$) δ 201.70, 141.58, 141.45, 137.15, 137.01, 132.79, 132.62, 132.55, 131.18, 129.77, 128.44, 128.40, 128.35, 128.31, 128.21, 127.85, 127.16, 127.12, 126.30, 126.18, 126.15, 126.10, 125.93, 60.03, 59.49, 57.76, 53.18, 52.97, 52.21, 51.23, 48.33, 45.06, 44.98, 44.05, 36.51, 15.96, 12.89. **HRMS**: calculated for $C_{28}H_{27}O$ $[M+H]^+$ 379.2056; found 379.2055.

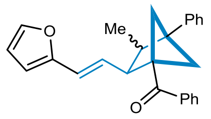

5h

5h, Rf = 0.5 (PE:EA = 10:1). The crude material was purified by flash column chromatography (using petroleum ether/EtOAc = 10:1) to provide the title compound as a white solid in 68% yield (50 mg). **^{1}H NMR** (400 MHz, $CDCl_3$) δ 7.88 (d, *J* = 7.5 Hz, 4H), 7.57 – 7.50 (m, 2H), 7.49 – 7.42 (m, 4H), 7.38 – 7.32 (m, 4H), 7.30 – 7.22 (m, 4H), 7.22 – 7.14 (m, 4H), 6.36 – 6.26 (m, 2H), 6.29 – 5.97 (m, 6H), 3.48 (t, *J* = 9.3 Hz, 1H), 2.92 – 2.83 (m, 1H), 2.79 – 2.66 (m, 1H), 2.59 (dd, *J* = 9.3, 6.7 Hz, 1H), 2.51 (dq, *J* = 9.3, 6.0, 4.6 Hz, 3H), 2.29 (tdd, *J* = 10.5, 5.4, 1.8 Hz, 3H), 2.21 – 2.10 (m, 2H), 1.05 (d, *J* = 6.8 Hz, 3H), 0.84 (d, *J* = 7.2 Hz, 3H). **^{13}C NMR** (101 MHz, $CDCl_3$) δ 201.61, 201.58, 152.54, 152.47, 141.58, 141.51, 141.47, 141.44, 136.93, 136.90, 132.65, 132.58, 128.55, 128.46, 128.41, 128.26, 128.22, 126.55, 126.31, 126.10, 125.93, 121.02, 119.58, 111.07, 111.00, 107.05, 106.93, 60.04, 59.48, 57.61, 53.21, 53.00, 52.10, 51.31, 48.48, 45.09, 44.93, 44.16, 36.48, 15.94, 12.90. **HRMS**: calculated for $C_{26}H_{25}O_2$ $[M+H]^+$ 369.1849; found 369.1847.

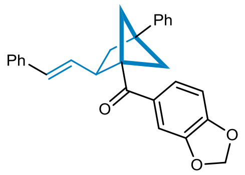

6a

6a, Rf = 0.3 (PE:EA = 10:1). The crude material was purified by flash column chromatography (using petroleum ether/EtOAc = 10:1) to provide the title compound as a white solid in 97% yield (79 mg). **^{1}H NMR** (400 MHz, $CDCl_3$) δ 7.56 (dd, *J* = 8.1, 1.7 Hz, 1H), 7.43 (d, *J* = 1.7 Hz, 1H), 7.38 (t, *J* = 7.5 Hz, 2H), 7.34 – 7.24 (m, 7H), 7.20 (ddd, *J* = 7.7, 3.8, 2.3 Hz, 1H), 6.86 (d, *J* = 8.1 Hz, 1H), 6.42 – 6.22 (m, 2H), 6.03 (s, 2H), 3.58 – 3.44 (m, 1H), 2.53 (ddd, *J* = 11.3, 8.6, 2.8 Hz, 1H), 2.38 (ddt, *J* = 9.1, 6.5, 3.3 Hz, 3H), 2.21 (ddt, *J* = 4.7, 3.0, 1.6 Hz, 1H), 2.11 – 1.99 (m, 1H). **^{13}C NMR** (101 MHz, $CDCl_3$) δ 199.31, 151.41, 147.98, 142.11, 137.01, 131.47, 131.28, 130.46, 128.34, 128.32, 127.16, 126.46, 126.15, 125.87, 124.60, 108.31, 107.78, 101.67, 60.11, 50.24, 49.62, 48.98, 42.56, 41.98. **HRMS**: calculated for $C_{28}H_{25}O_3$ $[M+H]^+$ 409.1798; found 409.1813.

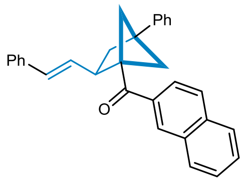

6b

6b, Rf = 0.5 (PE:EA = 10:1). The crude material was purified by flash column chromatography (using petroleum ether/EtOAc = 10:1) to provide the title compound as a white solid in 86% yield (71 mg). **^{1}H NMR** (400 MHz, $CDCl_3$) δ 8.46 (s, 1H), 7.99 (td, *J* = 6.1, 2.9 Hz, 2H), 7.90 (dd, *J* = 8.3, 3.9 Hz, 2H), 7.71 – 7.51 (m, 2H), 7.49 – 7.33 (m, 4H), 7.32 – 7.11 (m, 6H), 6.49 – 6.07 (m, 2H), 3.64 (td, *J* = 8.2, 3.7 Hz, 1H), 2.59 (ddd, *J* = 15.4, 8.8, 4.6 Hz, 2H), 2.52 – 2.40 (m, 2H), 2.30 (dt, *J* = 7.3, 2.1 Hz, 1H), 2.10 (dt, *J* = 11.2, 3.3 Hz, 1H). **^{13}C NMR** (101 MHz, $CDCl_3$) δ 201.53, 142.11, 136.94, 135.33, 134.28, 132.43, 131.40, 130.42, 129.77, 129.51, 128.36, 128.32, 128.29, 127.74, 127.15, 126.64, 126.52, 126.14, 125.92, 124.32, 60.52, 50.43, 49.88, 48.97, 42.45, 41.96. **HRMS**: calculated for $C_{31}H_{27}O$ $[M+H]^+$ 415.2056; found 415.2069.

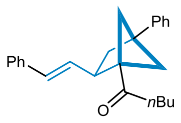

6c

6c, Rf = 0.5 (PE:EA = 10:1). The crude material was purified by flash column chromatography (using petroleum ether/EtOAc = 10:1) to provide the title compound as a white solid in 53% yield (36 mg). **^{1}H NMR** (400 MHz, $CDCl_3$) δ 7.55 – 7.16 (m, 10H), 6.50 (d, *J* = 15.7 Hz, 1H), 6.26 (dd, *J* = 15.7, 8.8 Hz, 1H), 3.33 (td, *J* = 8.7, 3.7 Hz, 1H), 2.54 – 2.36 (m, 3H), 2.13 (td, *J* = 7.0, 3.7 Hz, 1H), 2.06 (dt, *J* = 5.6, 3.0 Hz, 3H), 1.91 (ddd, *J* = 11.0, 4.0, 2.1 Hz, 1H), 1.66 – 1.46 (m, 2H), 1.35 – 1.19 (m, 2H), 0.87 (t, *J* = 7.3 Hz, 3H). **^{13}C NMR** (101 MHz, $CDCl_3$) δ 211.30, 142.21, 137.04, 131.32, 130.75, 128.49, 128.32, 127.31, 126.49, 126.17, 125.85, 60.52, 49.74, 48.59, 47.08, 41.84, 40.42, 38.89, 25.10, 22.35, 13.85. **HRMS**: calculated for $C_{25}H_{29}O$ $[M+H]^+$ 345.2213; found 345.2227.

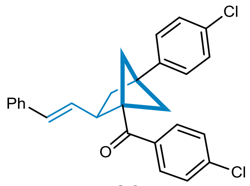

6d

6d, Rf = 0.4 (PE:EA = 10:1). The crude material was purified by flash column chromatography (using petroleum ether/EtOAc = 15:1) to provide the title compound as a white solid in 83% yield (72 mg). **^{1}H NMR** (400 MHz, $CDCl_3$) δ 7.81 (d, *J* = 8.2 Hz, 2H), 7.41 (d, *J* = 8.2 Hz, 2H), 7.31 (d, *J* = 8.1 Hz, 2H), 7.24 (ddd, *J* = 14.8, 9.6, 6.4 Hz, 7H), 6.39 – 6.08 (m, 2H), 3.48 (td, *J* = 8.1, 3.7 Hz, 1H), 2.48 (ddd, *J* = 11.3, 8.5, 2.8 Hz, 1H), 2.41 – 2.28 (m, 3H), 2.17 (d, *J* = 6.9 Hz, 1H), 1.99 (dt, *J* = 11.2, 3.3 Hz, 1H). **^{13}C NMR** (101 MHz, $CDCl_3$) δ 200.21, 140.40, 139.19, 136.80, 135.17, 132.37, 131.74, 129.86, 129.74, 128.88, 128.54, 128.45, 127.39, 127.32, 126.18, 60.22, 50.16, 49.41, 48.83, 42.38, 41.90. **HRMS**: calculated for $C_{27}H_{23}Cl_2O$ $[M+H]^+$ 433.1120; found 433.1129.

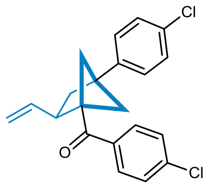

6e

6e, Rf = 0.4 (PE:EA = 10:1). The crude material was purified by flash column chromatography (using petroleum ether/EtOAc = 15:1) to provide the title compound as a white solid in 77% yield (55 mg). **^{1}H NMR** (400 MHz, $CDCl_3$) δ 7.82 (d, *J* = 8.5 Hz, 2H), 7.42 (d, *J* = 8.5 Hz, 2H), 7.30 (d, *J* = 8.5 Hz, 2H), 7.18 (d, *J* = 8.4 Hz, 2H), 5.83 (ddd, *J* = 16.9, 10.3, 8.3 Hz, 1H), 5.14 – 4.79 (m, 2H), 3.31 (td, *J* = 8.3, 3.5 Hz, 1H), 2.40 (ddd, *J* = 11.3, 8.6, 2.9 Hz, 1H), 2.35 – 2.26 (m, 2H), 2.22 (dd, *J* = 8.9, 7.2 Hz, 1H), 2.10 (ddd, *J* = 7.2, 2.9, 1.6 Hz, 1H), 1.91 (ddd, *J* = 11.1, 3.9, 2.7 Hz, 1H). **^{13}C NMR** (101 MHz, $CDCl_3$) δ 200.15, 140.45, 139.21, 138.11, 135.07, 132.31, 129.77, 128.86, 128.50, 127.31, 116.68, 59.89, 50.22, 49.37, 49.31, 42.12, 41.13. **HRMS**: calculated for $C_{21}H_{19}Cl_2O$ $[M+H]^+$ 357.0807; found 357.0811.

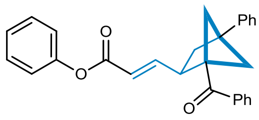

9a

9a, Rf = 0.4 (PE:EA = 10:1). The crude material was purified by flash column chromatography (using petroleum ether/EtOAc = 10:1) to provide the title compound as a white solid in 81% yield (66 mg). ^{1}**H NMR** (400 MHz, $CDCl_3$) δ 7.96 – 7.87 (m, 2H), 7.62 – 7.56 (m, 1H), 7.49 (dd, *J* = 8.4, 6.9 Hz, 2H), 7.40 – 7.34 (m, 4H), 7.33 – 7.25 (m, 3H), 7.25 – 7.16 (m, 2H), 7.10 – 7.04 (m, 2H), 5.92 (dd, *J* = 15.6, 1.0 Hz, 1H), 3.58 (td, *J* = 8.8, 3.7 Hz, 1H), 2.54 (ddd, *J* = 11.4, 8.6, 2.9 Hz, 1H), 2.48 – 2.33 (m, 3H), 2.27 (ddd, *J* = 7.2, 2.9, 1.6 Hz, 1H), 2.08 (ddd, *J* = 11.3, 3.9, 2.2 Hz, 1H). ^{13}**C NMR** (101 MHz, $CDCl_3$) δ 200.71, 164.25, 150.55, 150.44, 141.37, 136.62, 133.04, 129.30, 128.65, 128.43, 128.28, 126.73, 125.82, 125.68, 121.84, 121.51, 59.97, 50.09, 47.72, 42.40, 41.41. **HRMS**: calculated for $C_{28}H_{25}O_3$ $[M+H]^+$ 409.1798; found 409.1794.

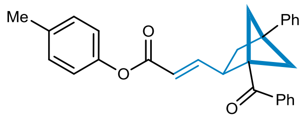

9b

9b, Rf = 0.4 (PE:EA = 10:1). The crude material was purified by flash column chromatography (using petroleum ether/EtOAc = 10:1) to provide the title compound as a white solid in 84% yield (71 mg). ^{1}**H NMR** (400 MHz, $CDCl_3$) δ 7.98 – 7.85 (m, 2H), 7.62 – 7.56 (m, 1H), 7.49 (dd, *J* = 8.4, 6.9 Hz, 2H), 7.37 (dd, *J* = 8.0, 6.8 Hz, 2H), 7.33 – 7.24 (m, 3H), 7.24 – 7.13 (m, 3H), 7.00 – 6.90 (m, 2H), 5.91 (dd, *J* = 15.6, 0.9 Hz, 1H), 3.57 (td, *J* = 8.8, 3.7 Hz, 1H), 2.54 (ddd, *J* = 11.4, 8.7, 2.8 Hz, 1H), 2.47 – 2.36 (m, 3H), 2.34 (s, 3H), 2.27 (ddd, *J* = 7.3, 2.9, 1.6 Hz, 1H), 2.08 (ddd, *J* = 11.3, 4.0, 2.3 Hz, 1H). ^{13}**C NMR** (101 MHz, $CDCl_3$) δ 200.70, 164.47, 150.22, 148.29, 141.38, 136.61, 135.28, 133.02, 129.81, 128.64, 128.43, 128.28, 126.72, 125.81, 121.91, 121.16, 59.96, 50.09, 50.06, 47.72, 42.38, 41.41, 20.81. **HRMS**: calculated for $C_{29}H_{27}O_3$ $[M+H]^+$ 423.1955; found 423.1954.

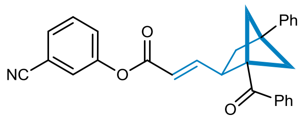

9c

9c, Rf = 0.3 (PE:EA = 10:1). The crude material was purified by flash column chromatography (using petroleum ether/EtOAc = 10:1) to provide the title compound as a white solid in 77% yield (67 mg). ^{1}**H NMR** (400 MHz, $CDCl_3$) δ 7.95 – 7.85 (m, 2H), 7.63 – 7.56 (m, 1H), 7.54 – 7.44 (m, 4H), 7.42 – 7.31 (m, 4H), 7.31 – 7.17 (m, 4H), 5.91 (dd, *J* = 15.5, 0.9 Hz, 1H), 3.58 (td, *J* = 8.8, 3.7 Hz, 1H), 2.55 (ddd, *J* = 11.4, 8.6, 2.8 Hz, 1H), 2.43 (dd, *J* = 5.2, 2.4 Hz, 2H), 2.40 – 2.33 (m, 1H), 2.27 (ddd, *J* = 7.4, 2.9, 1.6 Hz, 1H), 2.10 – 2.04 (m, 1H). ^{13}**C NMR** (101 MHz, $CDCl_3$) δ 200.61, 163.52, 151.92, 150.64, 141.18, 136.50, 133.15, 130.26, 129.41, 128.70, 128.46, 128.28, 126.80, 126.58, 125.80, 125.33, 120.95, 117.82, 113.30, 59.97, 50.12, 50.06, 47.77, 42.45, 41.40. **HRMS**: calculated for $C_{29}H_{24}NO_3$ $[M+H]^+$ 434.1751; found 434.1747.

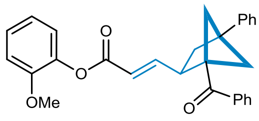

9d

9d, Rf = 0.4 (PE:EA = 10:1). The crude material was purified by flash column chromatography (using petroleum ether/EtOAc = 10:1) to provide the title compound as a white solid in 85% yield (74 mg). **^{1}H NMR** (400 MHz, $CDCl_3$) δ 7.95 – 7.86 (m, 2H), 7.62 – 7.55 (m, 1H), 7.53 – 7.45 (m, 2H), 7.40 – 7.33 (m, 2H), 7.31 – 7.24 (m, 3H), 7.24 – 7.16 (m, 2H), 7.01 (dd, *J* = 7.8, 1.7 Hz, 1H), 6.97 – 6.91 (m, 2H), 5.96 (dd, *J* = 15.6, 1.0 Hz, 1H), 3.79 (s, 3H), 3.64 – 3.51 (m, 1H), 2.53 (ddd, *J* = 11.4, 8.7, 2.8 Hz, 1H), 2.46 – 2.32 (m, 3H), 2.26 (ddd, *J* = 7.3, 2.9, 1.5 Hz, 1H), 2.13 – 2.05 (m, 1H). **^{13}C NMR** (101 MHz, $CDCl_3$) δ 200.72, 163.86, 151.10, 150.34, 141.45, 139.56, 136.65, 132.99, 128.63, 128.43, 128.29, 126.77, 126.71, 125.83, 122.81, 121.57, 120.68, 112.35, 59.93, 55.78, 50.15, 50.05, 47.75, 42.36, 41.37. **HRMS**: calculated for $C_{29}H_{27}O_4$ $[M+H]^+$ 439.1904; found 439.1900.

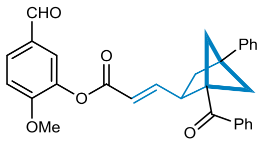

9e

9e, Rf = 0.3 (PE:EA = 10:1). The crude material was purified by flash column chromatography (using petroleum ether/EtOAc = 10:1) to provide the title compound as a white solid in 73% yield (68 mg). **^{1}H NMR** (400 MHz, $CDCl_3$) δ 9.85 (s, 1H), 7.94 – 7.87 (m, 2H), 7.75 (dd, *J* = 8.5, 2.0 Hz, 1H), 7.63 – 7.53 (m, 2H), 7.48 (t, *J* = 7.6 Hz, 2H), 7.36 (t, *J* = 7.5 Hz, 2H), 7.31 – 7.17 (m, 4H), 7.05 (d, *J* = 8.5 Hz, 1H), 5.94 (d, *J* = 15.6 Hz, 1H), 3.86 (s, 3H), 3.58 (td, *J* = 8.8, 3.6 Hz, 1H), 2.54 (ddd, *J* = 11.4, 8.6, 2.8 Hz, 1H), 2.47 – 2.31 (m, 3H), 2.26 (q, *J* = 2.4 Hz, 1H), 2.08 (dt, *J* = 11.6, 3.0 Hz, 1H). **^{13}C NMR** (101 MHz, $CDCl_3$) δ 200.65, 190.00, 163.44, 156.33, 151.19, 141.31, 139.99, 136.58, 133.05, 129.91, 129.82, 128.65, 128.42, 128.25, 126.73, 125.81, 123.57, 121.00, 111.93, 59.91, 56.13, 50.08, 50.05, 47.75, 42.37, 41.33. **HRMS**: calculated for $C_{30}H_{27}O_5$ $[M+H]^+$ 467.1853; found 467.1851.

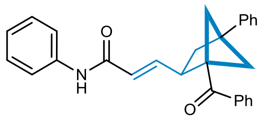

9f

9f, Rf = 0.3 (PE:EA = 5:1). The crude material was purified by flash column chromatography (using petroleum ether/EtOAc = 5:1) to provide the title compound as a white solid in 77% yield (63 mg). **^{1}H NMR** (400 MHz, $CDCl_3$) δ 7.96 – 7.77 (m, 2H), 7.54 (q, *J* = 7.5 Hz, 3H), 7.48 – 7.39 (m, 3H), 7.35 (dd, *J* = 8.6, 6.5 Hz, 2H), 7.26 (td, *J* = 8.3, 7.7, 5.0 Hz, 5H), 7.04 (dt, *J* = 25.4, 8.1 Hz, 2H), 5.83 (d, *J* = 15.1 Hz, 1H), 3.47 (td, *J* = 9.0, 3.7 Hz, 1H), 2.45 (ddd, *J* = 11.4, 8.5, 2.7 Hz, 1H), 2.35 (d, *J* = 2.8 Hz, 3H), 2.26 – 2.15 (m, 1H), 1.99 (ddd, *J* = 11.3, 4.0, 2.1 Hz, 1H). **^{13}C NMR** (101 MHz, $CDCl_3$) δ 201.47, 163.46, 145.50, 141.48, 137.80, 136.72, 133.01, 128.93, 128.62, 128.40, 128.32, 126.67, 125.82, 125.05, 124.30, 119.84, 59.83, 50.24, 50.17, 47.53, 42.46, 41.47. **HRMS**: calculated for $C_{28}H_{26}NO_2$ $[M+H]^+$ 408.1958; found 408.1955.

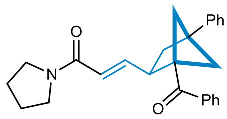

9g

9g, Rf = 0.2 (PE:EA = 2:1). The crude material was purified by flash column chromatography (using petroleum ether/EtOAc = 2:1) to provide the title compound as a white solid in 85% yield (65 mg). **^{1}H NMR** (400 MHz, $CDCl_3$) δ 7.92 – 7.81 (m, 2H), 7.57 – 7.49 (m, 1H), 7.44 (dd, *J* = 8.3,

6.8 Hz, 2H), 7.32 (dd, J = 8.0, 6.9 Hz, 2H), 7.28 – 7.19 (m, 3H), 6.91 (dd, J = 15.1, 8.7 Hz, 1H), 5.94 (dd, J = 15.2, 0.9 Hz, 1H), 3.49 (td, J = 8.8, 3.7 Hz, 1H), 3.46 – 3.38 (m, 2H), 3.28 (dt, J = 10.1, 6.6 Hz, 1H), 3.17 (dt, J = 10.0, 6.5 Hz, 1H), 2.46 (ddd, J = 11.3, 8.7, 2.8 Hz, 1H), 2.40 – 2.27 (m, 3H), 2.22 (ddd, J = 6.8, 2.9, 1.5 Hz, 1H), 2.00 (ddd, J = 11.2, 3.9, 2.5 Hz, 1H), 1.94 – 1.69 (m, 4H). **^{13}C NMR** (101 MHz, $CDCl_3$) δ 201.12, 164.07, 144.32, 141.65, 136.83, 132.68, 128.42, 128.29, 128.21, 126.51, 125.73, 123.05, 59.90, 50.35, 50.08, 47.61, 46.24, 45.60, 42.10, 41.52, 25.86, 24.11. **HRMS**: calculated for $C_{26}H_{28}NO_2$ $[M+H]^+$ 386.2115; found 386.2129.

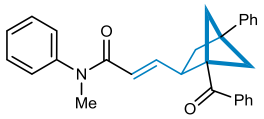

9h

9h, Rf = 0.4 (PE:EA = 5:1). The crude material was purified by flash column chromatography (using petroleum ether/EtOAc = 5:1) to provide the title compound as a white solid in 82% yield (69 mg). **^{1}H NMR** (400 MHz, $CDCl_3$) δ 7.81 (d, J = 7.6 Hz, 2H), 7.57 – 7.52 (m, 1H), 7.48 – 7.42 (m, 2H), 7.34 – 7.28 (m, 2H), 7.28 – 7.18 (m, 6H), 7.04 – 6.80 (m, 3H), 5.62 (d, J = 14.1 Hz, 1H), 3.36 – 3.26 (m, 4H), 2.46 – 2.22 (m, 4H), 2.18 (d, J = 6.1 Hz, 1H), 1.93 (dt, J = 11.2, 2.7 Hz, 1H). **^{13}C NMR** (101 MHz, $CDCl_3$) δ 200.80, 144.83, 143.19, 141.68, 136.66, 132.68, 129.27, 128.43, 128.31, 128.26, 127.13, 126.90, 126.53, 125.75, 122.93, 59.96, 50.09, 50.00, 47.72, 42.21, 41.49, 37.21. **HRMS**: calculated for $C_{29}H_{28}NO_2$ $[M+H]^+$ 422.2115; found 422.2111.

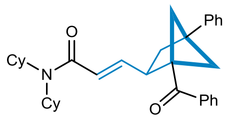

9i

9i, Rf = 0.4 (PE:EA = 4:1). The crude material was purified by flash column chromatography (using petroleum ether/EtOAc = 5:1) to provide the title compound as a white solid in 87% yield (86 mg). **^{1}H NMR** (400 MHz, $CDCl_3$) δ 7.97 – 7.78 (m, 2H), 7.57 – 7.52 (m, 1H), 7.46 – 7.42 (m, 2H), 7.35 – 7.31 (m, 2H), 7.29 – 7.19 (m, 3H), 6.75 (dd, J = 15.1, 8.8 Hz, 1H), 6.04 (d, J = 15.1 Hz, 1H), 3.52 (td, J = 8.8, 3.7 Hz, 1H), 3.15 (br, 2H), 2.47 (ddd, J = 11.3, 8.6, 2.8 Hz, 1H), 2.40 – 2.31 (m, 3H), 2.22 – 2.18 (m, 2H), 2.06 – 2.02 (m, 1H), 1.83 – 1.63 (m, 4H), 1.57 (d, J = 11.5 Hz, 2H), 1.53 – 1.37 (m, 5H), 1.34 – 0.95 (m, 8H). **^{13}C NMR** (101 MHz, $CDCl_3$) δ 201.06, 165.97, 142.97, 141.80, 136.68, 132.74, 128.49, 128.32, 126.52, 125.81, 125.02, 60.14, 55.46, 50.42, 50.11, 47.98, 42.11, 41.59, 31.49, 30.26, 29.84, 26.34, 25.81, 25.33, 25.06. **HRMS**: calculated for $C_{34}H_{42}NO_2$ $[M+H]^+$ 496.3210; found 496.3207.

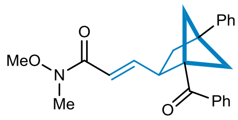

9j

9j, Rf = 0.4 (PE:EA = 2:1). The crude material was purified by flash column chromatography (using petroleum ether/EtOAc = 5:1) to provide the title compound as a white solid in 74% yield (56 mg). **^{1}H NMR** (400 MHz, $CDCl_3$) δ 7.87 (dt, J = 7.1, 1.4 Hz, 2H), 7.63 – 7.49 (m, 1H), 7.45 (dd, J = 8.2, 6.8 Hz, 2H), 7.35 (dd, J = 8.0, 6.8 Hz, 2H), 7.31 – 7.20 (m, 3H), 7.00 (dd, J = 15.4, 8.9 Hz, 1H), 6.26 (d, J = 15.4 Hz, 1H), 3.54 (td, J = 8.8, 3.8 Hz, 1H), 3.45 (s, 3H), 3.16 (s, 3H), 2.49 (ddd, J = 11.3,

8.6, 2.8 Hz, 1H), 2.43 – 2.28 (m, 3H), 2.24 (ddd, *J* = 7.1, 2.9, 1.6 Hz, 1H), 2.03 (ddd, *J* = 11.2, 4.0, 2.6 Hz, 1H). **^{13}C NMR** (101 MHz, $CDCl_3$) δ 200.94, 166.07, 146.64, 141.65, 136.75, 132.78, 128.50, 128.37, 128.30, 126.61, 125.81, 120.05, 61.47, 60.01, 50.38, 50.13, 47.81, 42.14, 41.53, 32.20. **HRMS**: calculated for $C_{24}H_{26}NO_3$ [M+H] $^+$ 376.1907; found 376.1905.

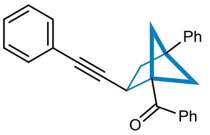

10a

10a, Rf = 0.4 (PE:EA = 10:1). The crude material was purified by flash column chromatography (using petroleum ether/EtOAc = 14:1) to provide the title compound as a colorless oil in 87% yield (63 mg). **^{1}H NMR** (400 MHz, $CDCl_3$) δ 8.02 (d, *J* = 7.2 Hz, 2H),7.59 – 7.54 (m, 1H), 7.50 – 7.43 (m, 2H), 7.39 – 7.34 (m, 2H), 7.33 – 7.28 (m, 2H), 7.28 – 7.11 (m, 6H), 3.64 (ddd, *J* = 9.0, 3.9, 1.7 Hz, 1H), 2.70 – 2.49 (m, 2H), 2.40 – 2.29 (m, 3H), 2.26 (dt, *J* = 10.9, 3.0 Hz, 1H). **^{13}C NMR** (101 MHz, $CDCl_3$) δ 200.80, 141.50, 137.01, 132.77, 131.54, 128.66, 128.40, 128.08, 127.81, 126.65, 125.89, 123.23, 90.78, 83.42, 59.66, 49.93, 48.88, 44.06, 43.51, 36.79. **HRMS**: calculated for $C_{27}H_{23}O$ [M+H] $^+$ 363.1743; found 363.1741.

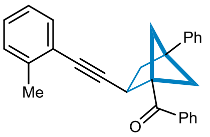

10b

10b, Rf = 0.6 (PE:EA = 8:1). The crude material was purified by flash column chromatography (using petroleum ether/EtOAc = 15:1) to provide the title compound as a white solid in 84% yield (63 mg). **^{1}H NMR** (400 MHz, $CDCl_3$) δ 7.99 (d, *J* = 7.8 Hz, 2H), 7.60 – 7.53 (m, 1H), 7.50 – 7.44 (m, 2H), 7.39 – 7.35 (m, 2H), 7.31 (d, *J* = 7.9 Hz, 2H), 7.28 – 7.22 (m, 2H), 7.20 – 7.12 (m, 2H), 7.10 – 7.05 (m, 1H), 3.78 – 3.66 (m, 1H), 2.67 (ddd, *J* = 11.2, 8.9, 2.7 Hz, 1H), 2.62 – 2.54 (m, 1H), 2.39 – 2.17 (m, 7H). **^{13}C NMR** (101 MHz, $CDCl_3$) δ 200.56, 141.52, 140.04, 136.92, 132.75, 131.83, 129.21, 128.55, 128.43, 128.40, 127.79, 126.66, 125.90, 125.29, 123.01, 94.58, 82.19, 59.83, 49.97, 49.18, 43.74, 36.99, 20.64. **HRMS**: calculated for $C_{28}H_{25}O$ [M+H] $^+$ 377.1900; found 377.1899.

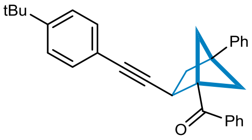

10c

10c, Rf = 0.6 (PE:EA = 8:1). The crude material was purified by flash column chromatography (using petroleum ether/EtOAc = 15:1) to provide the title compound as a white solid in 75% yield (63 mg). **^{1}H NMR** (400 MHz, $CDCl_3$) δ 8.12 – 7.95 (m, 2H), 7.61 – 7.55 (m, 1H), 7.51 – 7.45 (m, 2H), 7.40 – 7.33 (m, 2H), 7.32 – 7.25 (m, 5H), 7.23 (d, *J* = 8.3 Hz, 2H), 3.65 (ddd, *J* = 8.8, 3.9, 1.7 Hz, 1H), 2.73 – 2.47 (m, 2H), 2.41 – 2.12 (m, 4H), 1.31 (s, 9H). **^{13}C NMR** (101 MHz, $CDCl_3$) δ 200.84, 150.98, 141.55, 137.06, 132.70, 131.23, 128.65, 128.37, 126.61, 125.87, 125.06, 120.23, 90.07, 83.45, 59.65, 49.92, 48.92, 44.03, 43.57, 36.82, 34.63, 31.12. **HRMS**: calculated for $C_{31}H_{31}O$ [M+H] $^+$ 419.2369; found 419.2370.

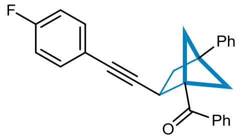

10d

10d, Rf = 0.6 (PE:EA = 8:1). The crude material was purified by flash column chromatography (using petroleum ether/EtOAc = 15:1) to provide the title compound as a white solid in 91% yield (69 mg). **^{1}H NMR** (400 MHz, $CDCl_3$) δ 8.00 (d, *J* = 7.3 Hz, 2H), 7.59 – 7.55 (m, 1H), 7.48 – 7.44 (m, 2H), 7.38 – 7.34 (m, 2H), 7.32 – 7.17 (m, 5H), 6.95 – 6.91 (m, 2H), 3.62 (ddd, *J* = 8.9, 4.0, 1.7 Hz, 1H), 2.69 – 2.47 (m, 2H), 2.36 – 2.27 (m, 3H), 2.24 (dt, *J* = 10.8, 3.1 Hz, 1H). **^{13}C NMR** (101 MHz, $CDCl_3$) δ 200.69, 162.19 (d, $J_{C\text{-}F}$ = 248.6 Hz), 141.43, 136.99, 133.37 (d, $J_{C\text{-}F}$ = 8.5 Hz), 132.80, 128.62, 128.41, 126.68, 125.87, 119.30 (d, $J_{C\text{-}F}$ = 3.5 Hz), 115.41, 115.19, 90.42, 82.38, 59.67, 49.94, 48.86, 44.06, 43.43, 36.75. **^{19}F NMR** (377 MHz, $CDCl_3$) δ -111.65. **HRMS**: calculated for $C_{27}H_{22}FO$ $[M+H]^+$ 381.1649; found 381.1648.

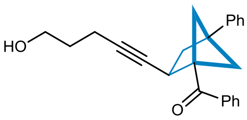

10e

10e, Rf = 0.2 (PE:EA = 4:1). The crude material was purified by flash column chromatography (using petroleum ether/EtOAc = 4:1) to provide the title compound as a colorless oil in 94% yield (65 mg). **^{1}H NMR** (400 MHz, $CDCl_3$) δ 8.00 – 7.91 (m, 2H), 7.60 – 7.53 (m, 1H), 7.46 (dd, *J* = 8.4, 6.9 Hz, 2H), 7.33 (dd, *J* = 8.3, 6.8 Hz, 2H), 7.28 – 7.21 (m, 3H), 3.68 – 3.60 (m, 2H), 3.39 (ddd, *J* = 8.6, 4.0, 2.0 Hz, 1H), 2.57 – 2.41 (m, 2H), 2.32 – 2.18 (m, 5H), 2.08 (dt, *J* = 10.8, 3.3 Hz, 1H), 1.91 (s, 1H), 1.67 – 1.60 (m, 2H). **^{13}C NMR** (101 MHz, $CDCl_3$) δ 201.02, 141.58, 136.78, 132.82, 128.55, 128.40, 128.34, 126.57, 125.85, 82.68, 81.65, 61.46, 59.75, 49.84, 48.88, 43.70, 43.42, 36.44, 31.21, 15.21. **HRMS**: calculated for $C_{24}H_{25}O_2$ $[M+H]^+$ 345.1849; found 345.1850.

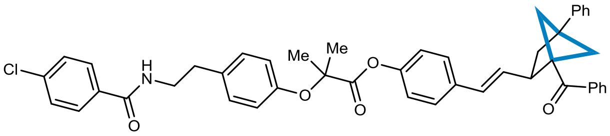

11a

11a, Rf = 0.2 (PE:EA = 4:1). The crude material was purified by flash column chromatography (using petroleum ether/EtOAc = 4:1) to provide the title compound as a white solid in 63% yield (91 mg). **^{1}H NMR** (400 MHz, $CDCl_3$) δ 7.98 – 7.82 (m, 2H), 7.67 – 7.57 (m, 2H), 7.59 – 7.50 (m, 1H), 7.47 – 7.42 (m, 2H), 7.39 – 7.22 (m, 7H), 7.22 – 7.16 (m, 2H), 7.12 (d, *J* = 8.5 Hz, 2H), 6.97 – 6.85 (m, 4H), 6.33 (t, *J* = 5.9 Hz, 1H), 6.26 – 6.15 (m, 2H), 3.69 – 3.64 (m, 2H), 3.53 – 3.48 (m, 1H), 2.87 (t, *J* = 6.9 Hz, 2H), 2.51 (ddd, *J* = 11.3, 8.6, 2.8 Hz, 1H), 2.38 (ddt, *J* = 18.9, 16.1, 7.5 Hz, 3H), 2.25 – 2.16 (m, 1H), 2.02 (dq, *J* = 11.3, 3.6 Hz, 1H), 1.73 (s, 6H). **^{13}C NMR** (101 MHz, $CDCl_3$) δ 201.72, 172.81, 166.63, 154.04, 149.57, 141.99, 137.62, 136.95, 135.09, 132.73, 132.57, 130.92, 130.27, 129.56, 128.74, 128.49, 128.34, 128.26, 128.24, 127.10, 126.52, 125.87, 121.09, 119.38, 79.20, 60.30, 50.19, 49.84, 48.76, 42.33, 41.88, 41.29, 34.60, 25.36. **HRMS**: calculated for $C_{46}H_{43}ClNO_5$ $[M+H]^+$ 724.2824; found 724.2844.

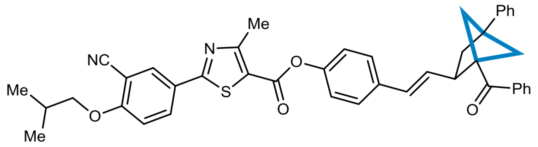

11b

11b, Rf = 0.3 (PE:EA = 4:1). The crude material was purified by flash column chromatography (using petroleum ether/EtOAc = 4:1) to provide the title compound as a white solid in 67% yield (91 mg)**.** **^{1}H NMR** (400 MHz, $CDCl_3$) δ 8.22 (d, *J* = 2.3 Hz, 1H), 8.12 (dd, *J* = 8.9, 2.4 Hz, 1H), 7.89 (dd, *J* = 8.5, 1.4 Hz, 2H), 7.59 – 7.53 (m, 1H), 7.46 (dd, *J* = 8.3, 6.9 Hz, 2H), 7.40 – 7.21 (m, 7H), 7.15 – 7.07 (m, 2H), 7.03 (d, *J* = 8.9 Hz, 1H),6.31 – 6.19 (m, 2H), 3.91 (d, *J* = 6.5 Hz, 2H), 3.57 – 3.47 (m, 1H), 2.81 (s, 3H), 2.52 (ddd, *J* = 11.2, 8.6, 2.7 Hz, 1H), 2.46 – 2.33 (m, 3H), 2.28 – 2.15 (m, 2H), 2.03 (dt, *J* = 11.4, 2.7 Hz, 1H), 1.10 (d, *J* = 6.7 Hz, 6H). **^{13}C NMR** (101 MHz, $CDCl_3$) δ 201.67, 168.05, 162.91, 162.60, 160.29, 149.27, 142.01, 137.00, 135.19, 132.71, 132.61, 132.13, 130.98, 130.33, 128.49, 128.34, 128.26, 127.15, 126.52, 125.87, 125.77, 121.46, 120.54, 115.28, 112.63, 102.99, 75.66, 60.32, 50.19, 49.86, 48.77, 42.35, 41.89, 28.09, 18.99, 17.63. **HRMS**: calculated for $C_{43}H_{39}N_2O_4S$ $[M+H]^+$ 679.2625; found 679.2647.

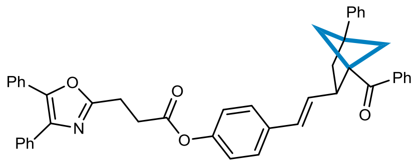

11c

11c, Rf = 0.2 (PE:EA = 5:1). The crude material was purified by flash column chromatography (using petroleum ether/EtOAc = 5:1) to provide the title compound as a white solid in 72% yield (94 mg)**.** **^{1}H NMR** (400 MHz, $CDCl_3$) δ 7.98 – 7.81 (m, 2H), 7.69 – 7.61 (m, 2H), 7.55 (dd, *J* = 8.6, 6.1 Hz, 3H), 7.50 – 7.40 (m, 2H), 7.40 – 7.28 (m, 10H), 7.28 – 7.23 (m, 1H), 7.23 – 7.17 (m, 2H), 6.99 (d, *J* = 8.6 Hz, 2H), 6.26 – 6.16 (m, 2H), 3.57 – 3.43 (m, 1H), 3.31 (t, *J* = 7.3 Hz, 2H), 3.14 (t, *J* = 7.3 Hz, 2H), 2.51 (ddd, *J* = 11.3, 8.6, 2.7 Hz, 1H), 2.39 (tdd, *J* = 16.0, 8.9, 6.7 Hz, 3H), 2.22 (dt, *J* = 7.2, 2.1 Hz, 1H), 2.02 (dt, *J* = 10.0, 2.7 Hz, 1H). **^{13}C NMR** (101 MHz, $CDCl_3$) δ 201.69, 170.50, 149.68, 145.64, 142.06, 137.04, 134.92, 132.69, 130.75, 130.42, 128.63, 128.56, 128.52, 128.49, 128.36, 128.27, 128.09, 127.08, 126.52, 126.43, 125.90, 121.41, 60.32, 50.21, 49.87, 48.77, 42.35, 41.90, 31.23, 23.47. **HRMS**: calculated for $C_{45}H_{38}NO_4$ $[M+H]^+$ 656.2795; found 656.2814.

3 Supplementary Figures

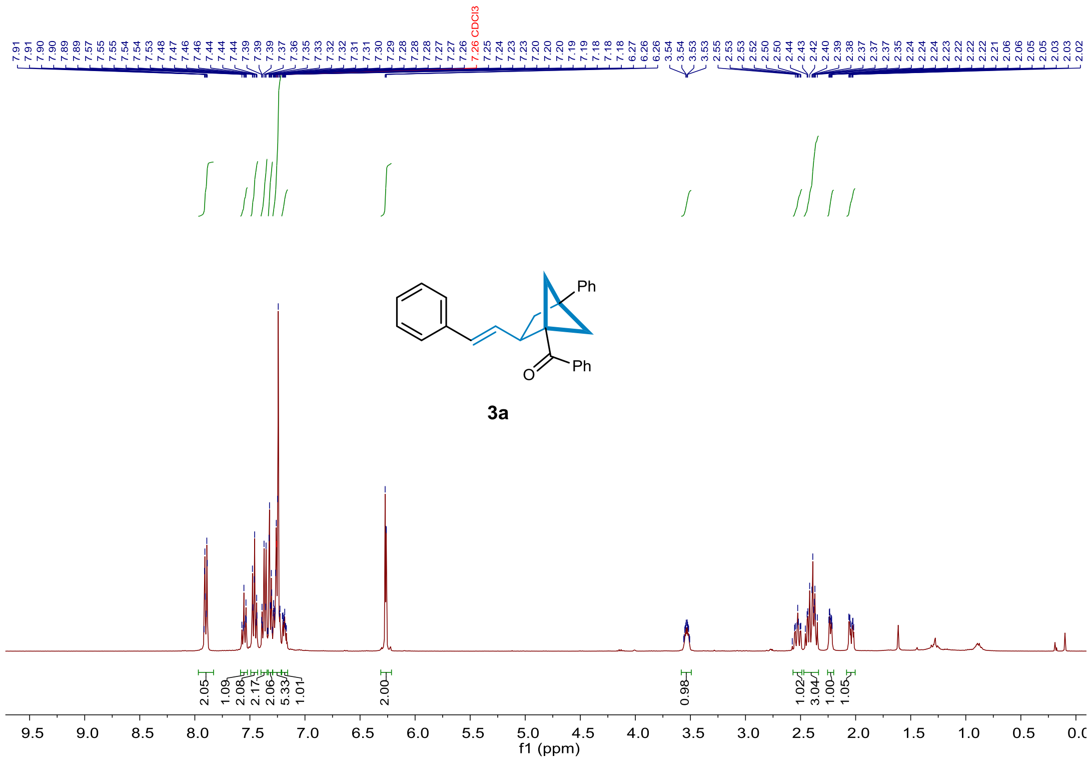

Supplementary Figure 15. ^{1}H NMR of the 3a (400 MHz, $CDCl_3$)

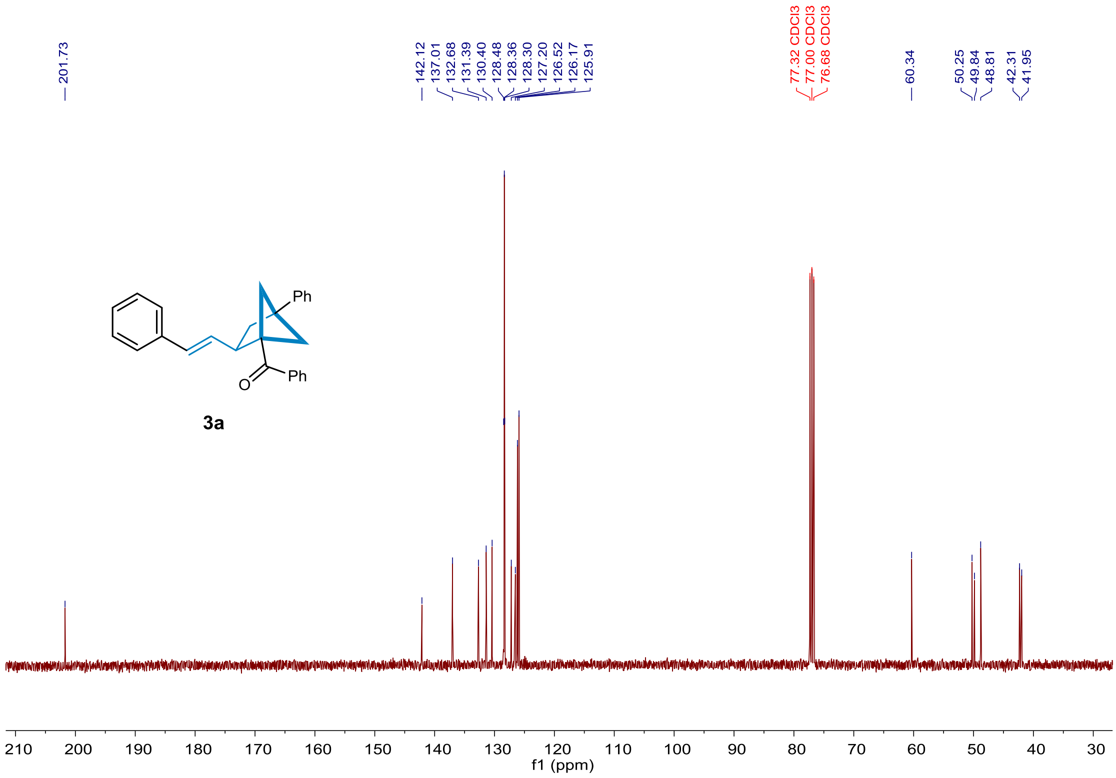

Supplementary Figure 16. ^{13}C NMR of the 3a (101 MHz, $CDCl_3$)

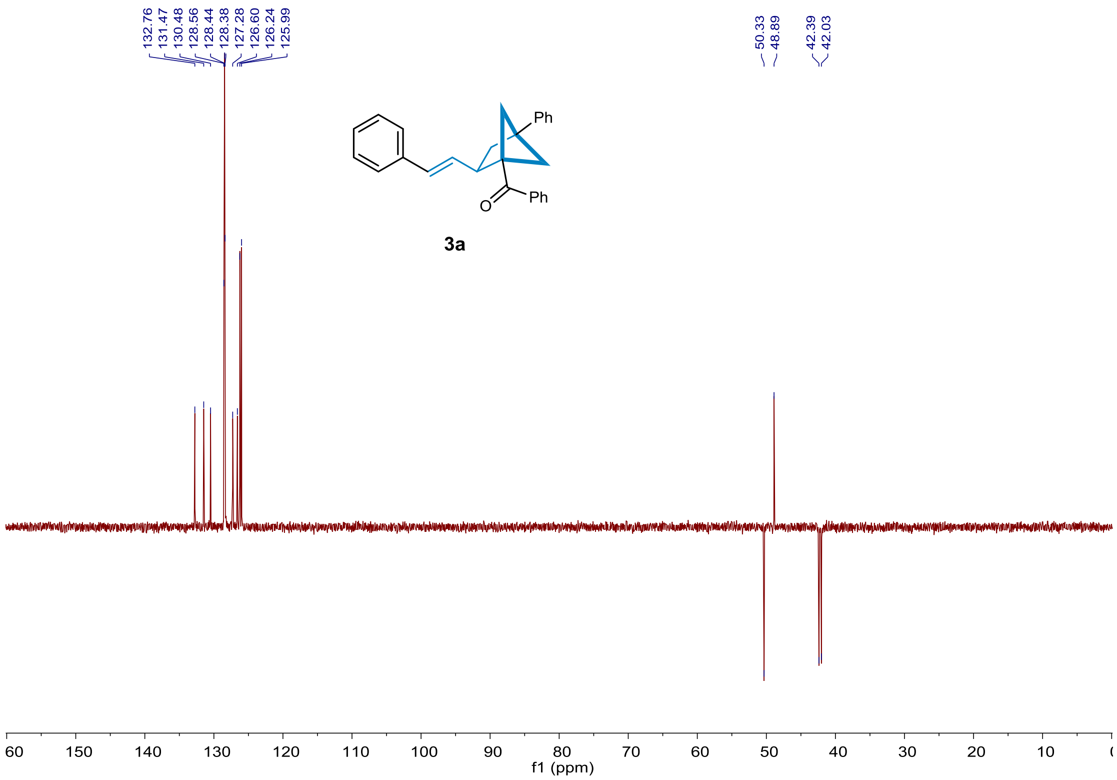

Supplementary Figure 17. **^{13}C NMR-DEPT 135 of the 3a** (101 MHz, $CDCl_3$)

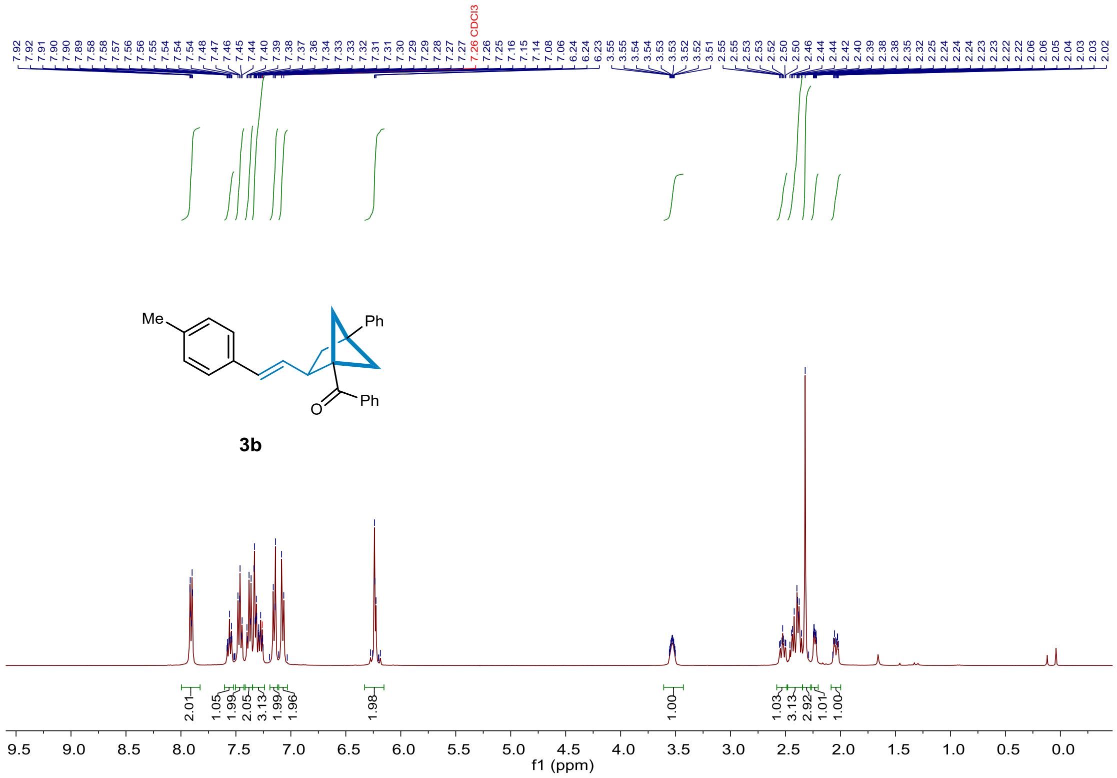

Supplementary Figure 18. **^{1}H NMR of the 3b** (400 MHz, $CDCl_3$)

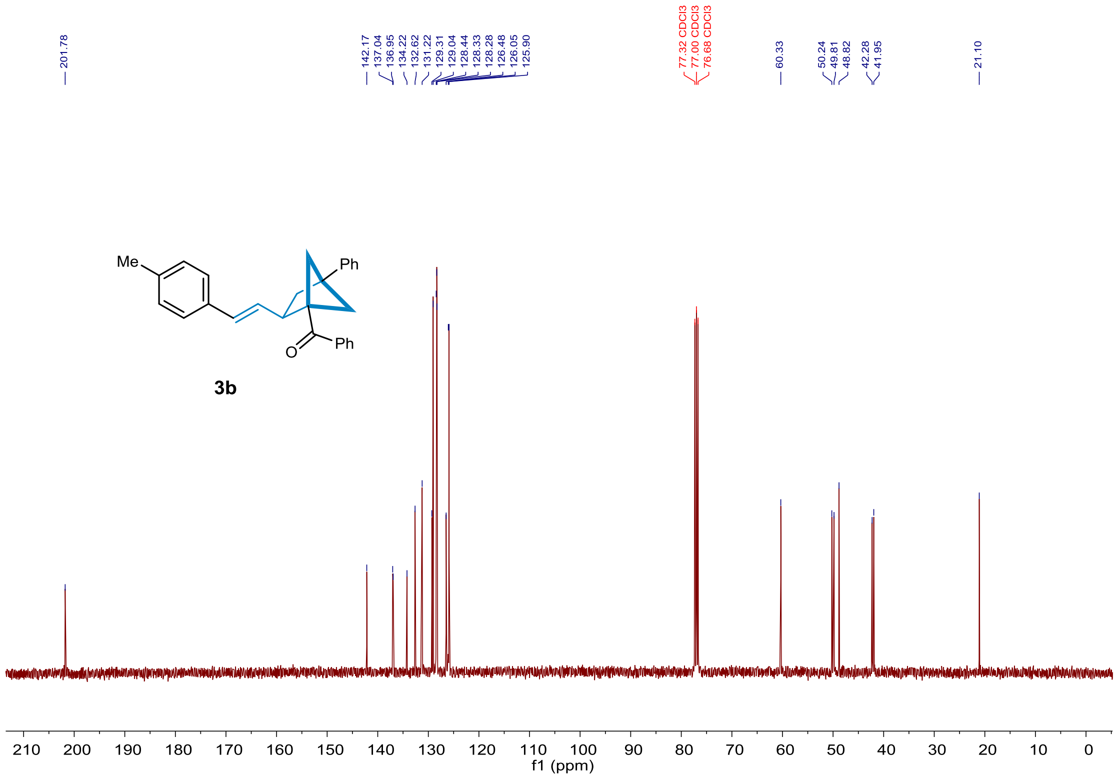

Supplementary Figure 19. **^{13}C NMR of the 3b** (101 MHz, $CDCl_3$)

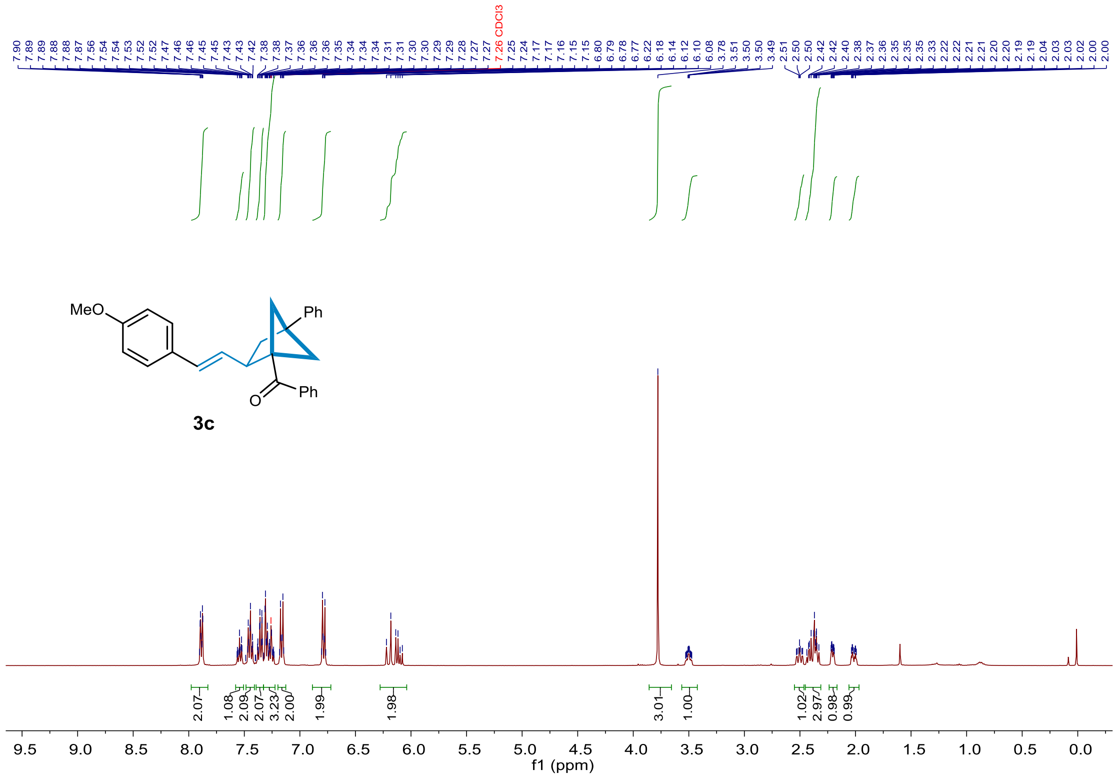

Supplementary Figure 20. ^{1}**H NMR of the 3c** (400 MHz, $CDCl_3$)

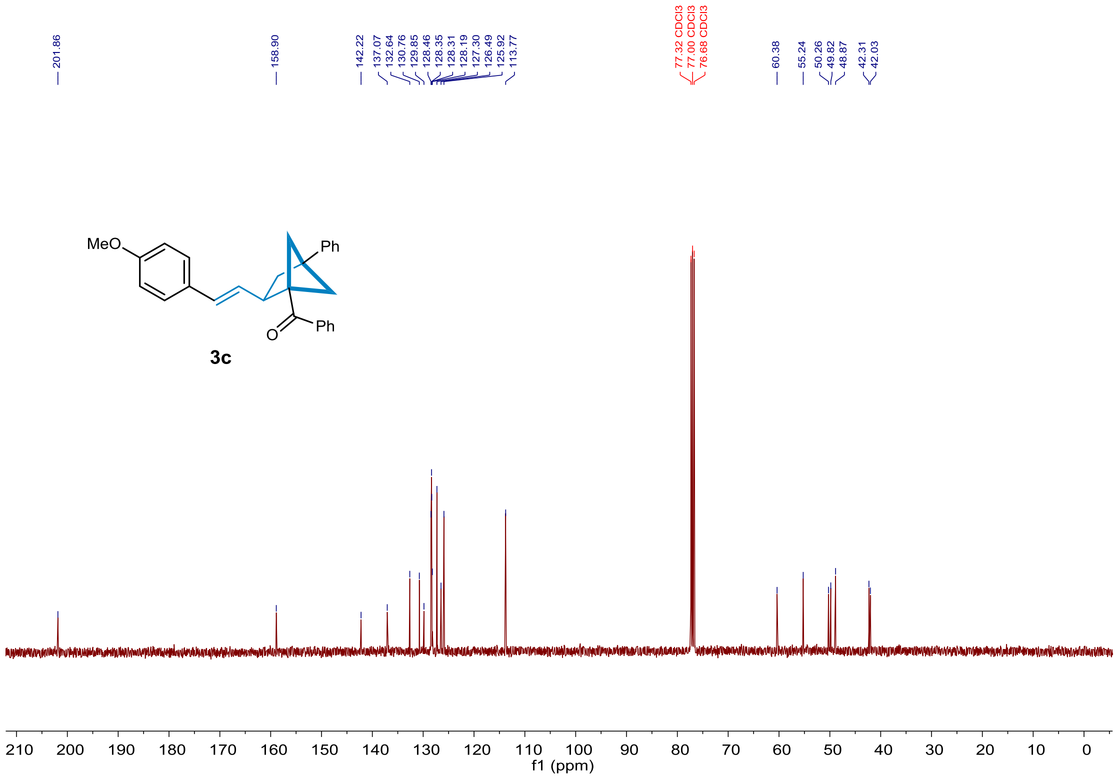

Supplementary Figure 21. ^{13}**C NMR of the 3c** (101 MHz, $CDCl_3$)

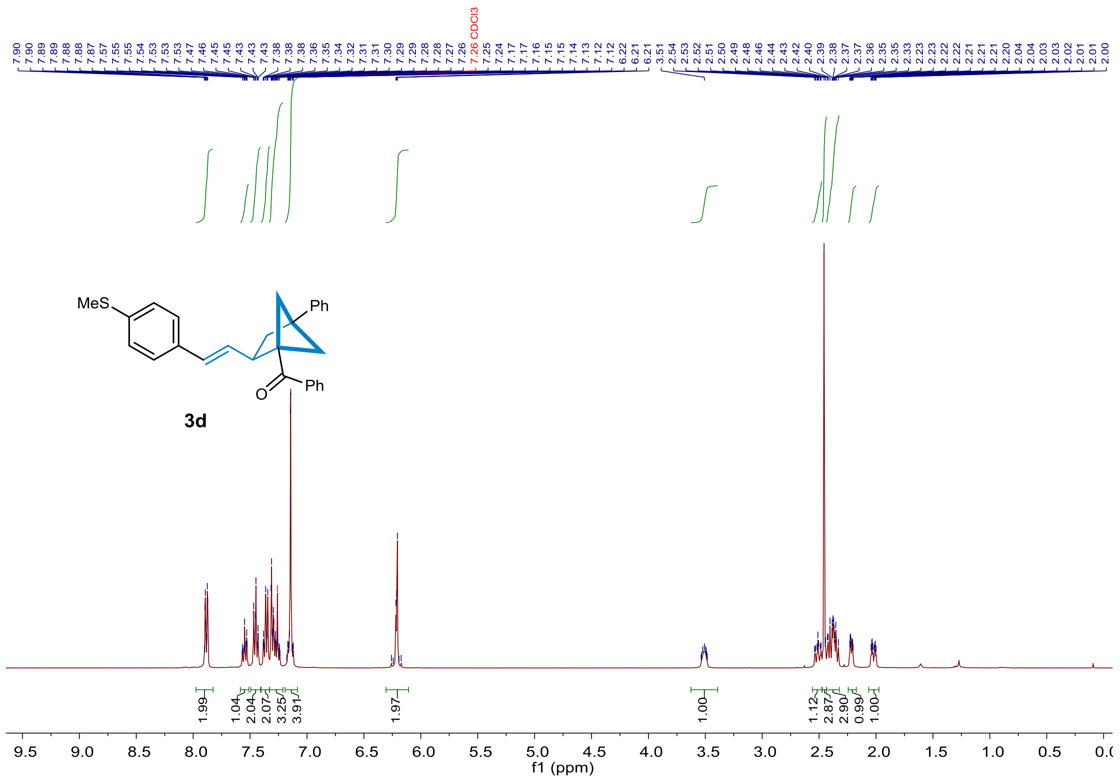

Supplementary Figure 22. **^{1}H NMR of the 3d** (400 MHz, $CDCl_3$)

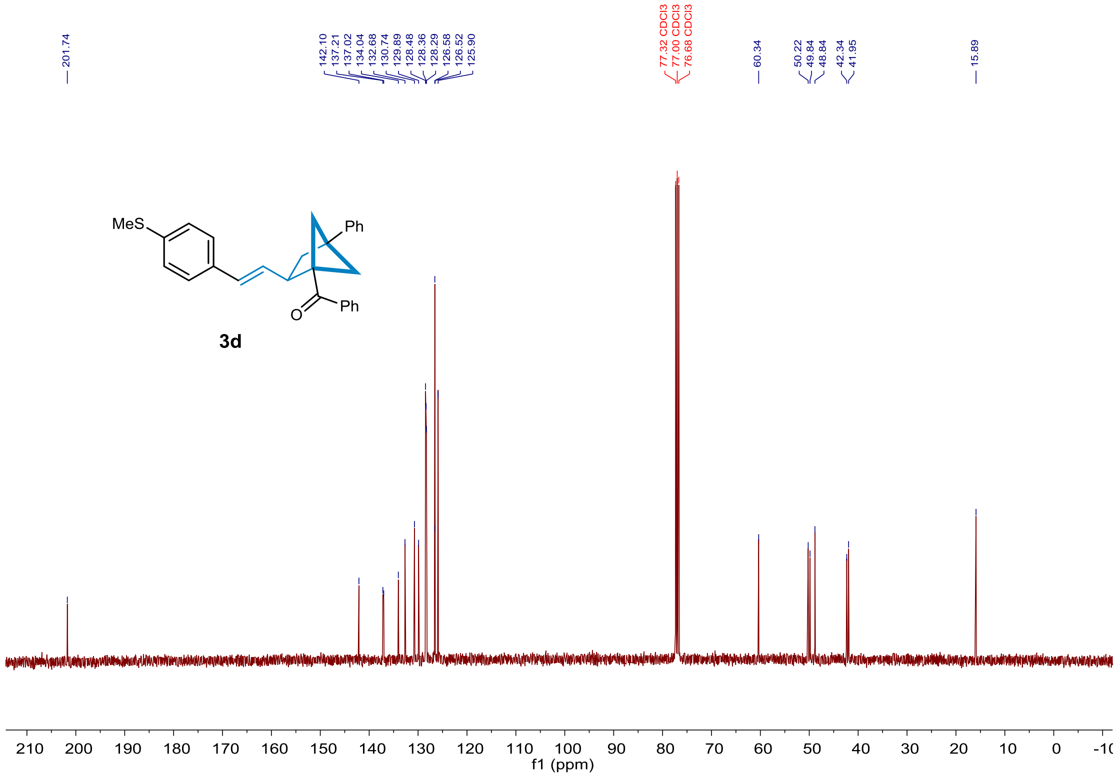

Supplementary Figure 23. **^{13}C NMR of the 3d** (101 MHz, $CDCl_3$)

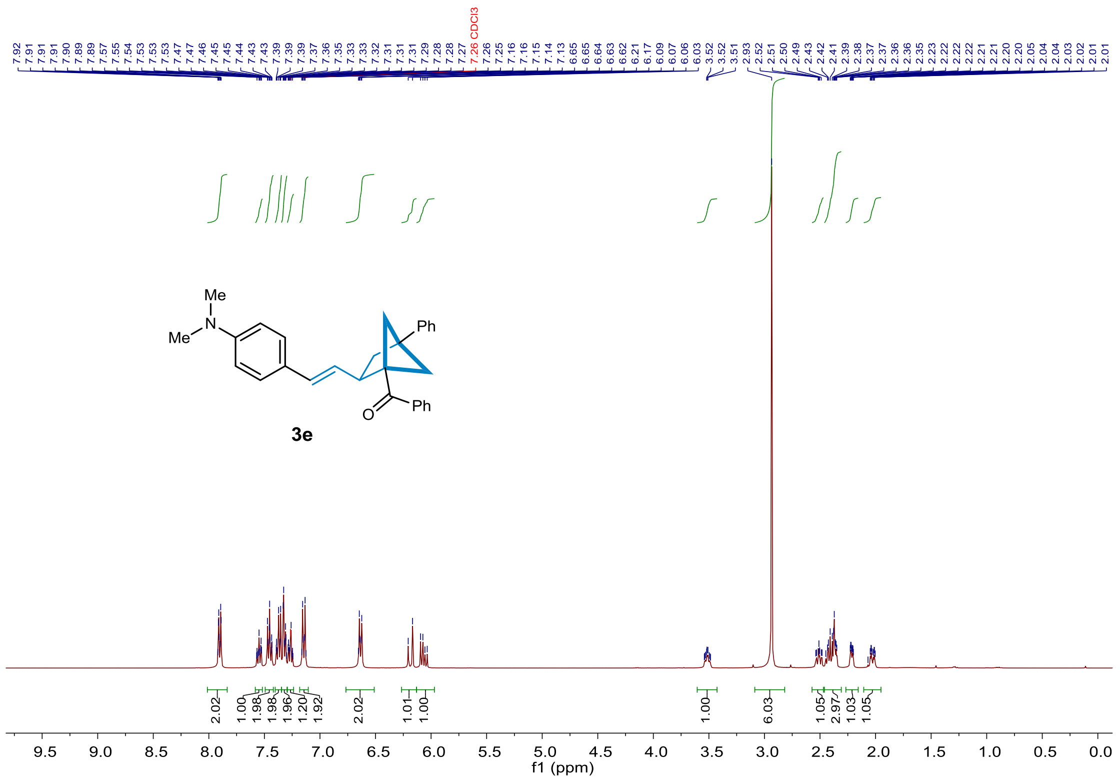

Supplementary Figure 24. **¹H NMR of the 3e** (400 MHz, $CDCl_3$)

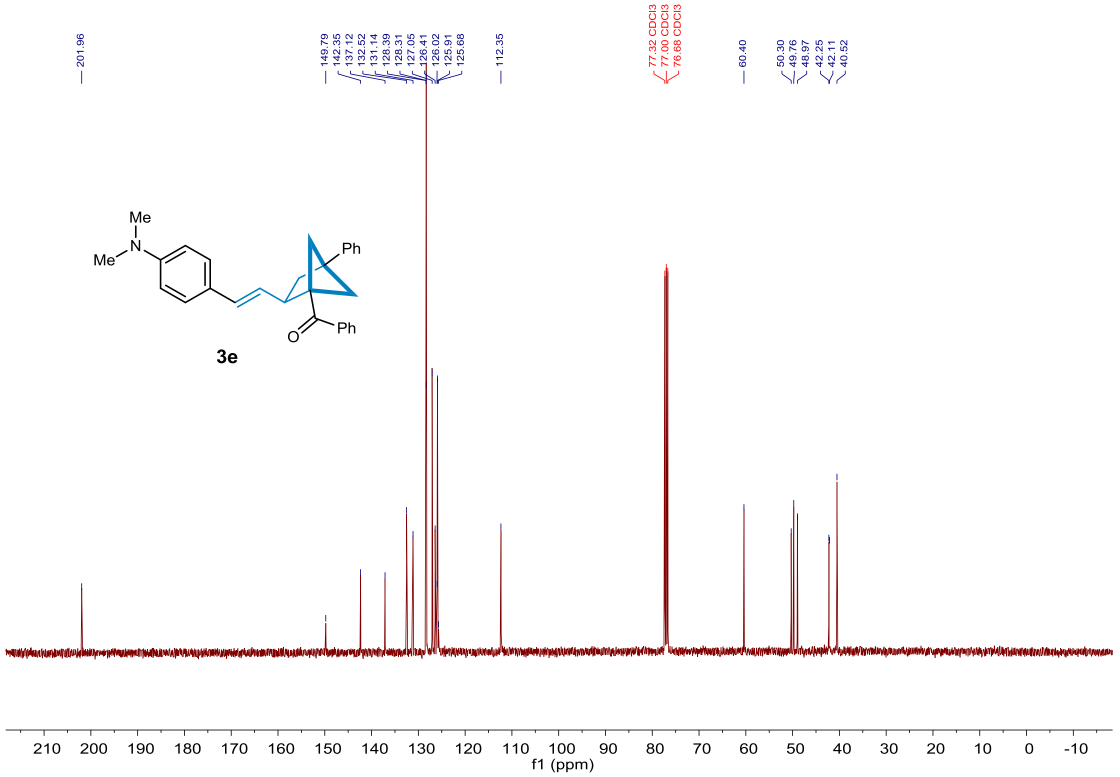

Supplementary Figure 25. **¹³C NMR of the 3e** (101 MHz, $CDCl_3$)

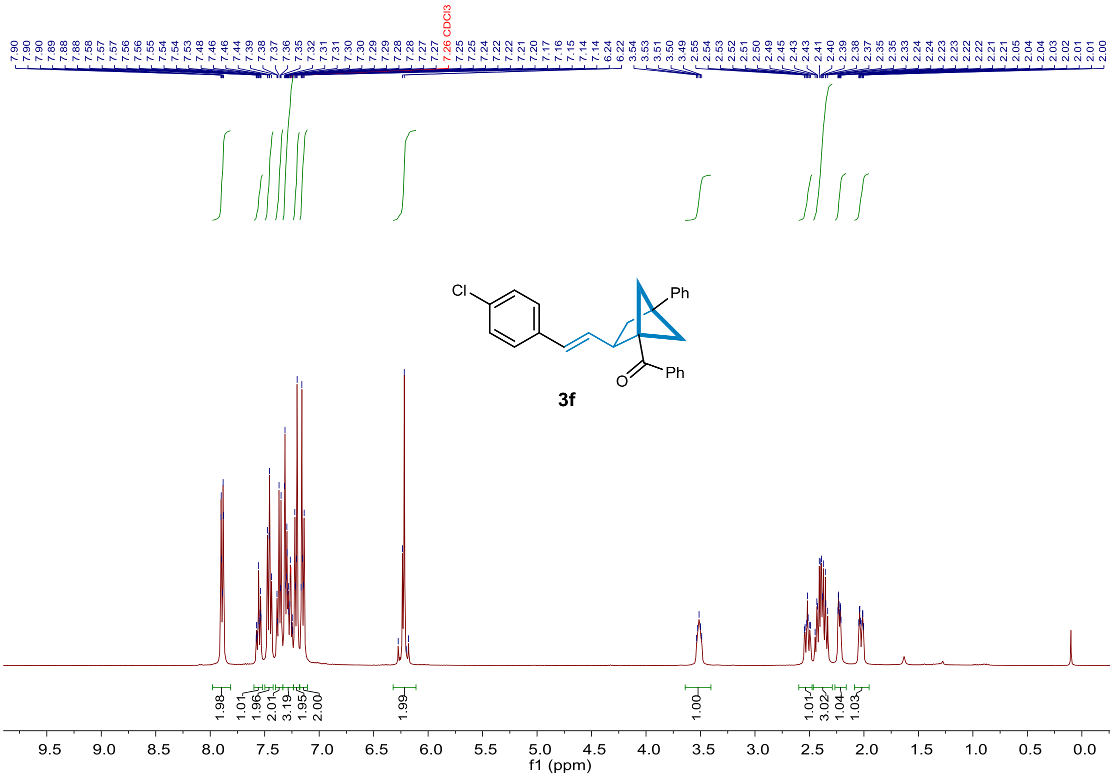

Supplementary Figure 26. ^{1}**H NMR of the 3f** (400 MHz, $CDCl_3$)

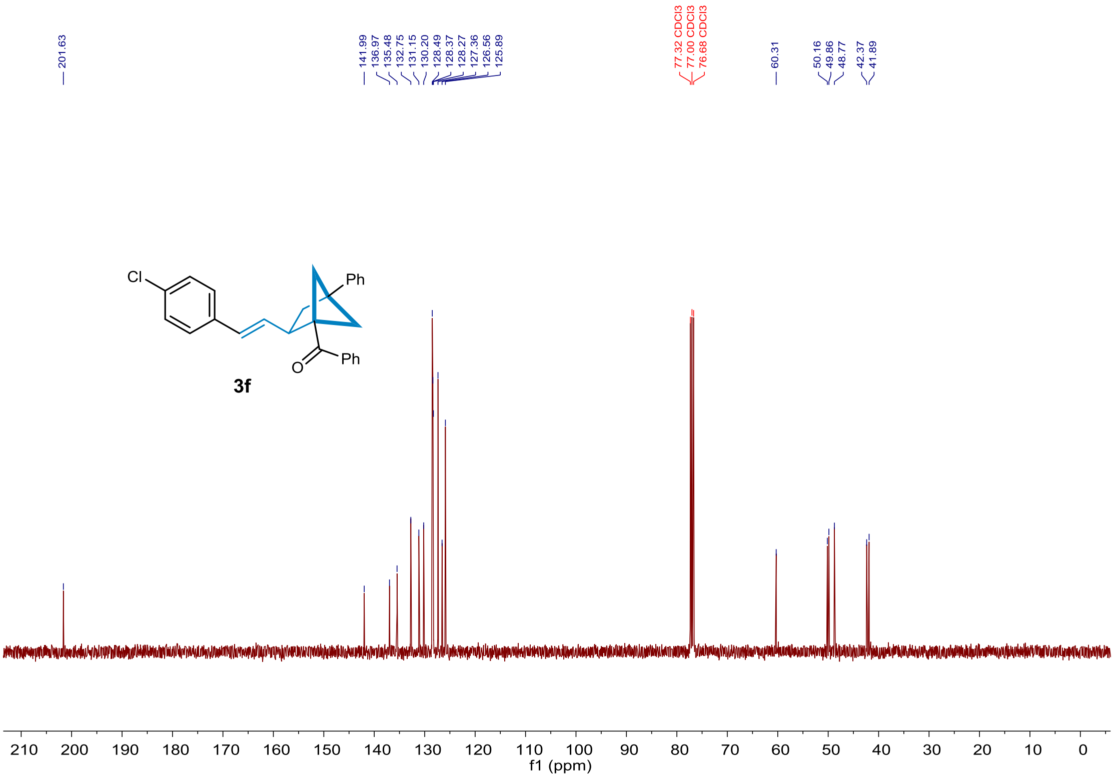

Supplementary Figure 27. ^{13}**C NMR of the 3f** (101 MHz, $CDCl_3$)

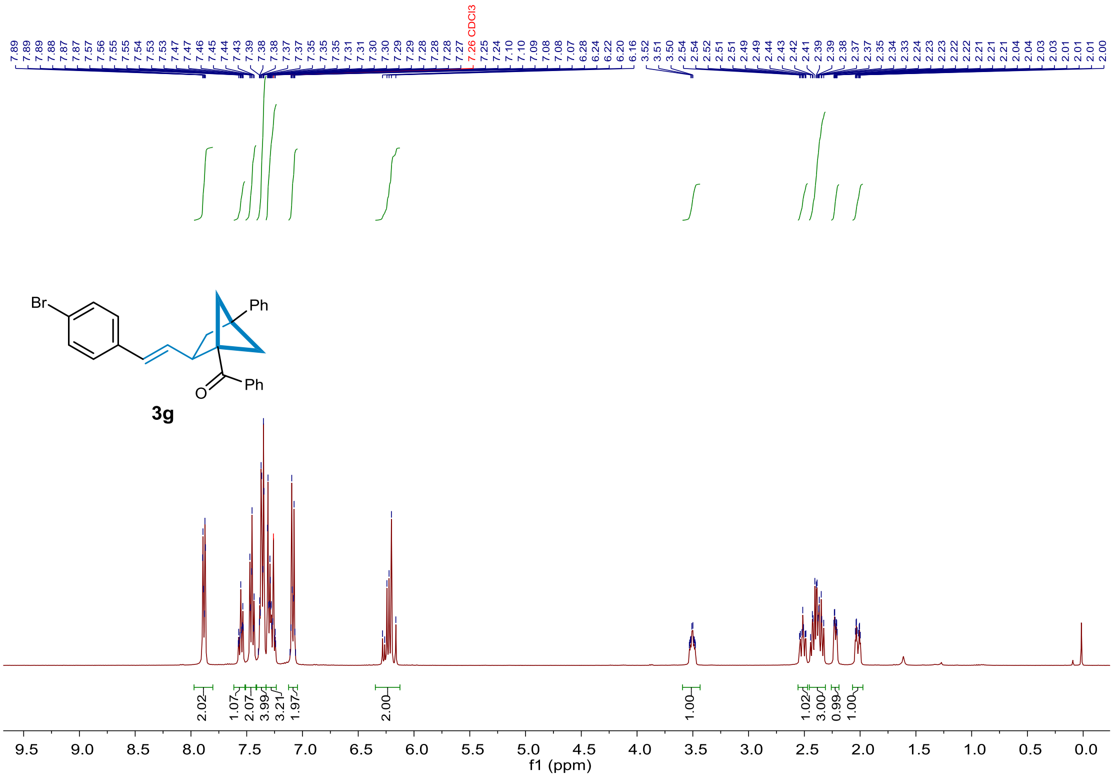

Supplementary Figure 28. **^{1}H NMR of the 3g** (400 MHz, $CDCl_3$)

^{13}C NMR (101 MHz, $CDCl_3$)

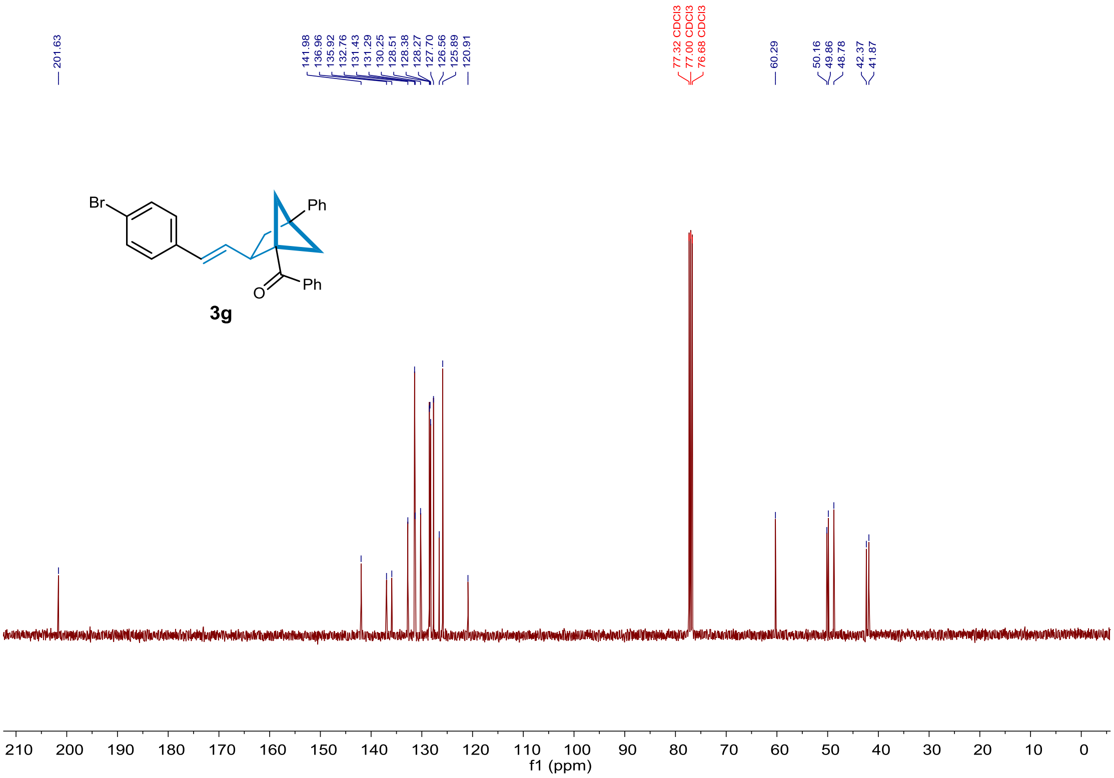

Supplementary Figure 29. **^{13}C NMR of the 3g** (101 MHz, $CDCl_3$)

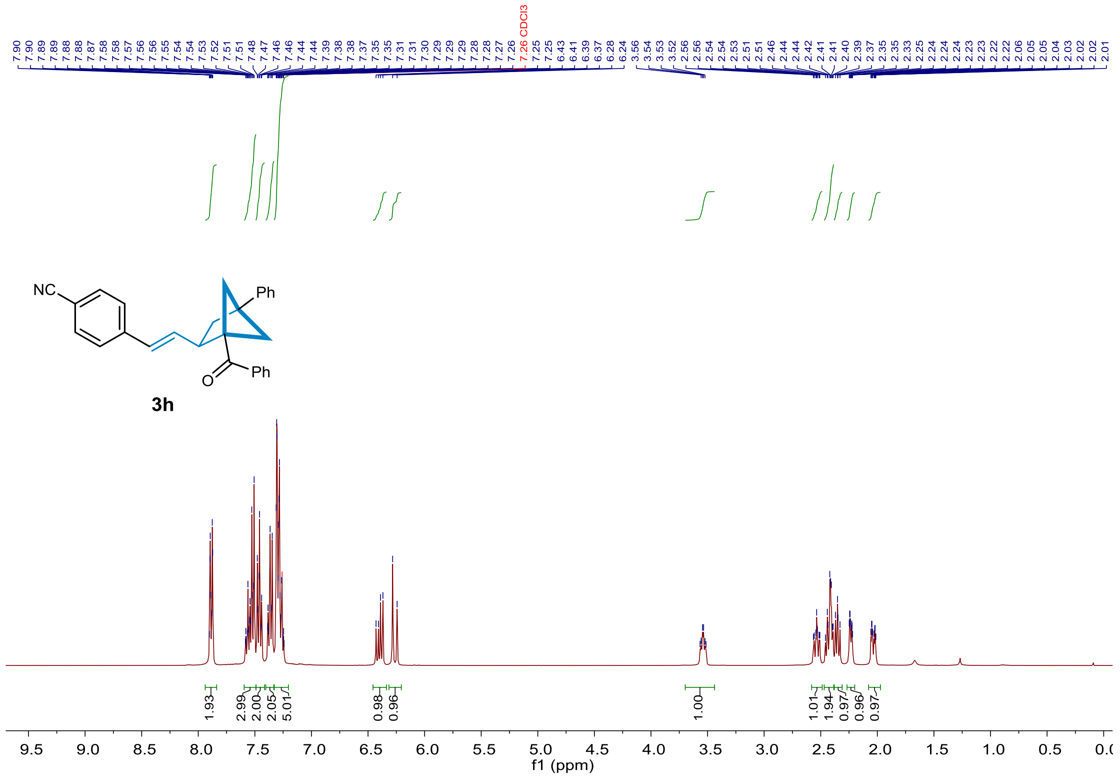

Supplementary Figure 30. **^{1}H NMR of the 3h** (400 MHz, $CDCl_3$)

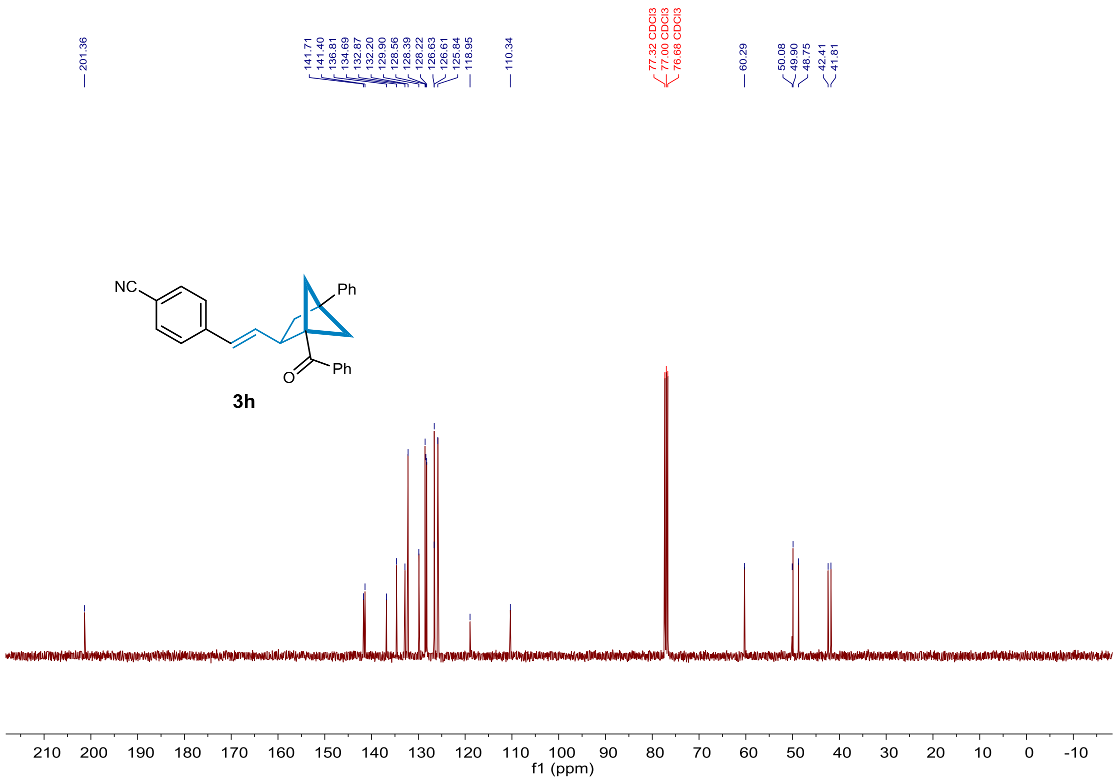

Supplementary Figure 31. **^{13}C NMR of the 3h** (101 MHz, $CDCl_3$)

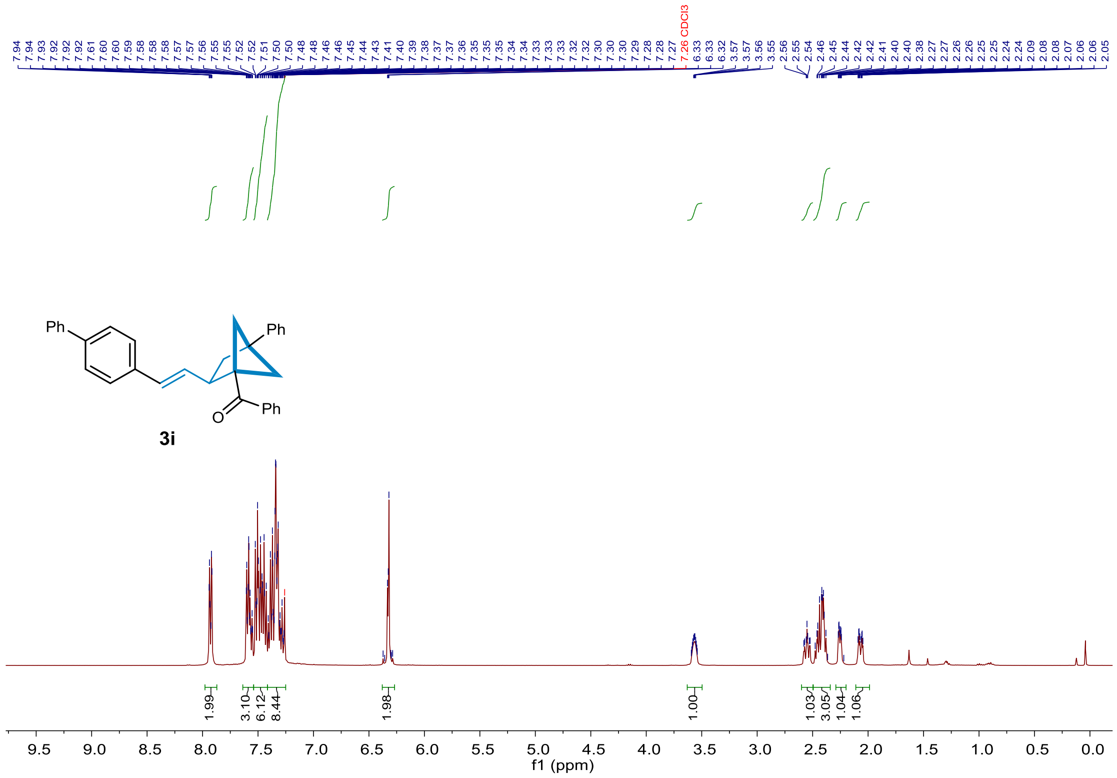

Supplementary Figure 32. **^{1}H NMR of the 3i** (400 MHz, $CDCl_3$)

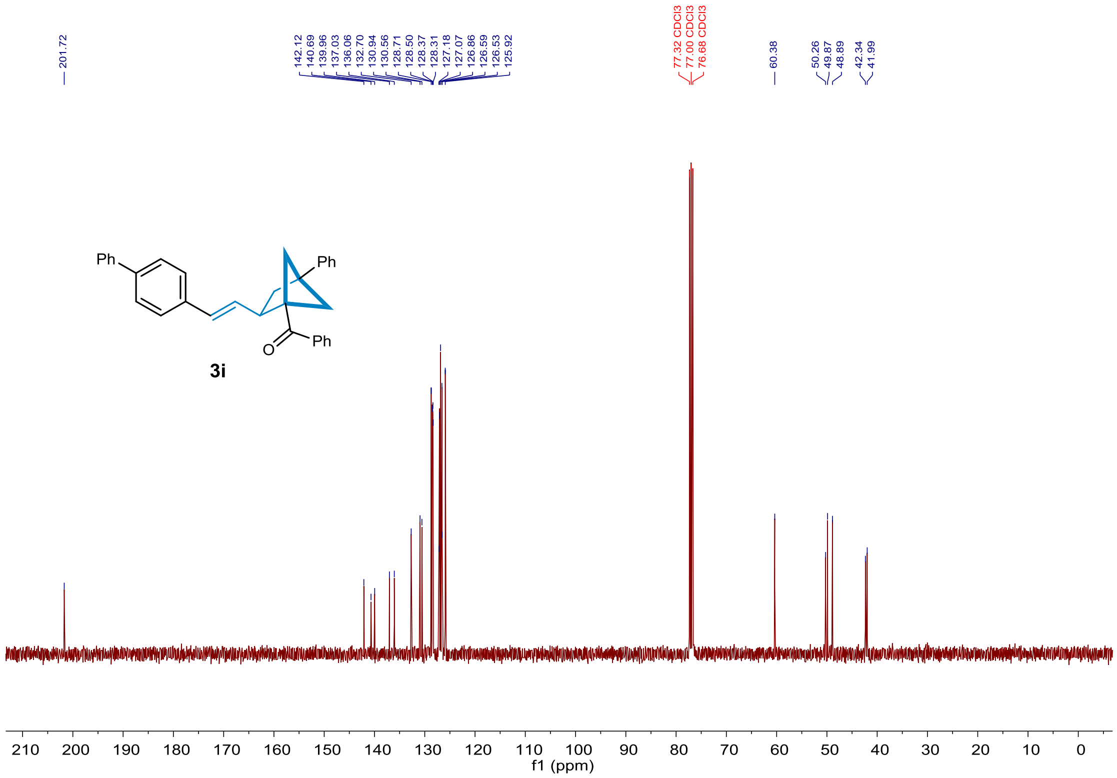

Supplementary Figure 33. **^{13}C NMR of the 3i** (101 MHz, $CDCl_3$)

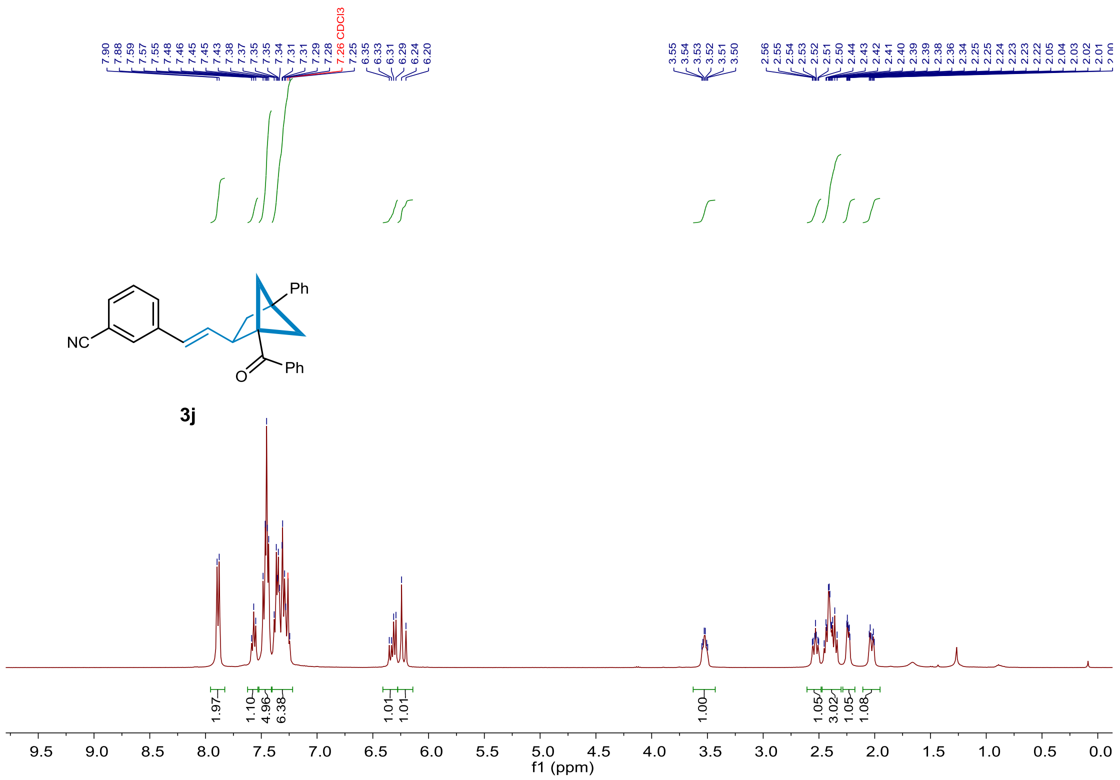

Supplementary Figure 34. ^{1}**H NMR of the 3j** (400 MHz, $CDCl_3$)

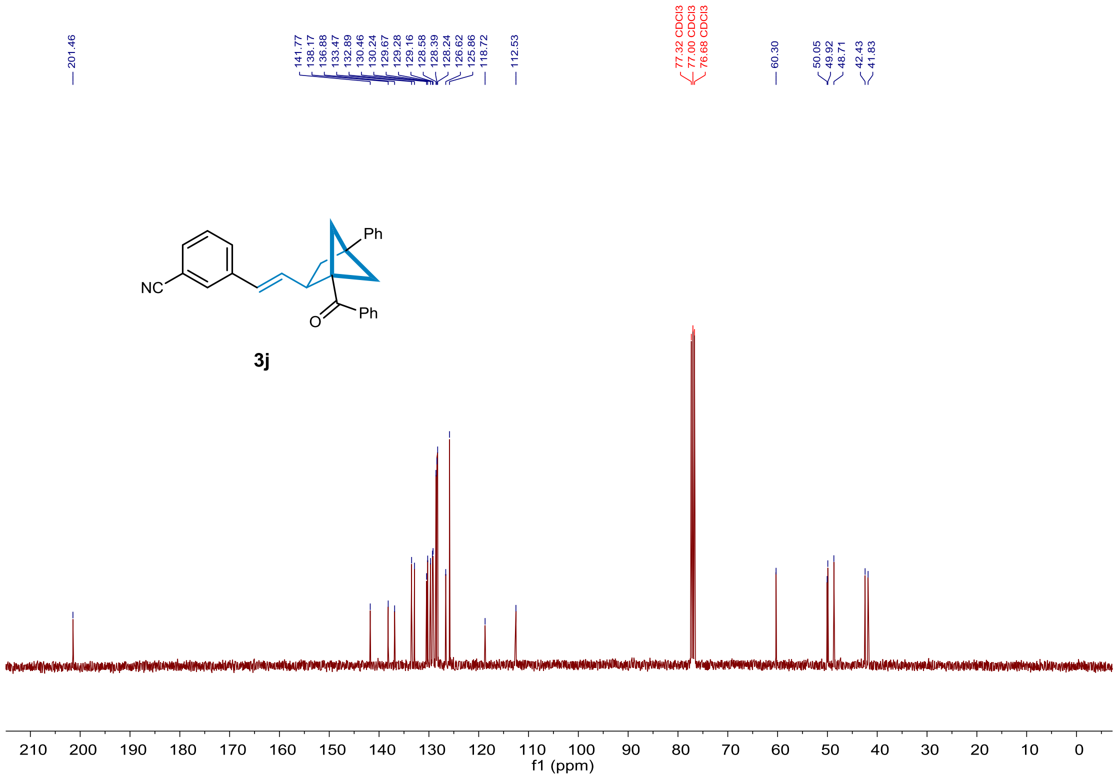

Supplementary Figure 35. ^{13}**C NMR of the 3j** (101 MHz, $CDCl_3$)

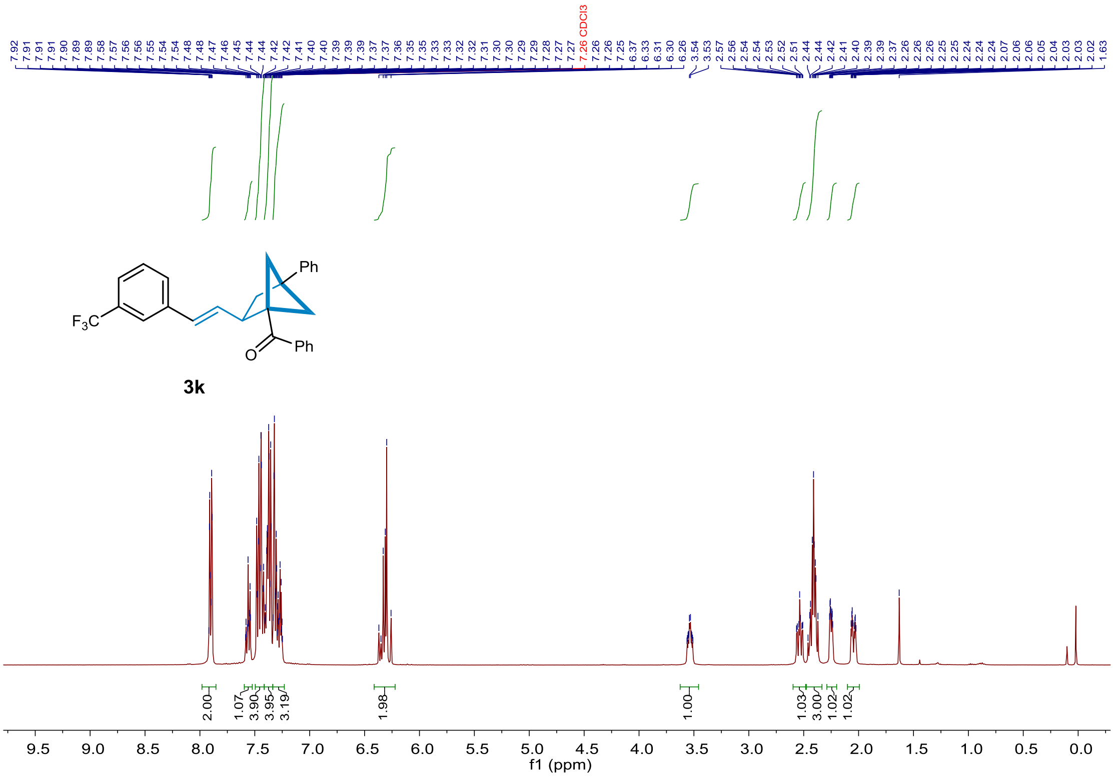

Supplementary Figure 36. **^{1}H NMR of the 3k** (400 MHz, $CDCl_3$)

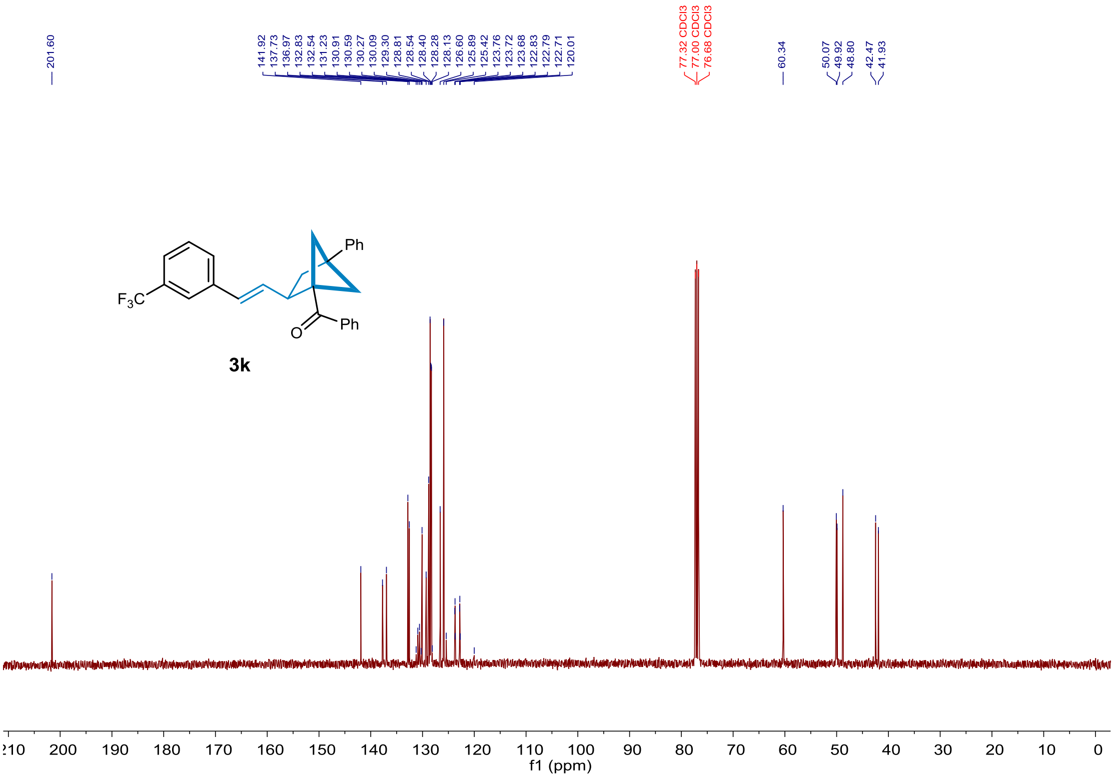

Supplementary Figure 37. **^{13}C NMR of the 3k** (101 MHz, $CDCl_3$)

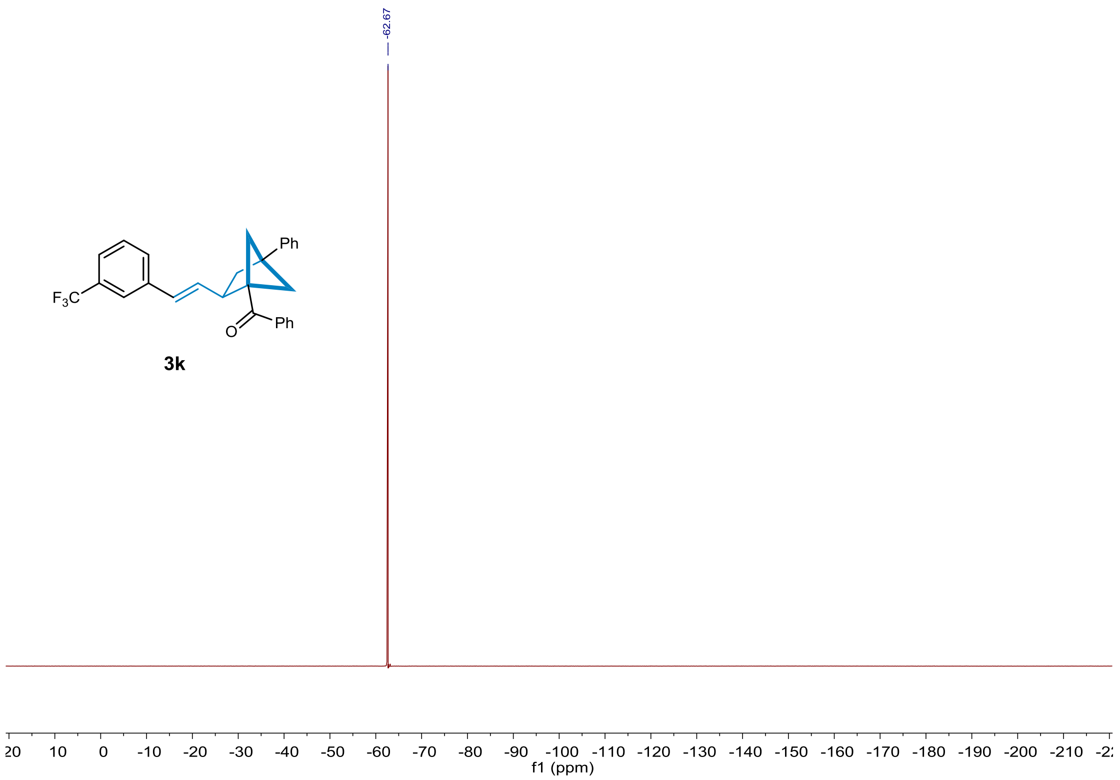

Supplementary Figure 38. ^{19}F NMR of the 3k (377 MHz, $CDCl_3$)

^{1}H NMR (400 MHz, $CDCl_3$)

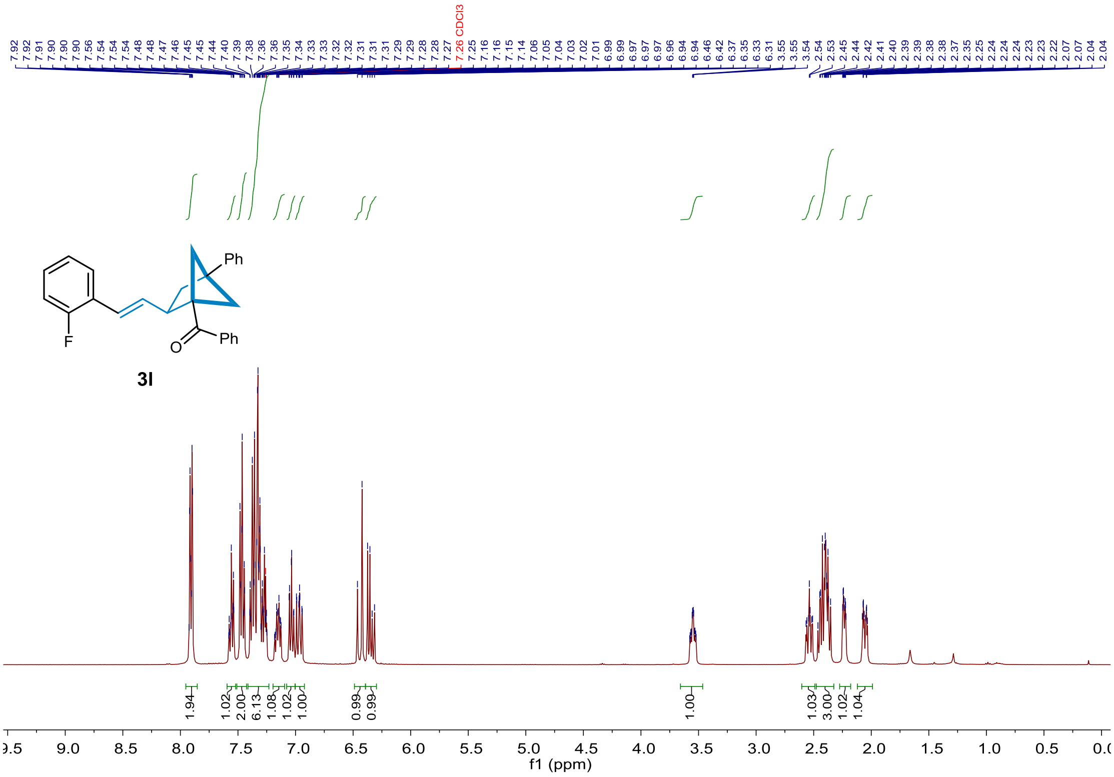

Supplementary Figure 39. ^{1}H NMR of the 3l (400 MHz, $CDCl_3$)

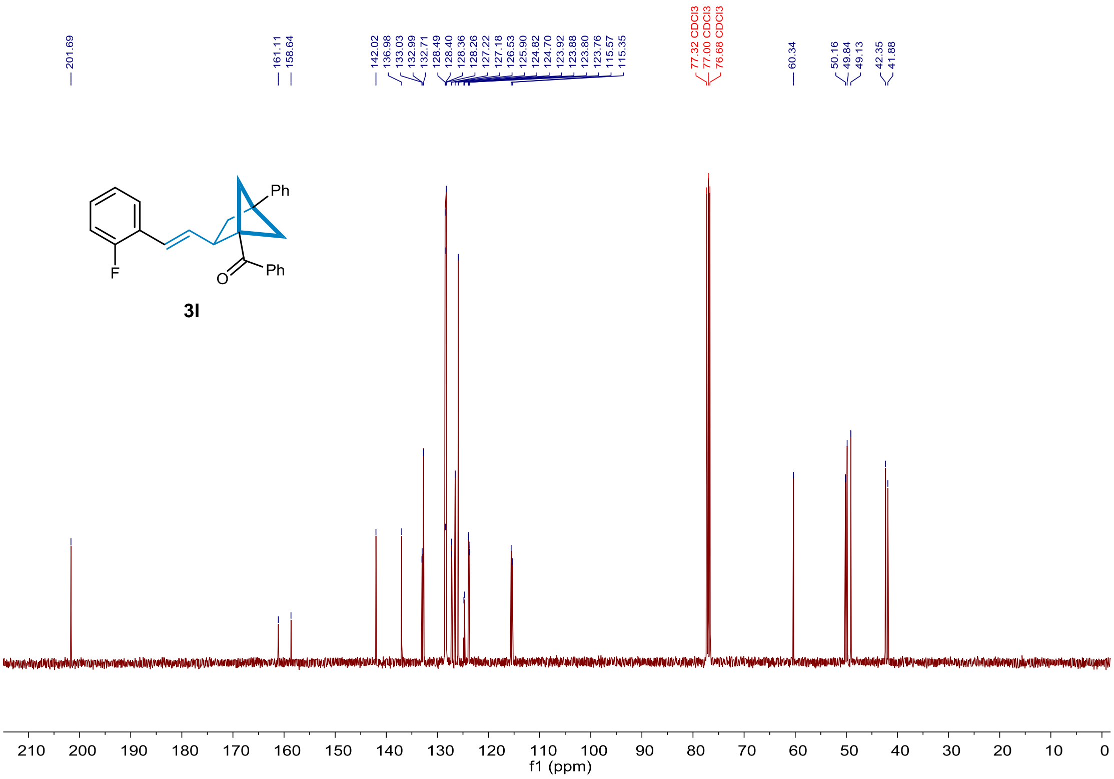

Supplementary Figure 40. ^{13}C NMR of the 3l (101 MHz, $CDCl_3$)

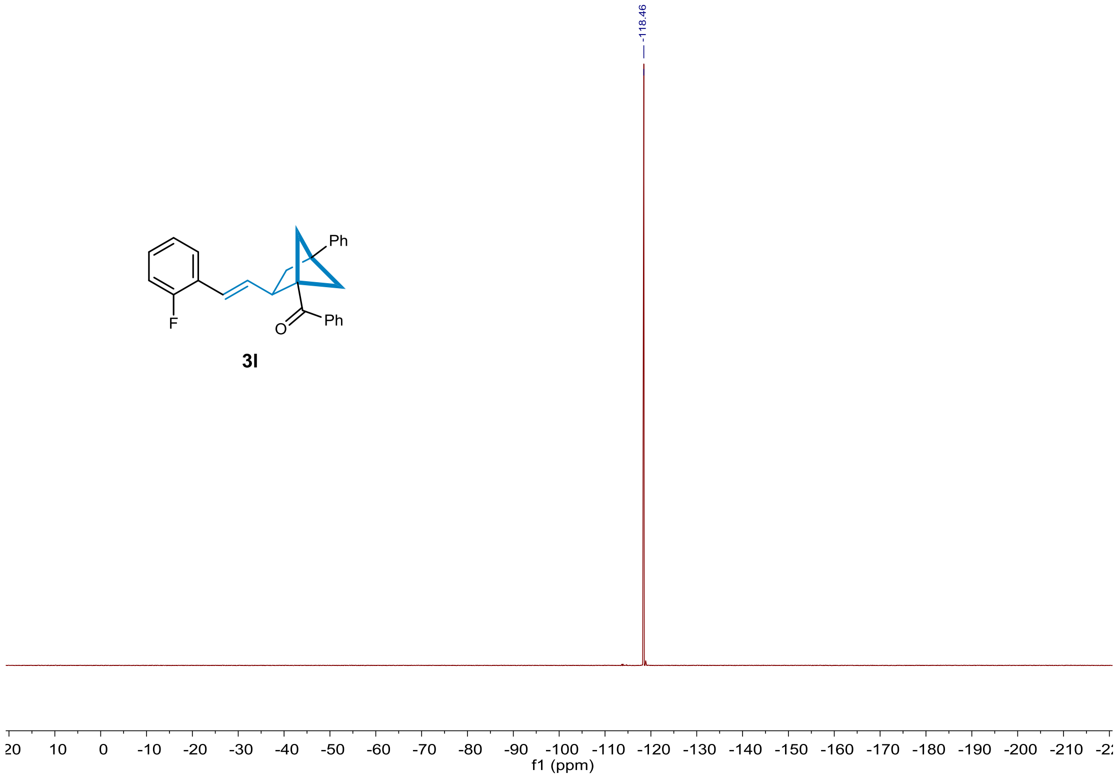

Supplementary Figure 41. **^{19}F NMR of the 3l** (377 MHz, $CDCl_3$)

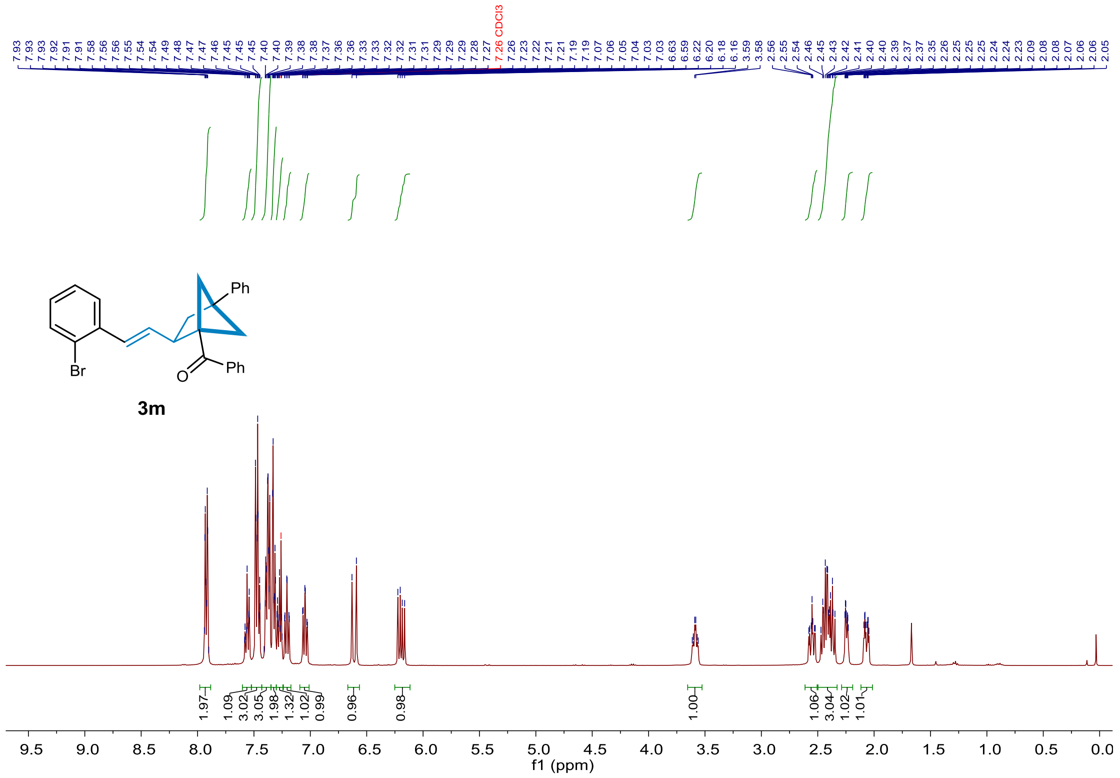

Supplementary Figure 42. **^{1}H NMR of the 3m** (400 MHz, $CDCl_3$)

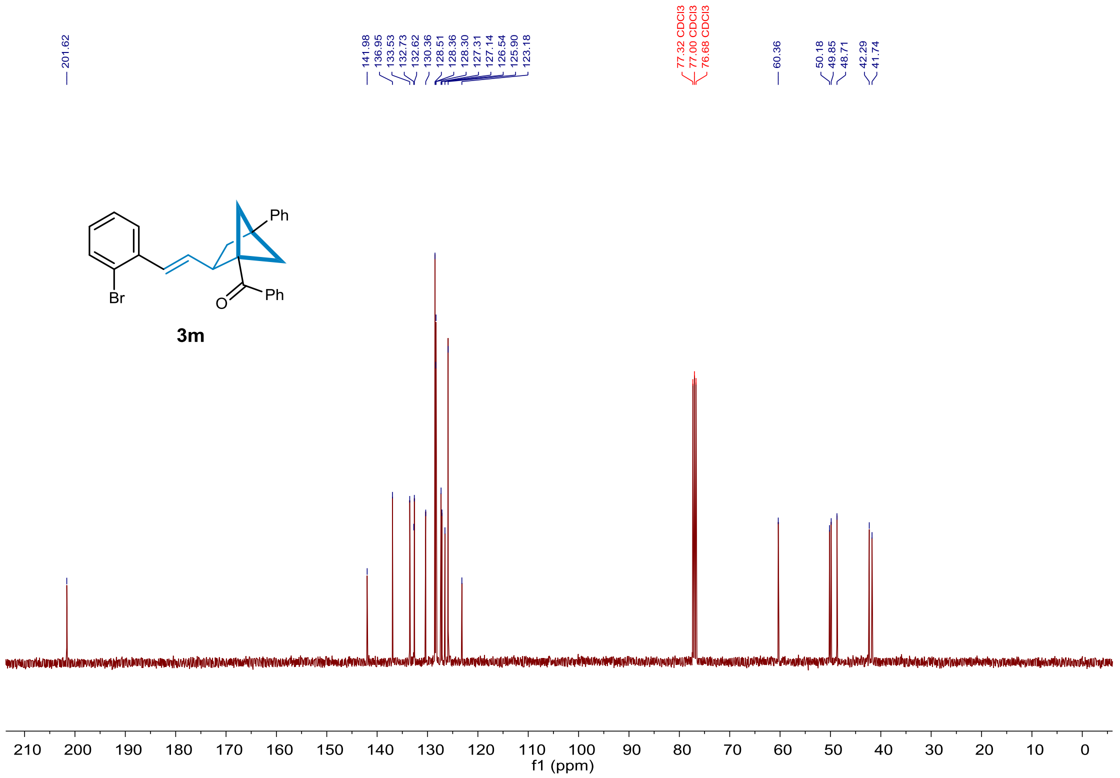

Supplementary Figure 43. **^{13}C NMR of the 3m** (101 MHz, $CDCl_3$)

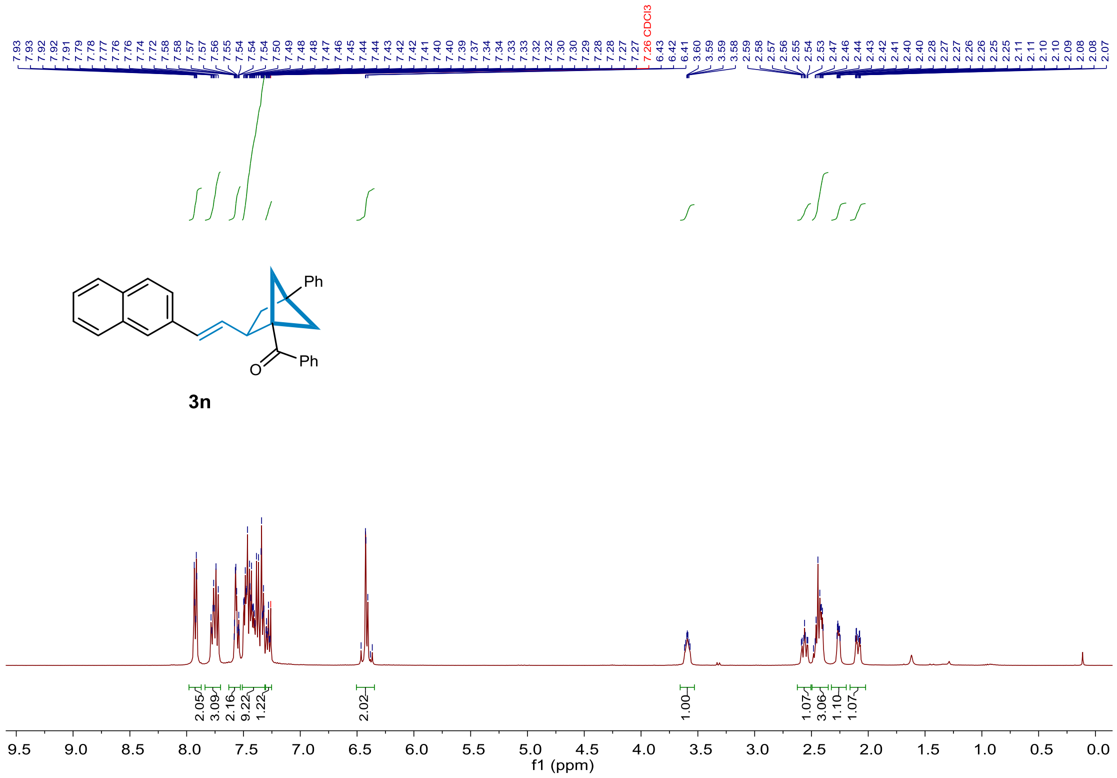

Supplementary Figure 44. **^{1}H NMR of the 3n** (400 MHz, $CDCl_3$)

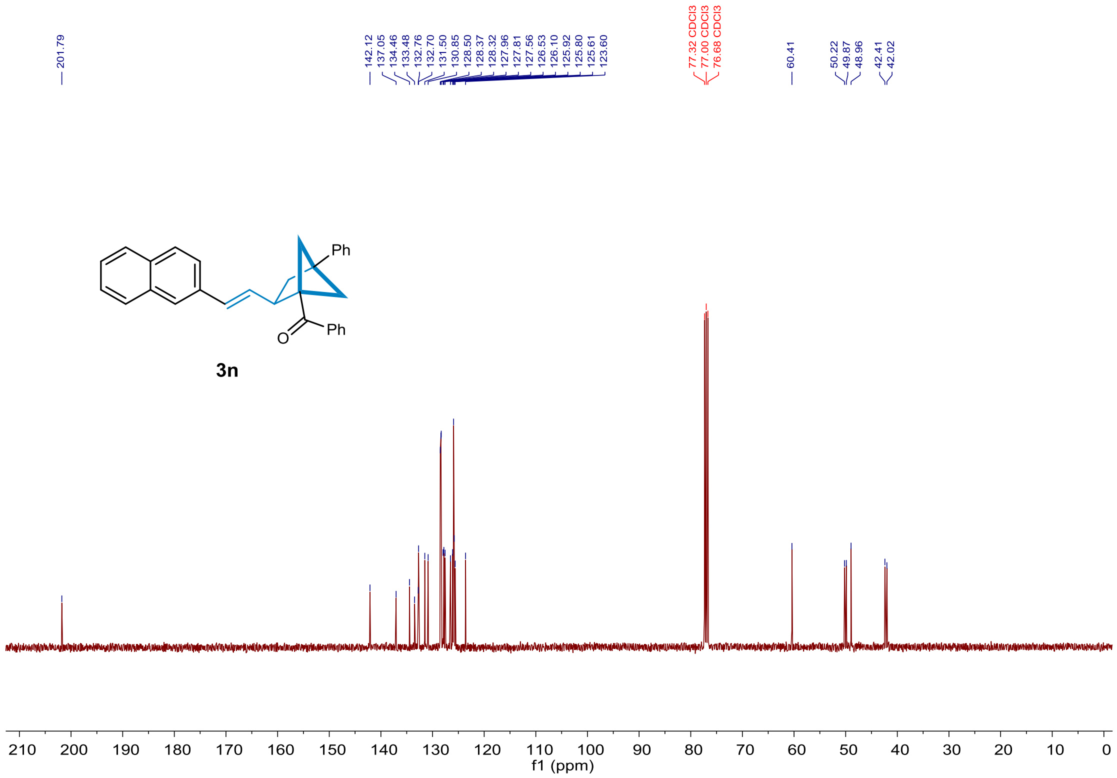

Supplementary Figure 45. **^{13}C NMR of the 3n** (101 MHz, $CDCl_3$)

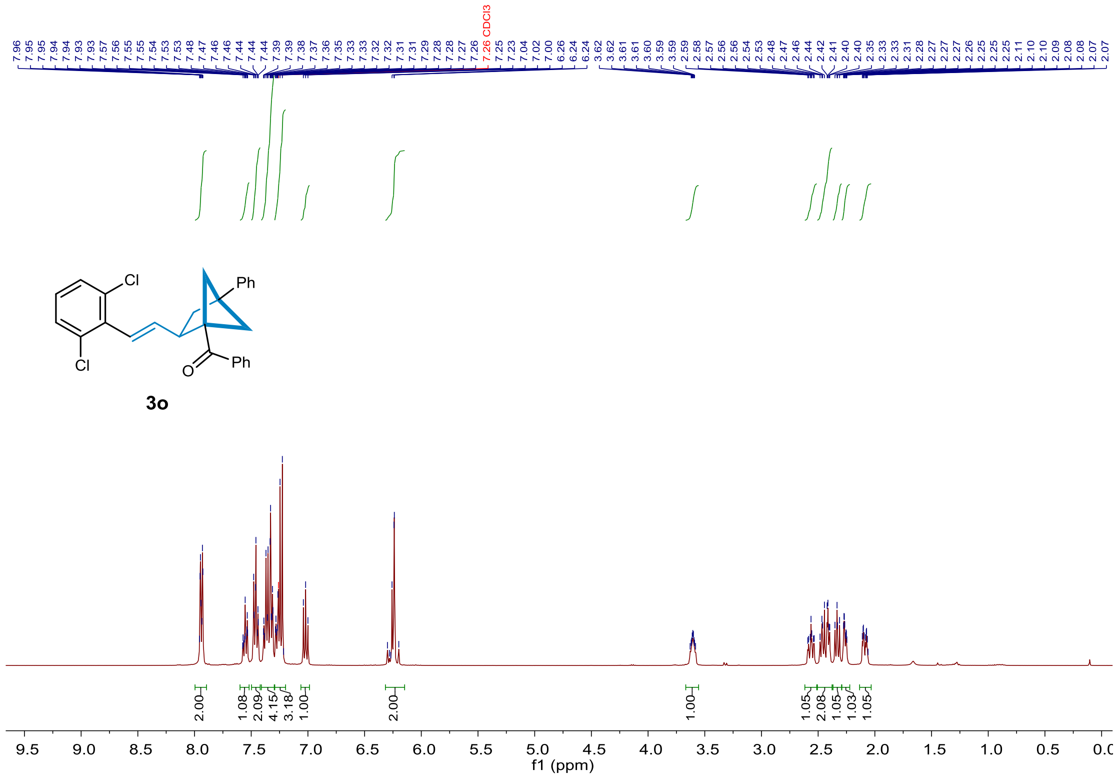

Supplementary Figure 46. **^{1}H NMR of the 3o** (400 MHz, $CDCl_3$)

^{13}C NMR (101 MHz, $CDCl_3$)

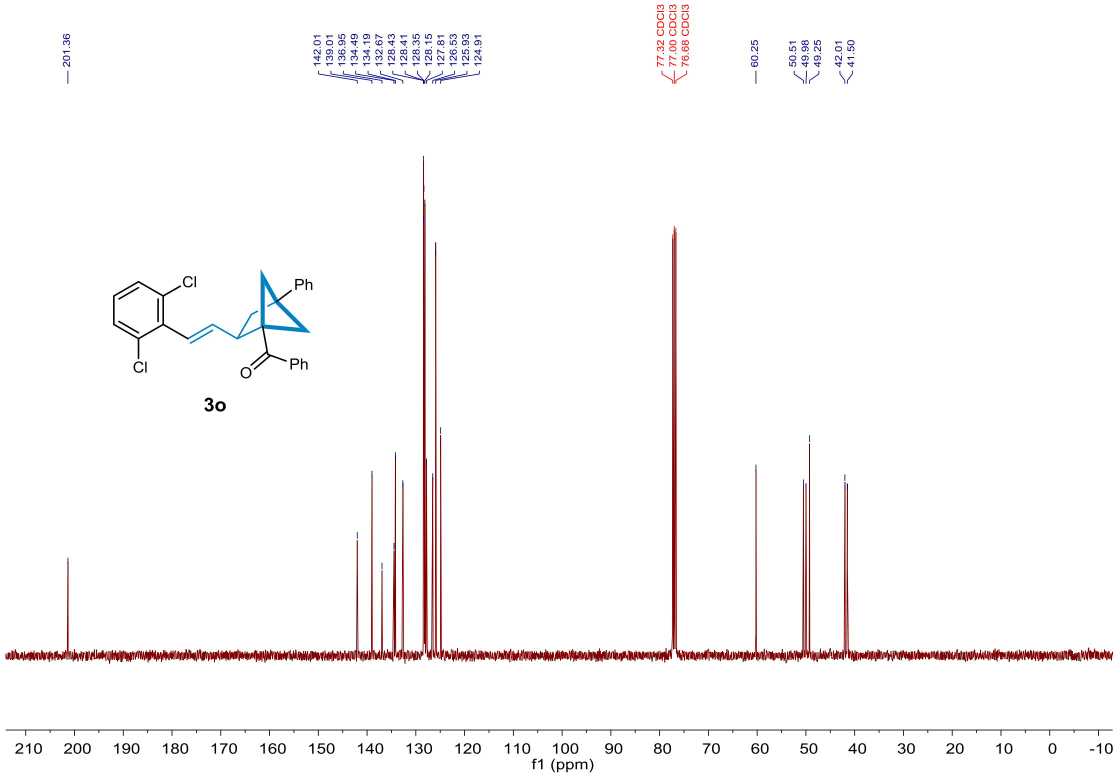

Supplementary Figure 47. **^{13}C NMR of the 3o** (101 MHz, $CDCl_3$)

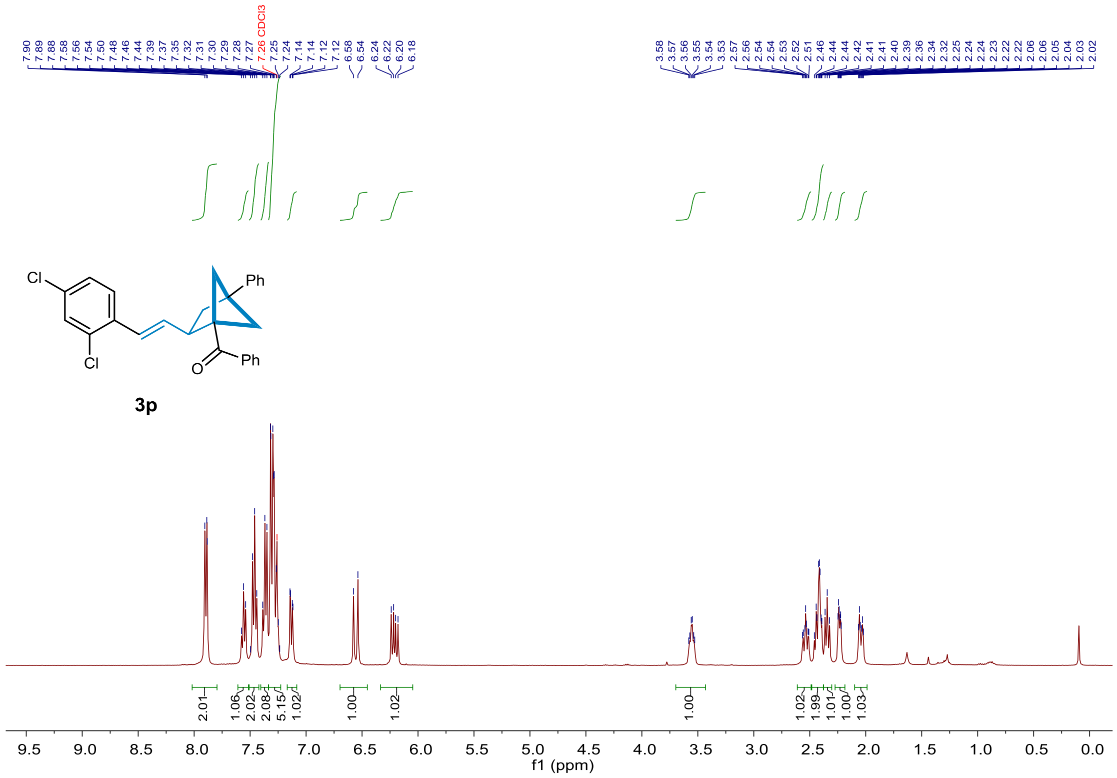

Supplementary Figure 48. **^{1}H NMR of the 3p** (400 MHz, $CDCl_3$)

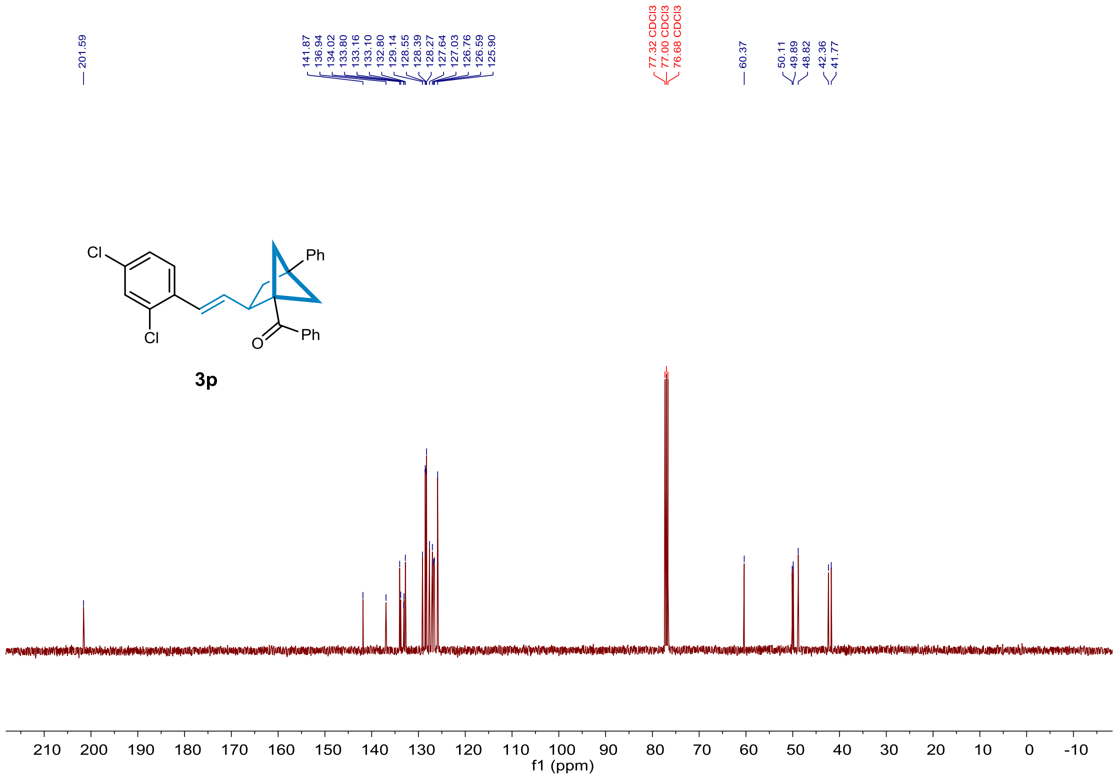

Supplementary Figure 49. **^{13}C NMR of the 3p** (101 MHz, $CDCl_3$)

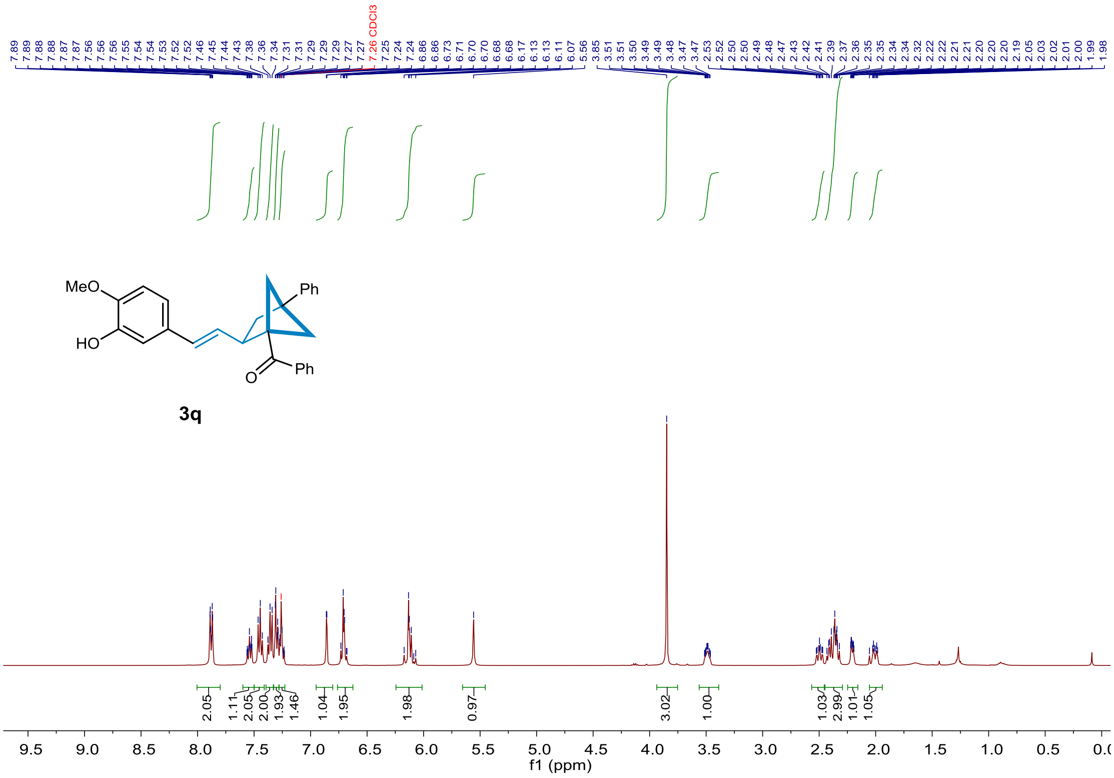

Supplementary Figure 50. **^{1}H NMR of the 3q** (400 MHz, $CDCl_3$)

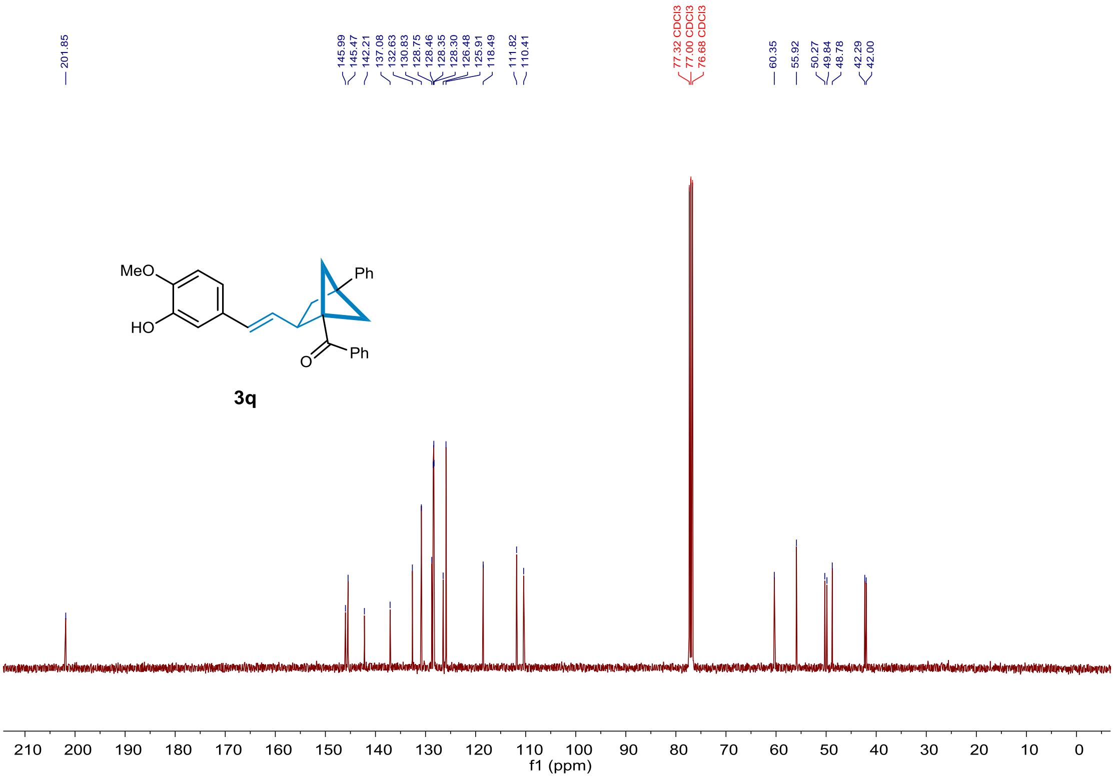

Supplementary Figure 51. **^{13}C NMR of the 3q** (101 MHz, $CDCl_3$)

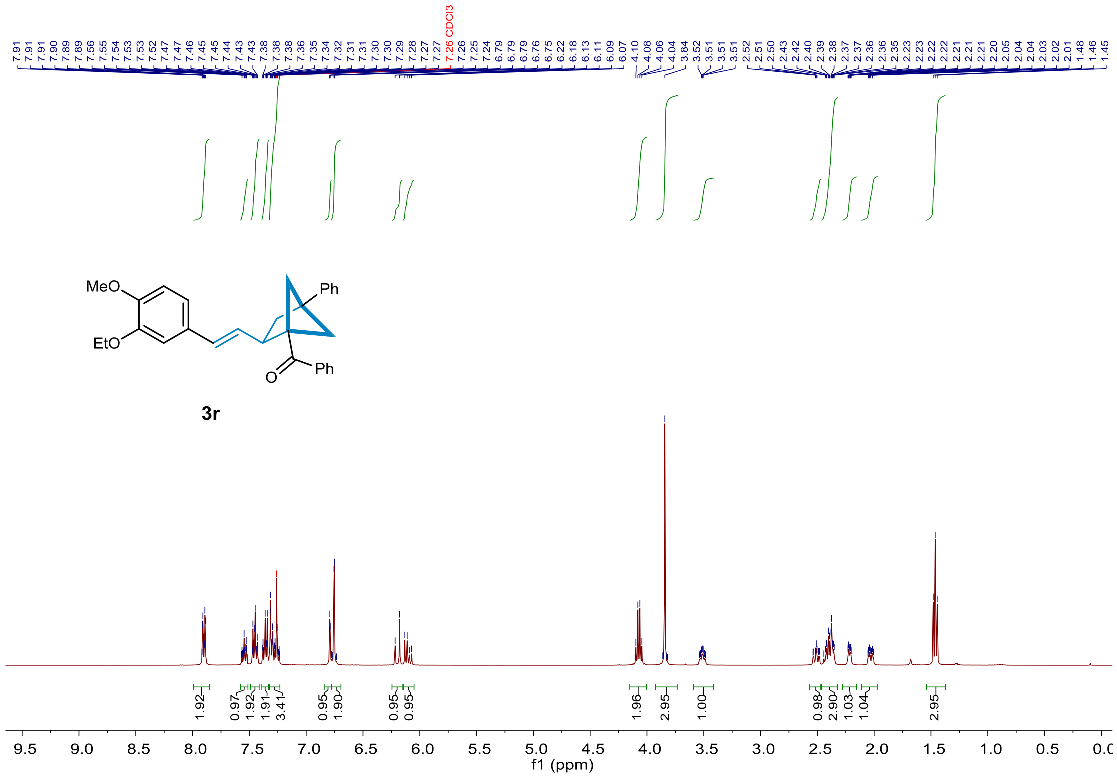

Supplementary Figure 52. **¹H NMR of the 3r** (400 MHz, $CDCl_3$)

¹³C NMR (101 MHz, $CDCl_3$)

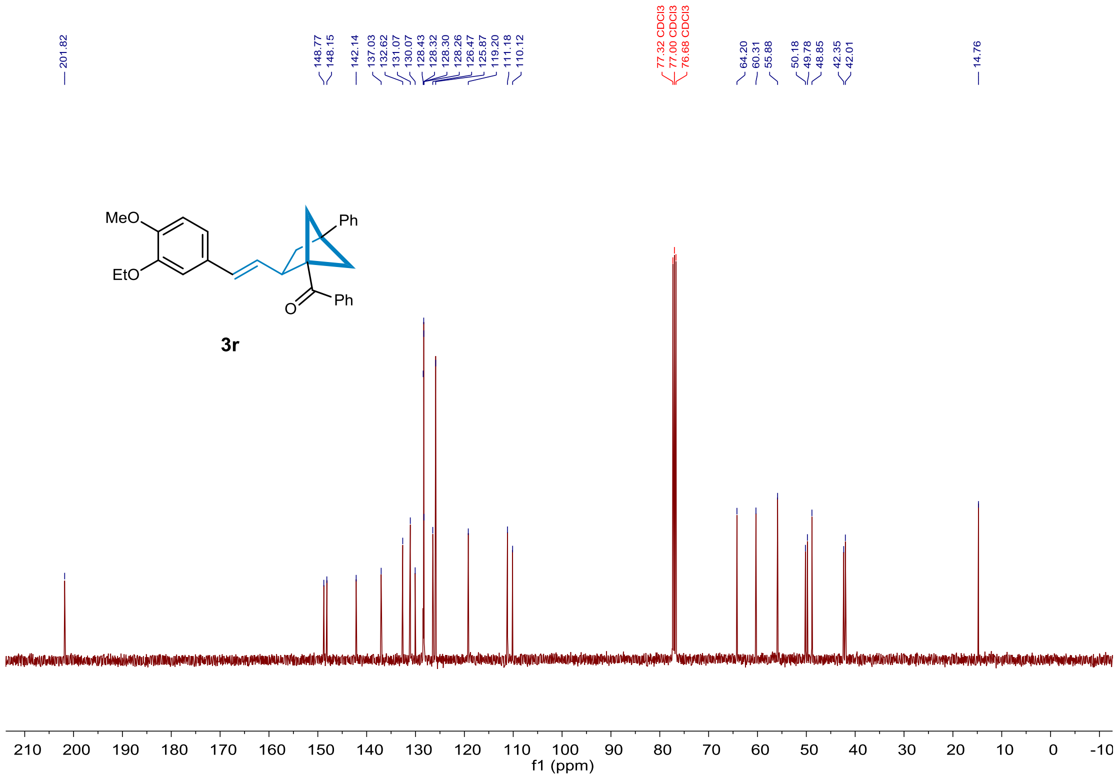

Supplementary Figure 53. **¹³C NMR of the 3r** (101 MHz, $CDCl_3$)

¹H NMR (400 MHz, $CDCl_3$)

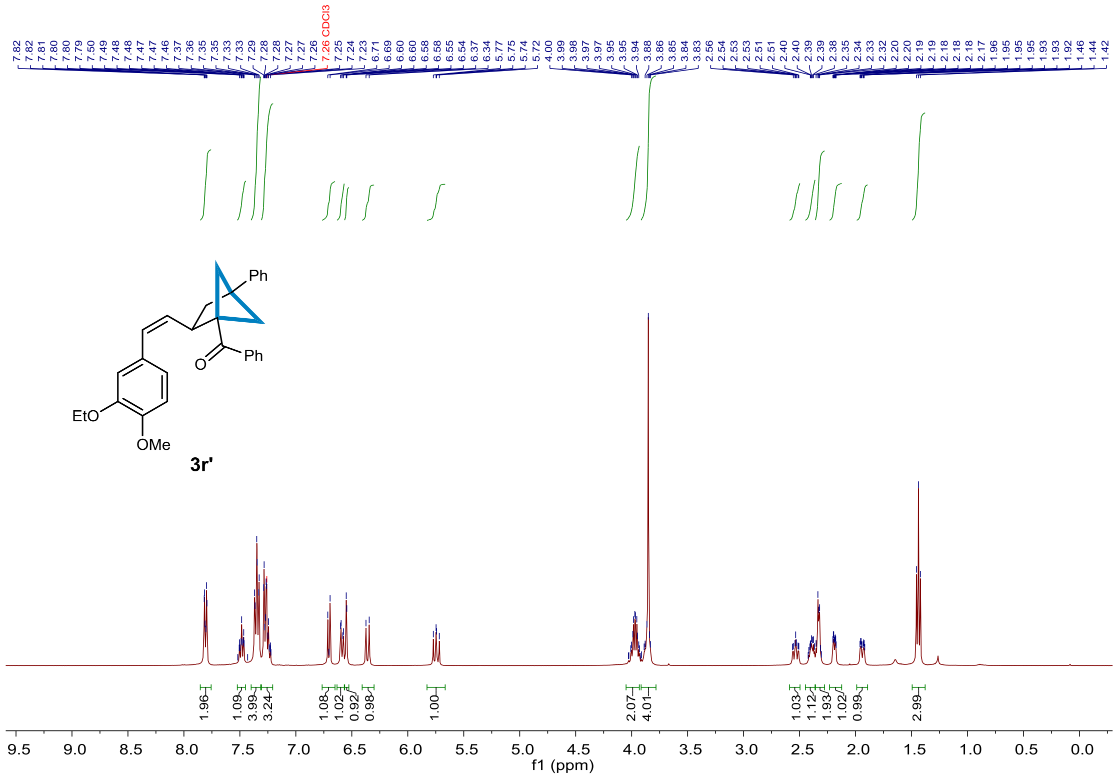

Supplementary Figure 54. **^{1}H NMR of the 3r'** (400 MHz, $CDCl_3$)

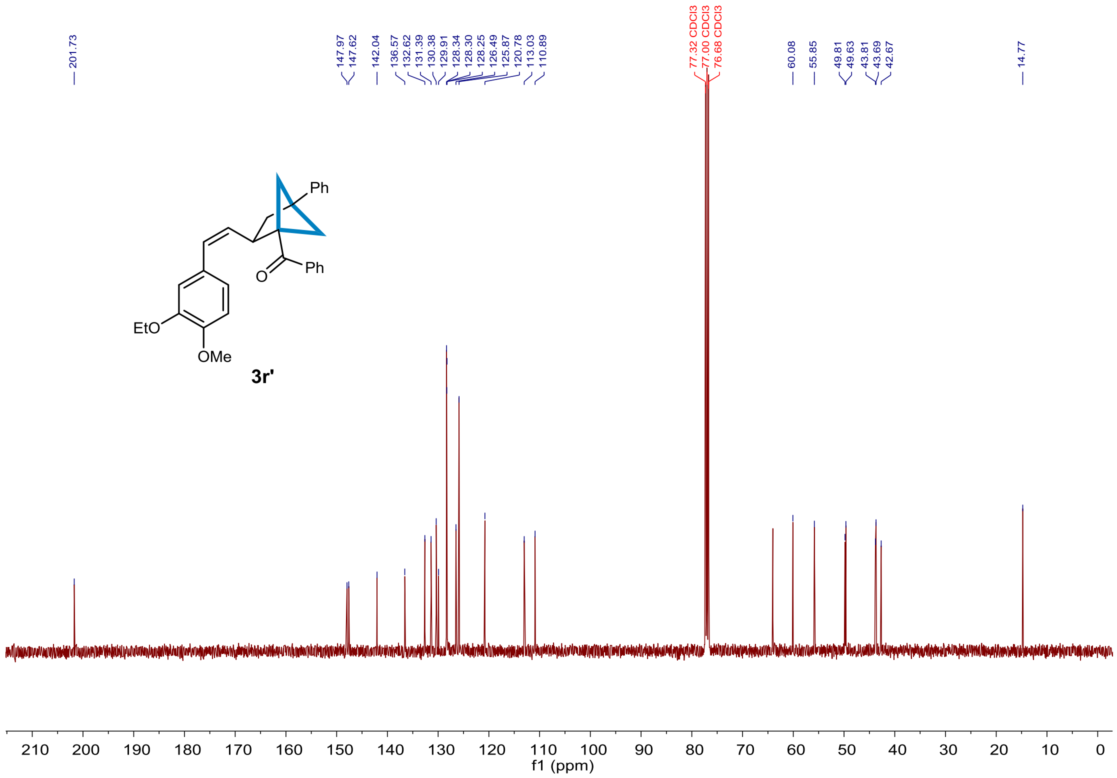

Supplementary Figure 55. **^{13}C NMR of the 3r'** (101 MHz, $CDCl_3$)

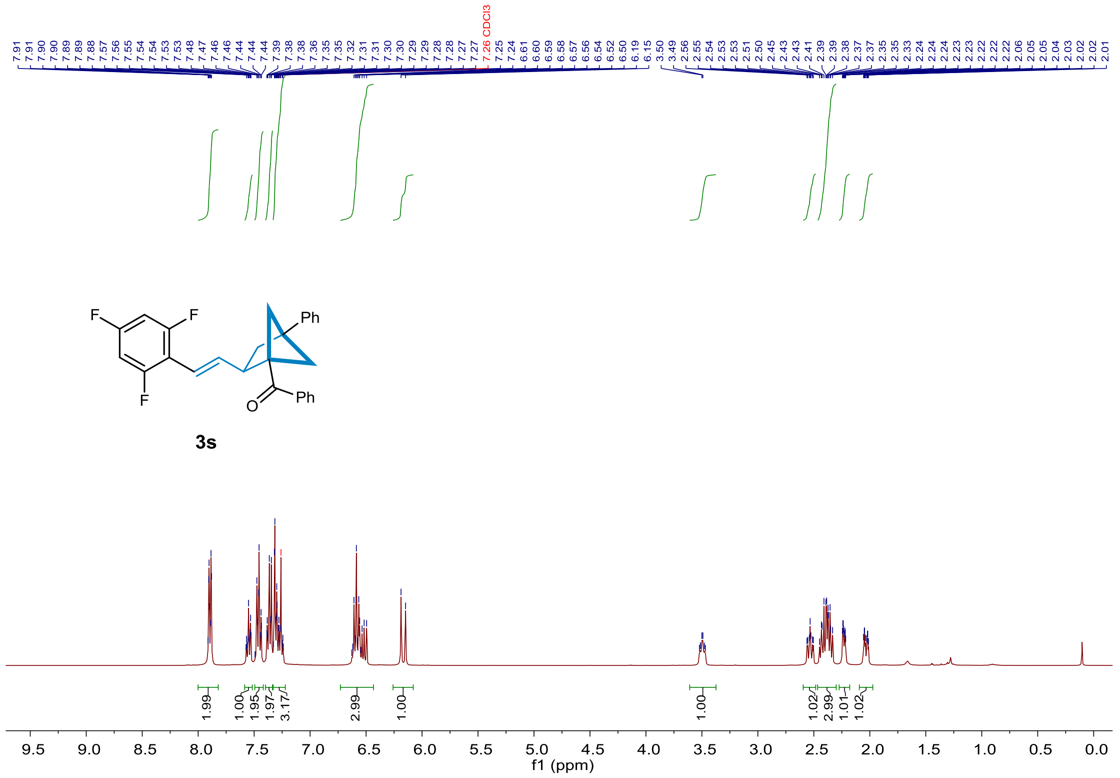

Supplementary Figure 56. **^{1}H NMR of the 3s** (400 MHz, $CDCl_3$)

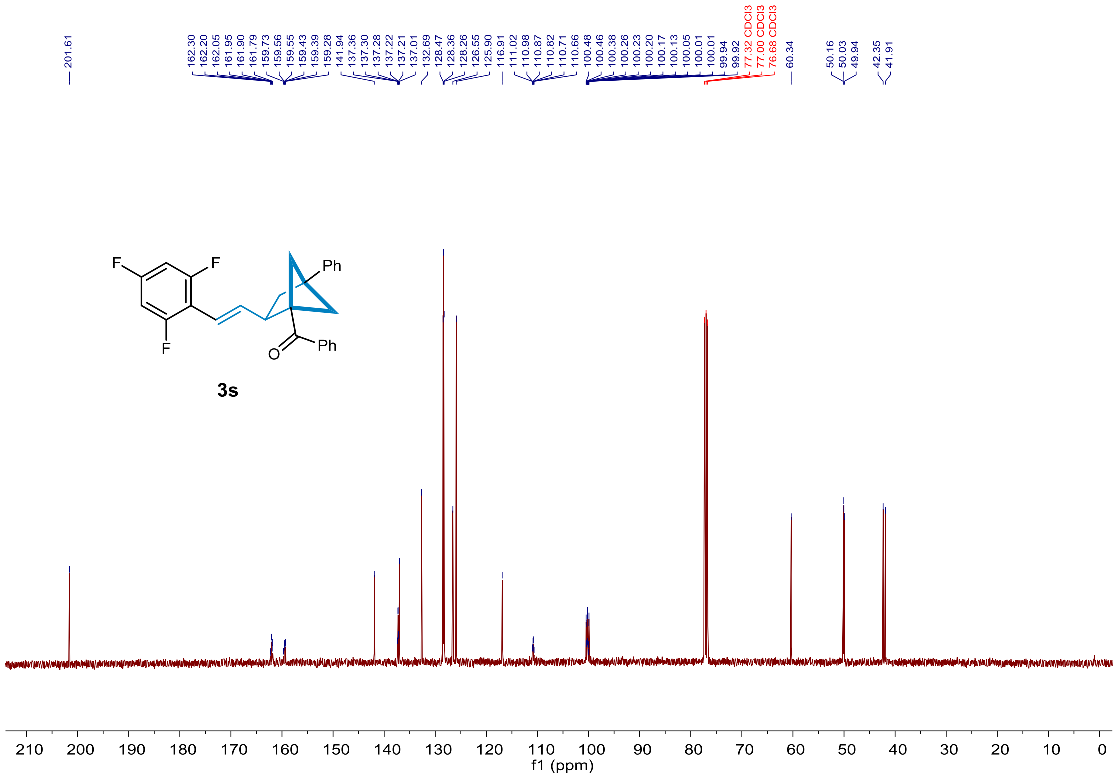

Supplementary Figure 57. **^{13}C NMR of the 3s** (101 MHz, $CDCl_3$)

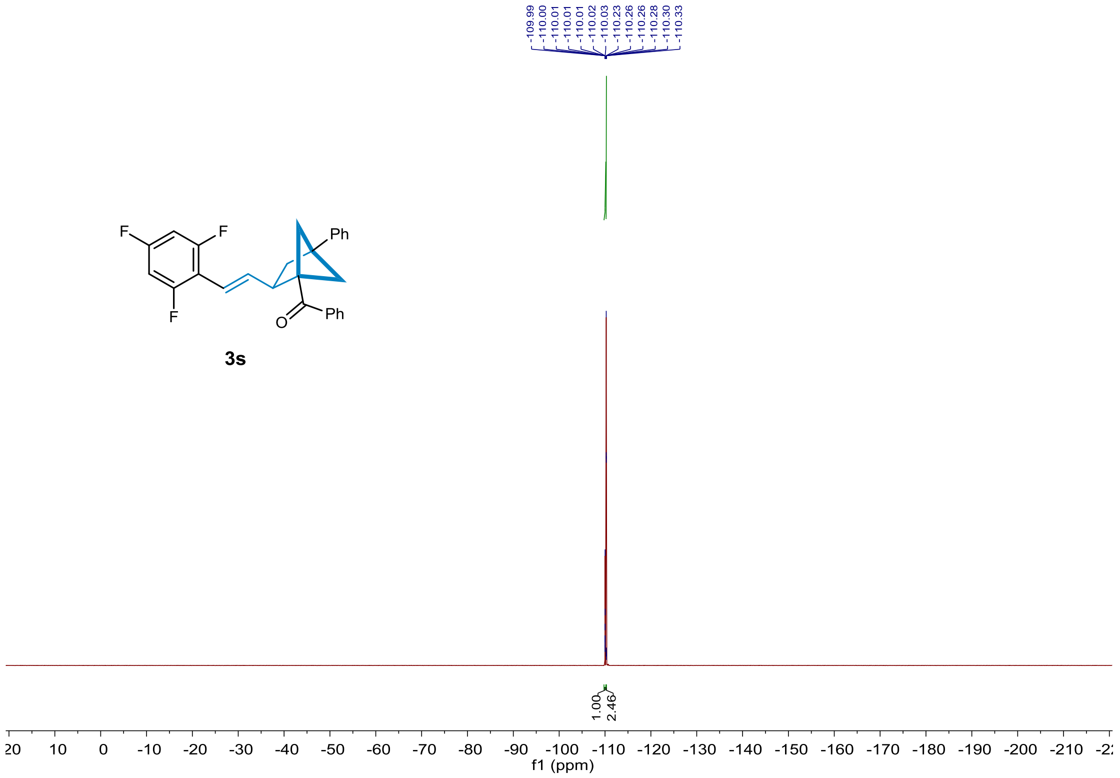

Supplementary Figure 58. **^{19}F NMR of the 3s** (377 MHz, $CDCl_3$)

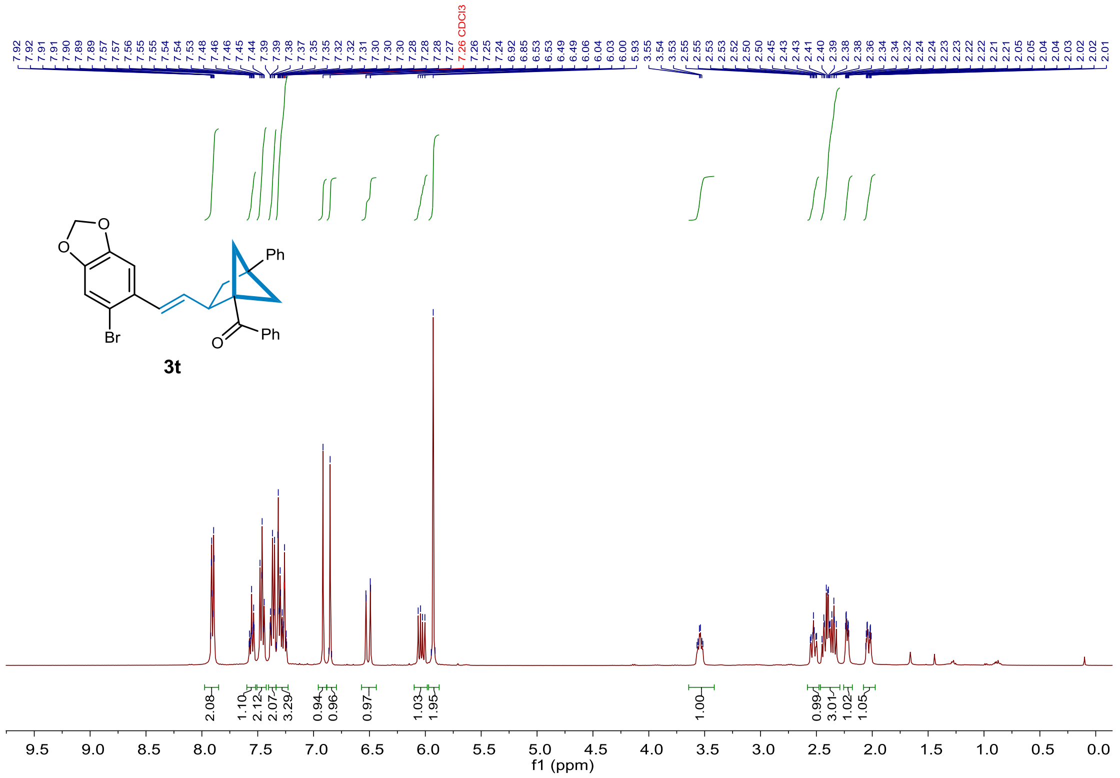

Supplementary Figure 59. **^{1}H NMR of the 3t** (400 MHz, $CDCl_3$)

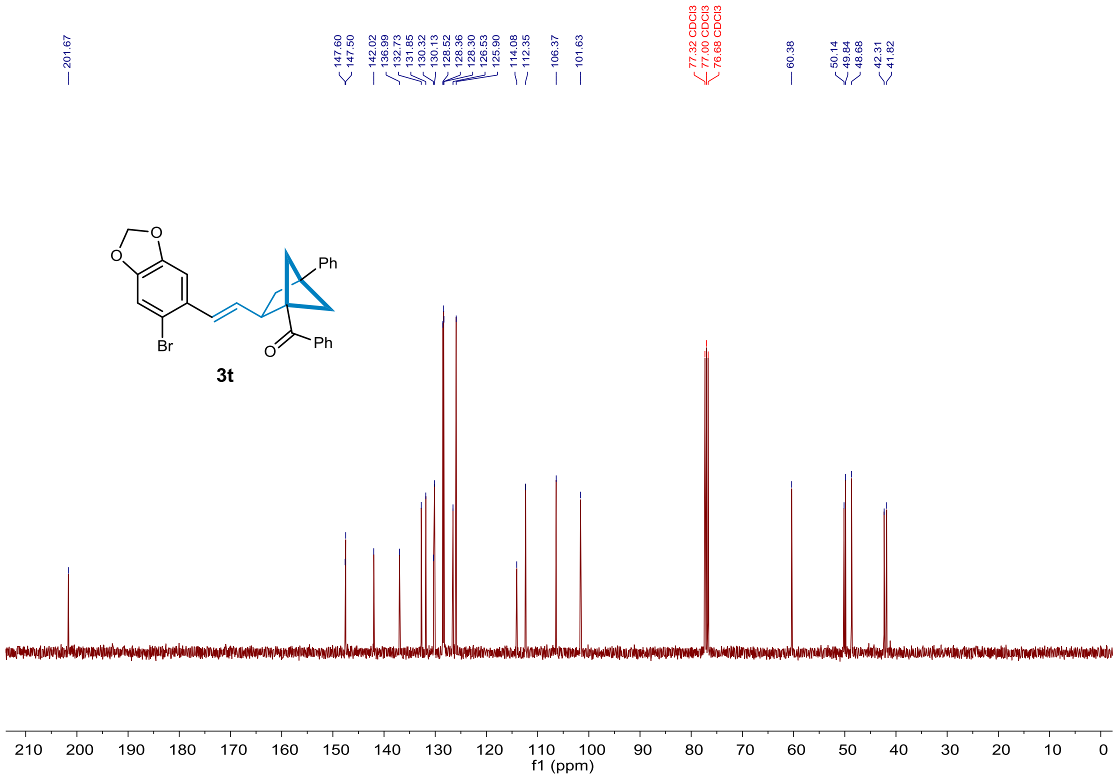

Supplementary Figure 60. **^{13}C NMR of the 3t** (101 MHz, $CDCl_3$)

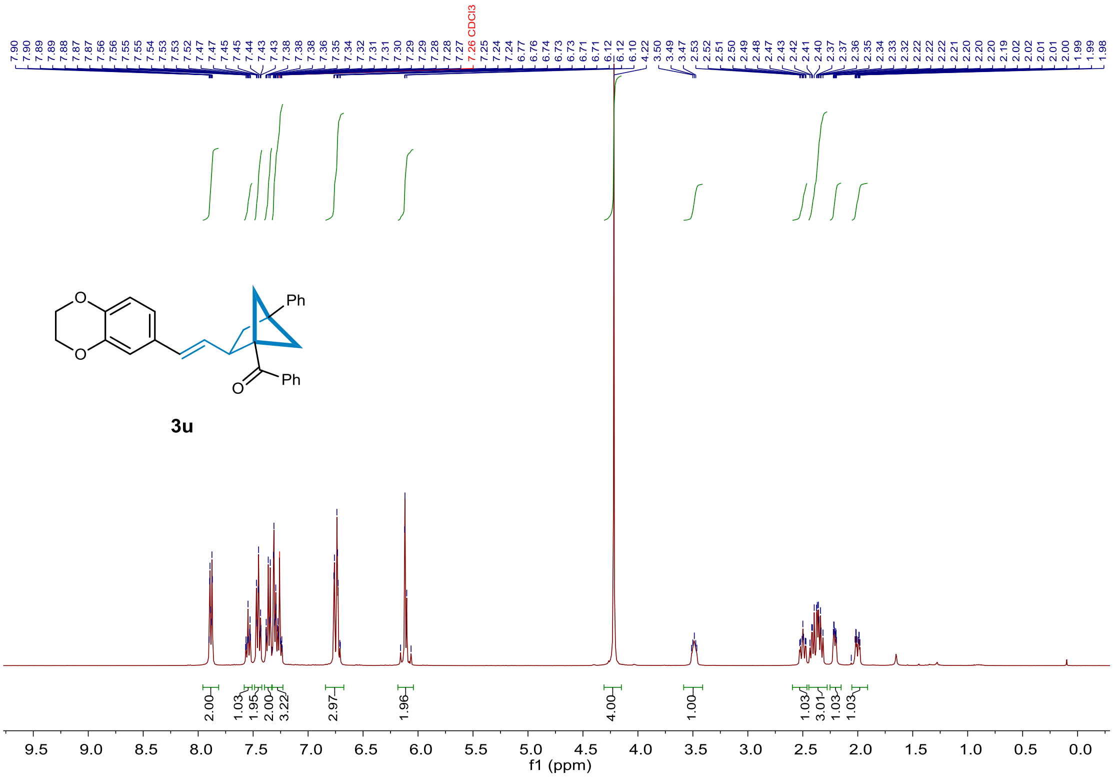

Supplementary Figure 61. **^{1}H NMR of the 3u** (400 MHz, $CDCl_3$)

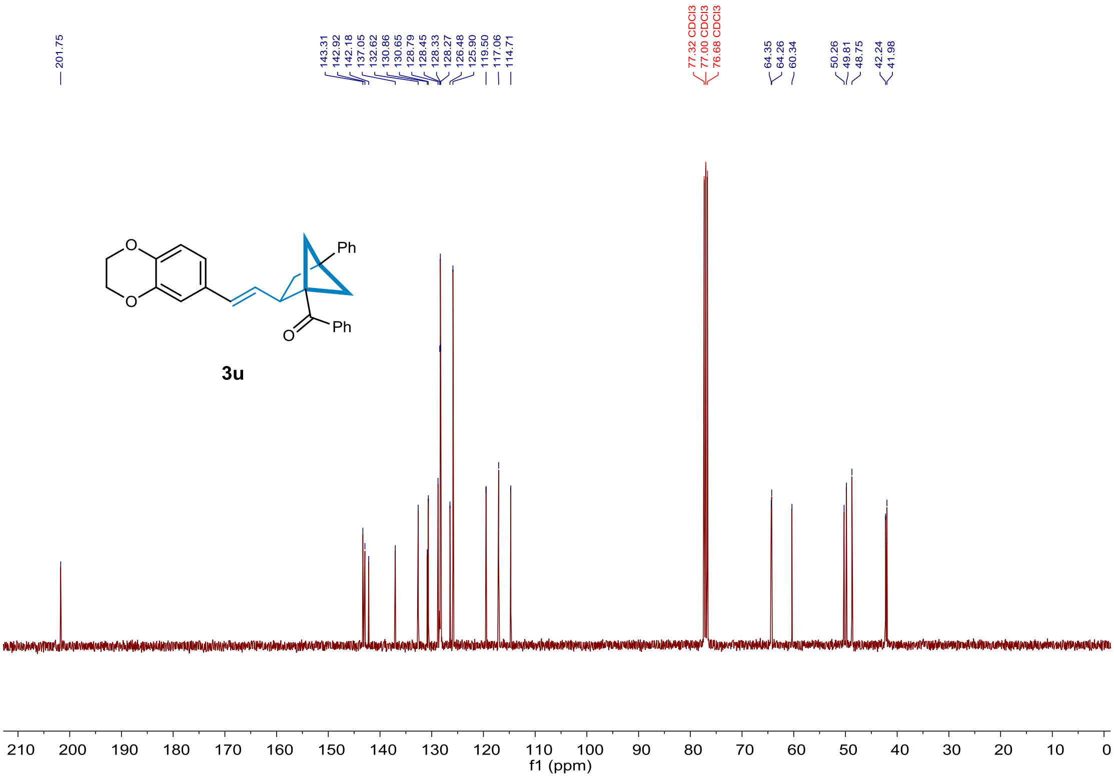

Supplementary Figure 62. **^{13}C NMR of the 3u** (101 MHz, $CDCl_3$)

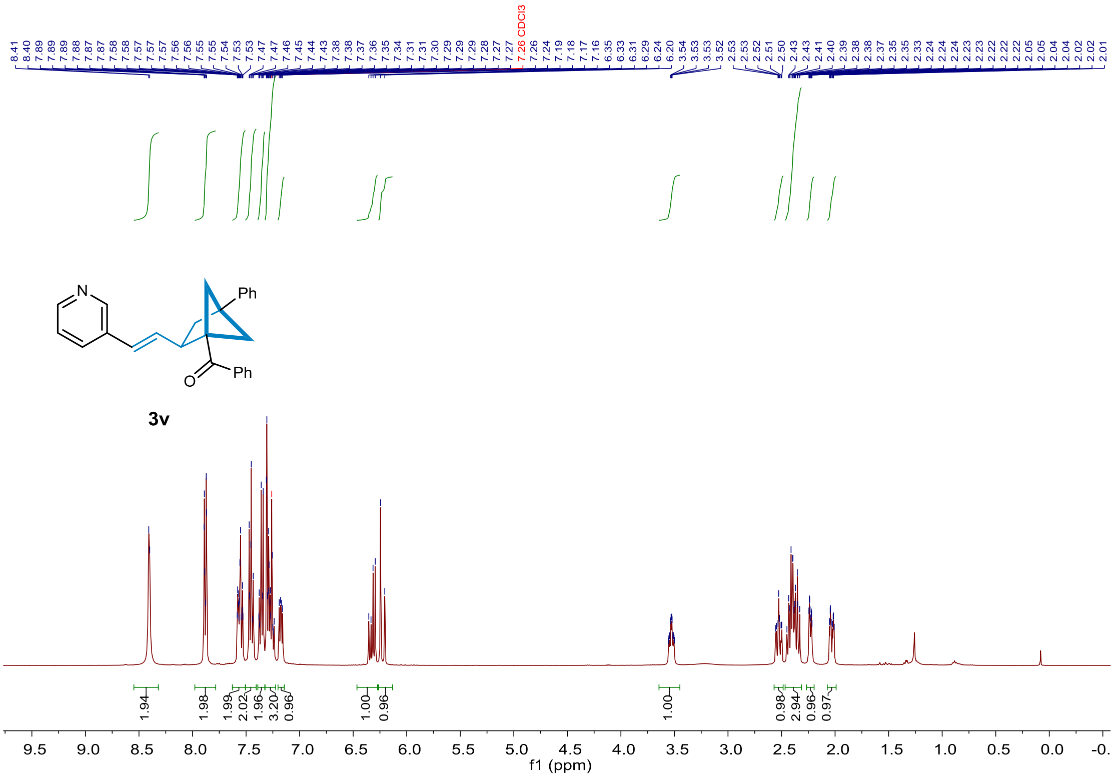

Supplementary Figure 63. **^{1}H NMR of the 3v** (400 MHz, $CDCl_3$)

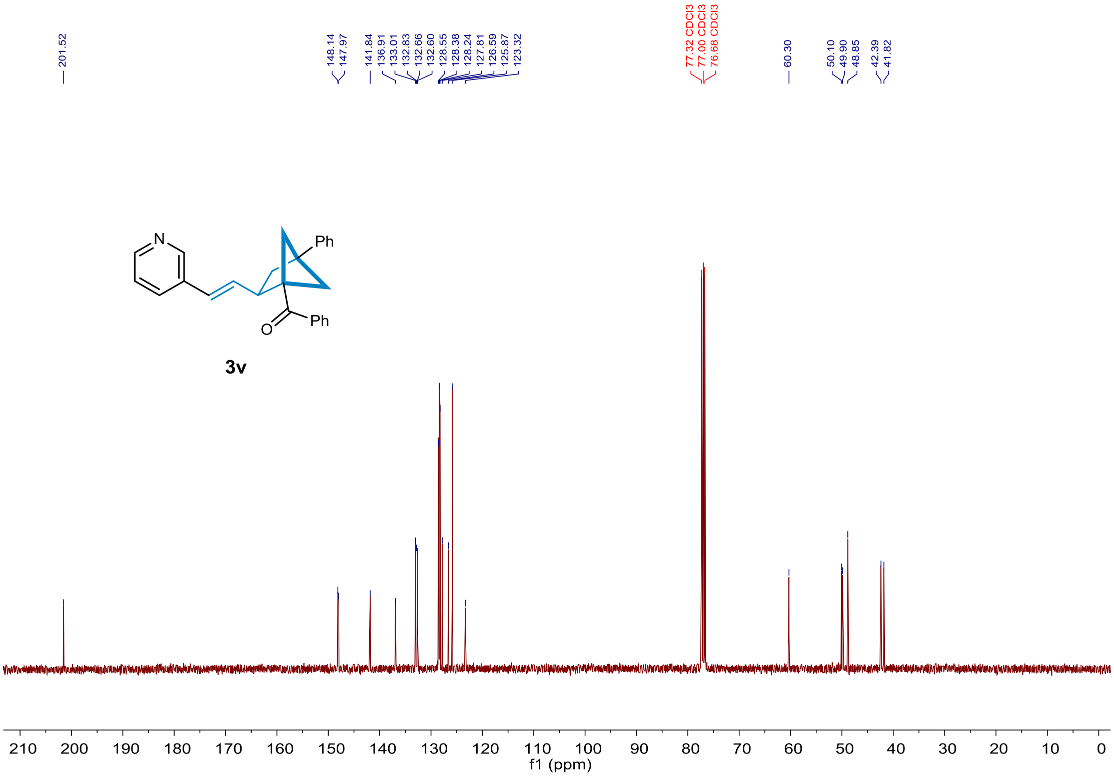

Supplementary Figure 64. **^{13}C NMR of the 3v** (101 MHz, $CDCl_3$)

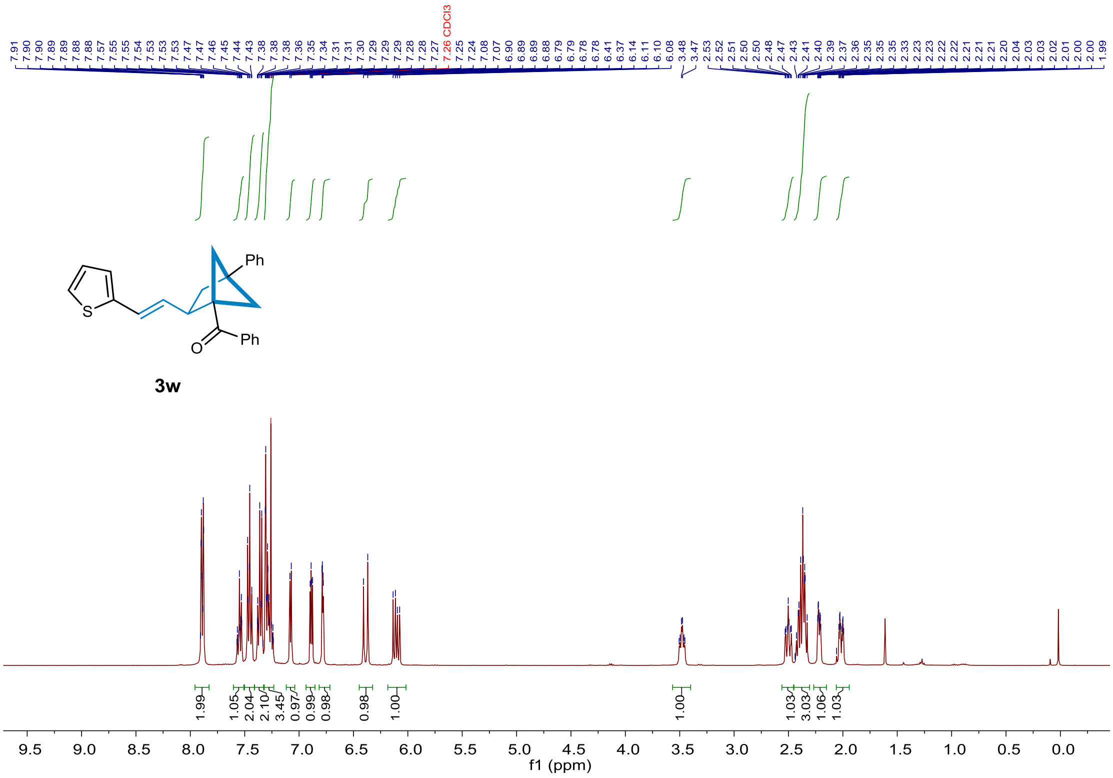

Supplementary Figure 65. **^{1}H NMR of the 3w** (400 MHz, $CDCl_3$)

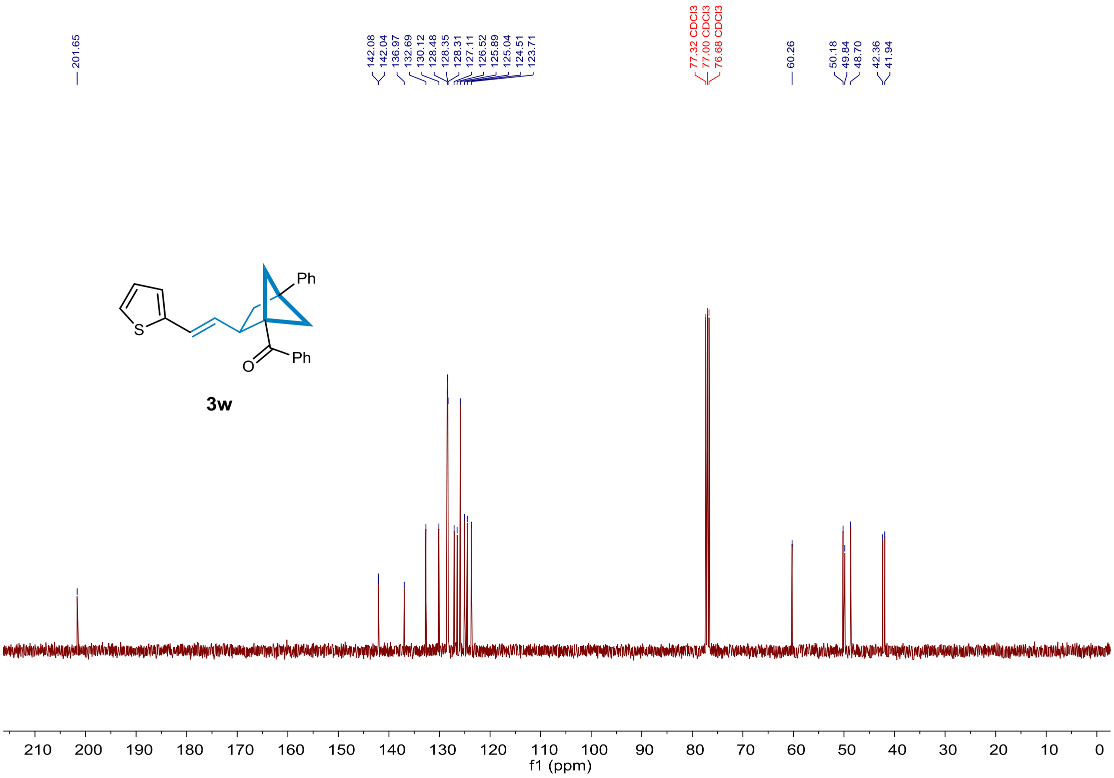

Supplementary Figure 66. **^{13}C NMR of the 3w** (101 MHz, $CDCl_3$)

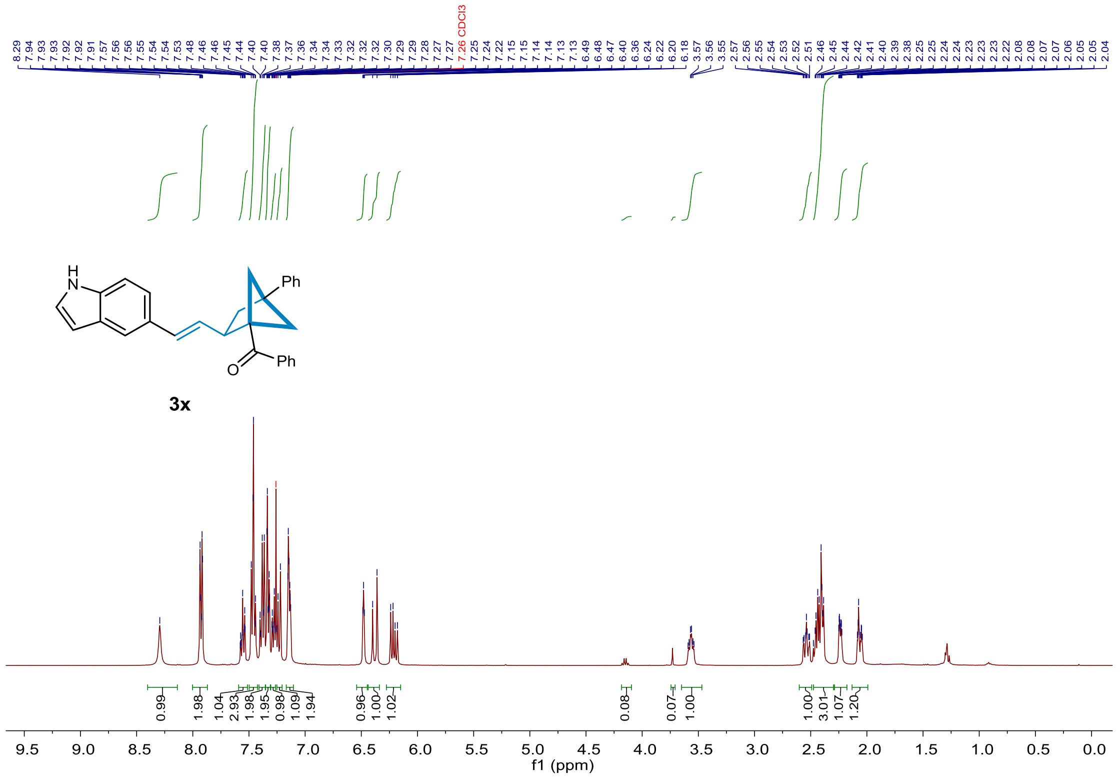

Supplementary Figure 67. **^{1}H NMR of the 3x** (400 MHz, $CDCl_3$)

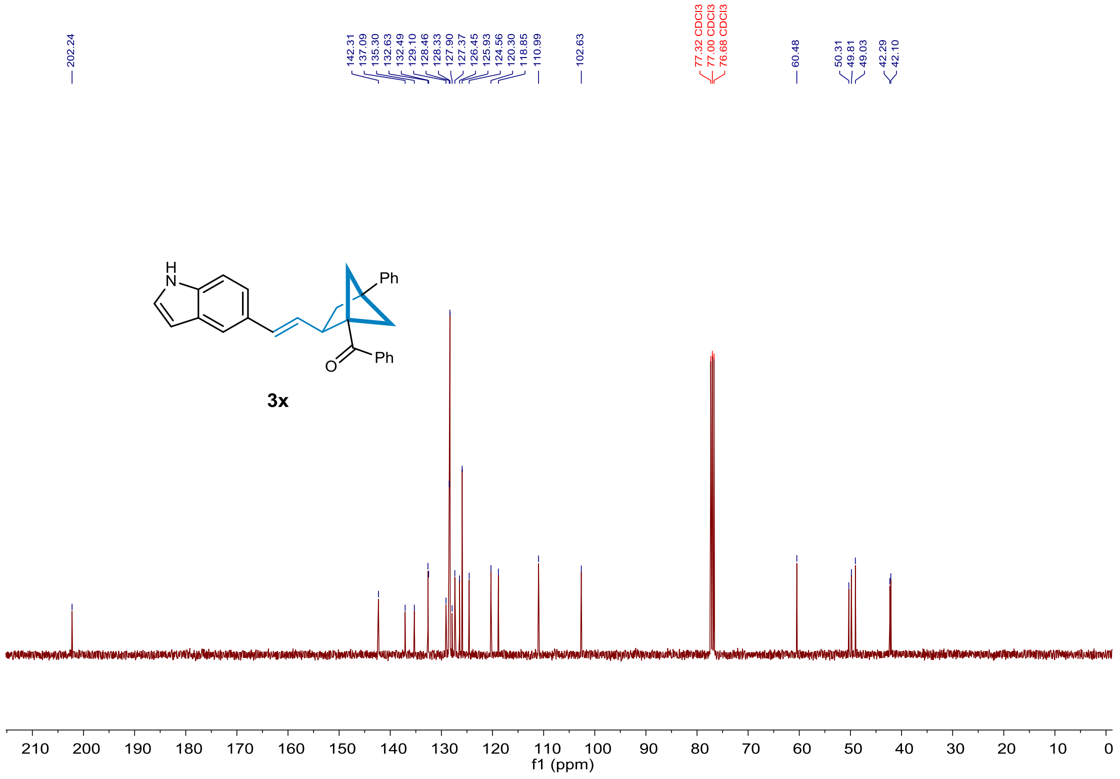

Supplementary Figure 68. **^{13}C NMR of the 3x** (101 MHz, $CDCl_3$)

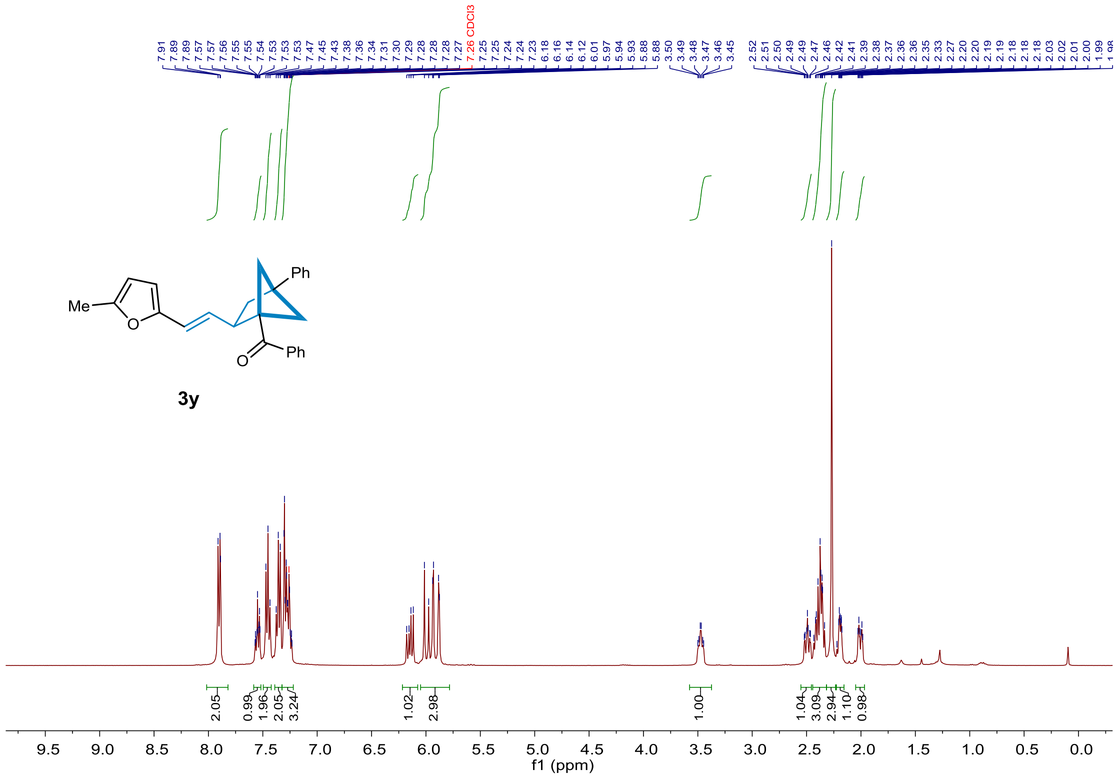

Supplementary Figure 69. ^{1}**H NMR of the 3y** (400 MHz, $CDCl_3$)

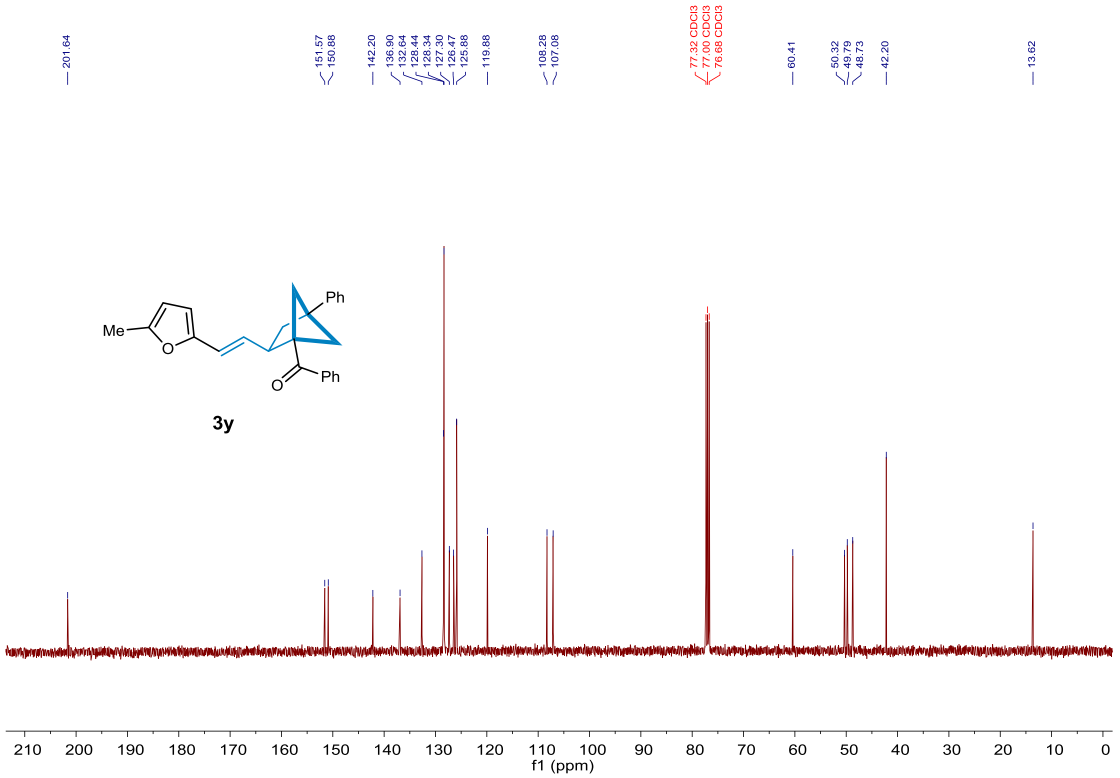

Supplementary Figure 70. ^{13}**C NMR of the 3y** (101 MHz, $CDCl_3$)

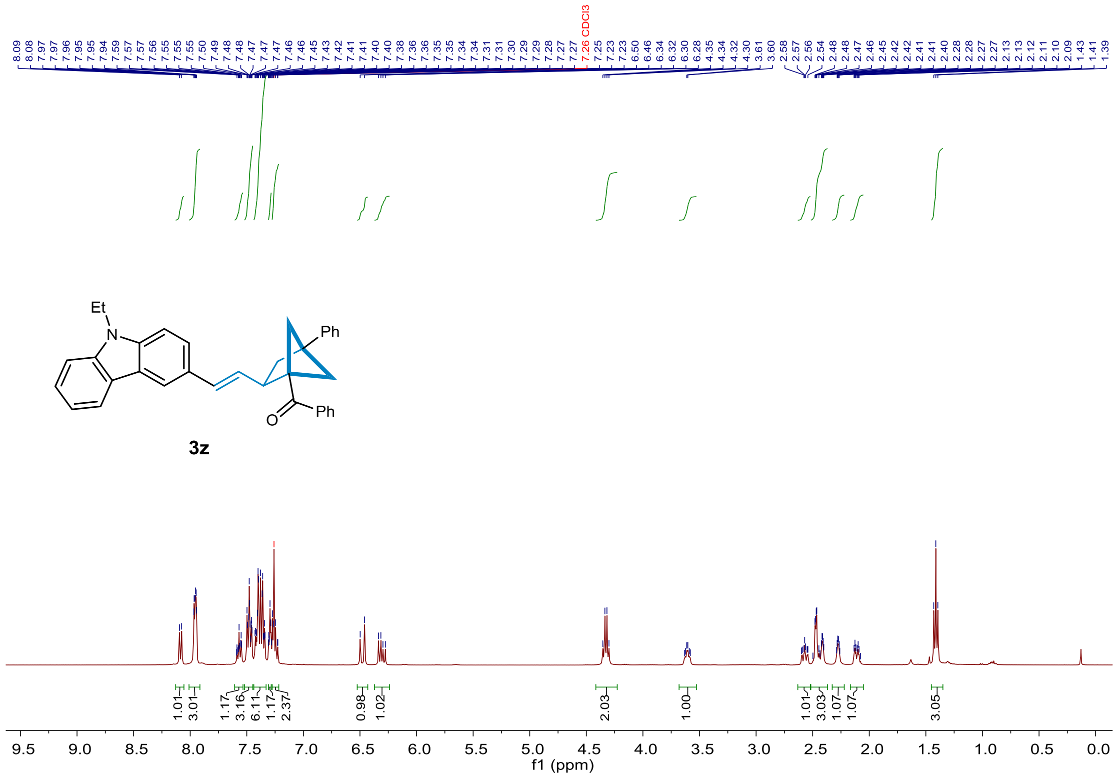

Supplementary Figure 71. **^{1}H NMR of the 3z** (400 MHz, $CDCl_3$)

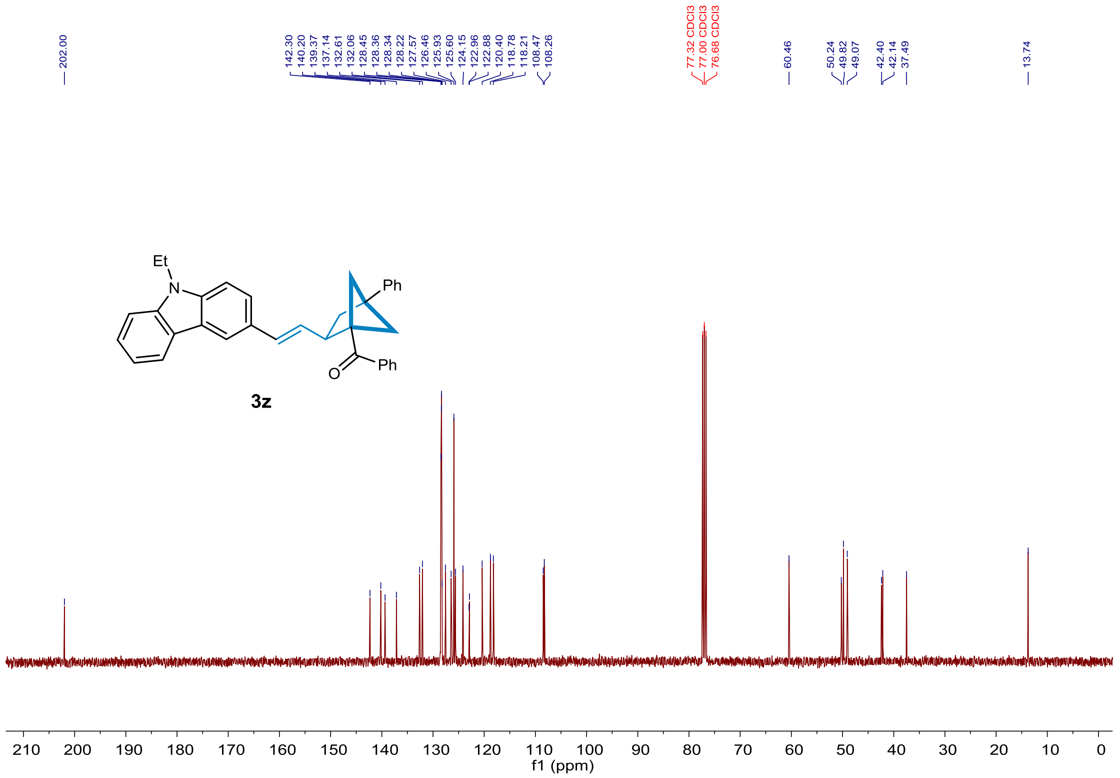

Supplementary Figure 72. **^{13}C NMR of the 3z** (101 MHz, $CDCl_3$)

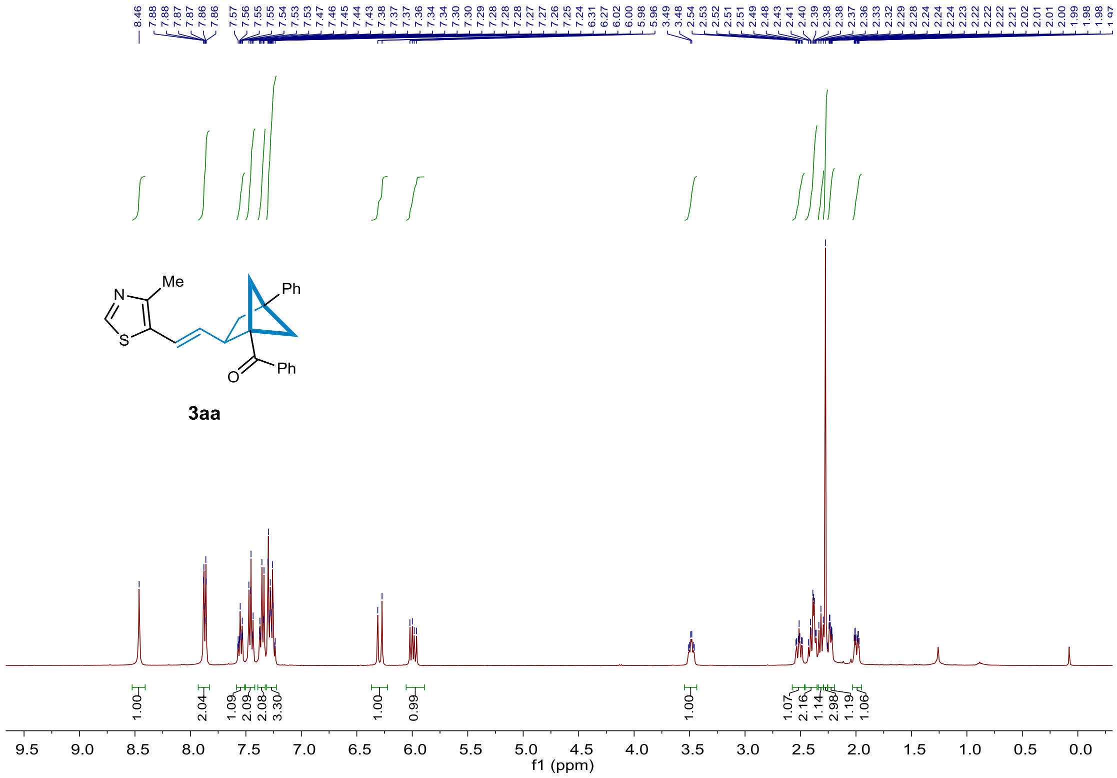

Supplementary Figure 73. **^{1}H NMR of the 3aa** (400 MHz, $CDCl_3$)

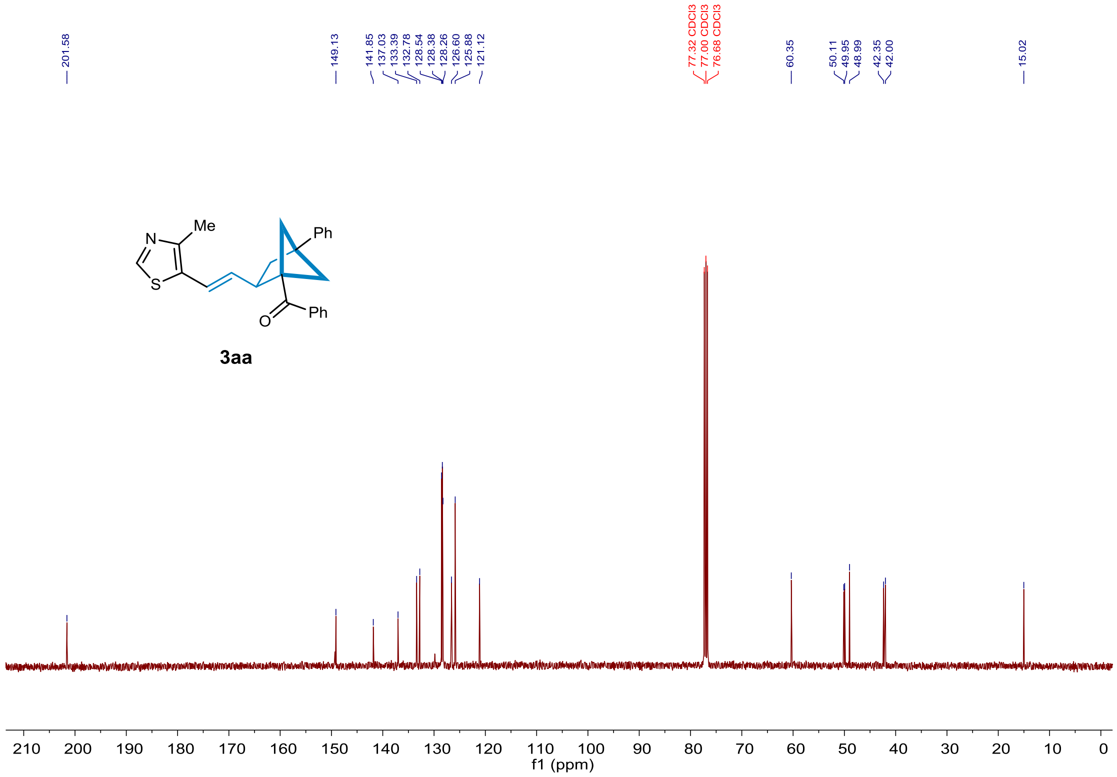

Supplementary Figure 74. **^{13}C NMR of the 3aa** (101 MHz, $CDCl_3$)

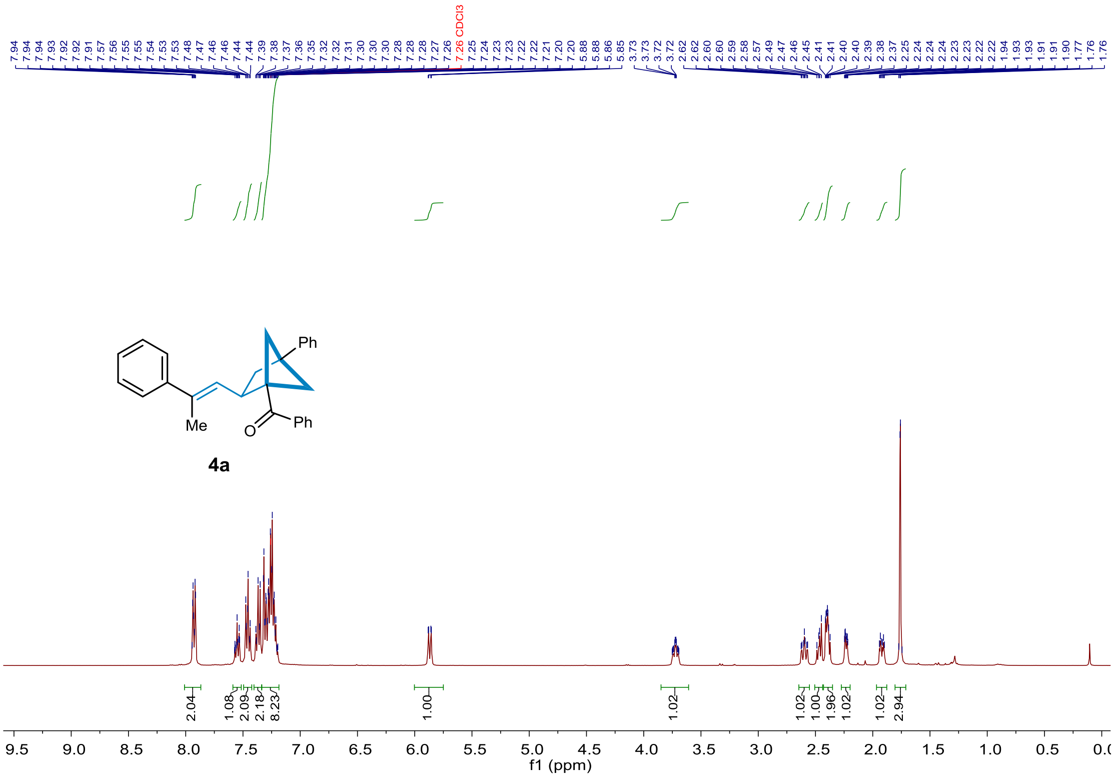

Supplementary Figure 75. **^{1}H NMR of the 4a** (400 MHz, $CDCl_3$)

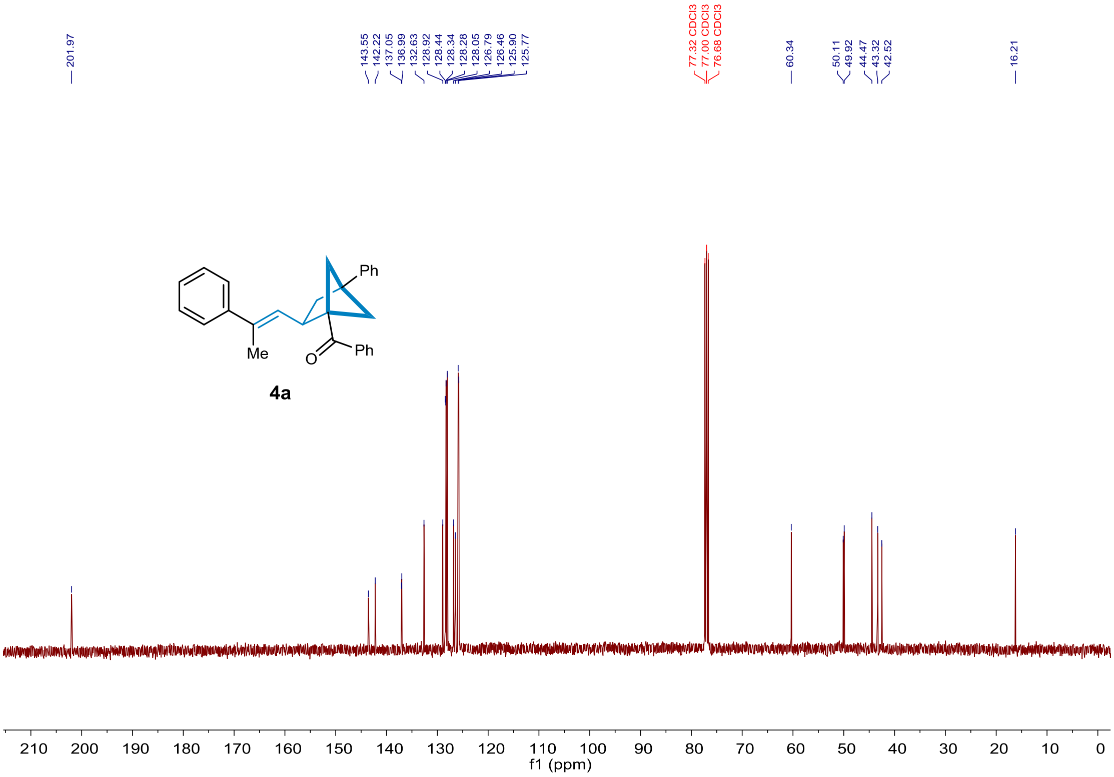

Supplementary Figure 76. **^{13}C NMR of the 4a** (101 MHz, $CDCl_3$)

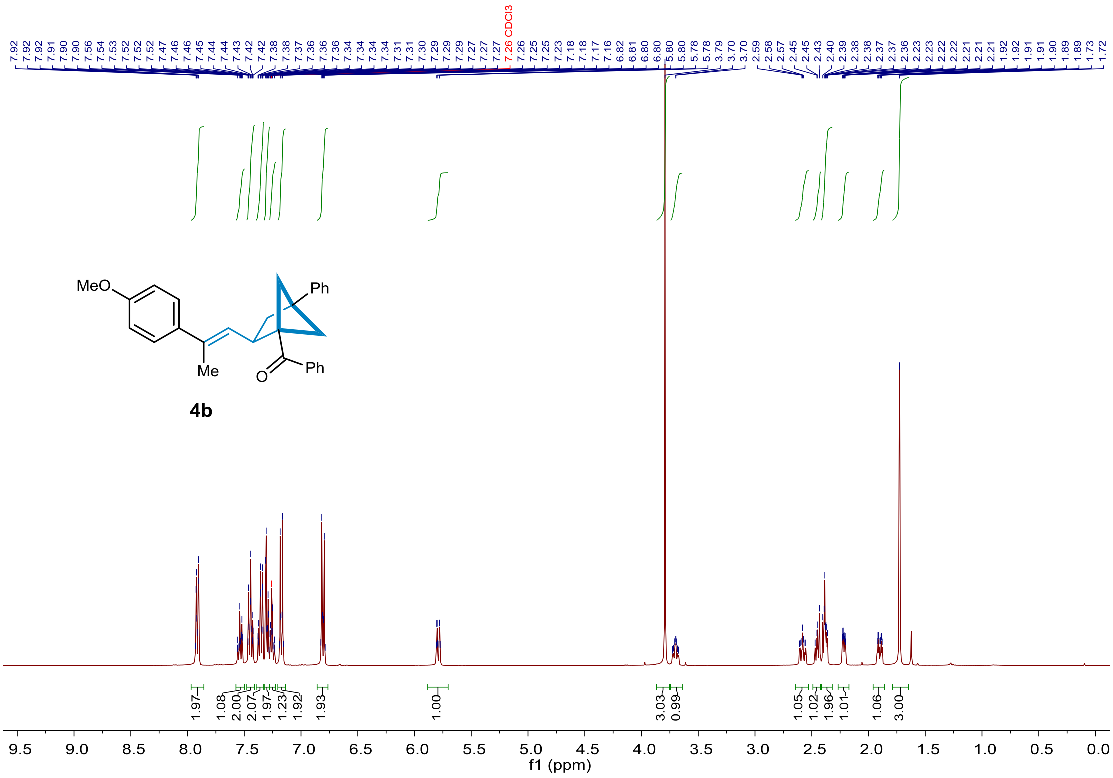

Supplementary Figure 77. ^{1}H NMR of the 4b (400 MHz, $CDCl_3$)

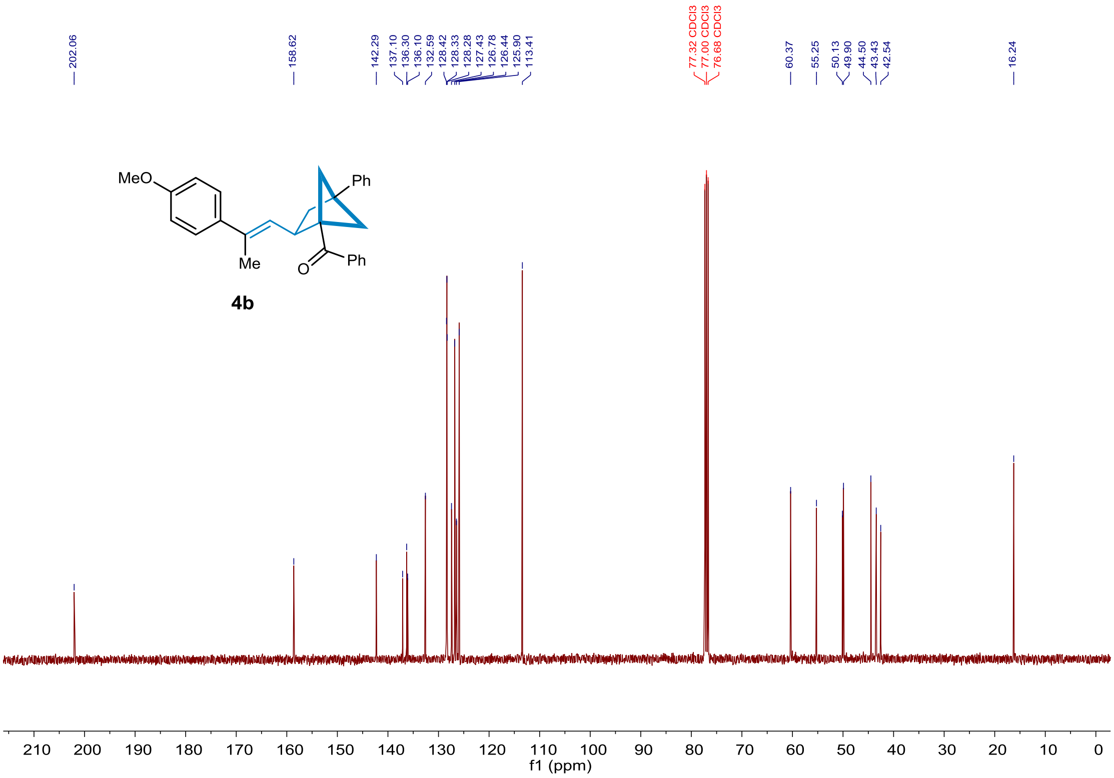

Supplementary Figure 78. ^{13}C NMR of the 4b (101 MHz, $CDCl_3$)

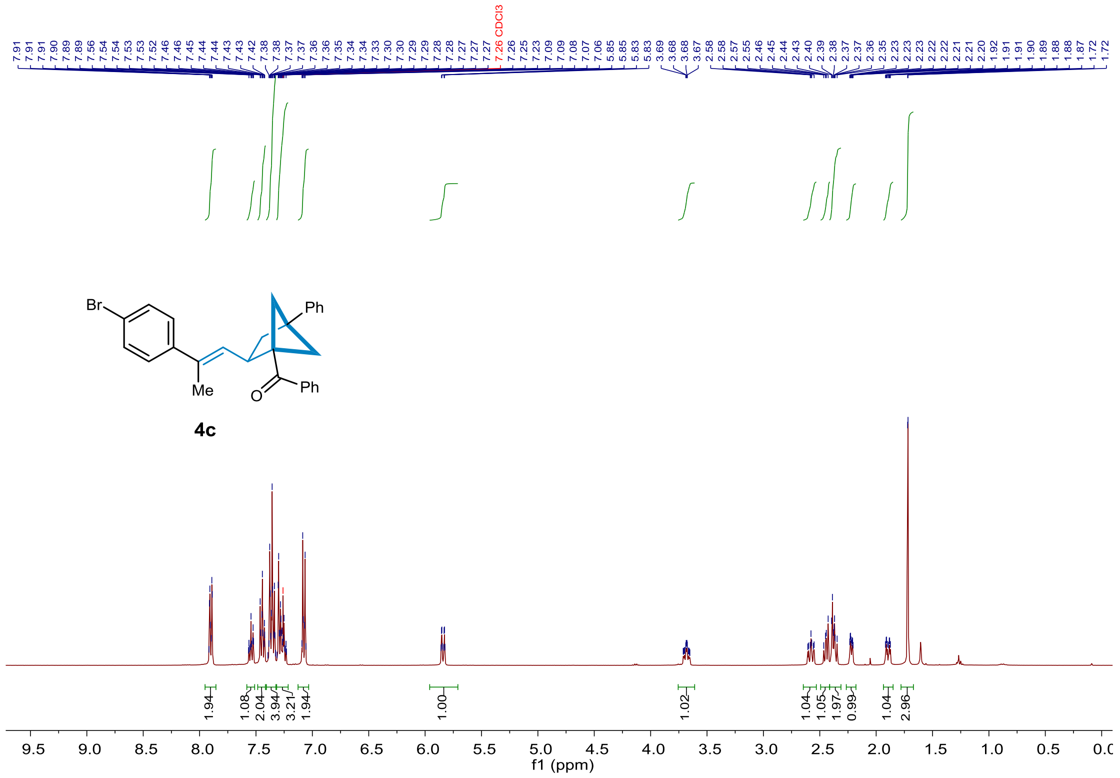

Supplementary Figure 79. **^{1}H NMR of the 4c** (400 MHz, $CDCl_3$)

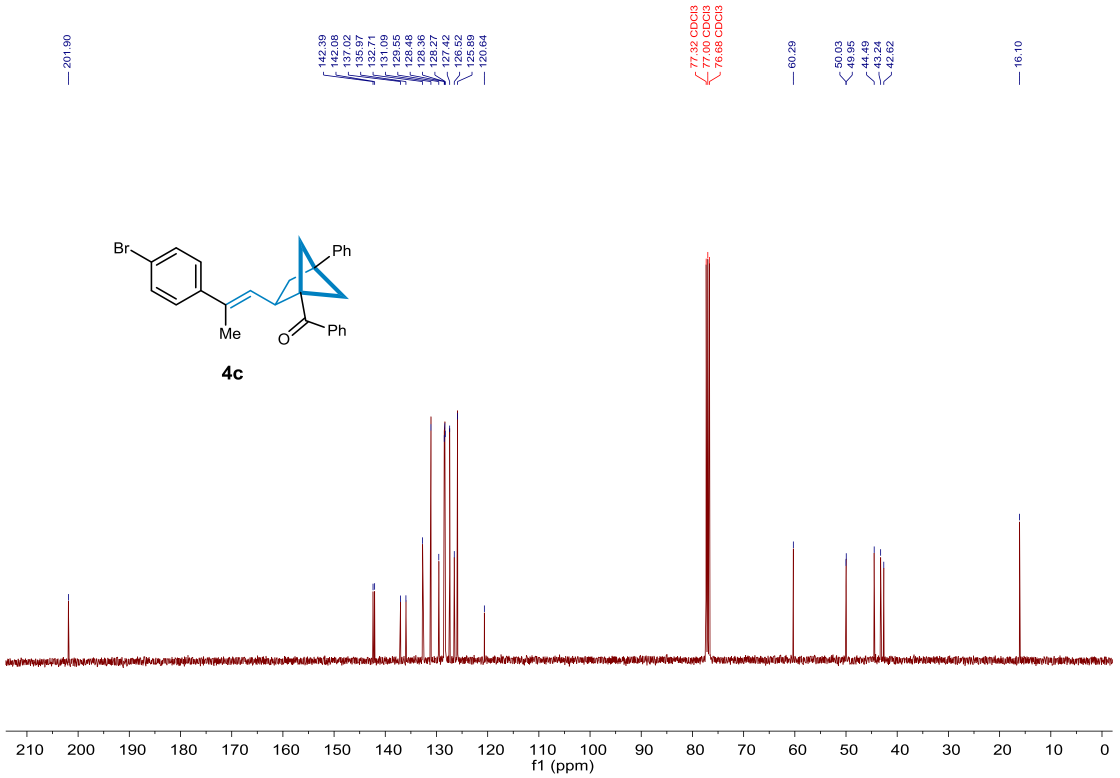

Supplementary Figure 80. **^{13}C NMR of the 4c** (101 MHz, $CDCl_3$)

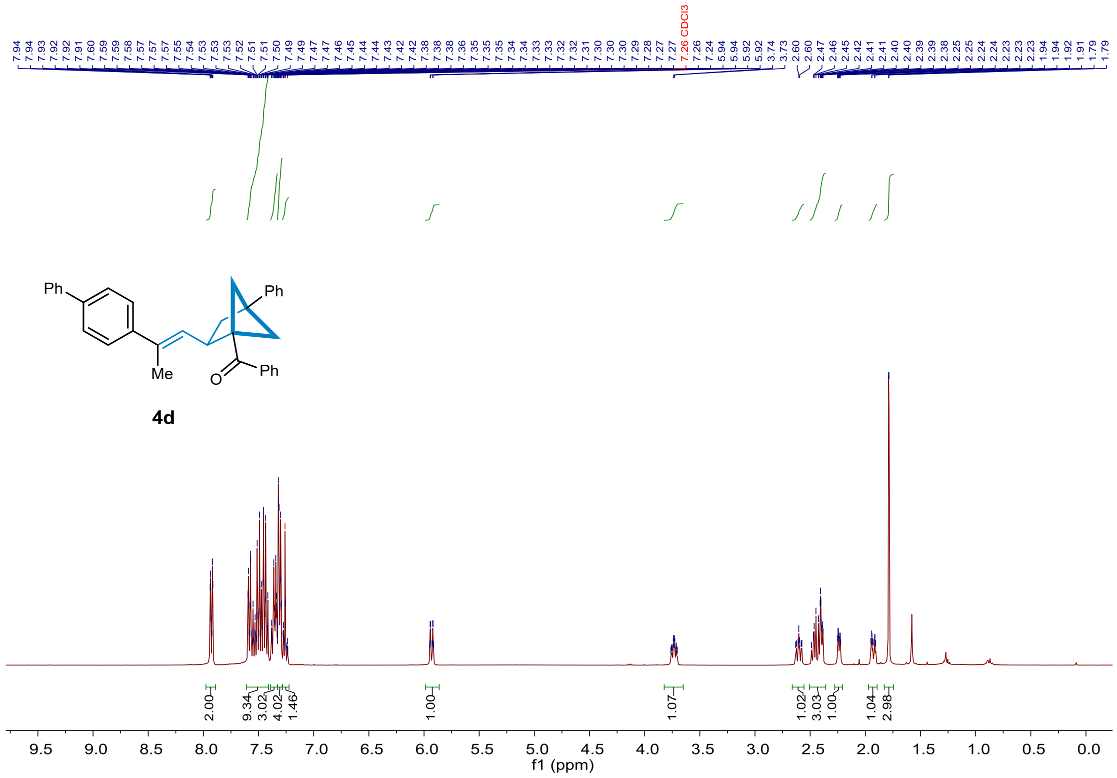

Supplementary Figure 81. **^{1}H NMR of the 4d** (400 MHz, $CDCl_3$)

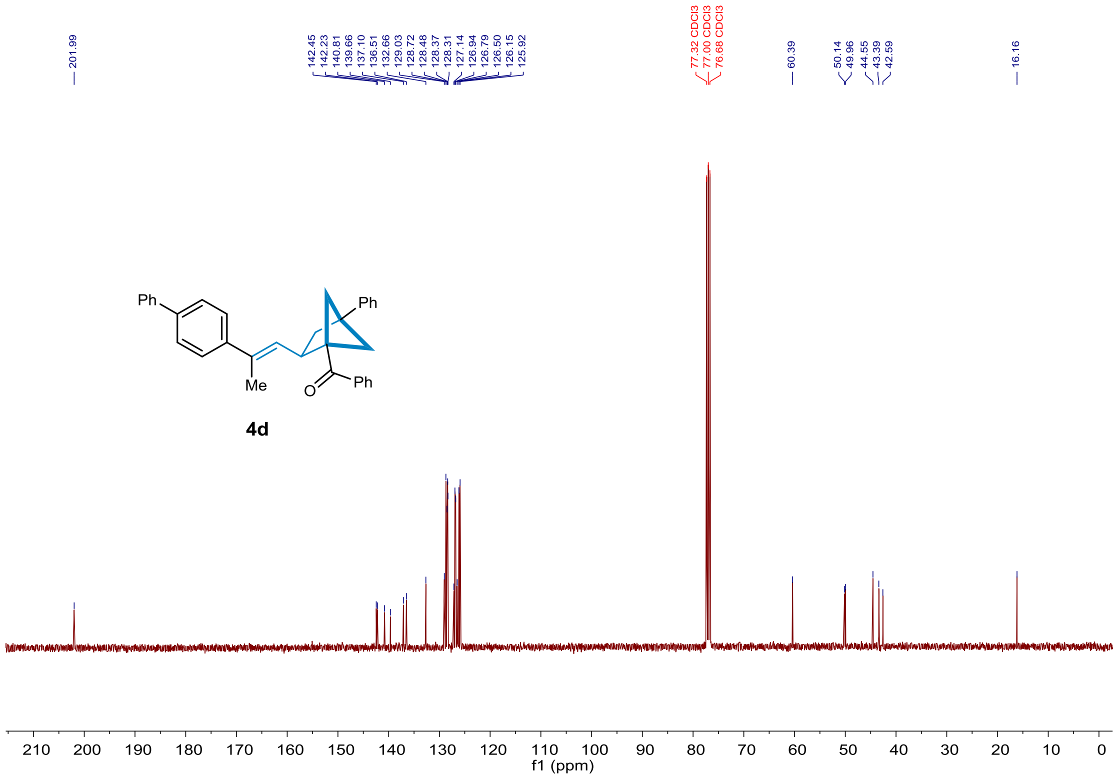

Supplementary Figure 82. **^{13}C NMR of the 4d** (101 MHz, $CDCl_3$)

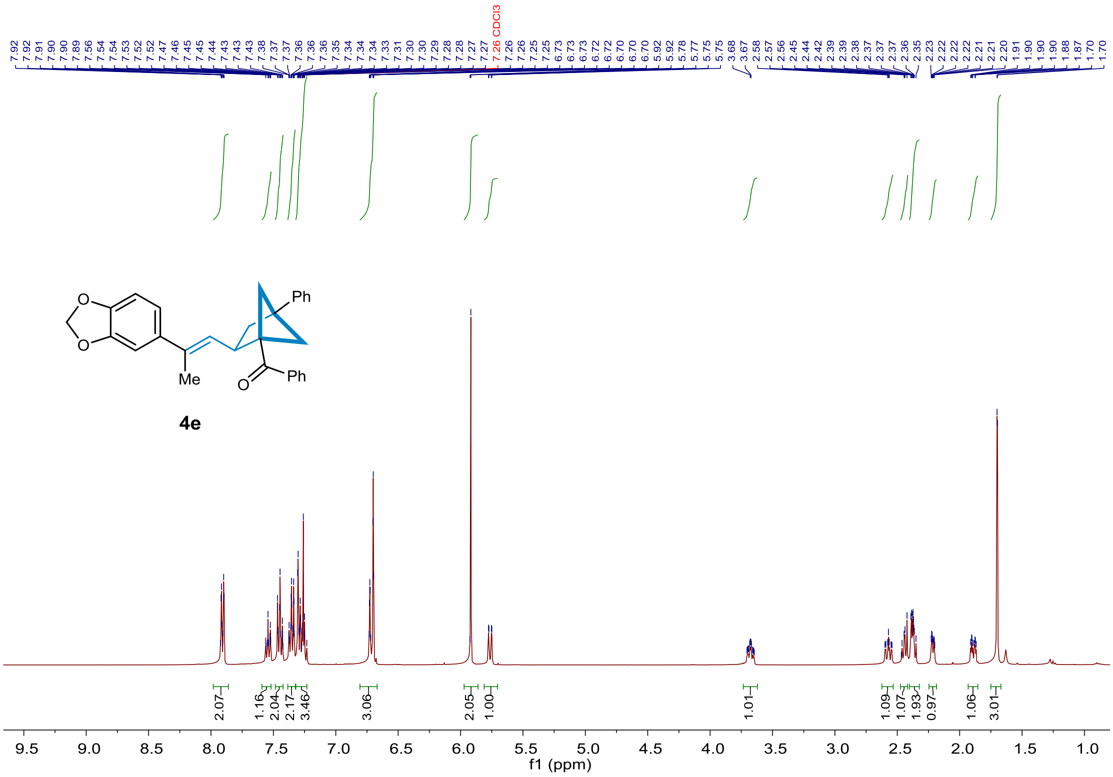

Supplementary Figure 83. ^{1}**H NMR of the 4e** (400 MHz, $CDCl_3$)

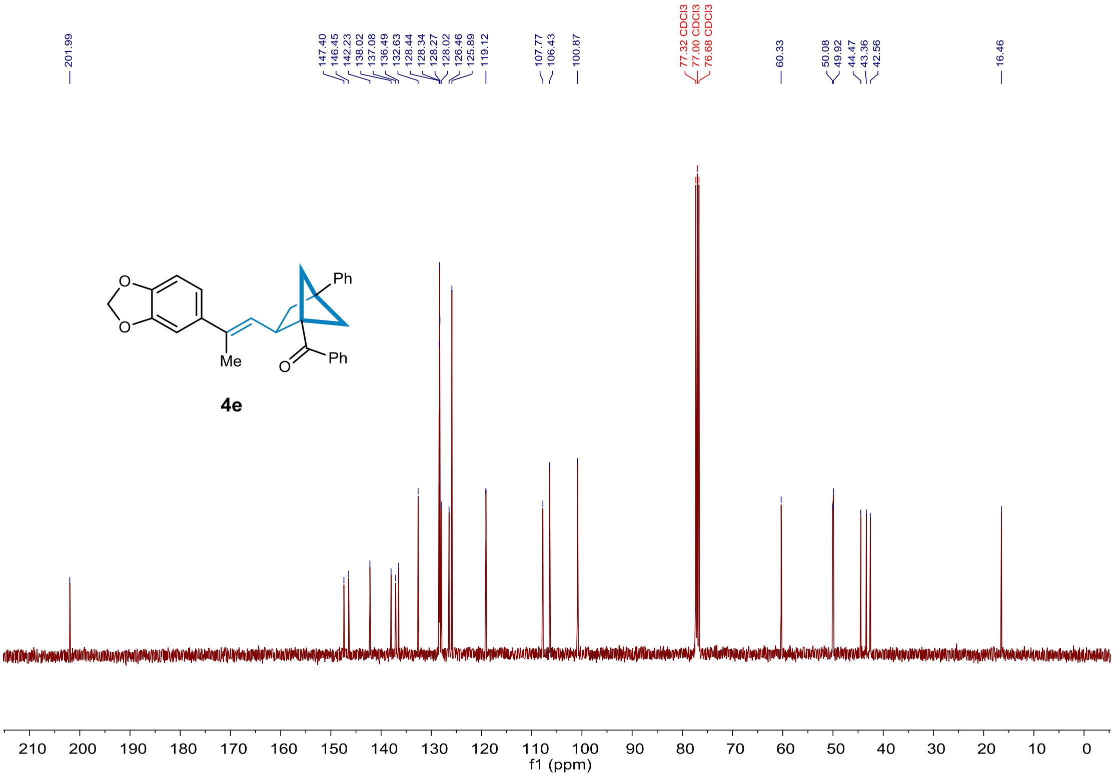

Supplementary Figure 84. ^{13}**C NMR of the 4e** (101 MHz, $CDCl_3$)

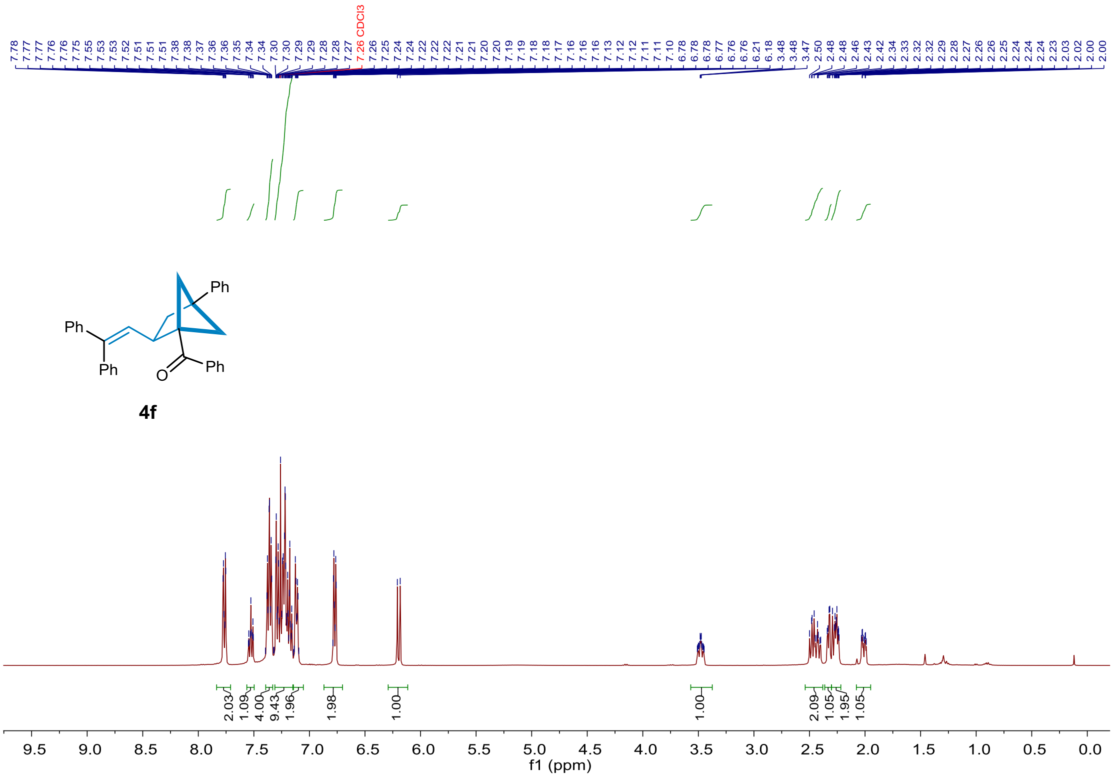

Supplementary Figure 85. **^{1}H NMR of the 4f** (400 MHz, $CDCl_3$)

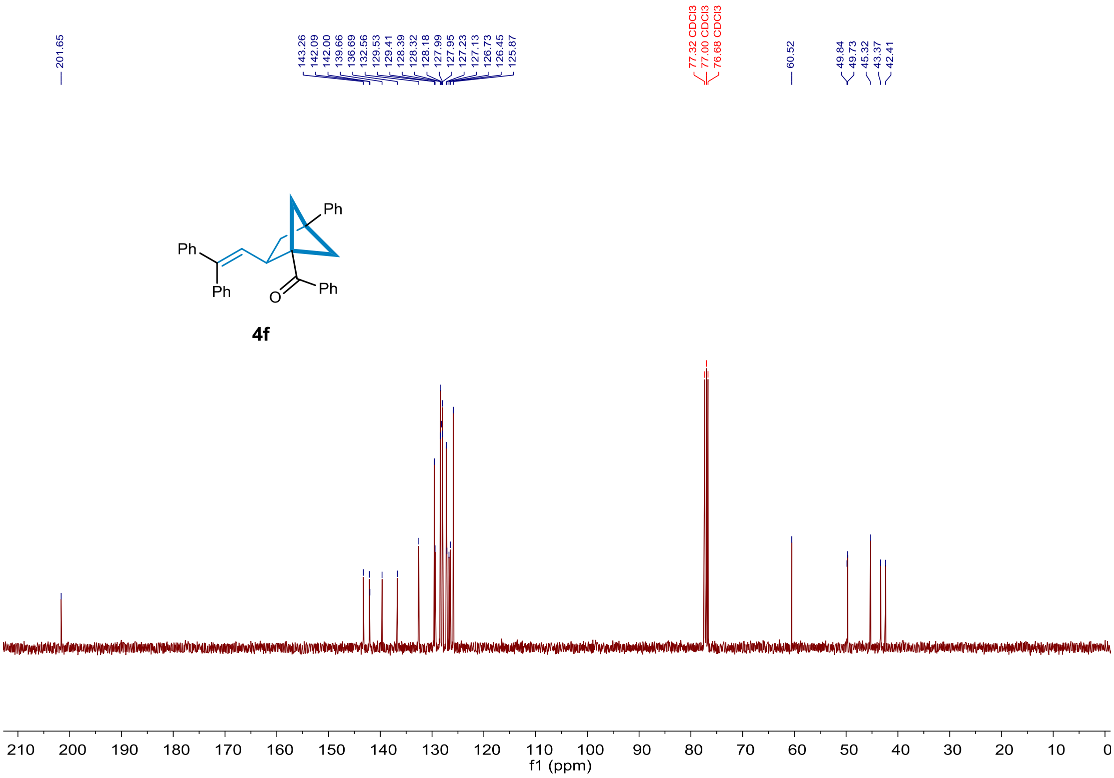

Supplementary Figure 86. **^{13}C NMR of the 4f** (101 MHz, $CDCl_3$)

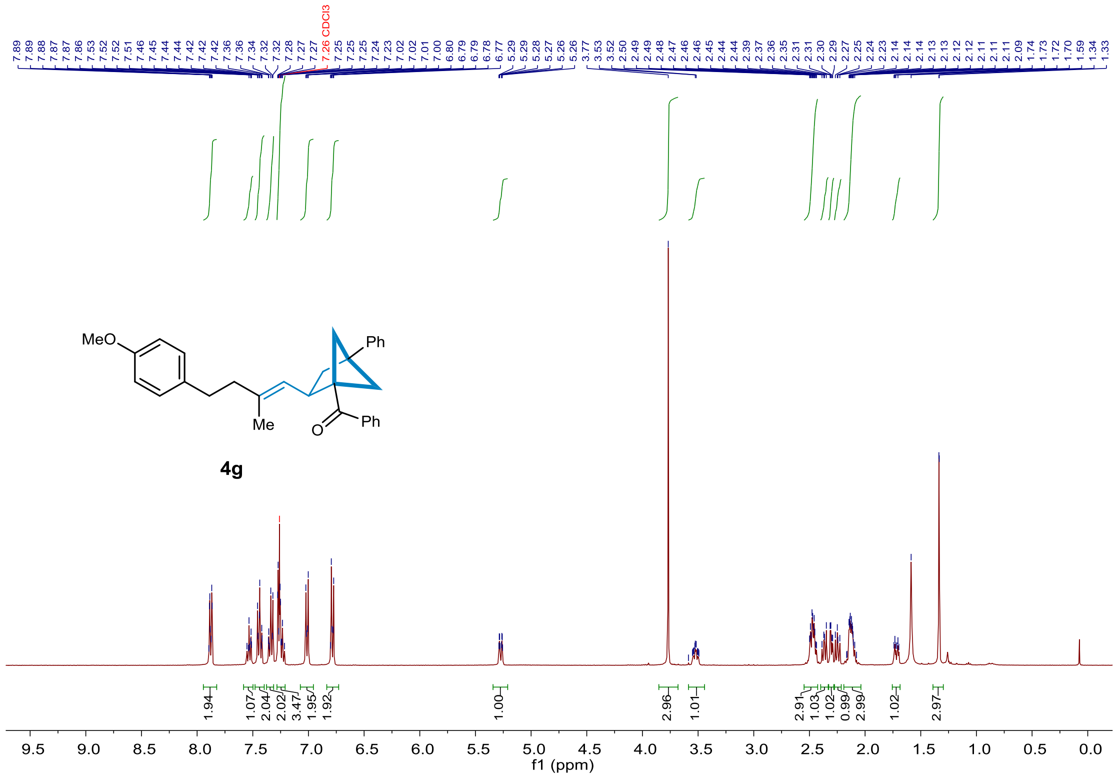

Supplementary Figure 87. **^{1}H NMR of the 4g** (400 MHz, $CDCl_3$)

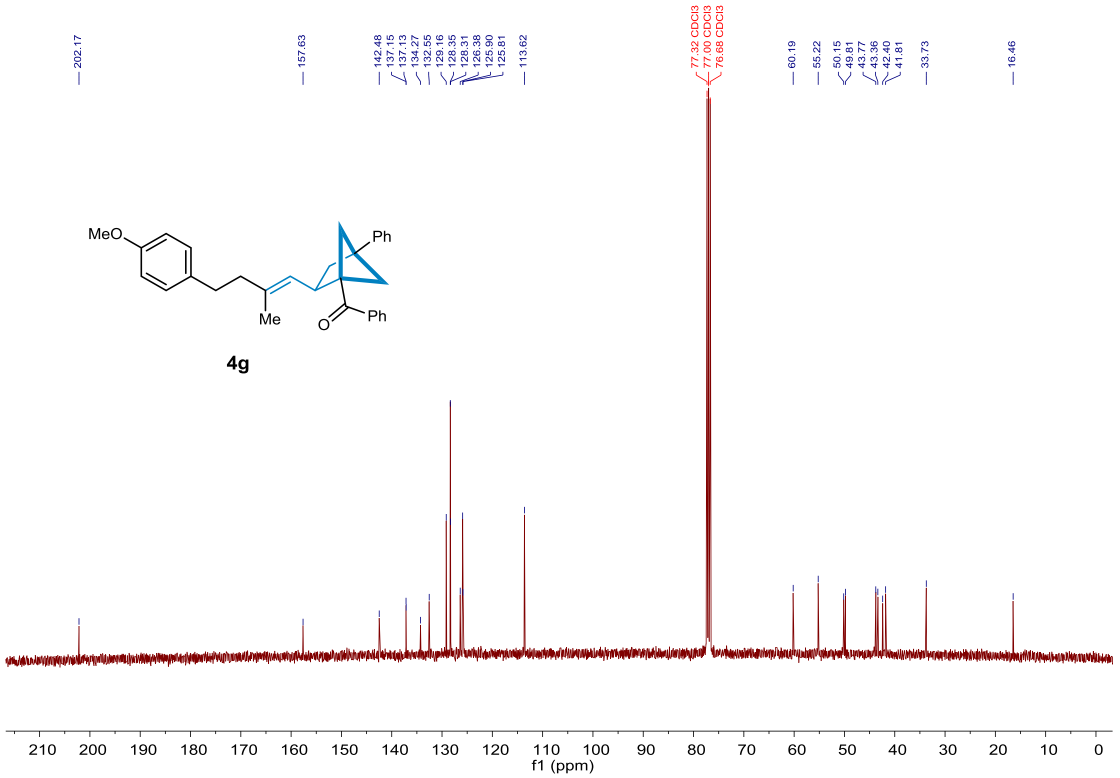

Supplementary Figure 88. **^{13}C NMR of the 4g** (101 MHz, $CDCl_3$)

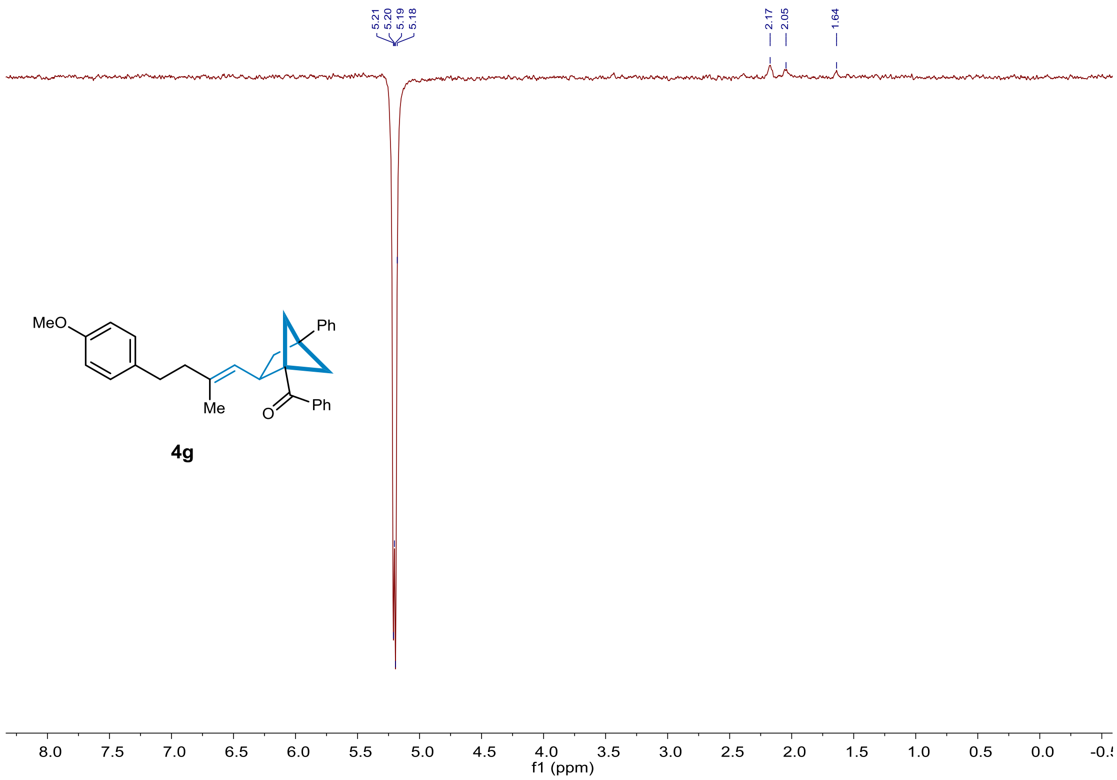

Supplementary Figure 89. 1D NOE of the 4g (500 MHz, $CDCl_3$)

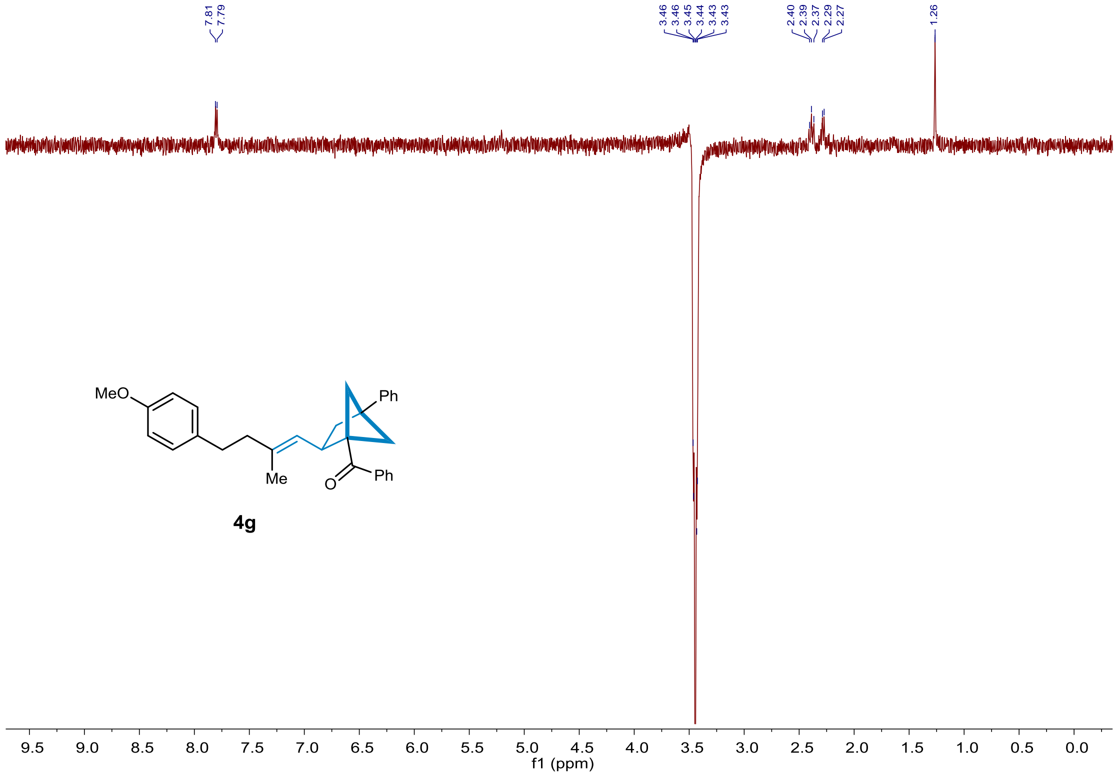

Supplementary Figure 90. 1D NOE of the 4g (500 MHz, $CDCl_3$)

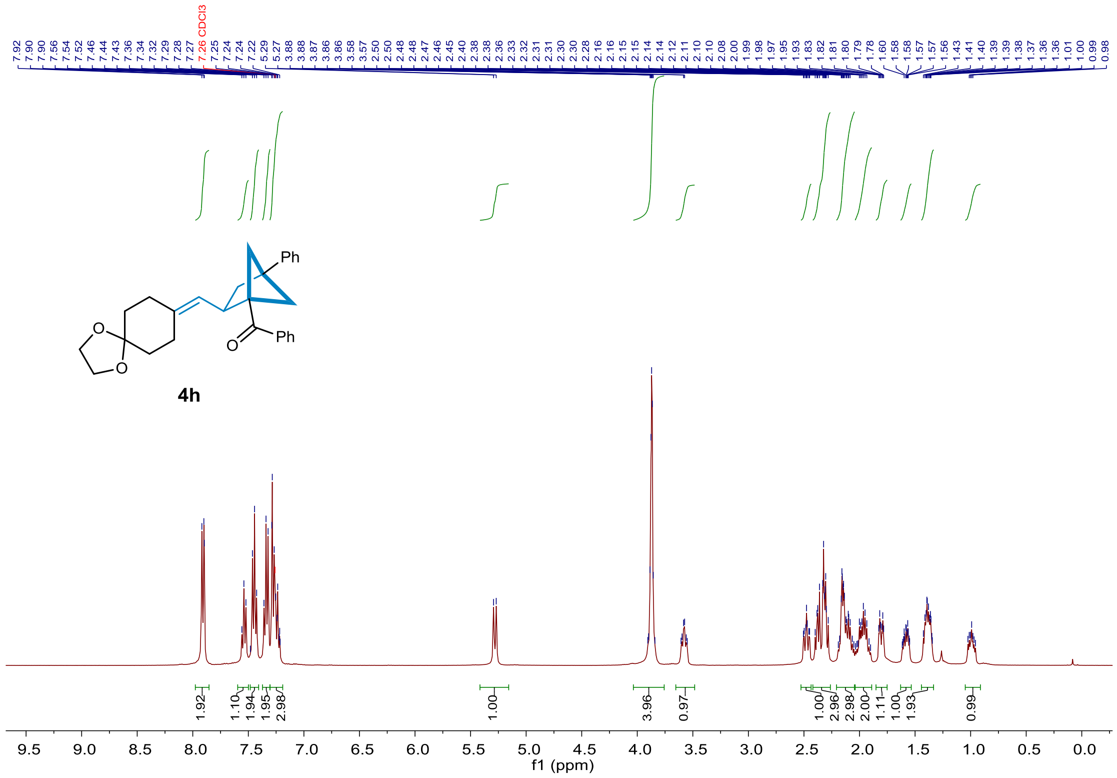

Supplementary Figure 91. **^{1}H NMR of the 4h** (400 MHz, $CDCl_3$)

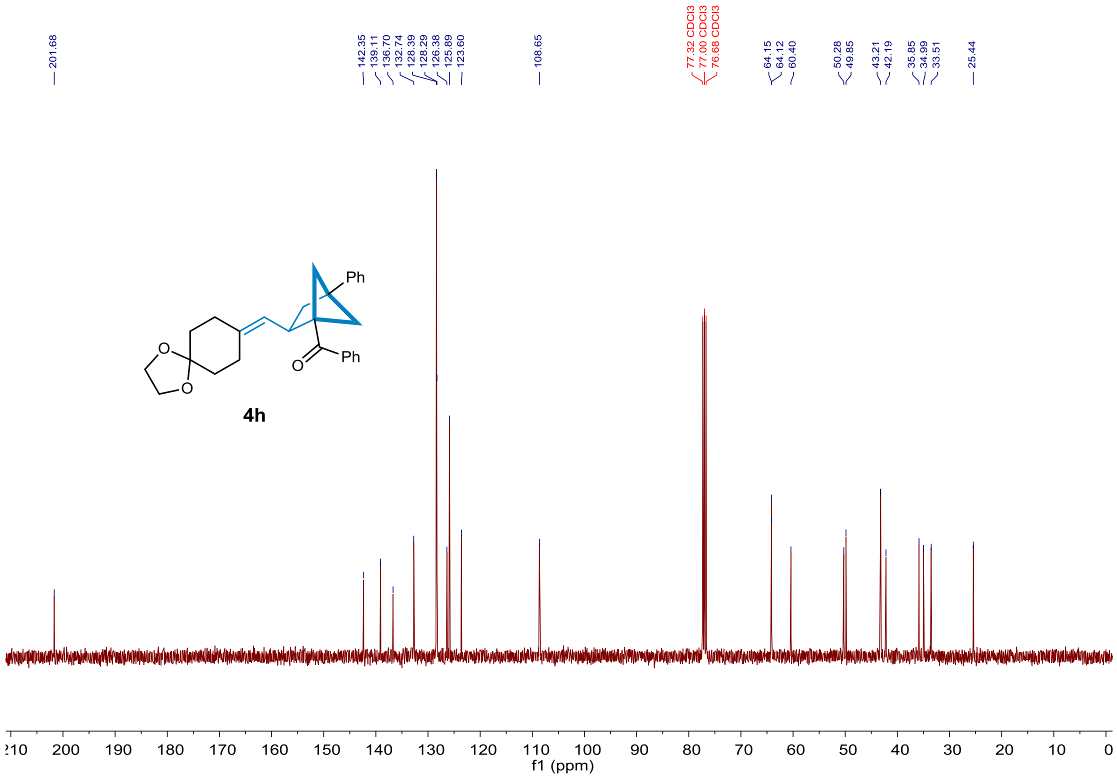

Supplementary Figure 92. **^{13}C NMR of the 4h** (101 MHz, $CDCl_3$)

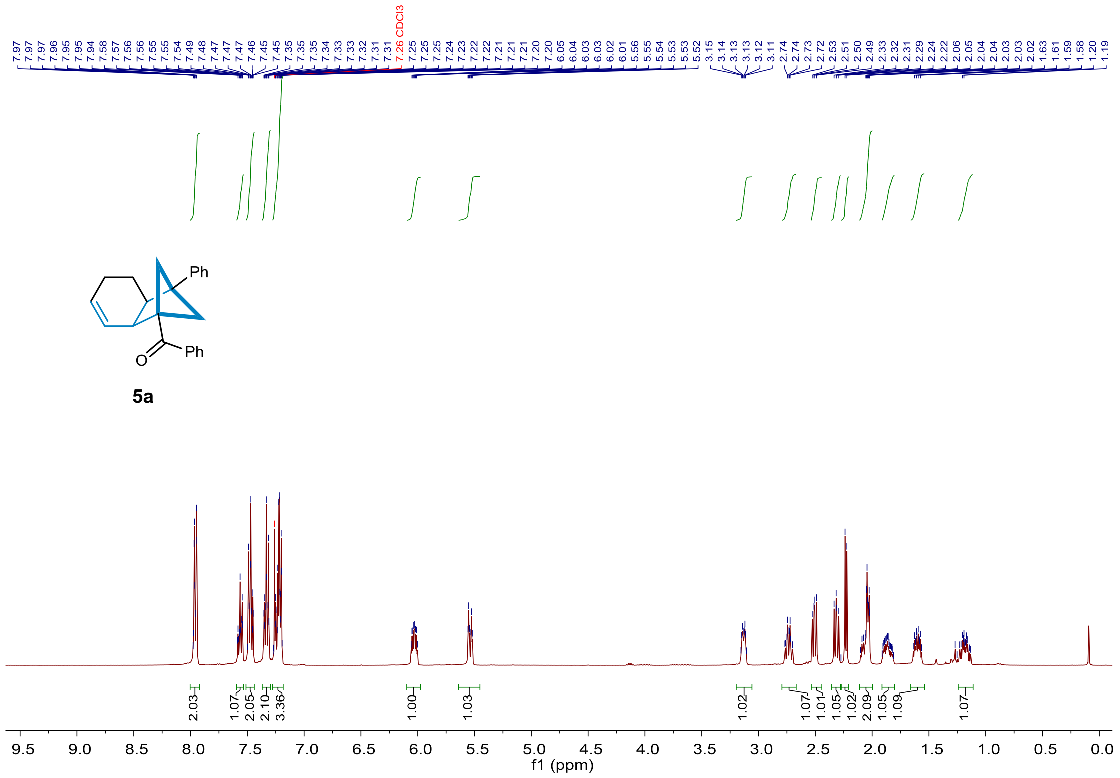

Supplementary Figure 93. ^{1}H NMR of the 5a (400 MHz, $CDCl_3$)

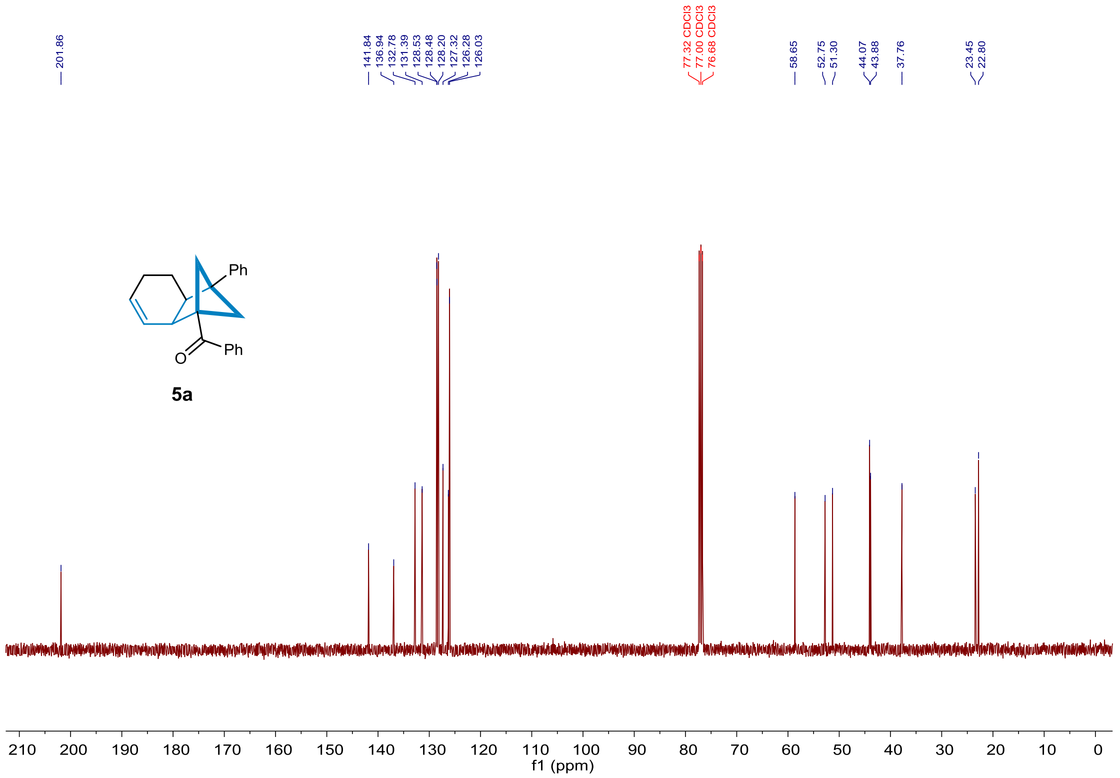

Supplementary Figure 94. ^{13}C NMR of the 5a (101 MHz, $CDCl_3$)

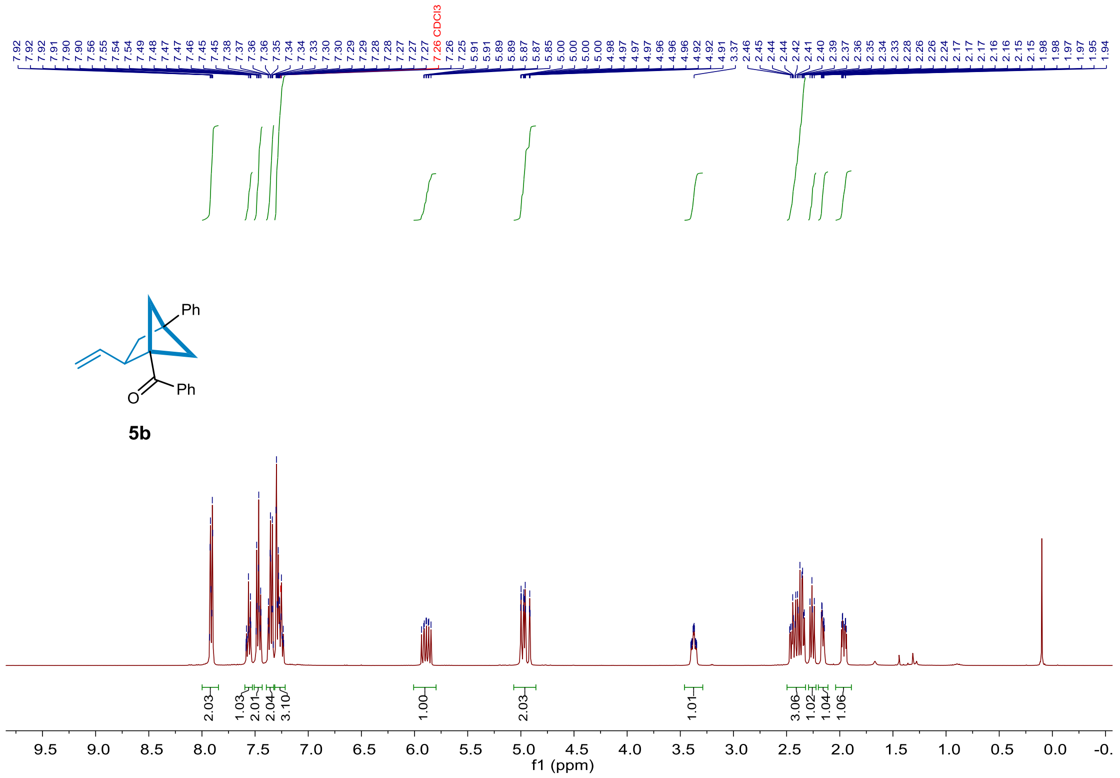

Supplementary Figure 95. **^{1}H NMR of the 5b** (400 MHz, $CDCl_3$)

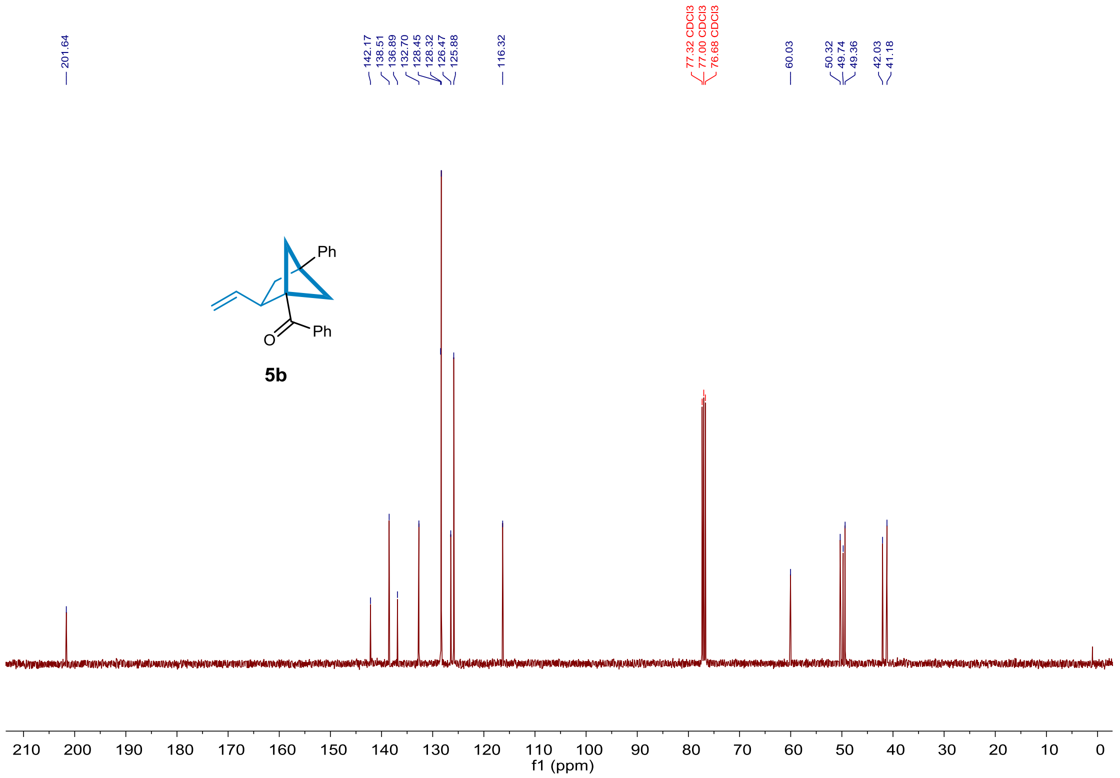

Supplementary Figure 96. **^{13}C NMR of the 5b** (101 MHz, $CDCl_3$)

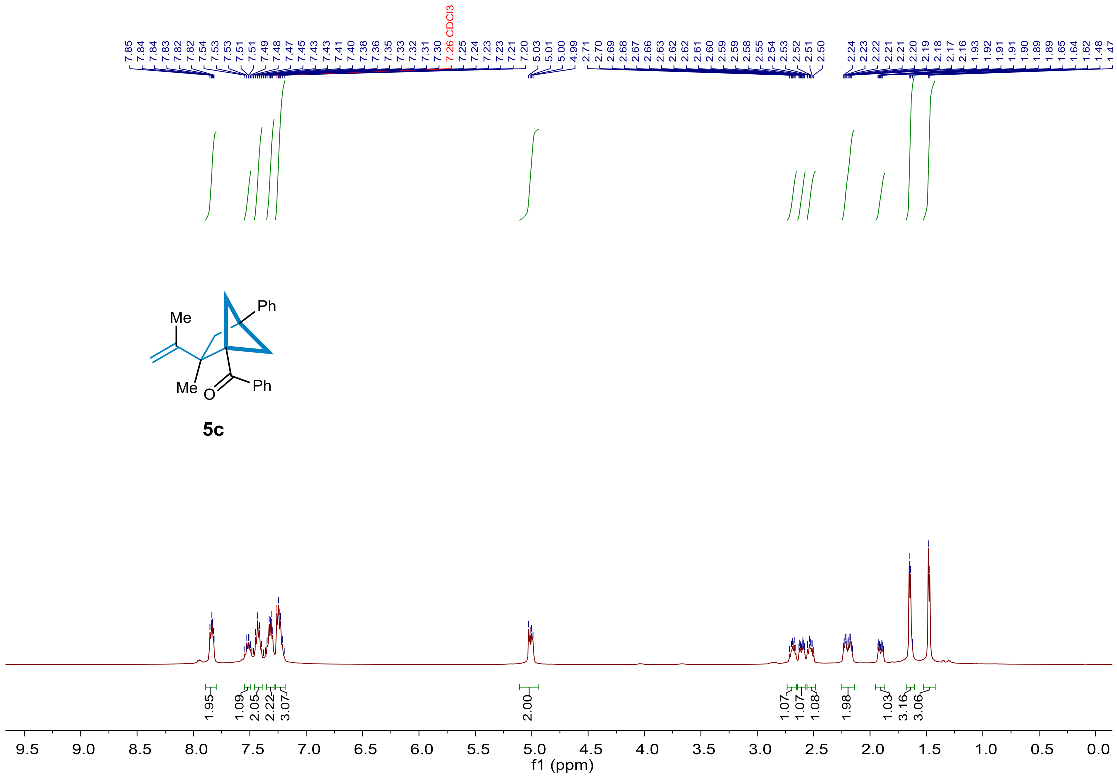

Supplementary Figure 97. **^{1}H NMR of the 5c** (400 MHz, $CDCl_3$)

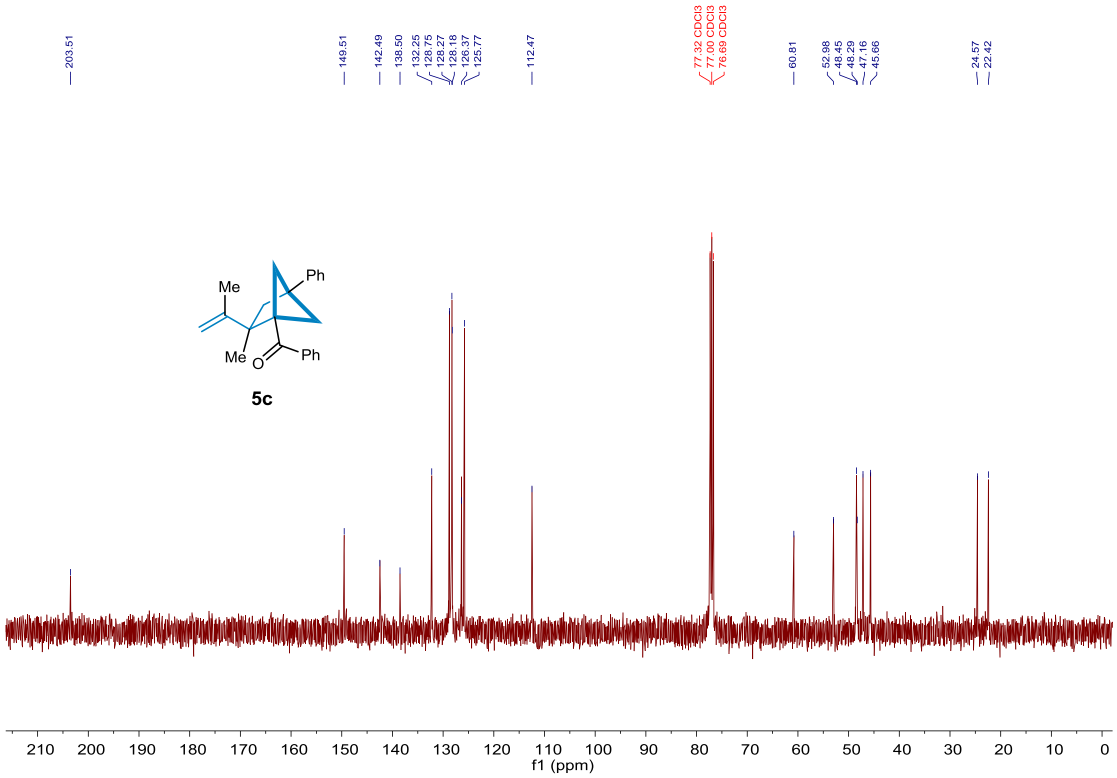

Supplementary Figure 98. **^{13}C NMR of the 5c** (101 MHz, $CDCl_3$)

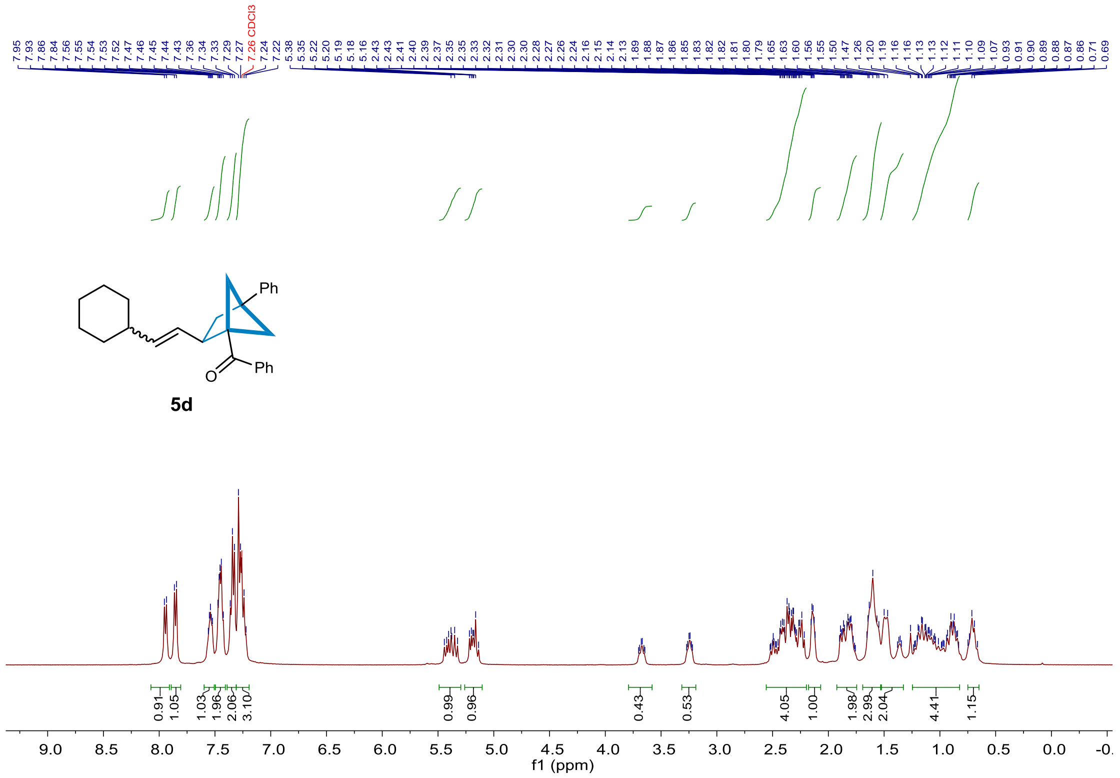

Supplementary Figure 99. **^{1}H NMR of the 5d** (400 MHz, $CDCl_3$)

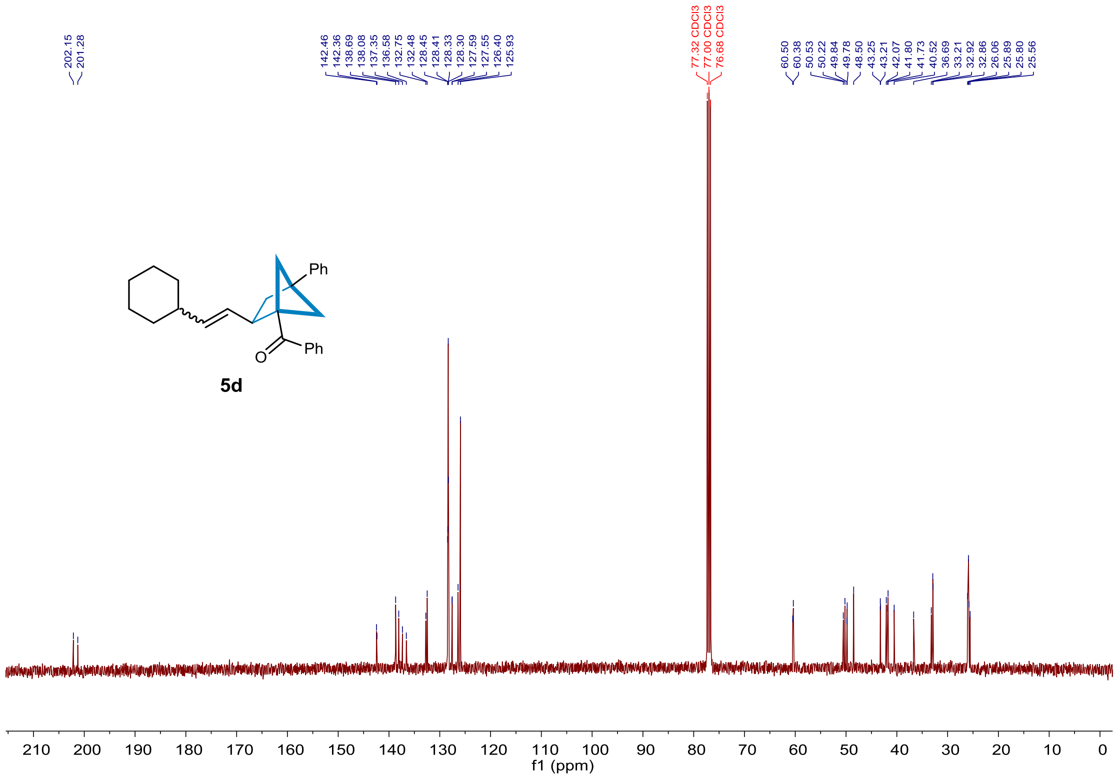

Supplementary Figure 100. **^{13}C NMR of the 5d** (101 MHz, $CDCl_3$)

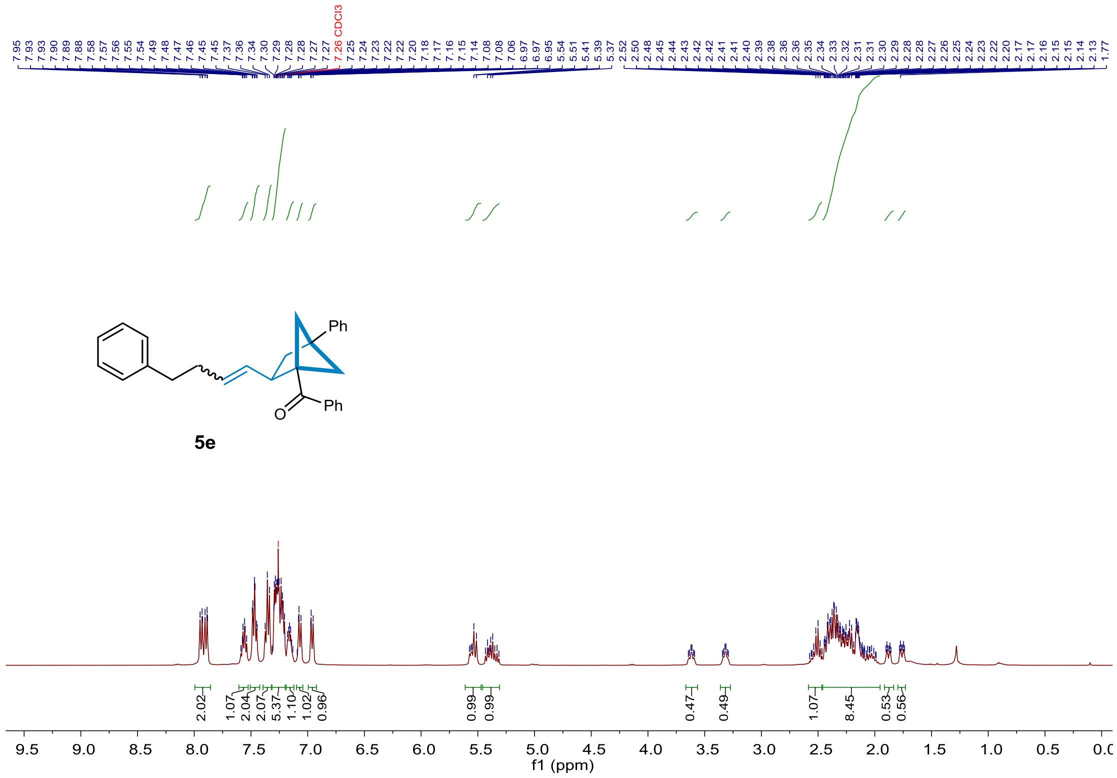

Supplementary Figure 101. **^{1}H NMR of the 5e** (400 MHz, $CDCl_3$)

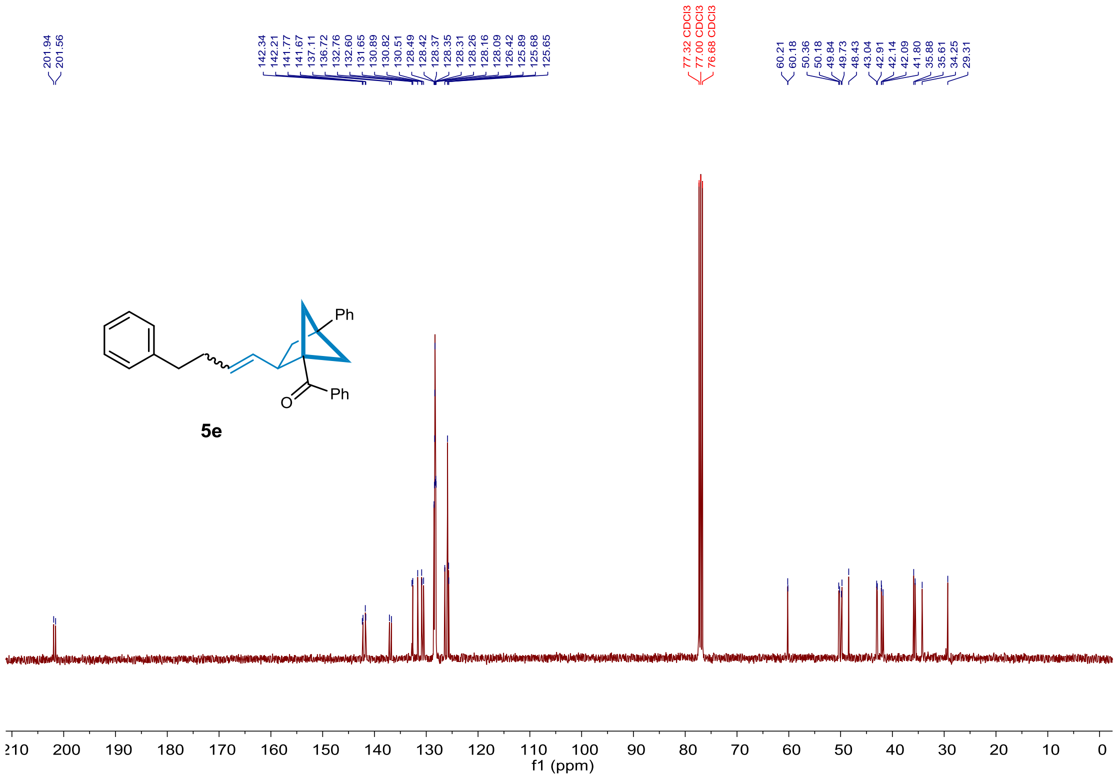

Supplementary Figure 102. **^{13}C NMR of the 5e** (101 MHz, $CDCl_3$)

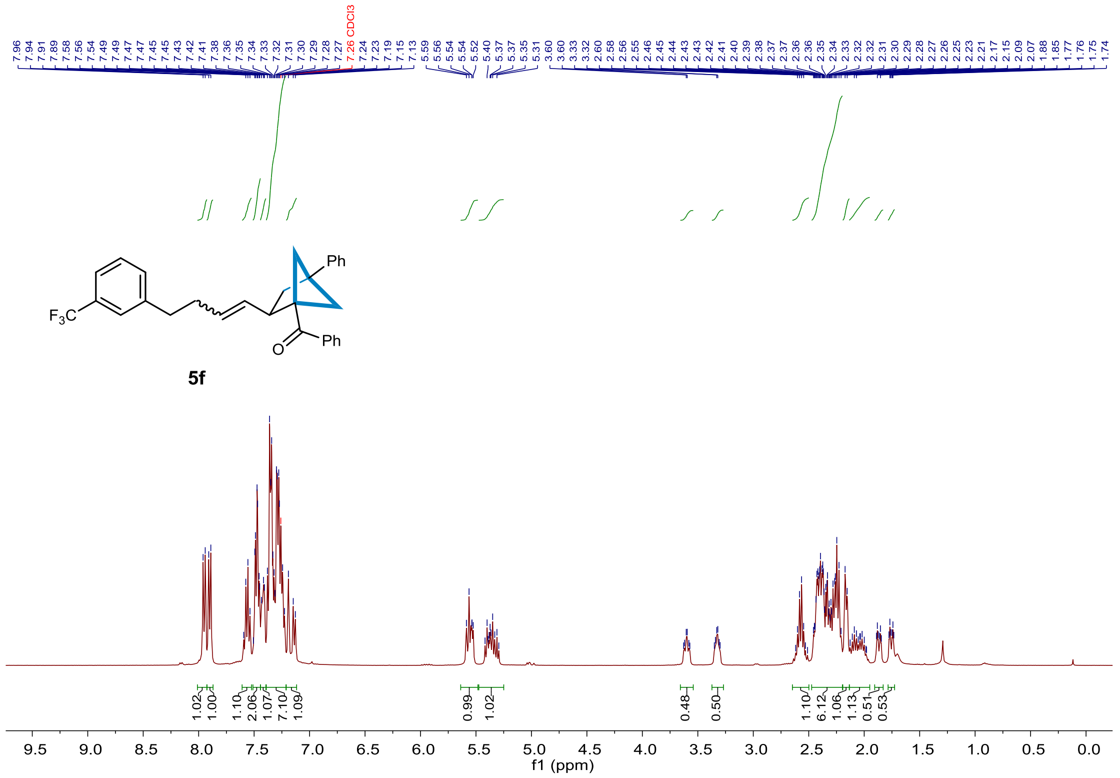

Supplementary Figure 103. ^{1}**H NMR of the 5f** (400 MHz, $CDCl_3$)

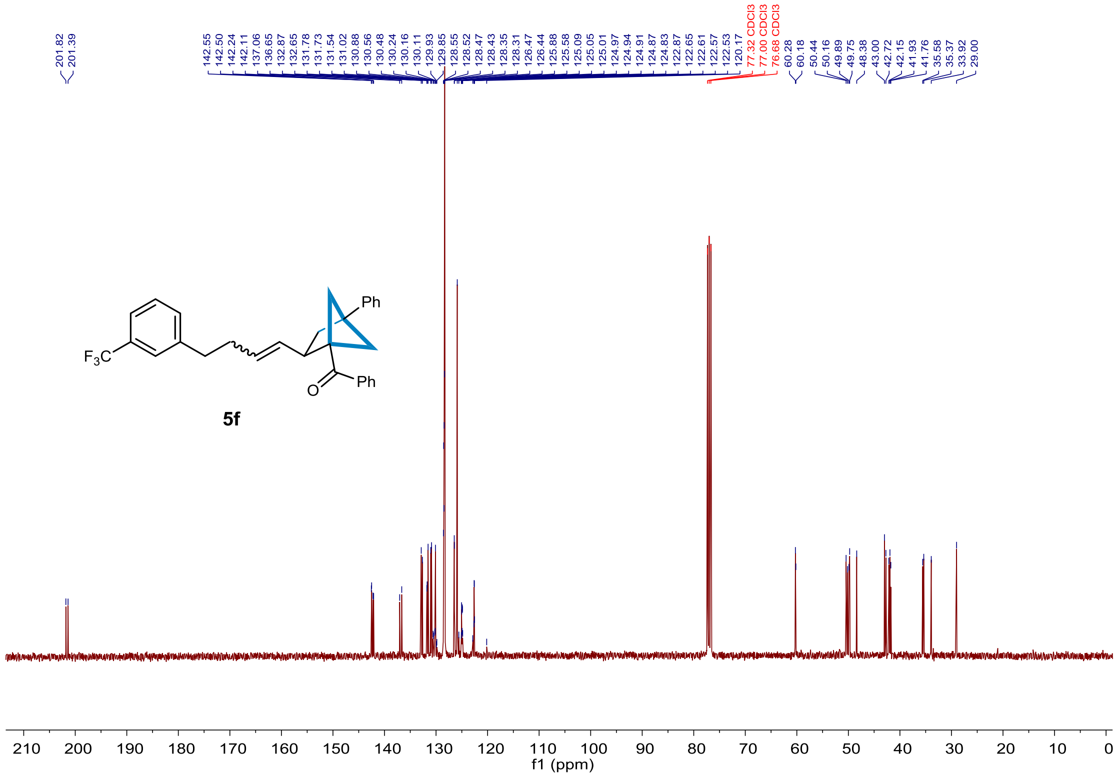

Supplementary Figure 104. ^{13}**C NMR of the 5f** (101 MHz, $CDCl_3$)

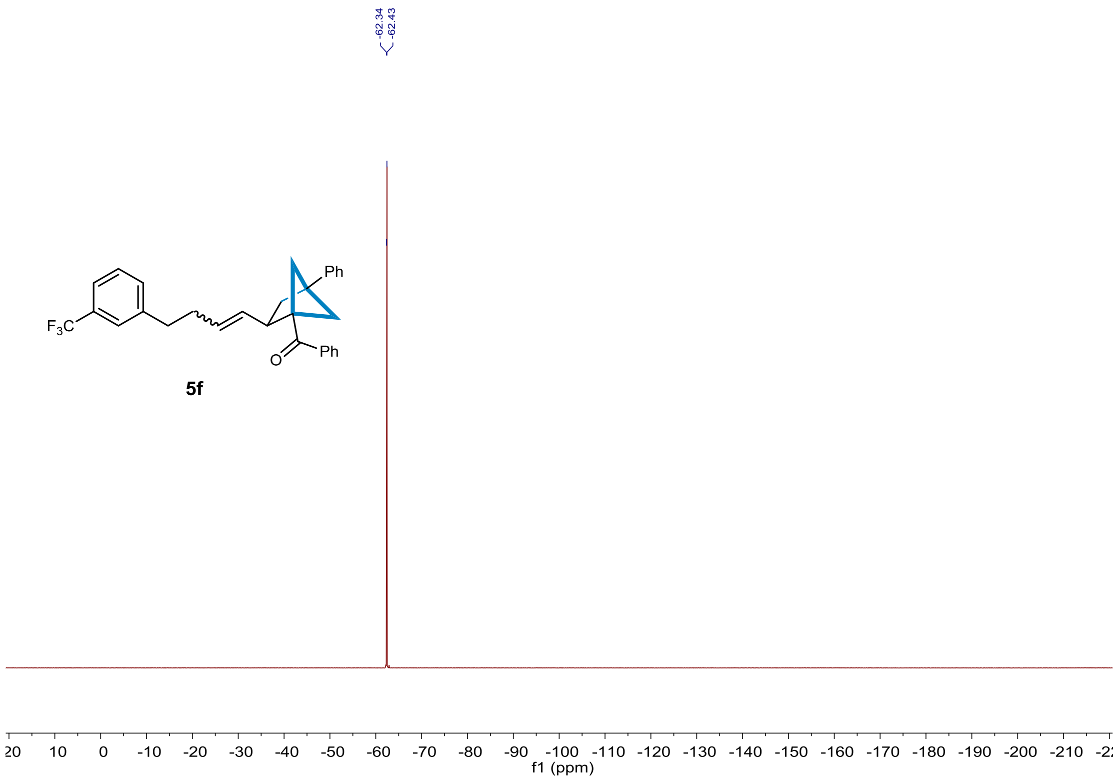

Supplementary Figure 105. **^{19}F NMR of the 5f** (377 MHz, $CDCl_3$)

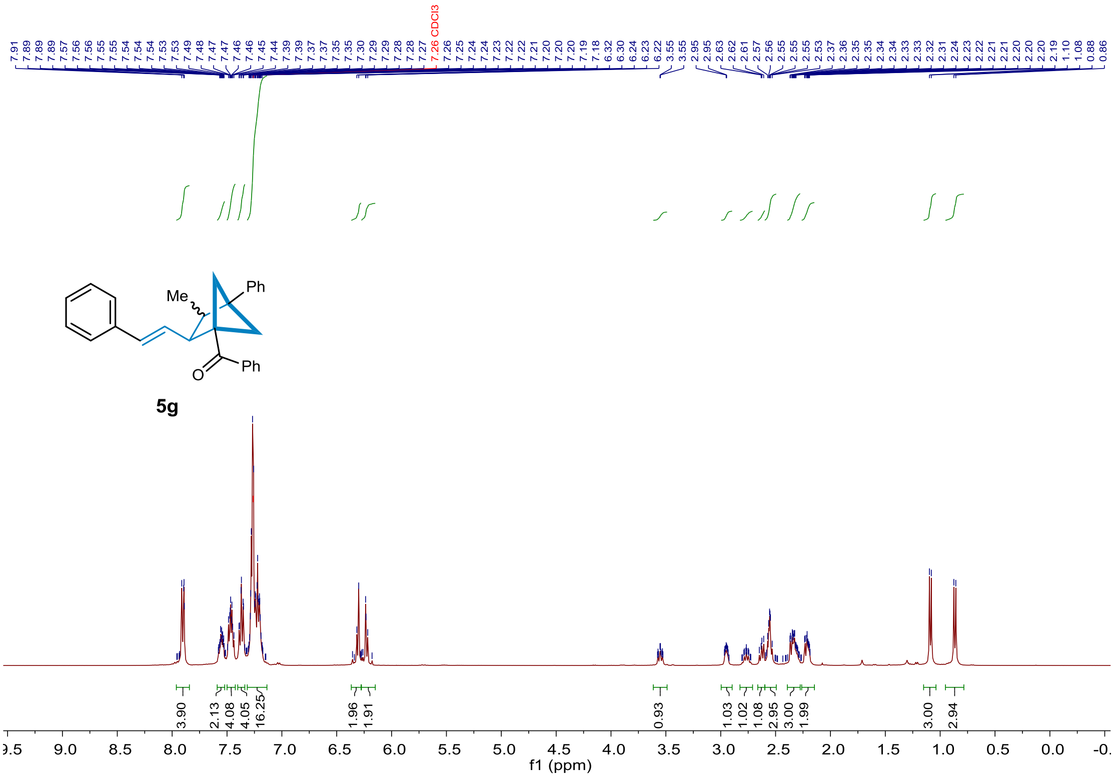

Supplementary Figure 106. **^{1}H NMR of the 5g** (400 MHz, $CDCl_3$)

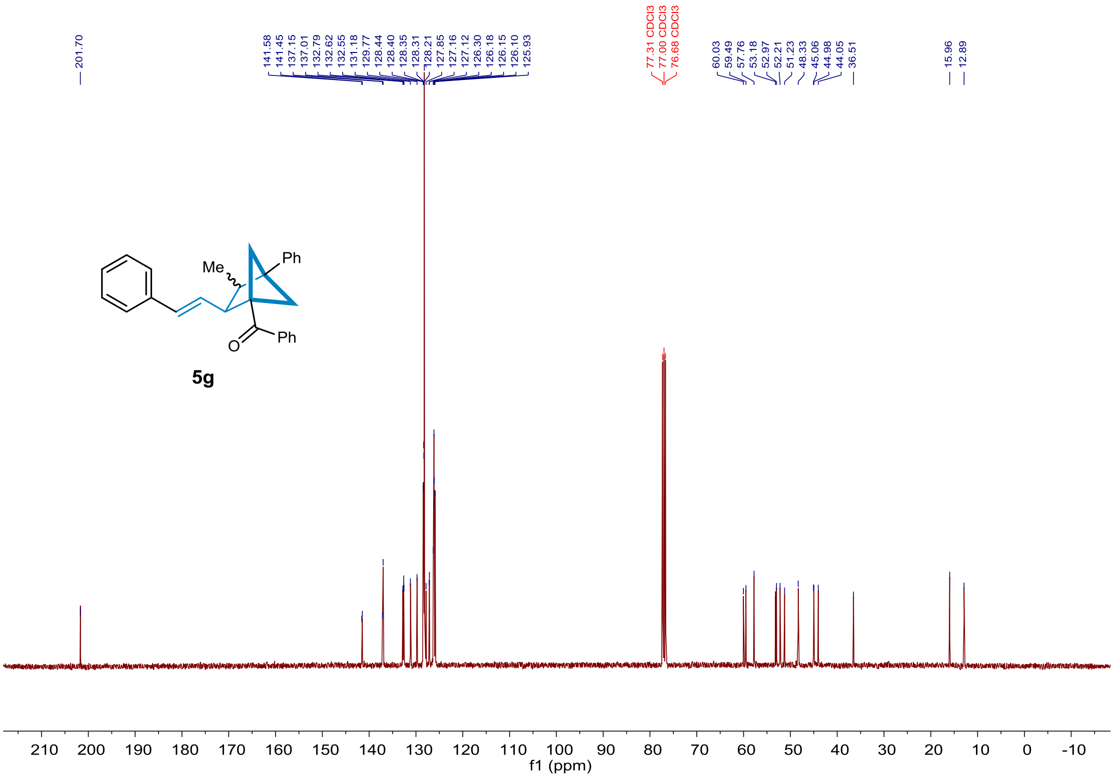

Supplementary Figure 107. **^{13}C NMR of the 5g** (101 MHz, $CDCl_3$)

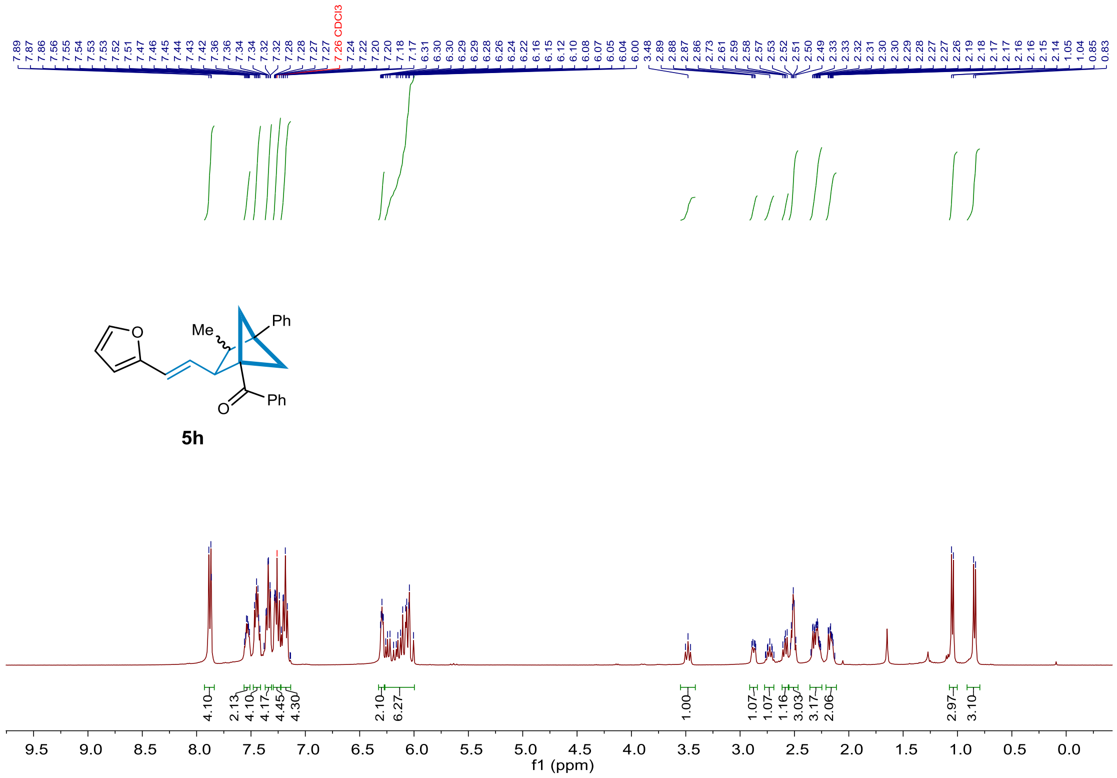

Supplementary Figure 108. **^{1}H NMR of the 5h** (400 MHz, $CDCl_3$)

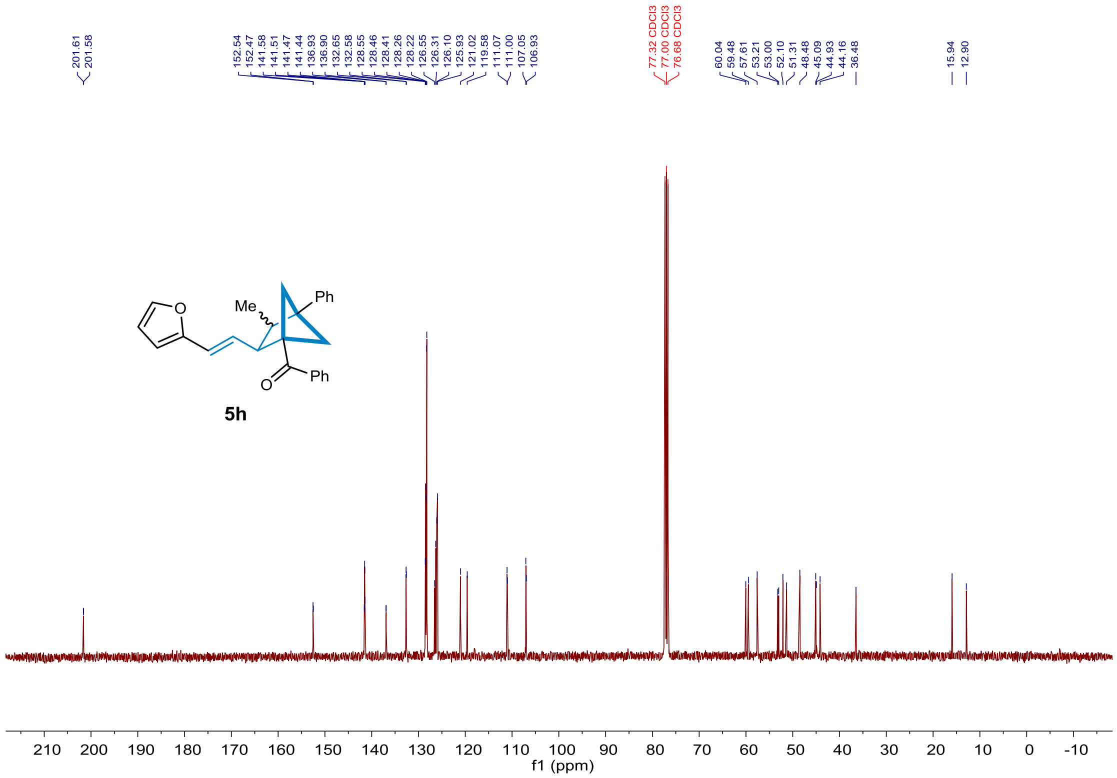

Supplementary Figure 109. **^{13}C NMR of the 5h** (101 MHz, $CDCl_3$)

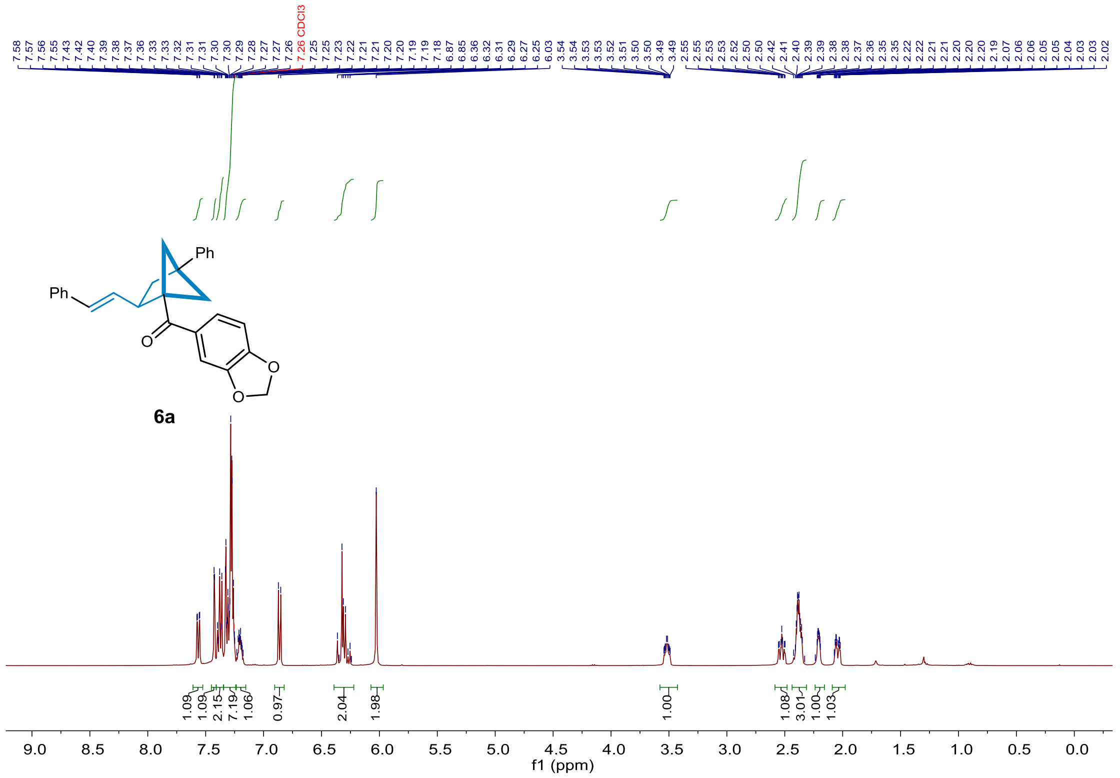

Supplementary Figure 110. ^{1}**H NMR of the 6a** (400 MHz, $CDCl_3$)

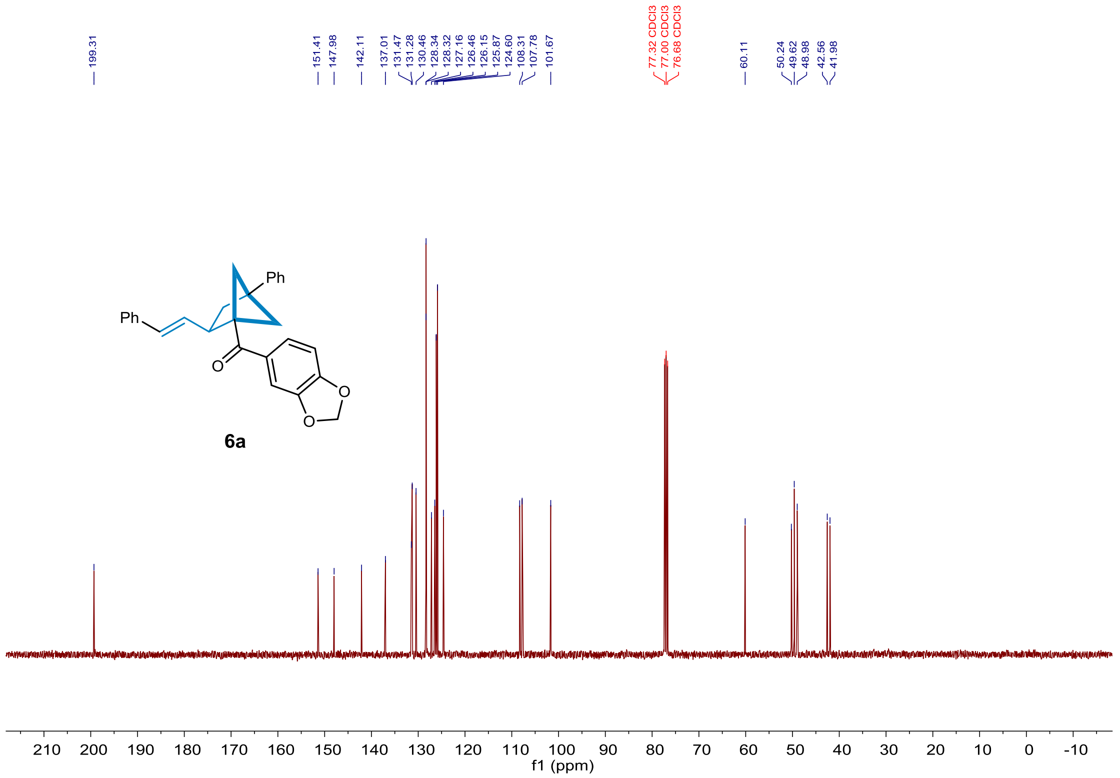

Supplementary Figure 111. ^{13}**C NMR of the 6a** (101 MHz, $CDCl_3$)

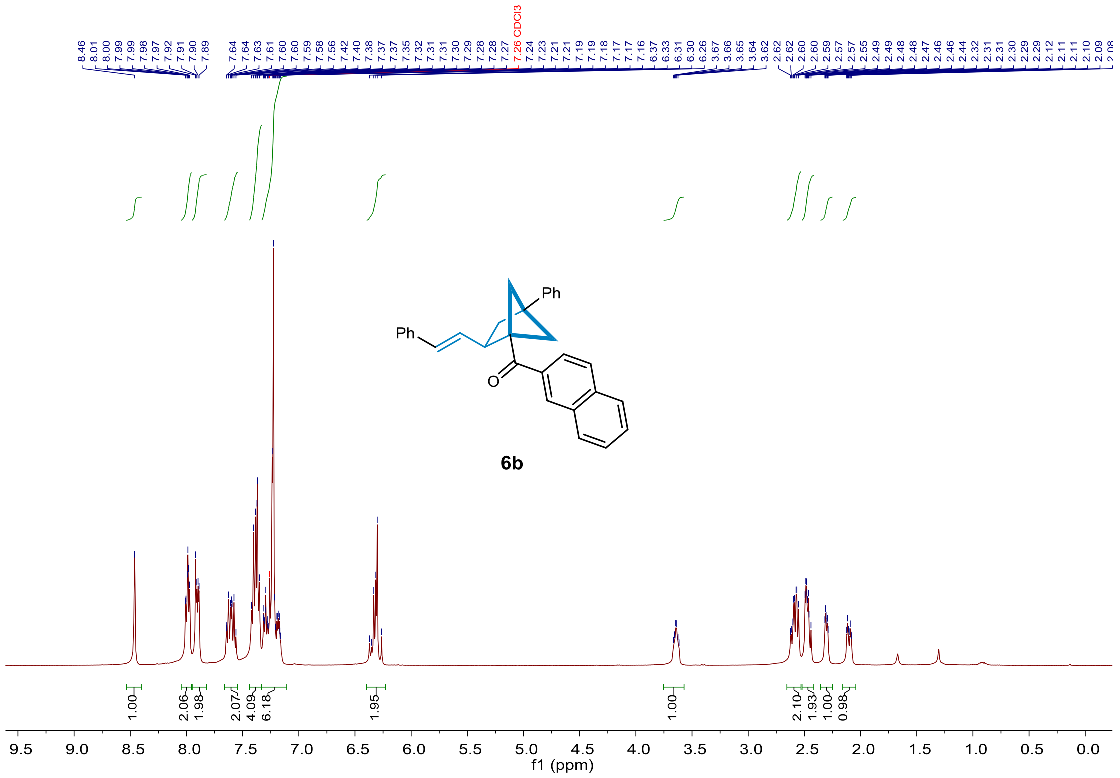

Supplementary Figure 112. **^{1}H NMR of the 6b** (400 MHz, $CDCl_3$)

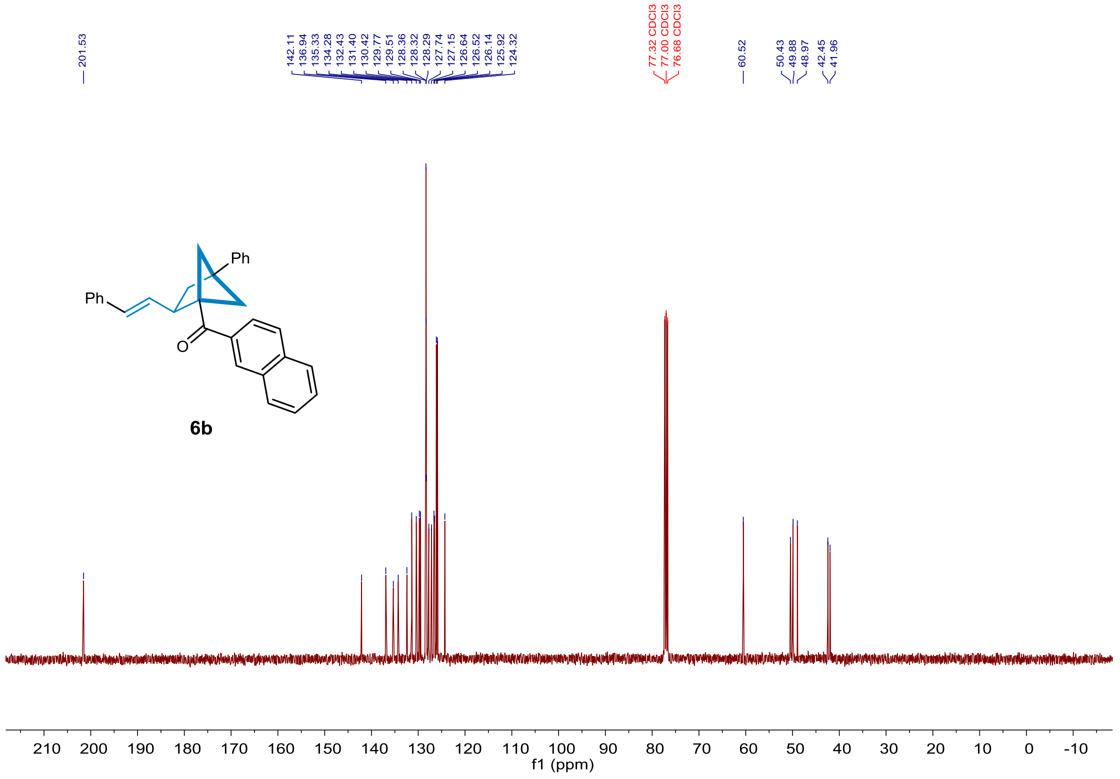

Supplementary Figure 113. **^{13}C NMR of the 6b** (101 MHz, $CDCl_3$)

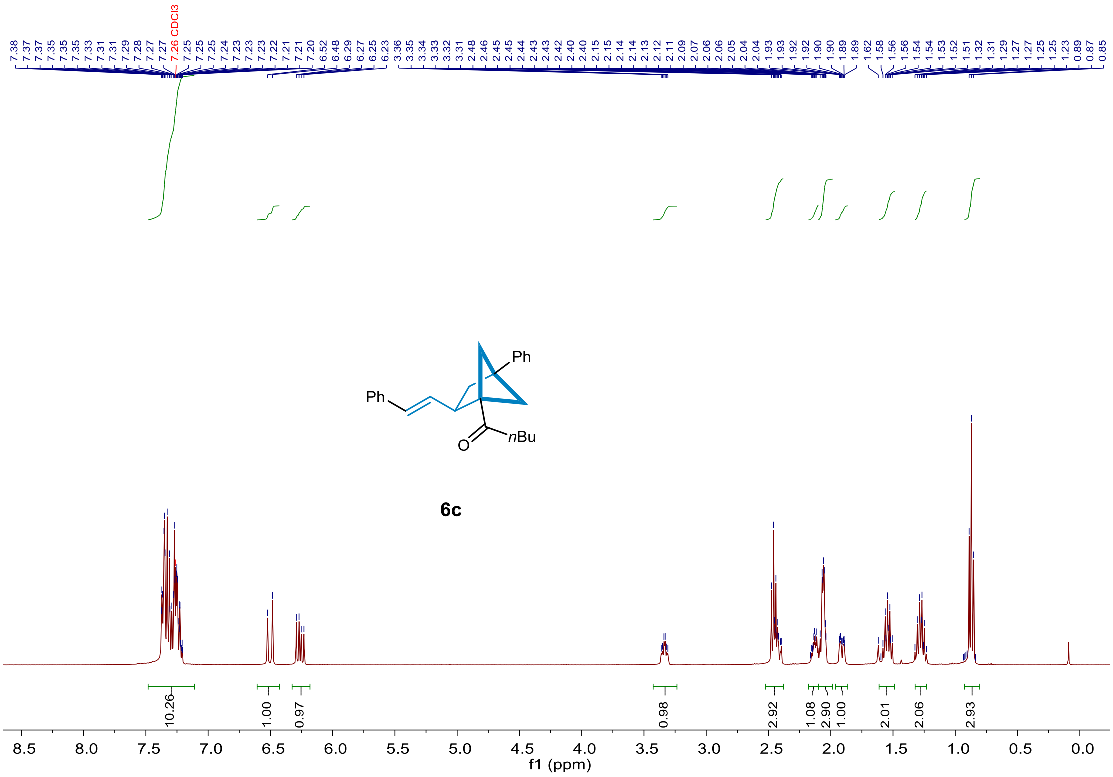

Supplementary Figure 114. ^{1}**H NMR of the 6c** (400 MHz, $CDCl_3$)

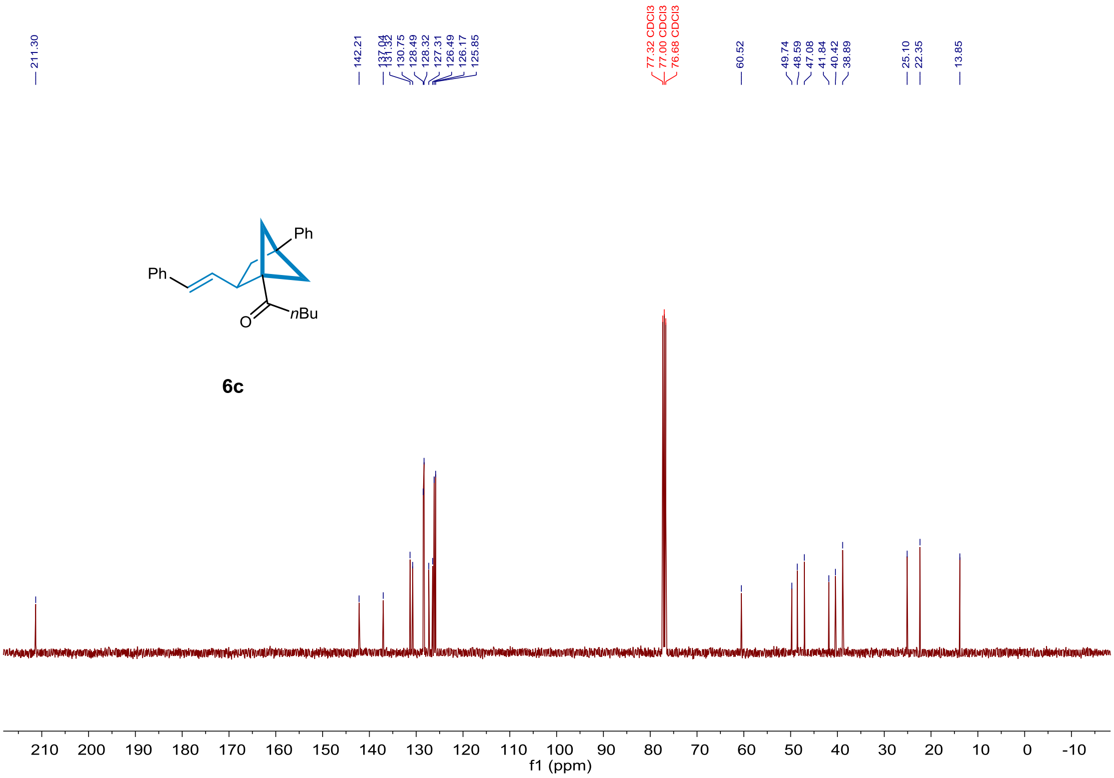

Supplementary Figure 115. ^{13}**C NMR of the 6c** (101 MHz, $CDCl_3$)

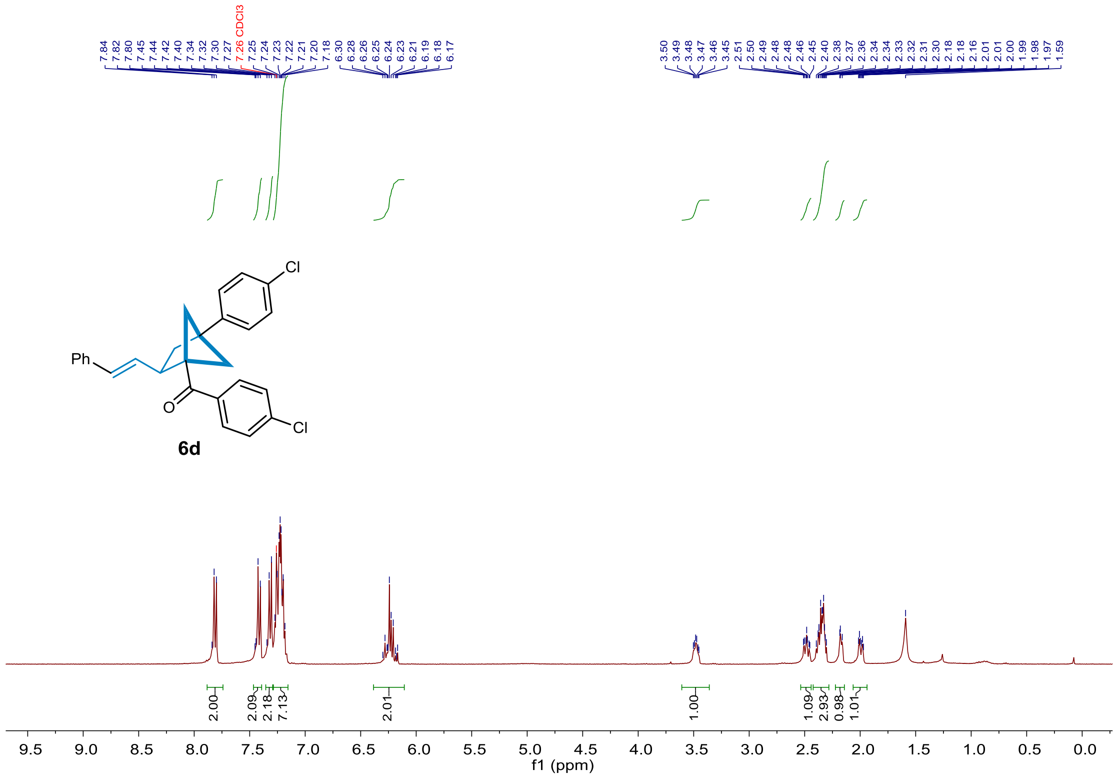

Supplementary Figure 116. **^{1}H NMR of the 6d** (400 MHz, $CDCl_3$)

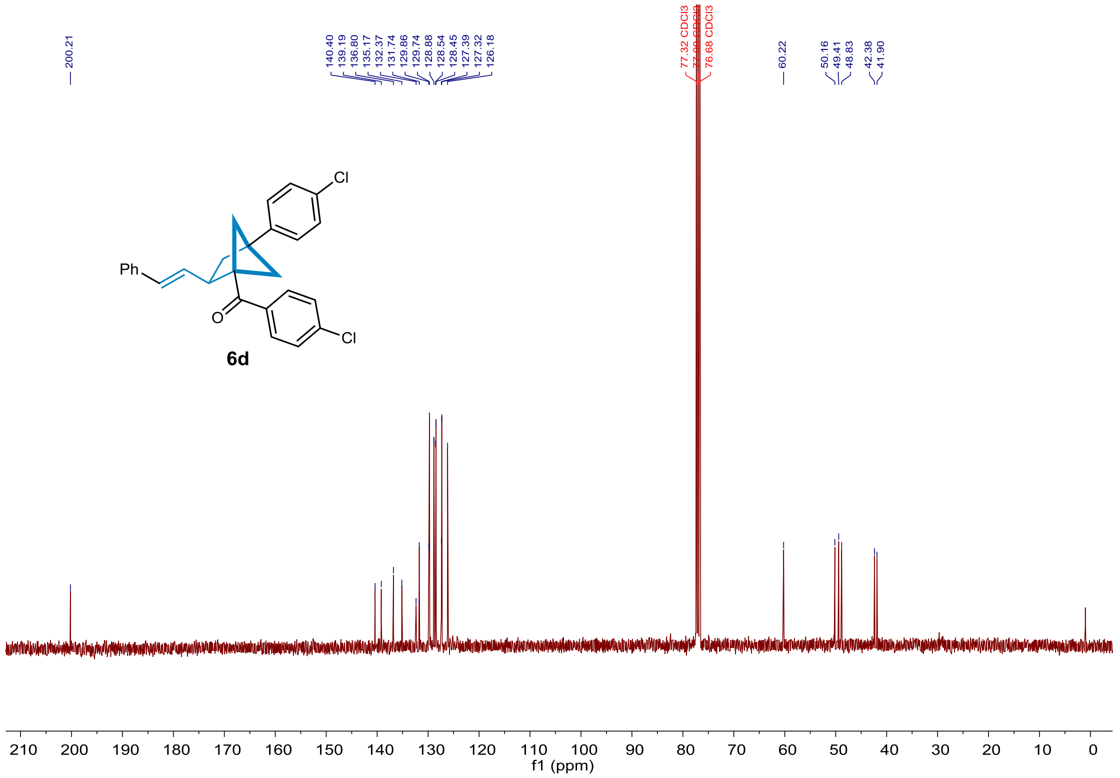

Supplementary Figure 117. **^{13}C NMR of the 6d** (101 MHz, $CDCl_3$)

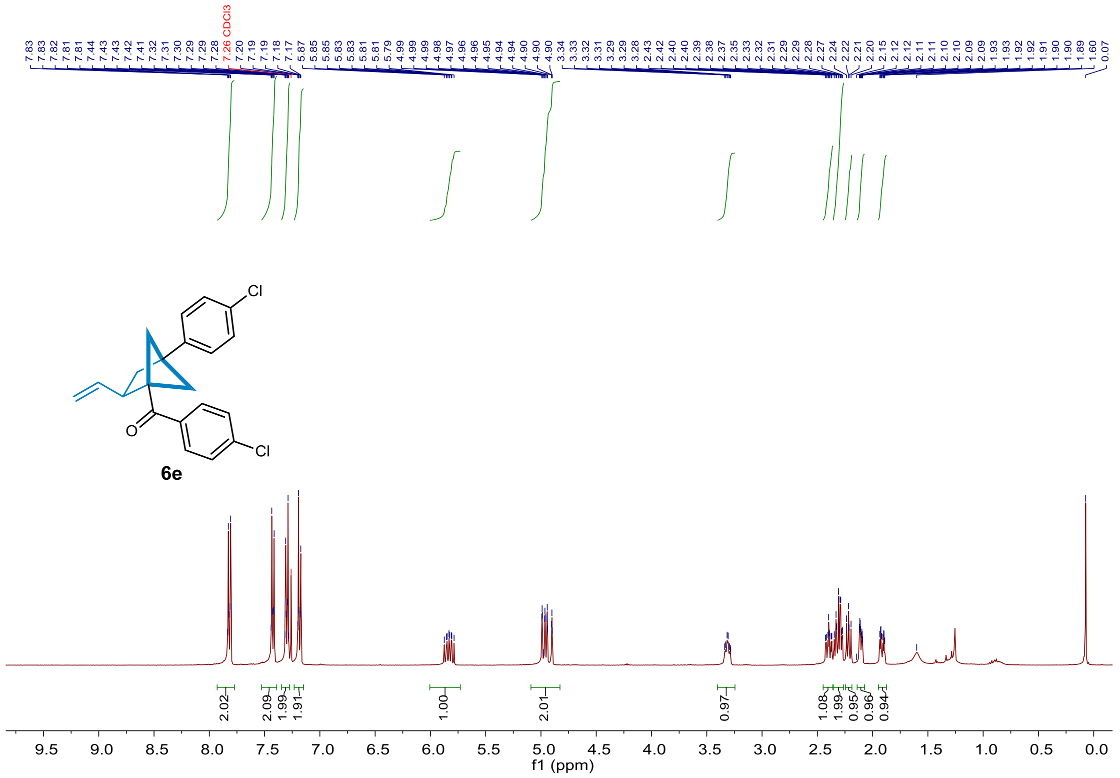

Supplementary Figure 118. ^{1}**H NMR of the 6e** (400 MHz, $CDCl_3$)

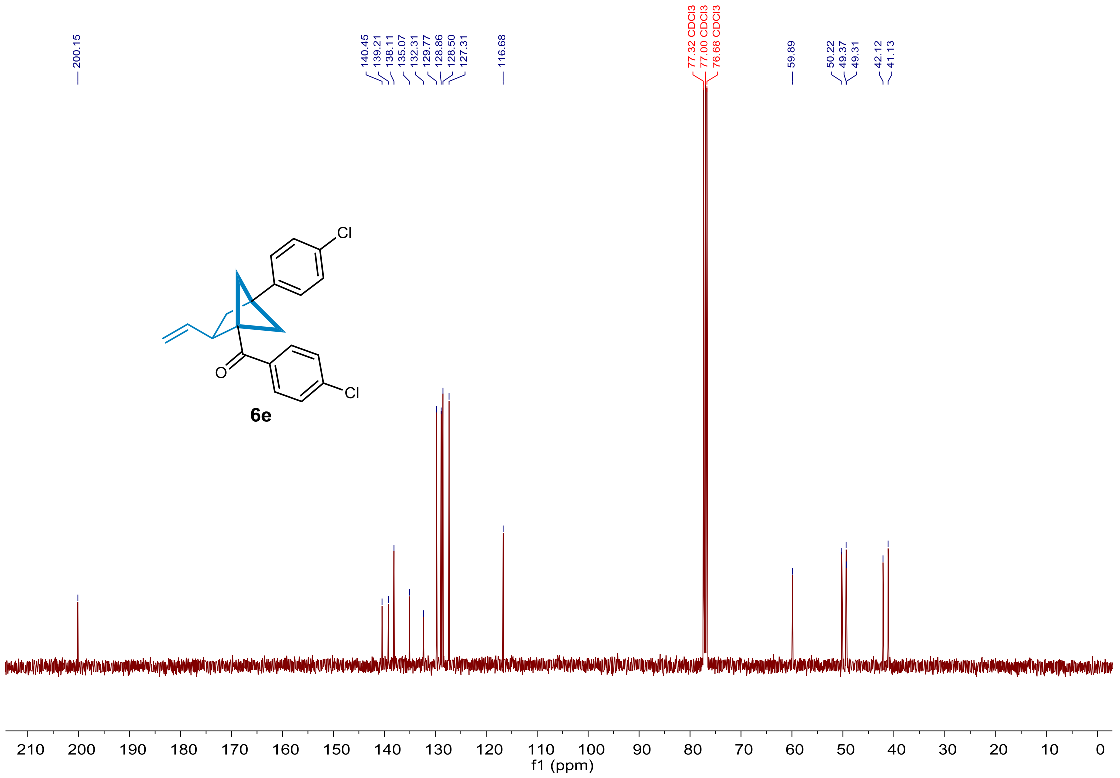

Supplementary Figure 119. ^{13}**C NMR of the 6e** (101 MHz, $CDCl_3$)

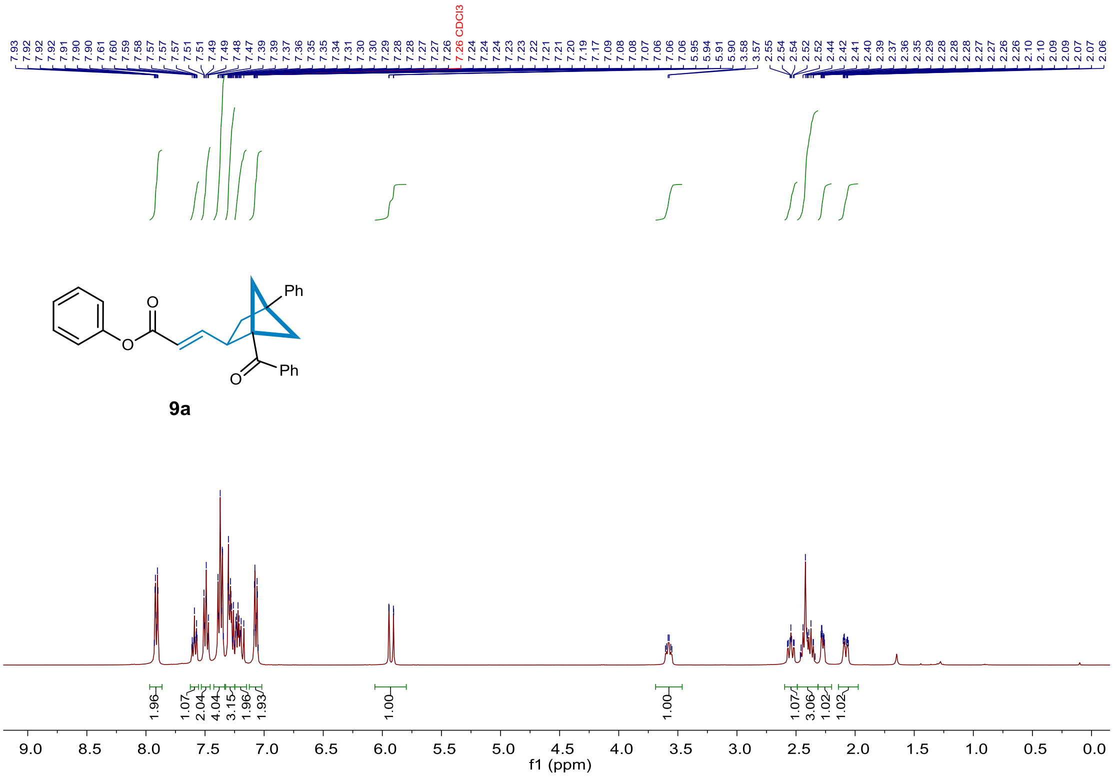

Supplementary Figure 120. **^{1}H NMR of the 9a** (400 MHz, $CDCl_3$)

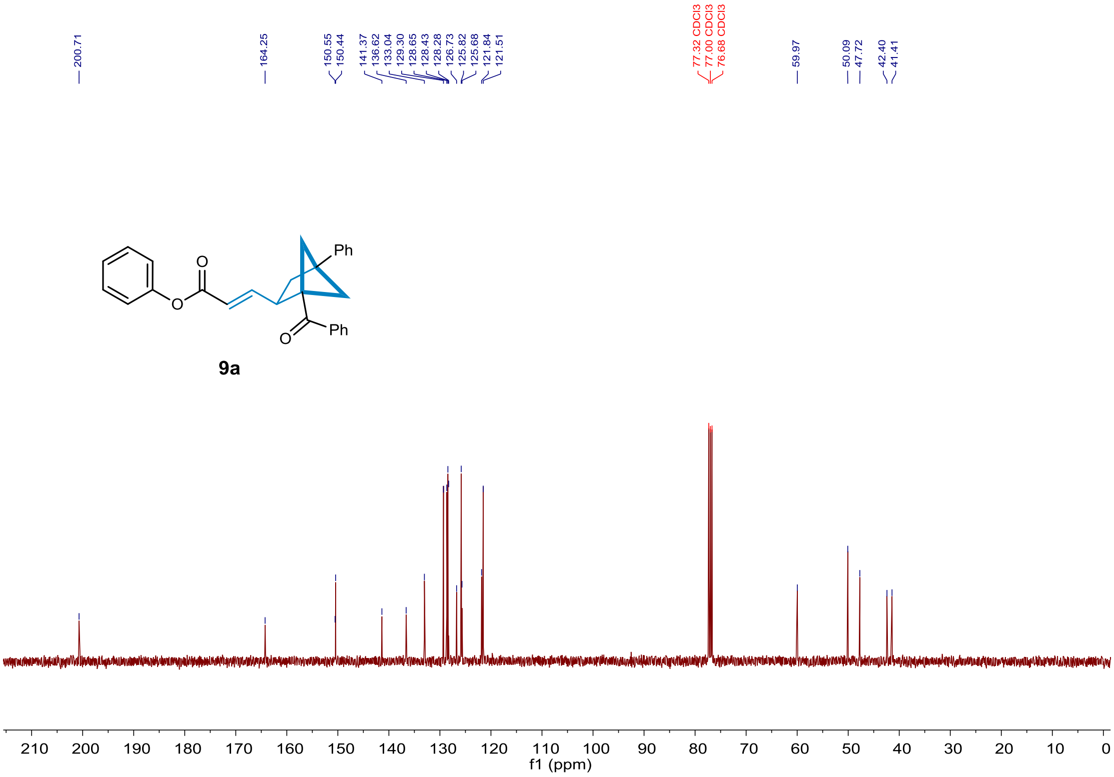

Supplementary Figure 121. **^{13}C NMR of the 9a** (101 MHz, $CDCl_3$)

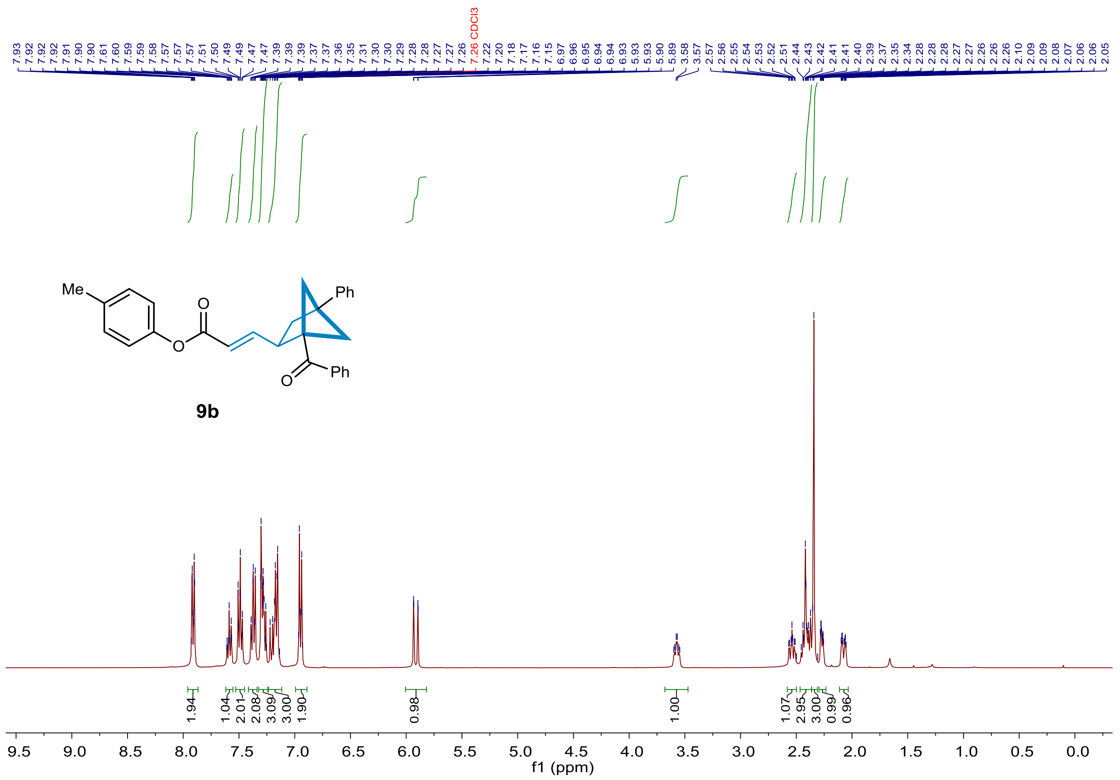

Supplementary Figure 122. ^{1}**H NMR of the 9b** (400 MHz, $CDCl_3$)

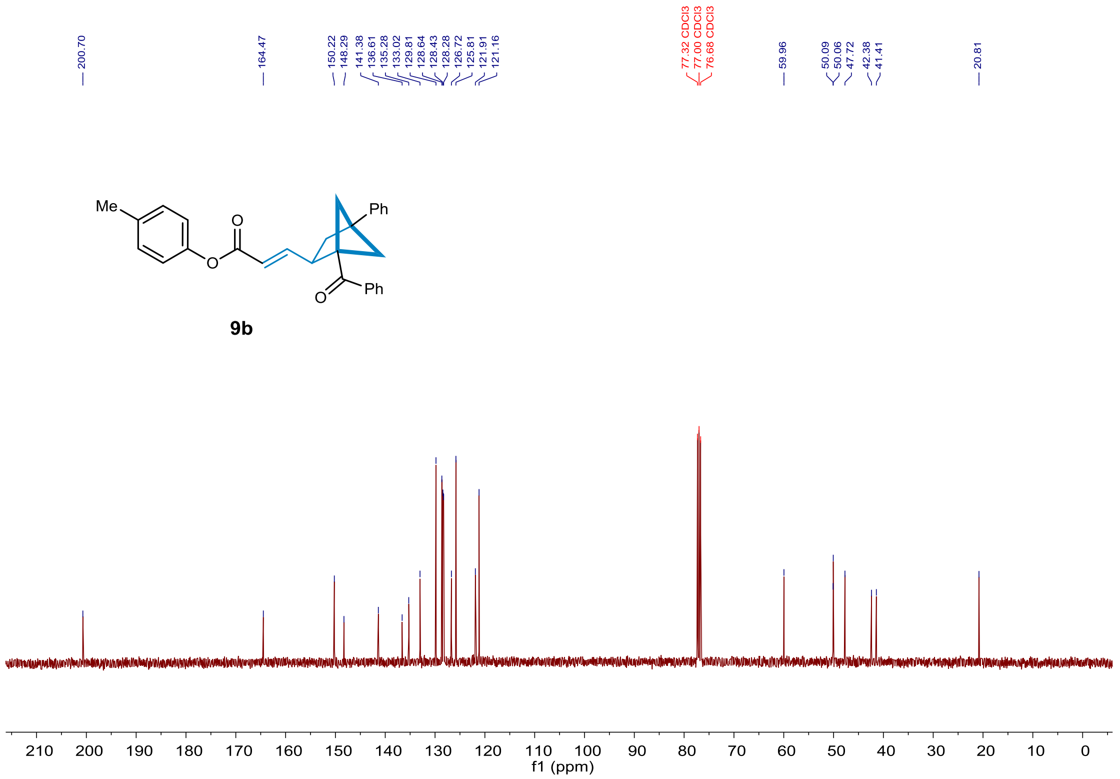

Supplementary Figure 123. ^{13}**C NMR of the 9b** (101 MHz, $CDCl_3$)

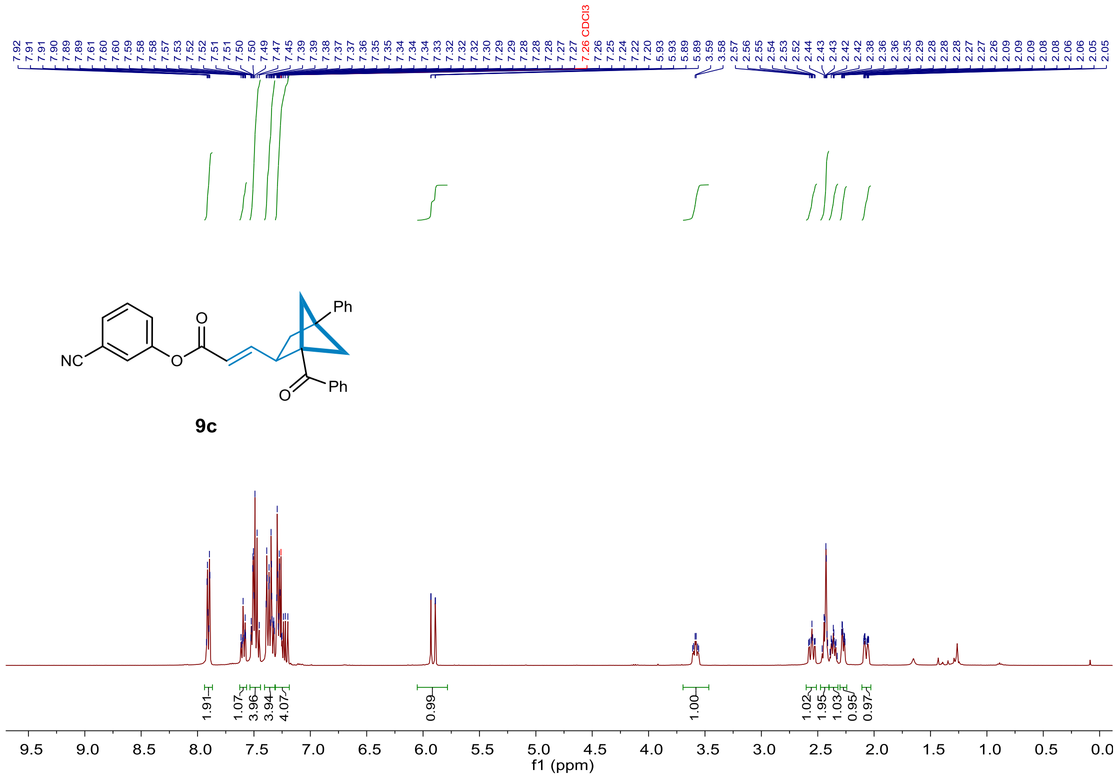

Supplementary Figure 124. **^{1}H NMR of the 9c** (400 MHz, $CDCl_3$)

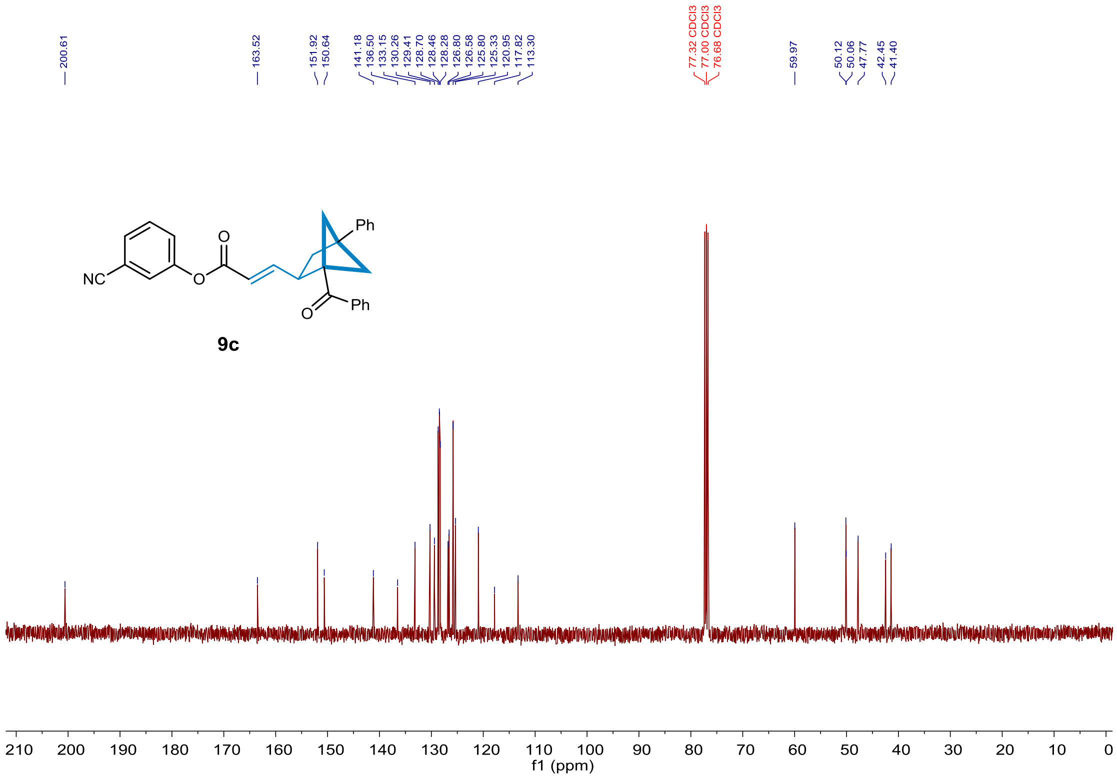

Supplementary Figure 125. **^{13}C NMR of the 9c** (101 MHz, $CDCl_3$)

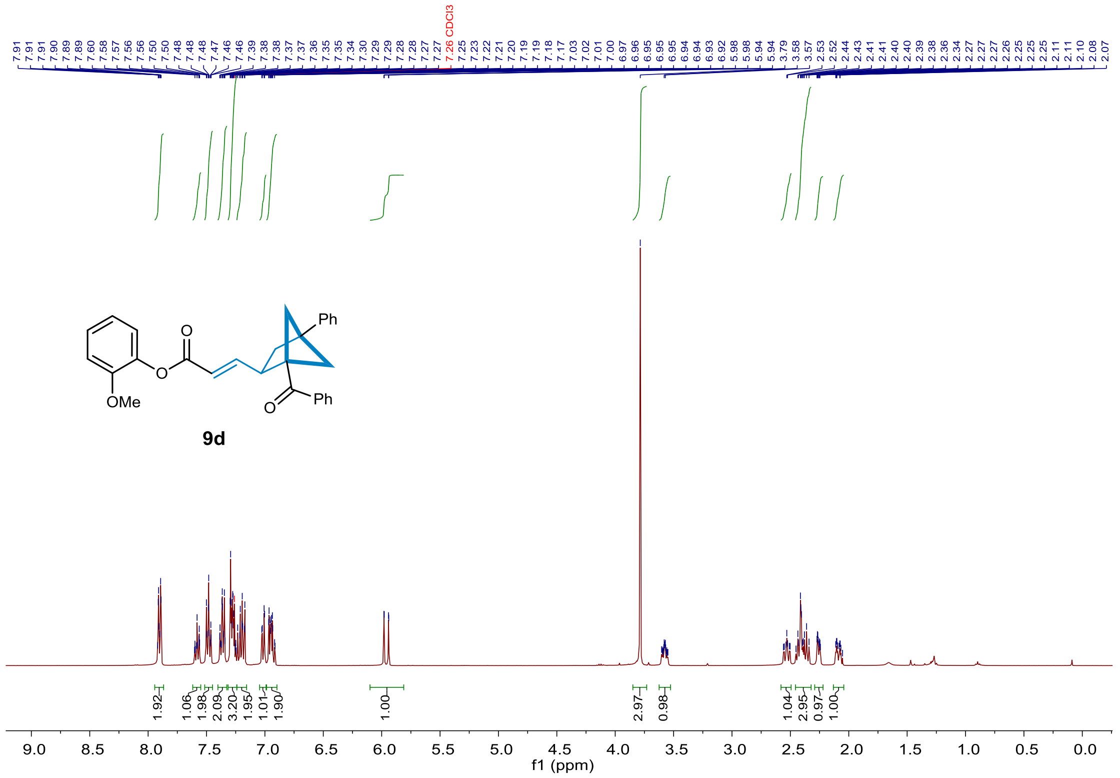

Supplementary Figure 126. **^{1}H NMR of the 9d** (400 MHz, $CDCl_3$)

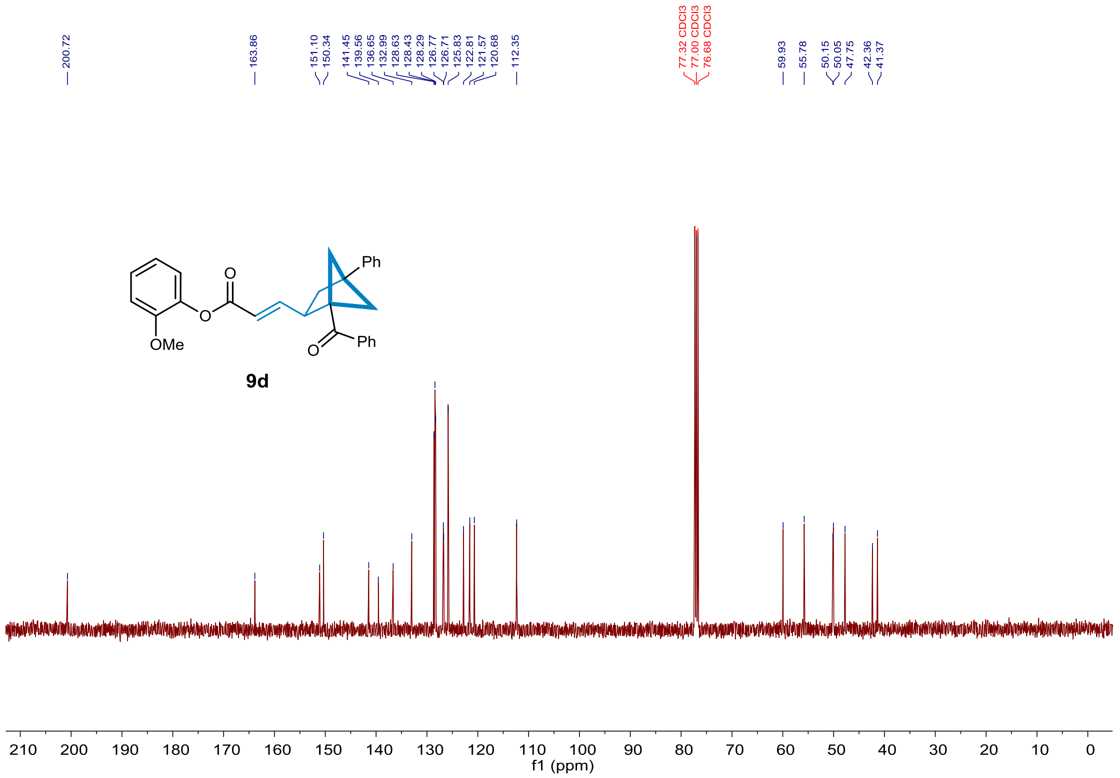

Supplementary Figure 127. **^{13}C NMR of the 9d** (101 MHz, $CDCl_3$)

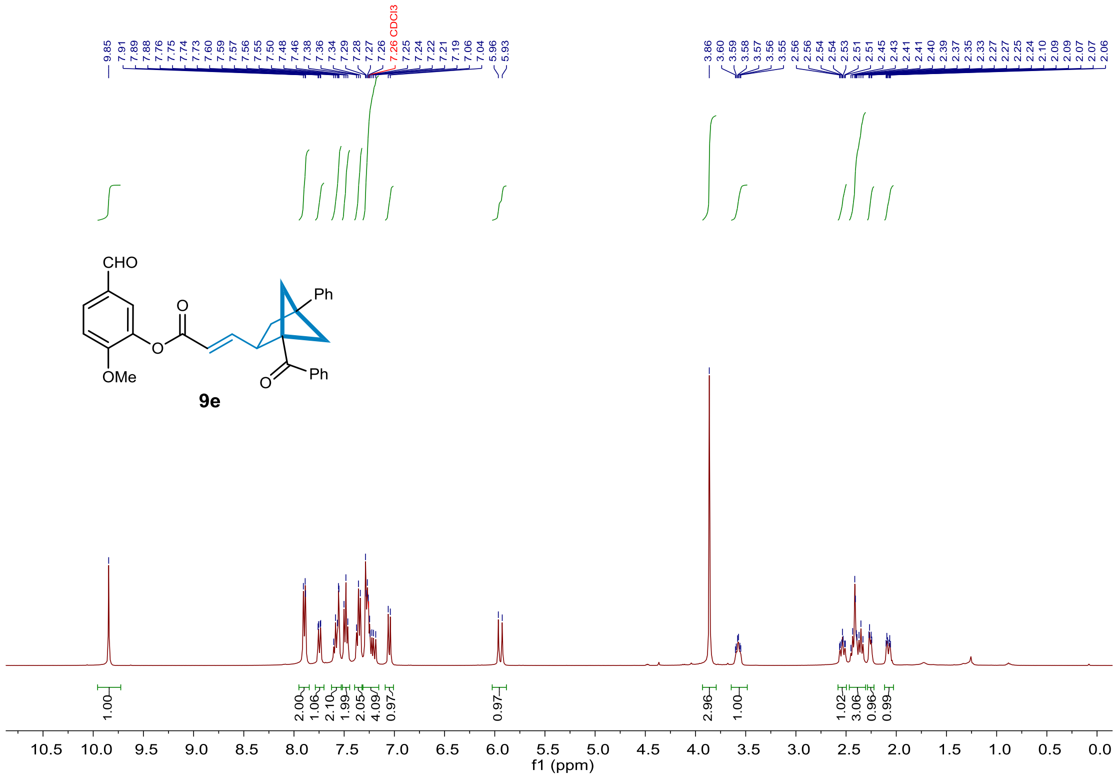

Supplementary Figure 128. ^{1}**H NMR of the 9e** (400 MHz, $CDCl_3$)

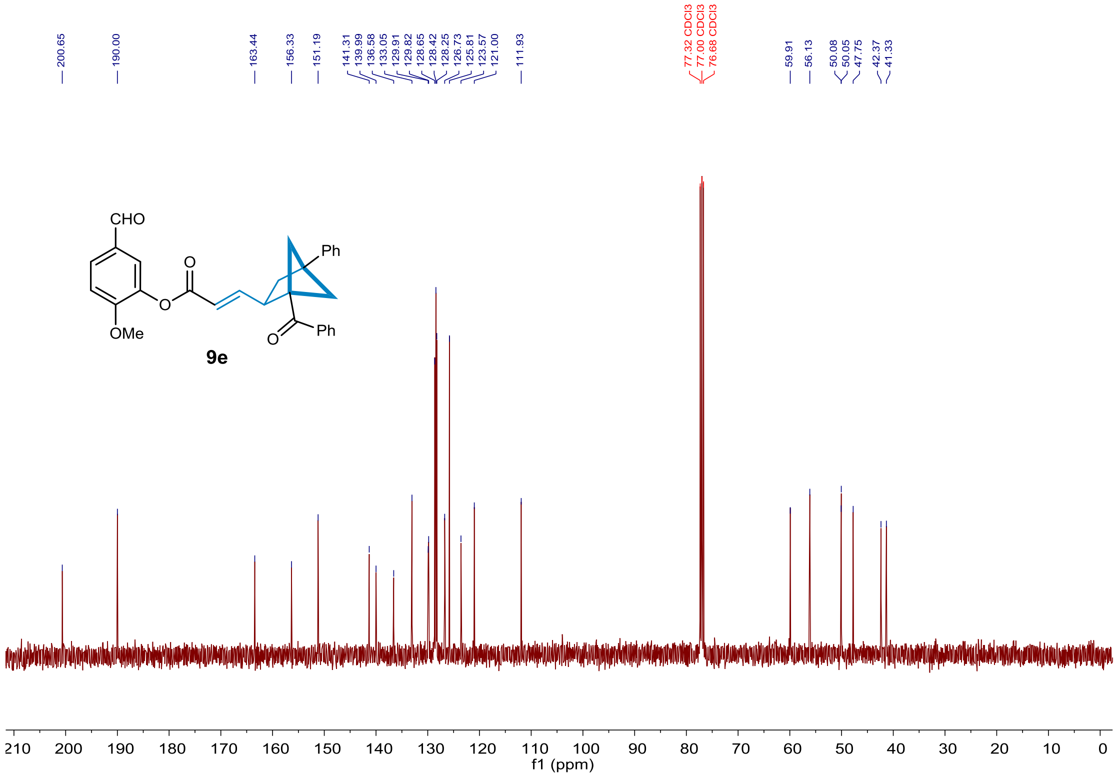

Supplementary Figure 129. ^{13}**C NMR of the 9e** (101 MHz, $CDCl_3$)

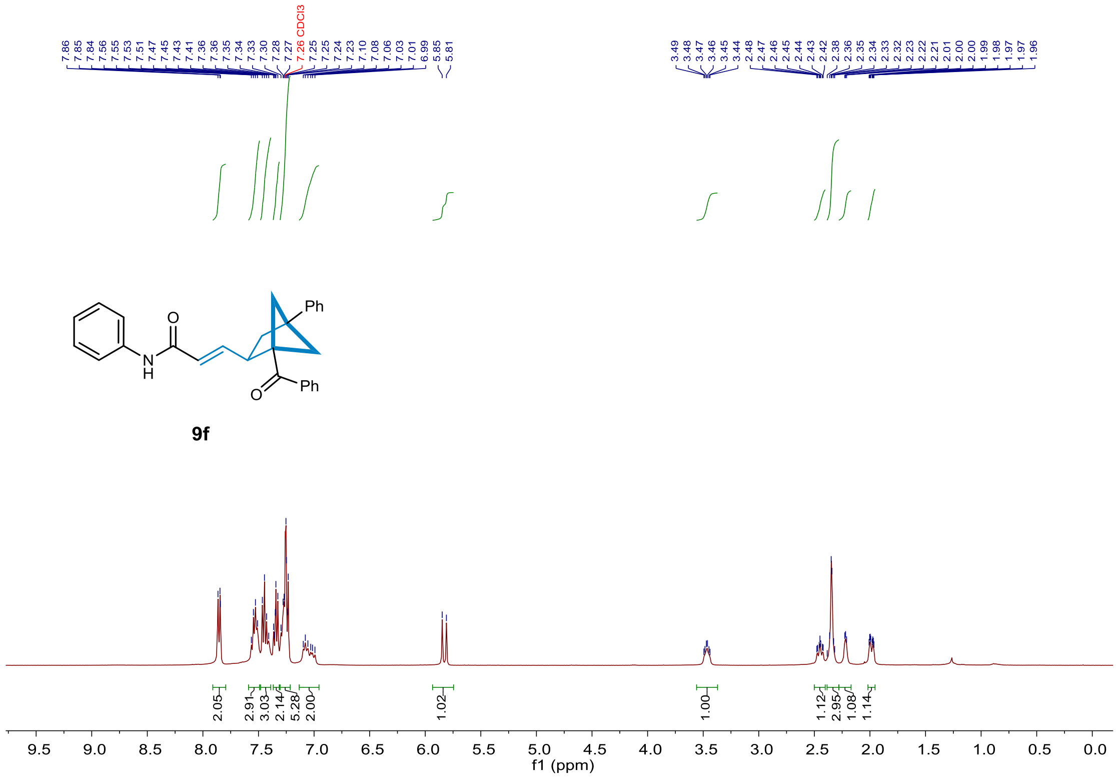

Supplementary Figure 130. **^{1}H NMR of the 9f** (400 MHz, $CDCl_3$)

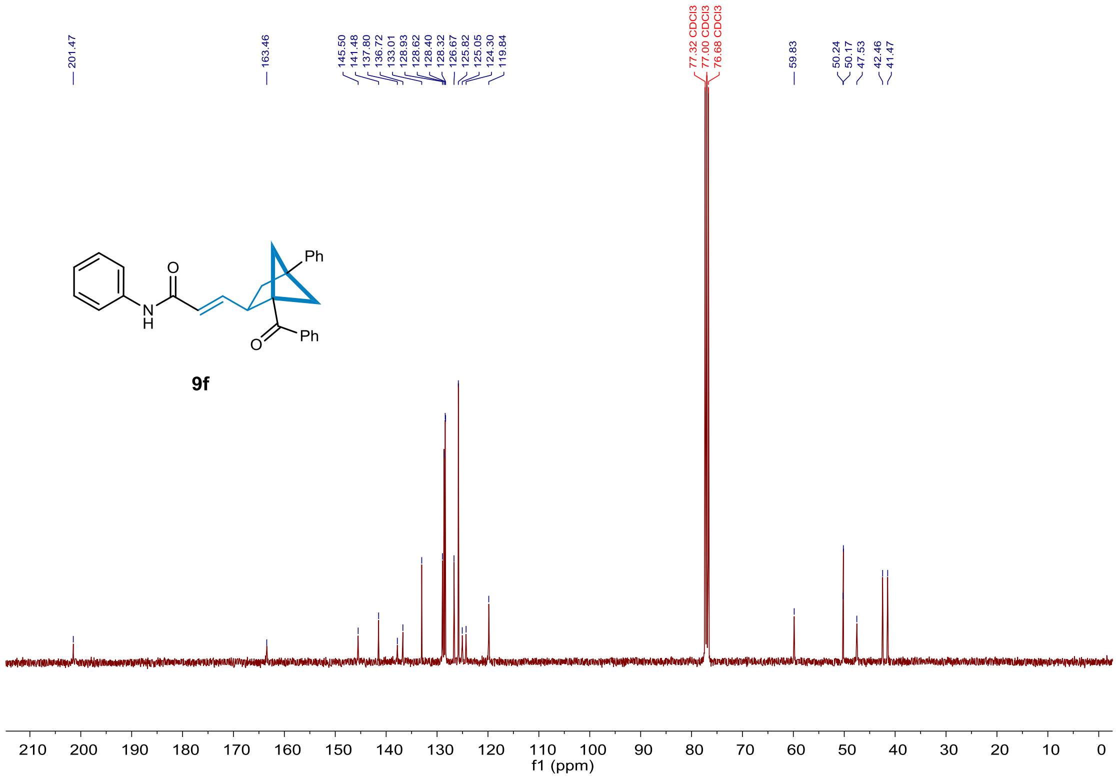

Supplementary Figure 131. **^{13}C NMR of the 9f** (101 MHz, $CDCl_3$)

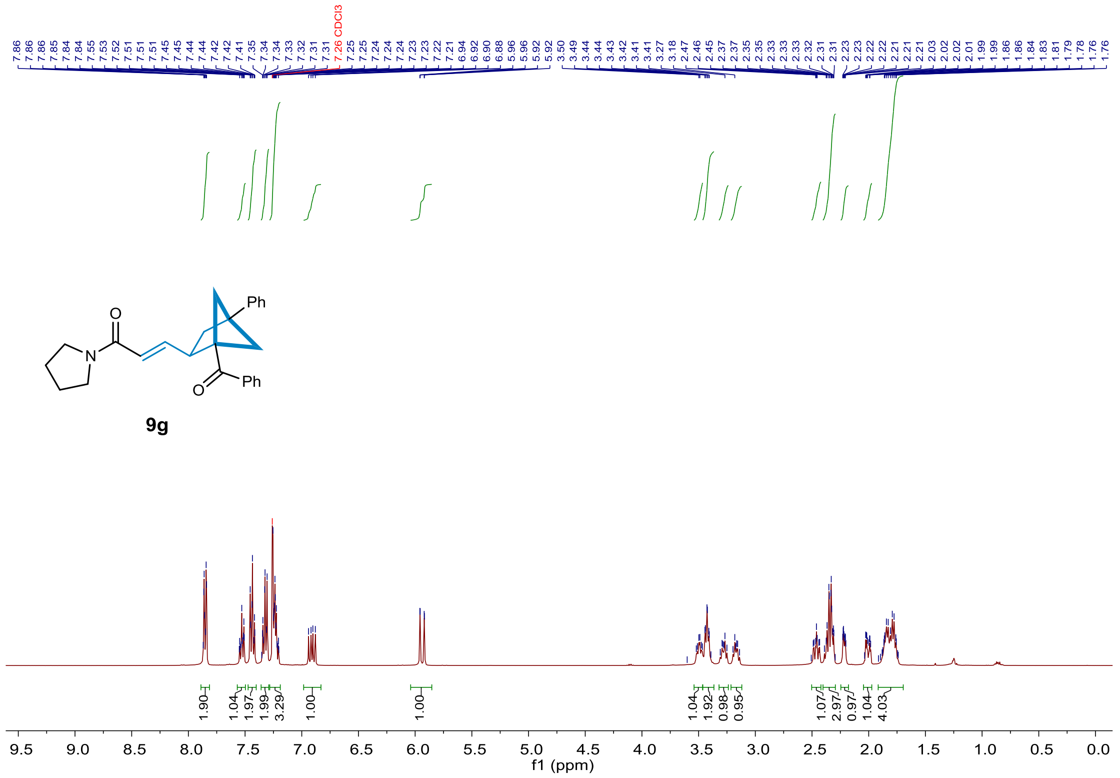

Supplementary Figure 132. ^{1}H NMR of the 9g (400 MHz, $CDCl_3$)

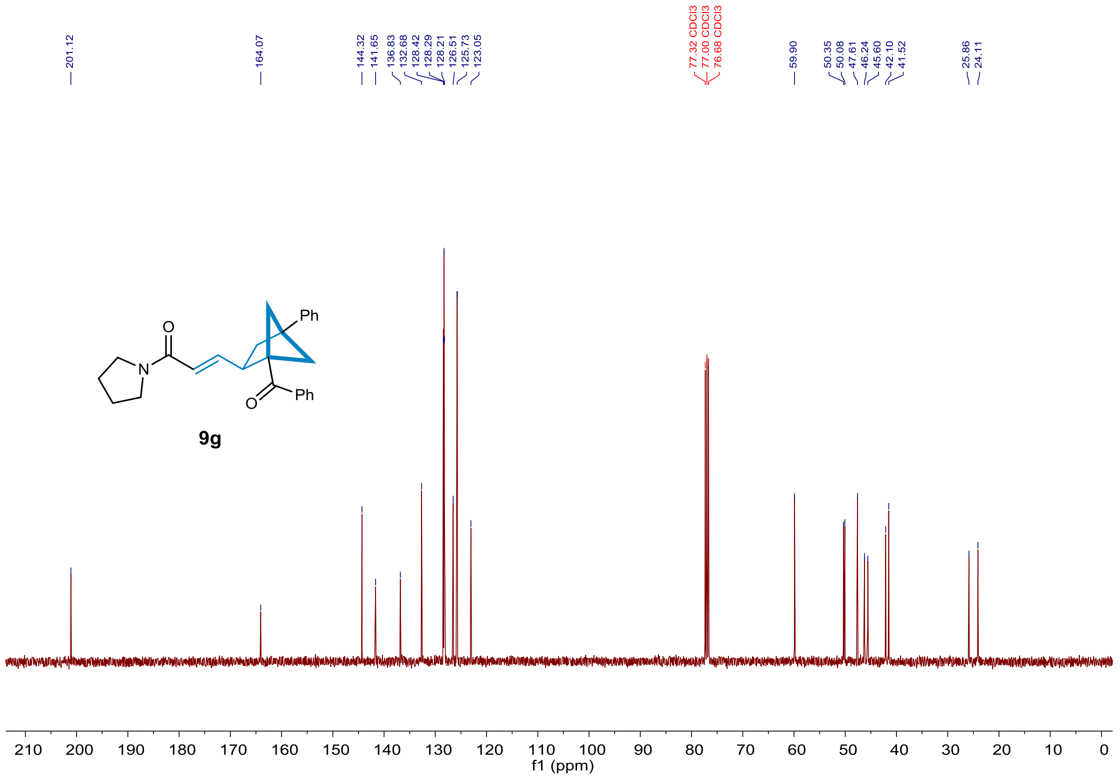

Supplementary Figure 133. ^{13}C NMR of the 9g (101 MHz, $CDCl_3$)

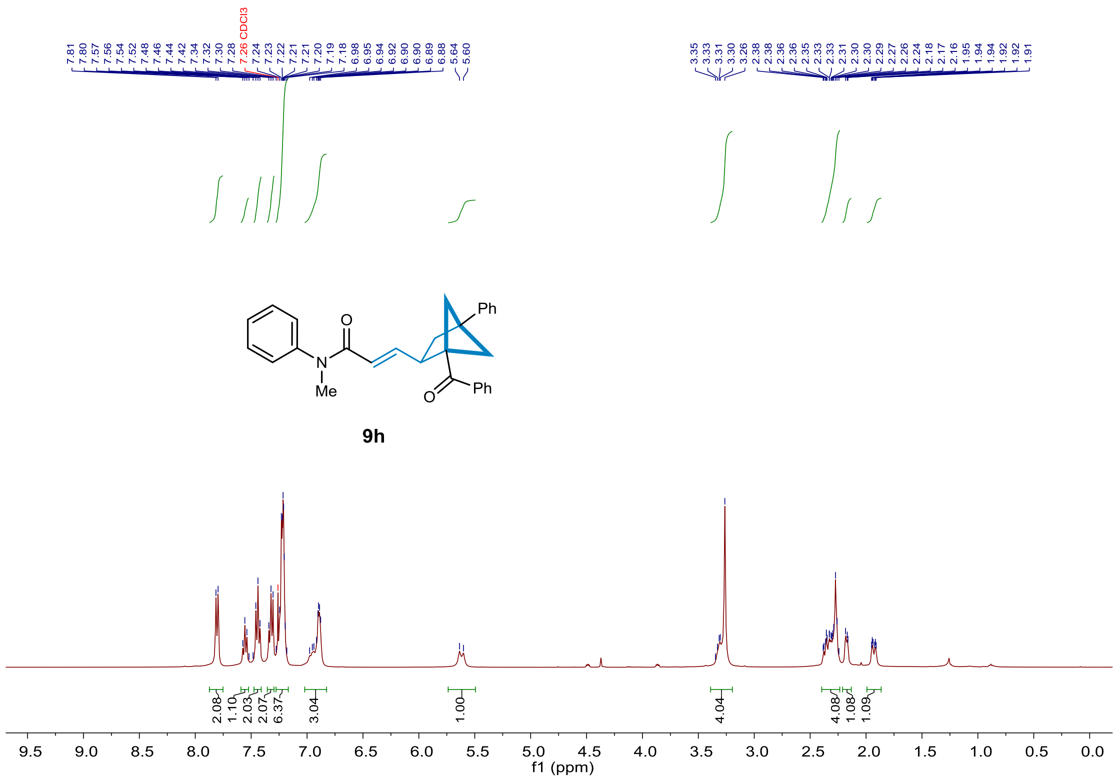

Supplementary Figure 134. ^{1}H NMR of the 9h (400 MHz, $CDCl_3$)

^{13}C NMR (101 MHz, $CDCl_3$)

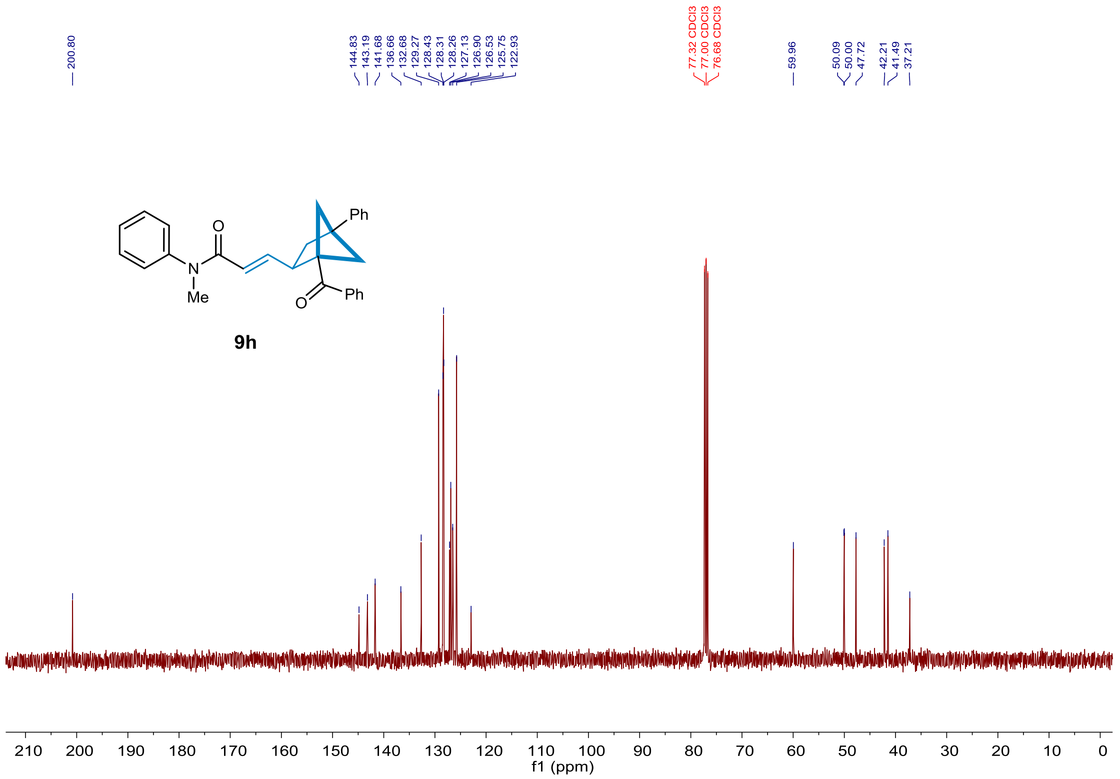

Supplementary Figure 135. ^{13}C NMR of the 9h (101 MHz, $CDCl_3$)

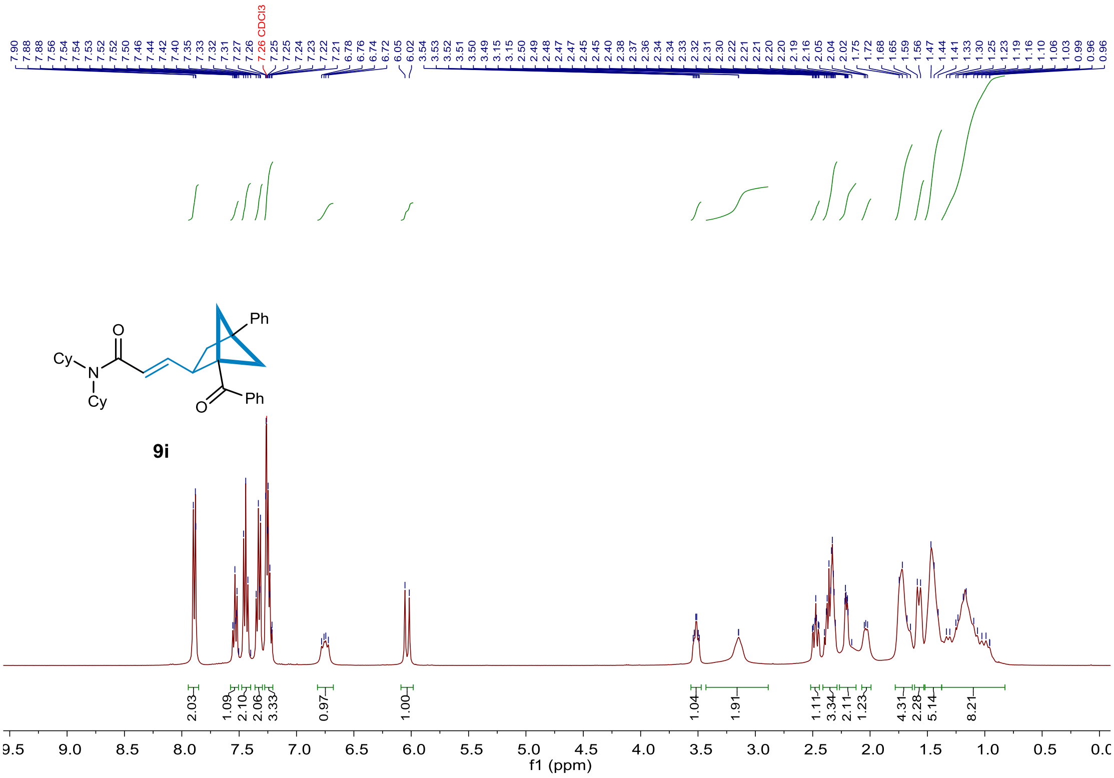

Supplementary Figure 136. ^{1}**H NMR of the 9i** (400 MHz, $CDCl_3$)

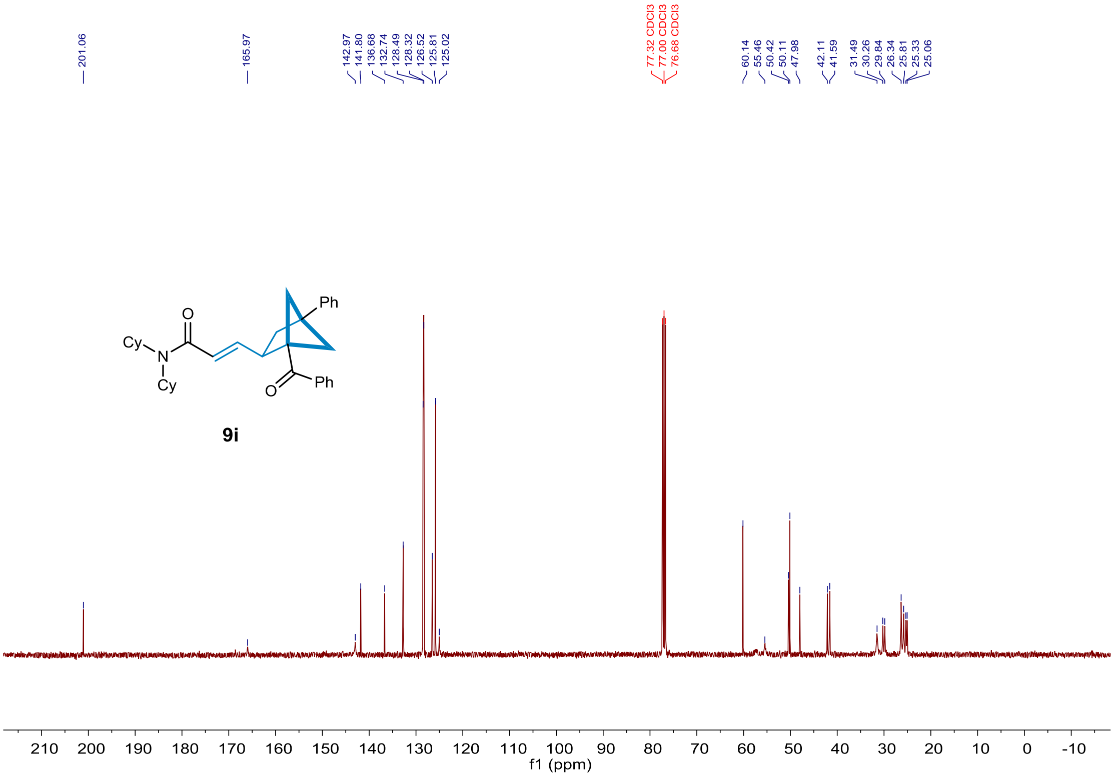

Supplementary Figure 137. ^{13}**C NMR of the 9i** (101 MHz, $CDCl_3$)

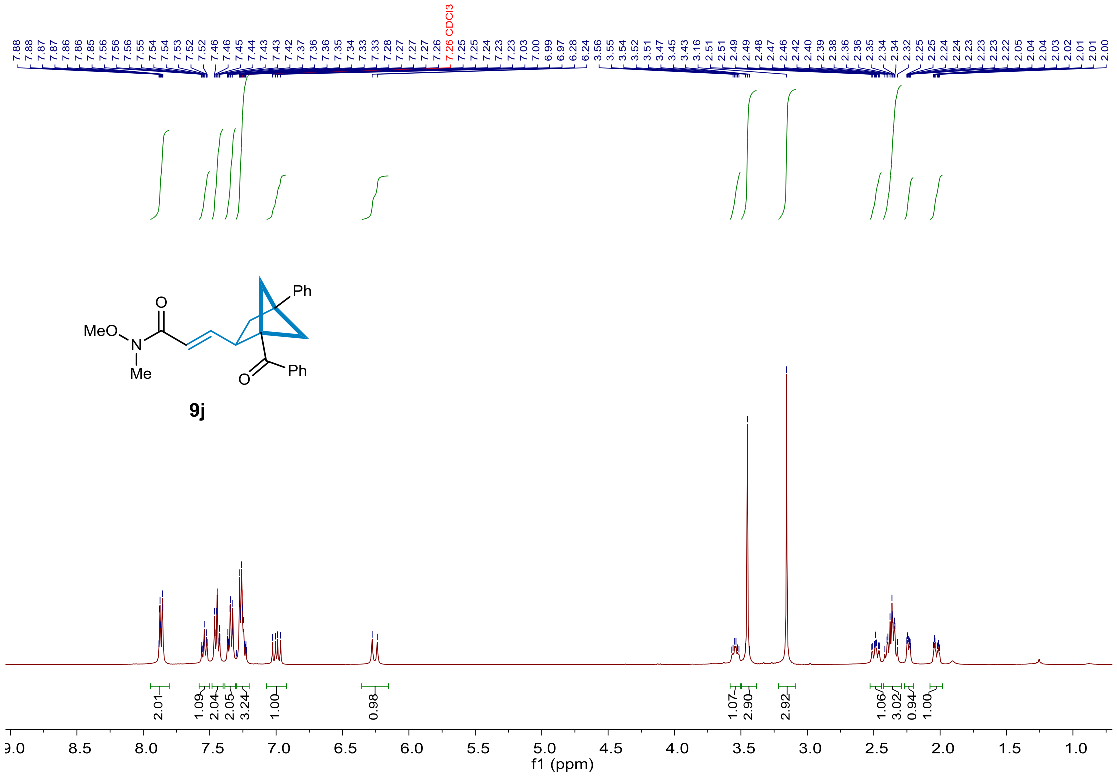

Supplementary Figure 138. **^{1}H NMR of the 9j** (400 MHz, $CDCl_3$)

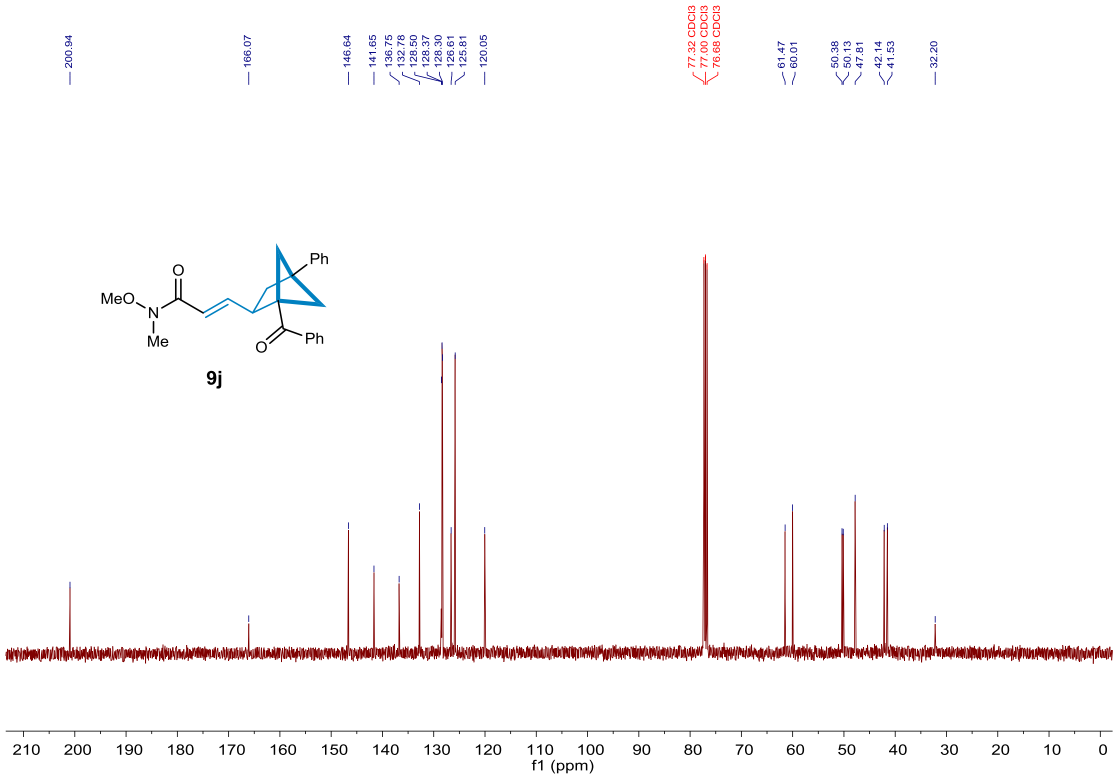

Supplementary Figure 139. **^{13}C NMR of the 9j** (101 MHz, $CDCl_3$)

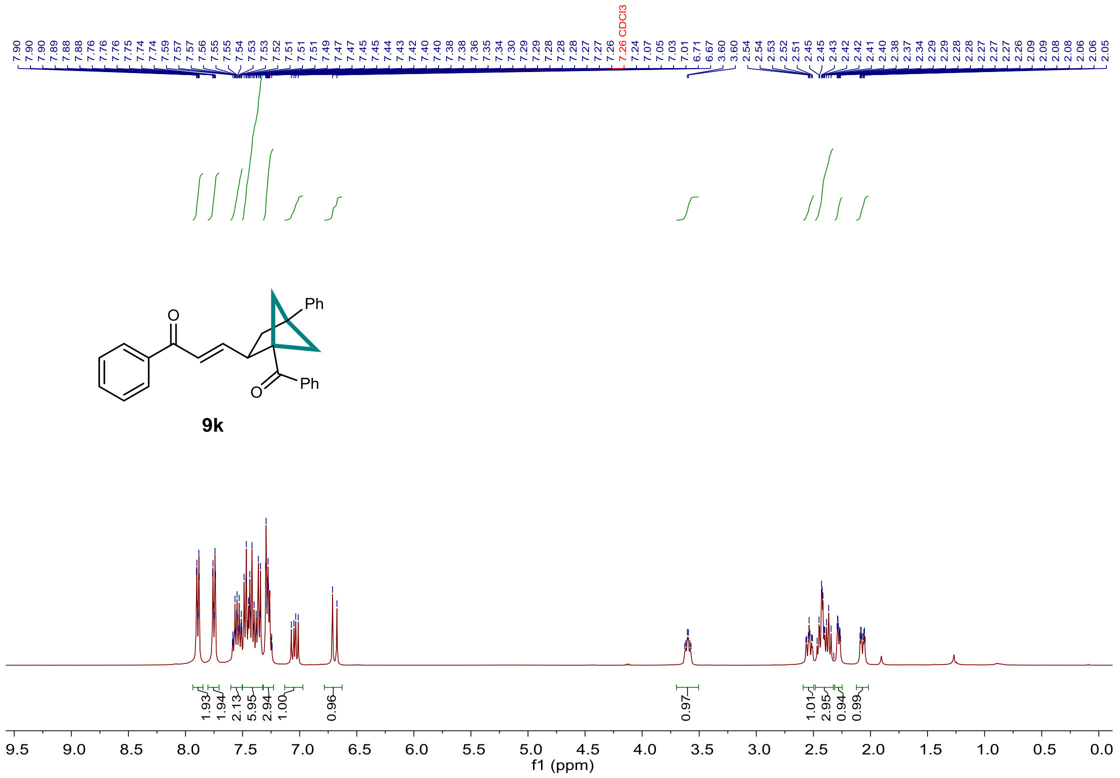

Supplementary Figure 140. **^{1}H NMR of the 9k** (400 MHz, $CDCl_3$)

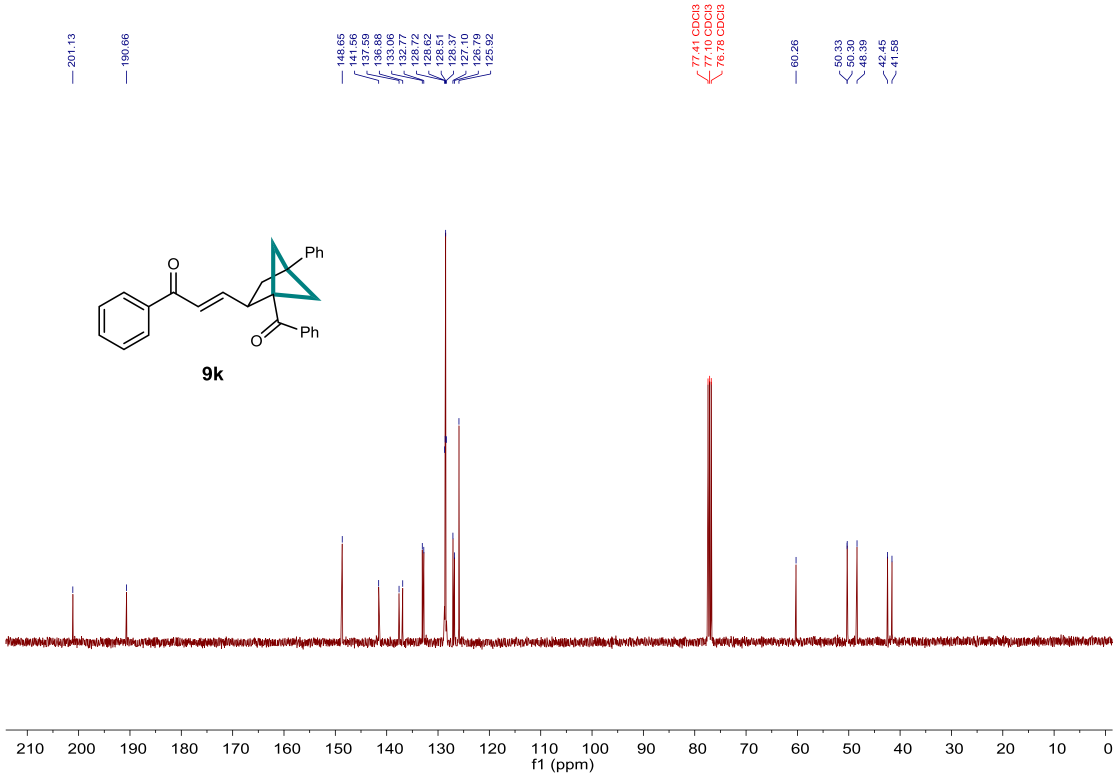

Supplementary Figure 141. **^{13}C NMR of the 9k** (101 MHz, $CDCl_3$)

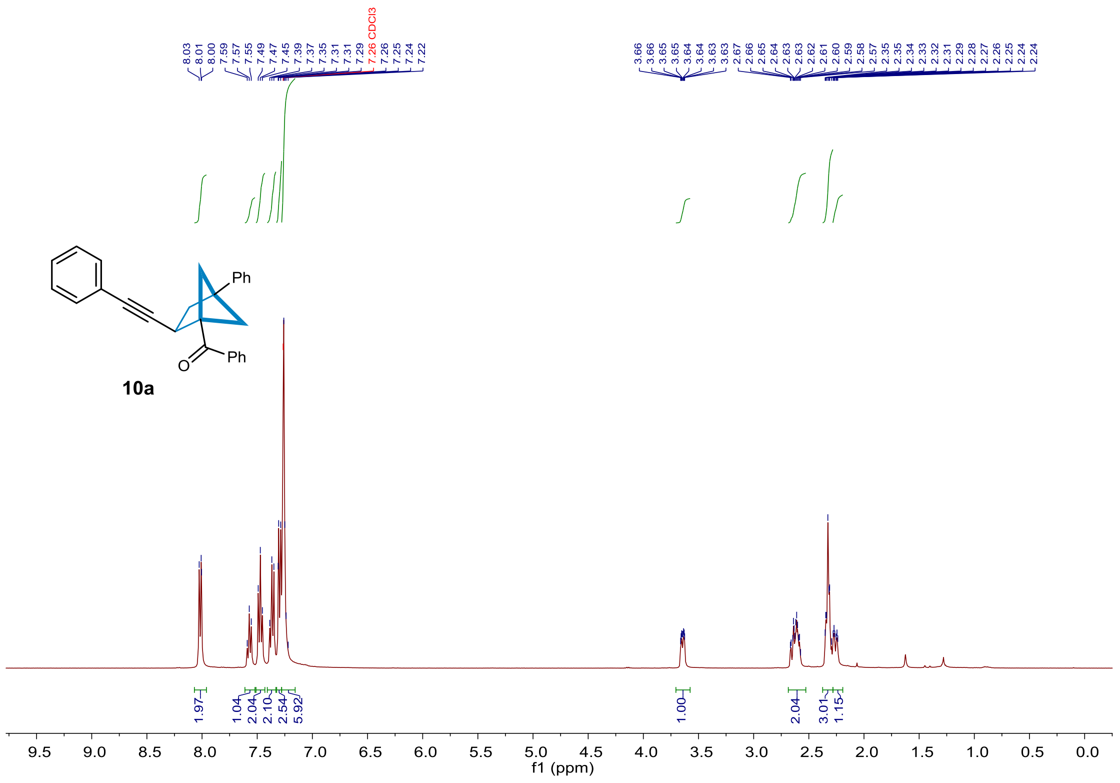

Supplementary Figure 142. **^{1}H NMR of the 10a** (400 MHz, $CDCl_3$)

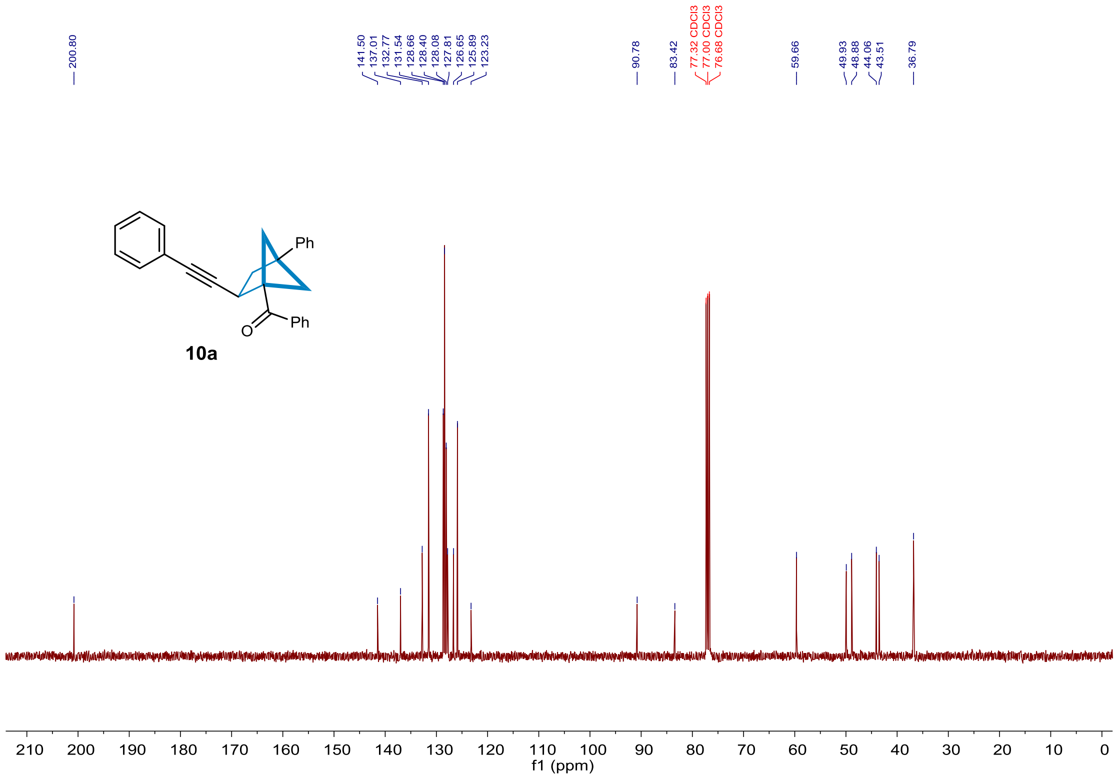

Supplementary Figure 143. **^{13}C NMR of the 10a** (101 MHz, $CDCl_3$)

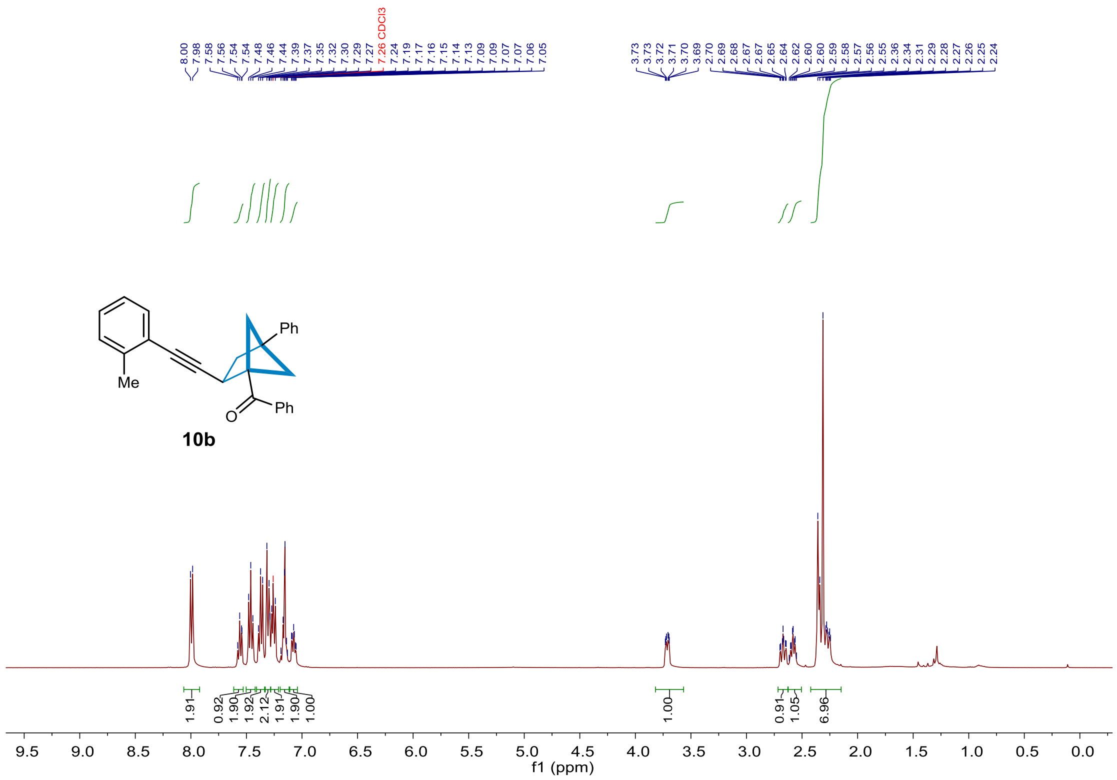

Supplementary Figure 144. ^{1}H NMR of the 10b (400 MHz, $CDCl_3$)

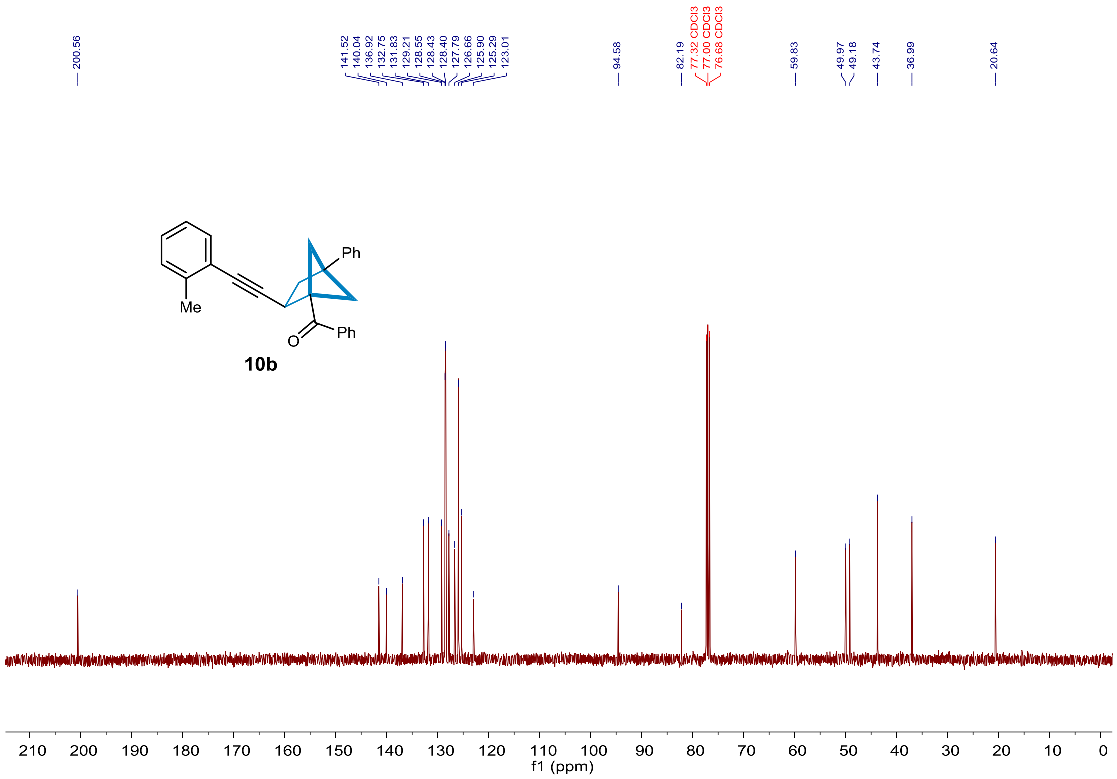

Supplementary Figure 145. ^{13}C NMR of the 10b (101 MHz, $CDCl_3$)

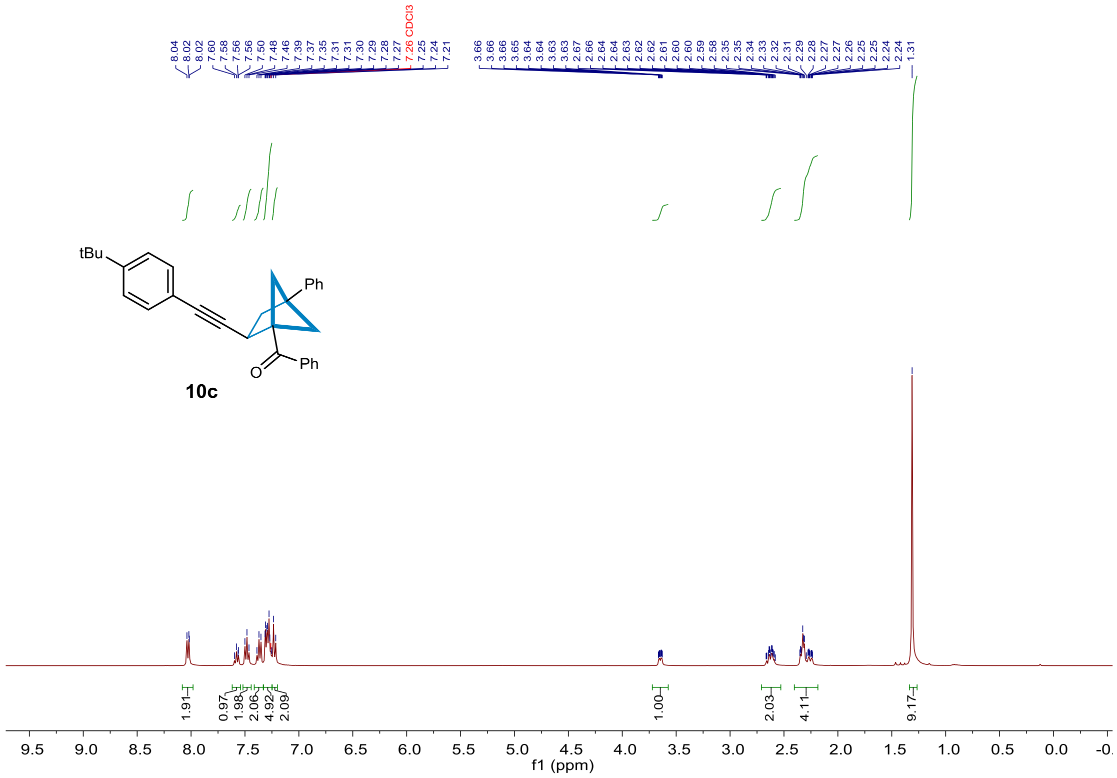

Supplementary Figure 146. ^{1}**H NMR of the 10c** (400 MHz, $CDCl_3$)

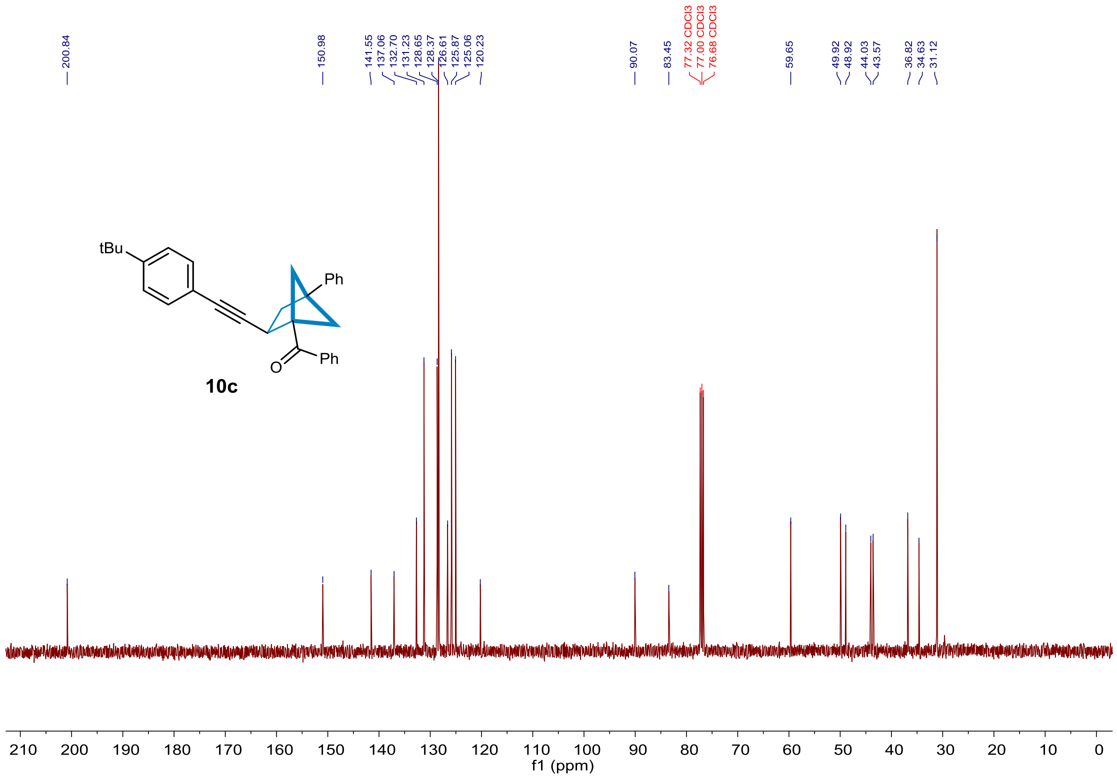

Supplementary Figure 147. ^{13}**C NMR of the 10c** (101 MHz, $CDCl_3$)

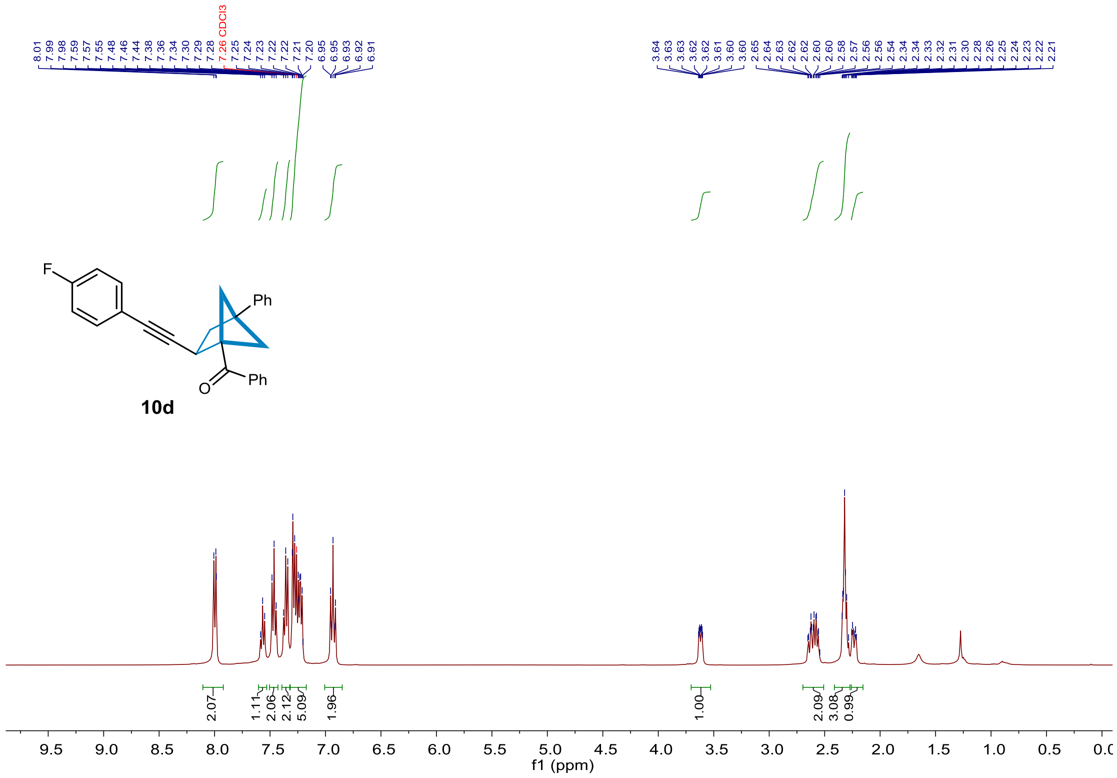

Supplementary Figure 148. ^{1}H NMR of the 10d (400 MHz, $CDCl_3$)

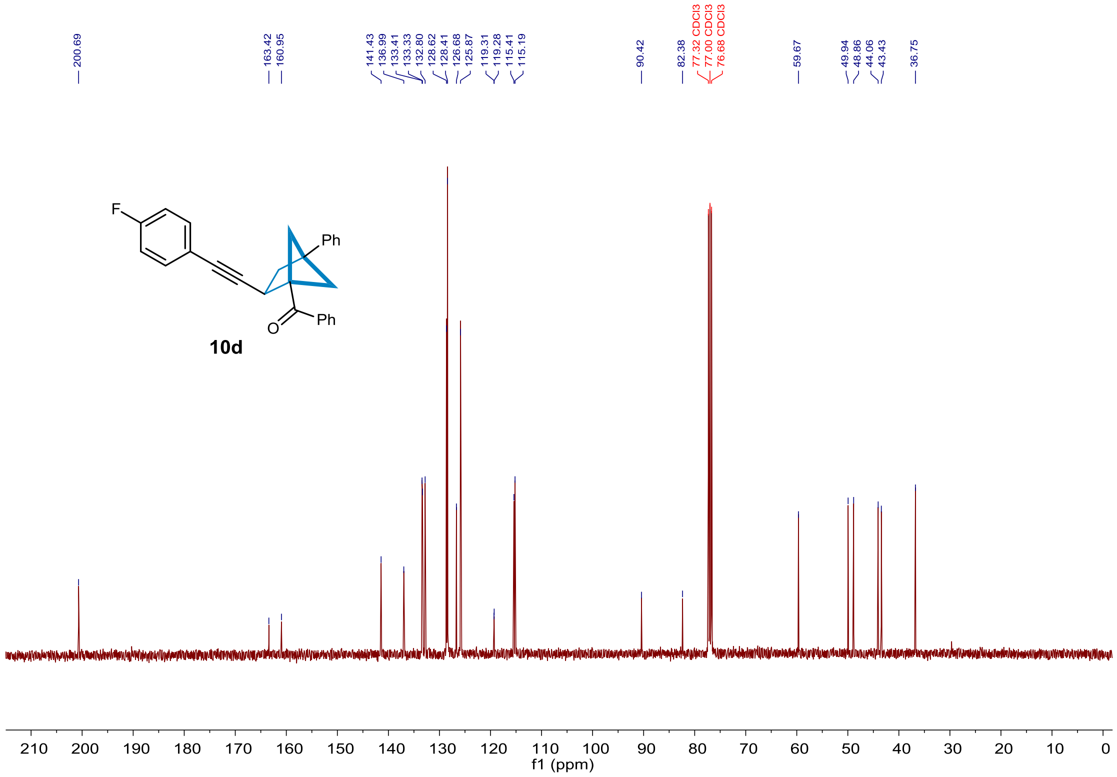

Supplementary Figure 149. ^{13}C NMR of the 10d (101 MHz, $CDCl_3$)

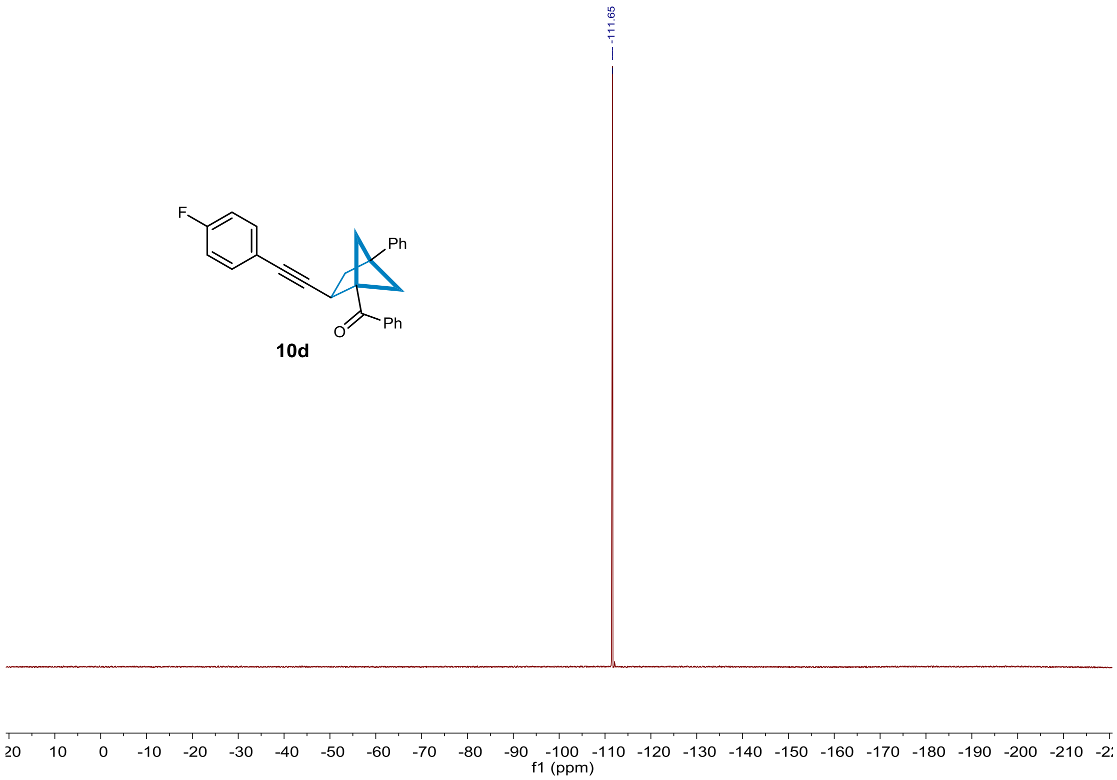

Supplementary Figure 150. **^{19}F NMR of the 10d** (377 MHz, $CDCl_3$)

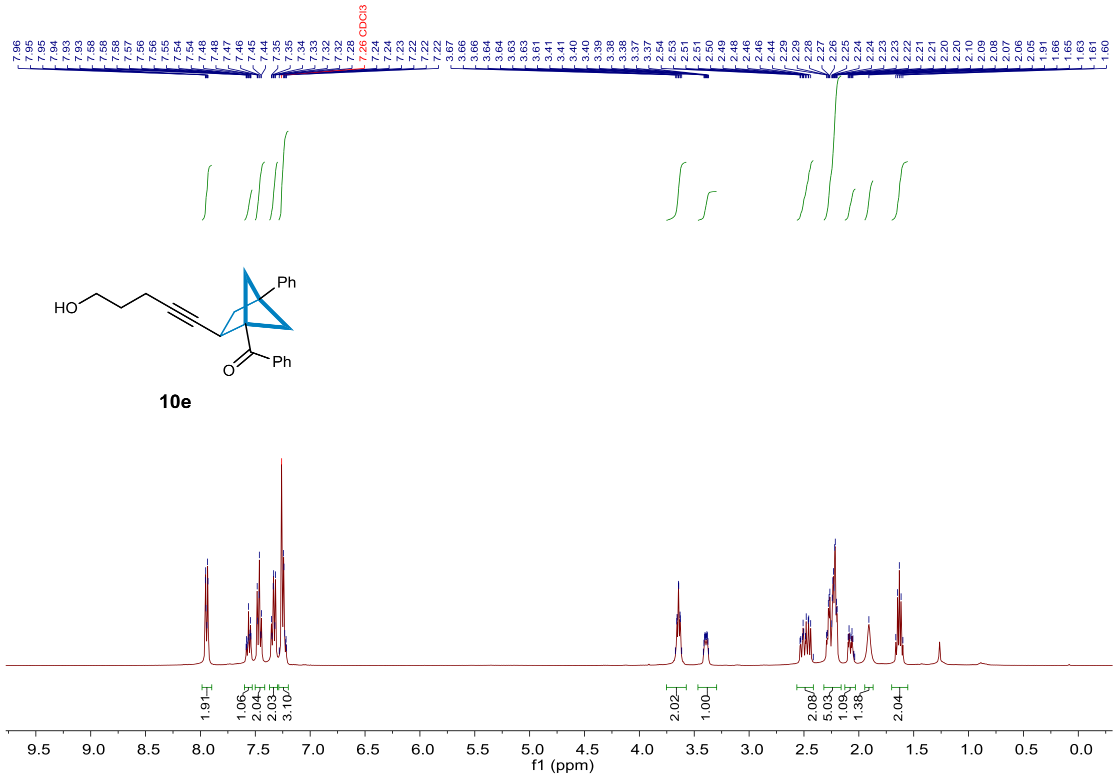

Supplementary Figure 151. **^{1}H NMR of the 10e** (400 MHz, $CDCl_3$)

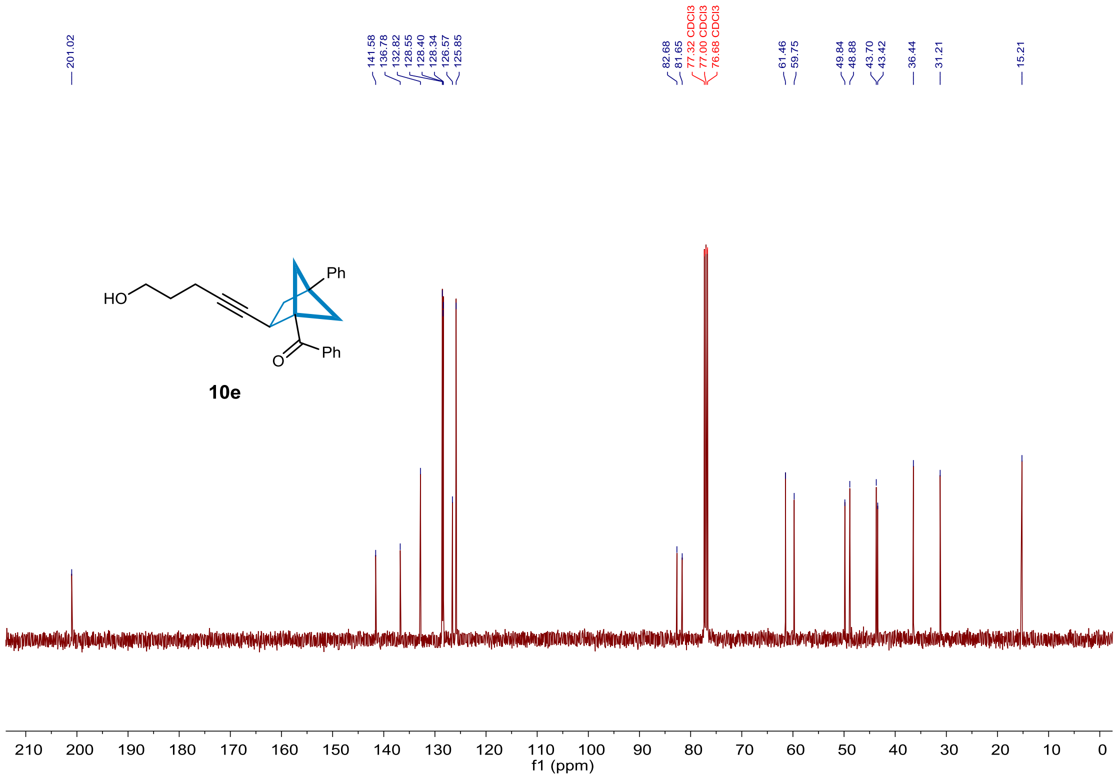

Supplementary Figure 152. **^{13}C NMR of the 10e** (101 MHz, $CDCl_3$)

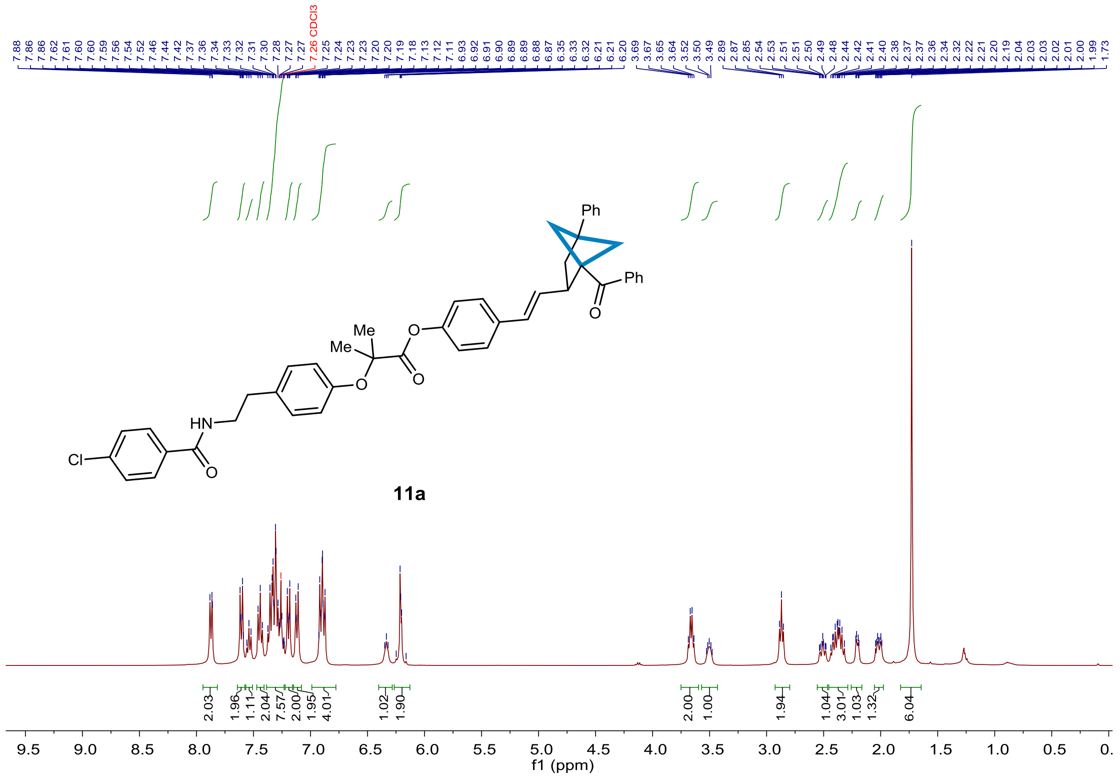

Supplementary Figure 153. **^{1}H NMR of the 11a** (400 MHz, $CDCl_3$)

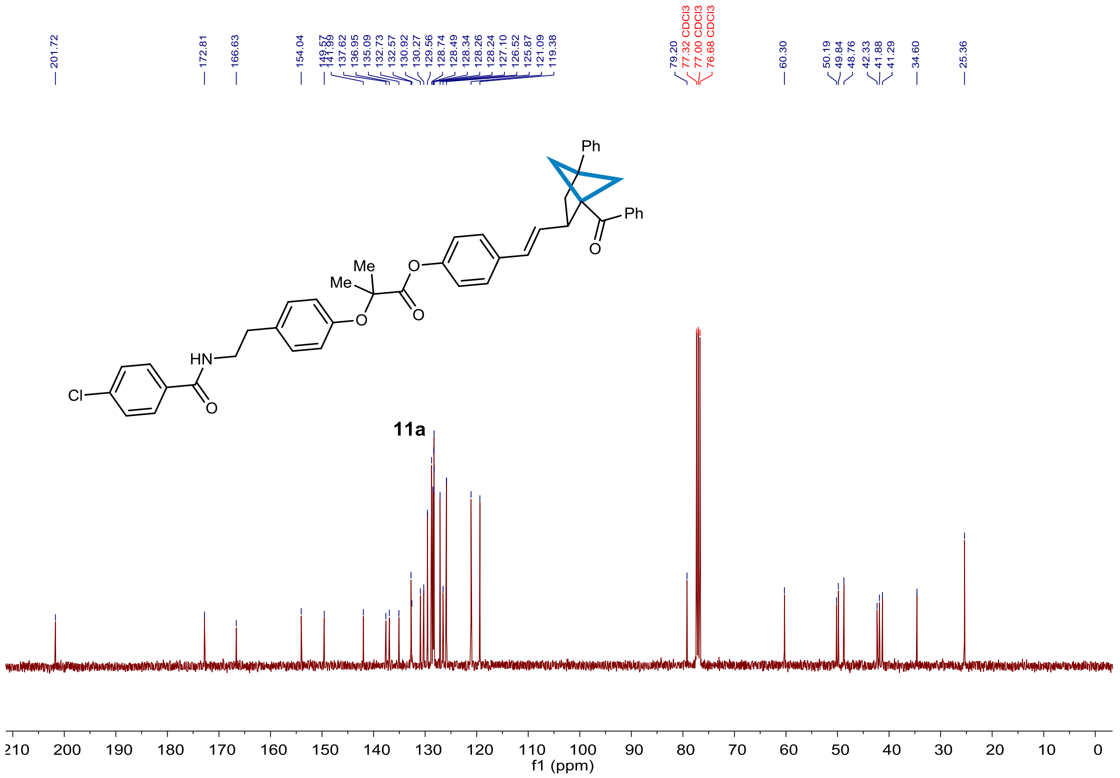

Supplementary Figure 154. **^{13}C NMR of the 11a** (101 MHz, $CDCl_3$)

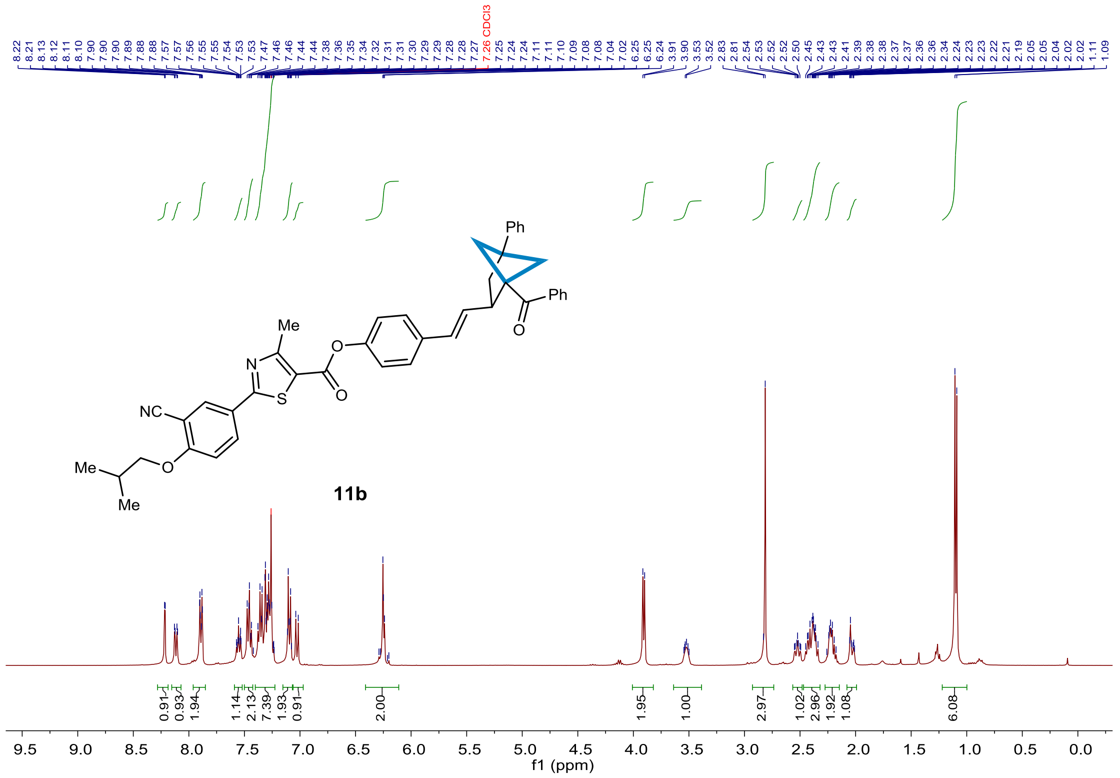

Supplementary Figure 155. **^{1}H NMR of the 11b** (400 MHz, $CDCl_3$)

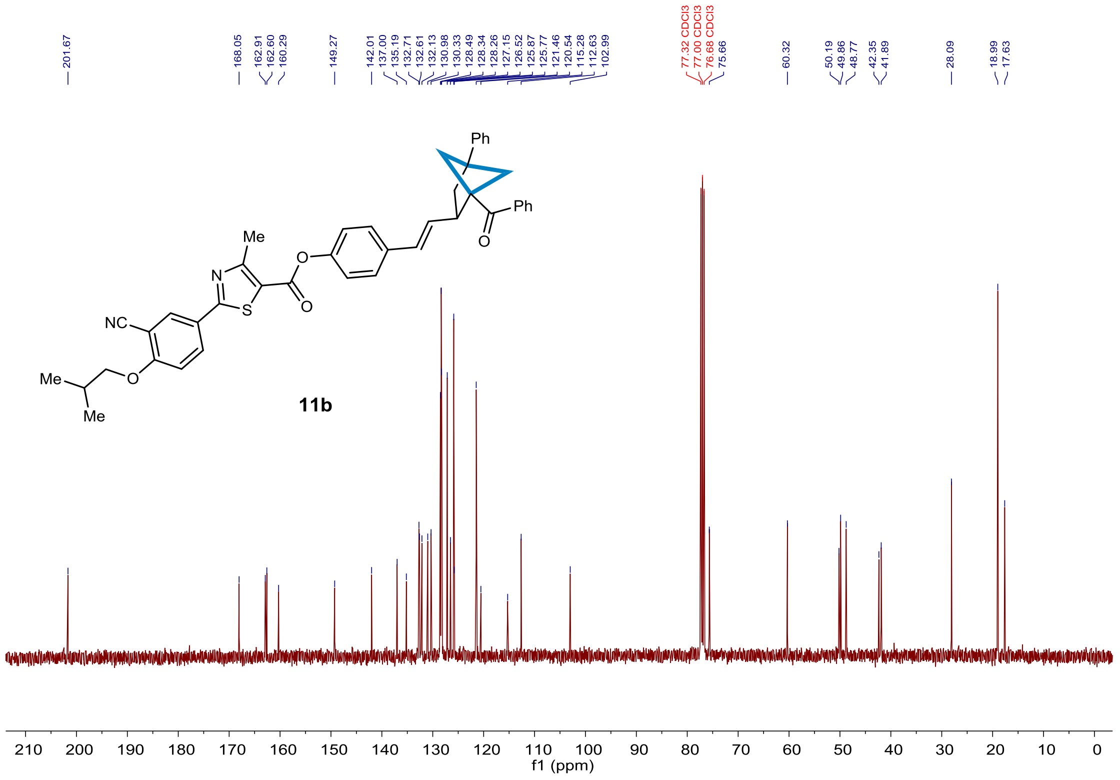

Supplementary Figure 156. **^{13}C NMR of the 11b** (101 MHz, $CDCl_3$)

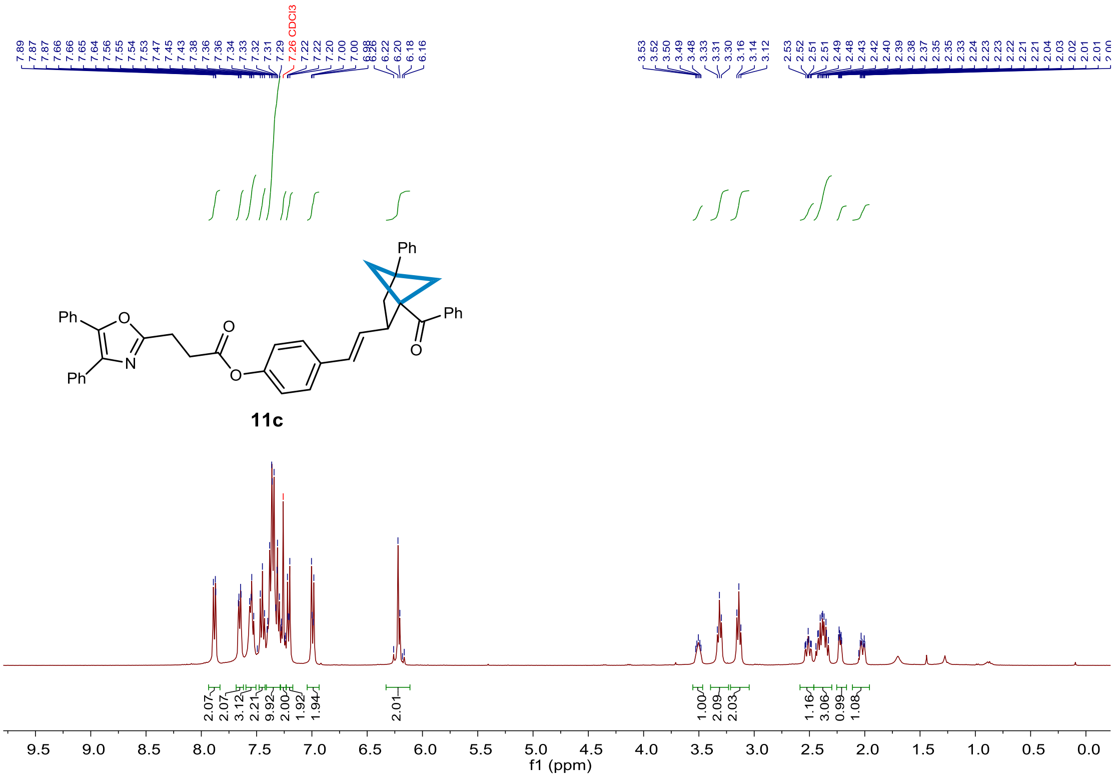

Supplementary Figure 157. **^{1}H NMR of the 11c** (400 MHz, $CDCl_3$)

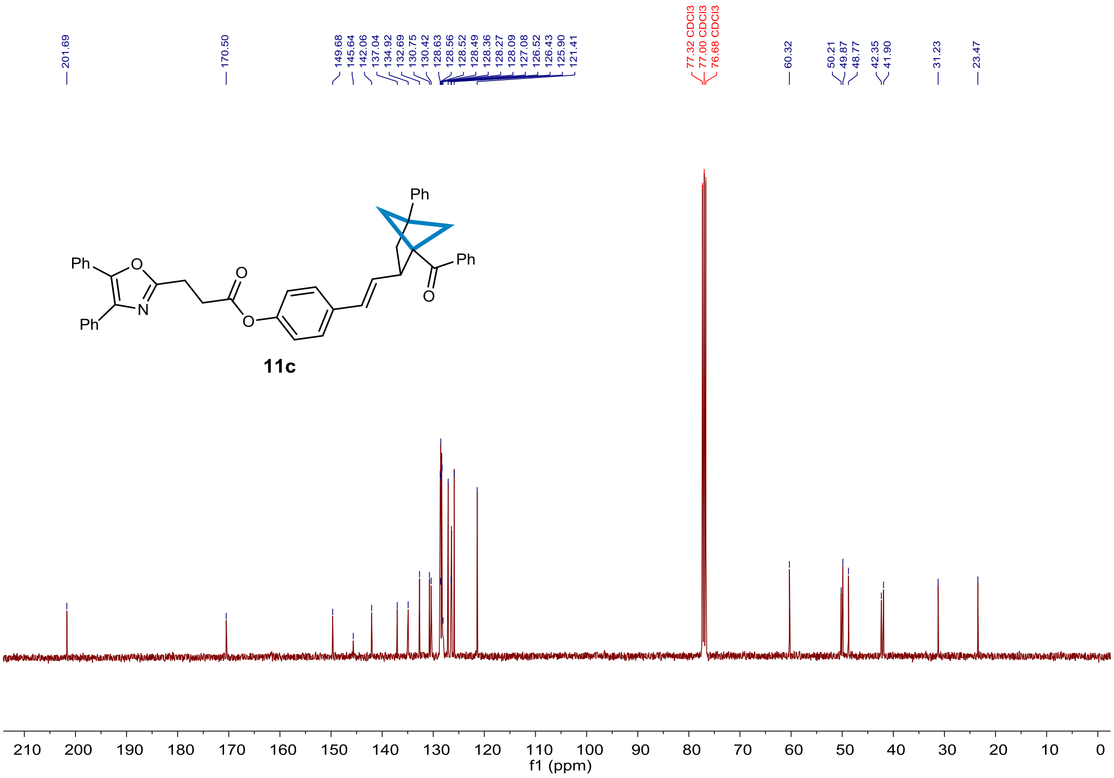

Supplementary Figure 158. **^{13}C NMR of the 11c** (101 MHz, $CDCl_3$)

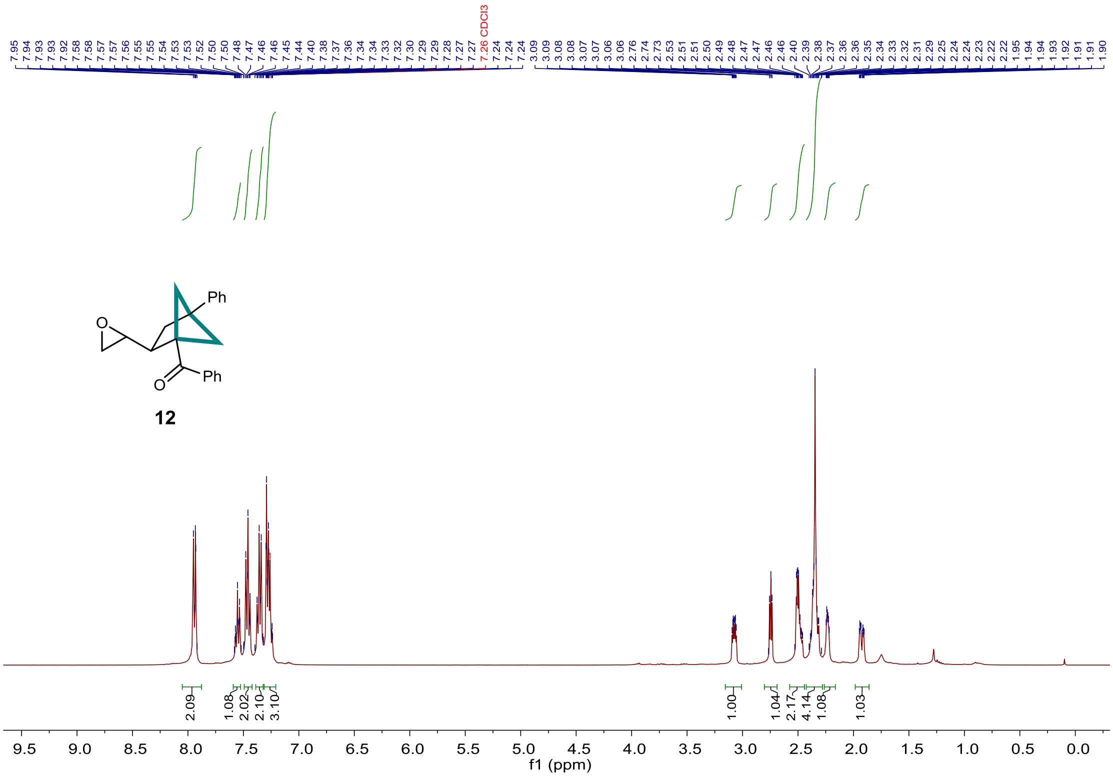

Supplementary Figure 159. **^{1}H NMR of the 12** (400 MHz, $CDCl_3$)

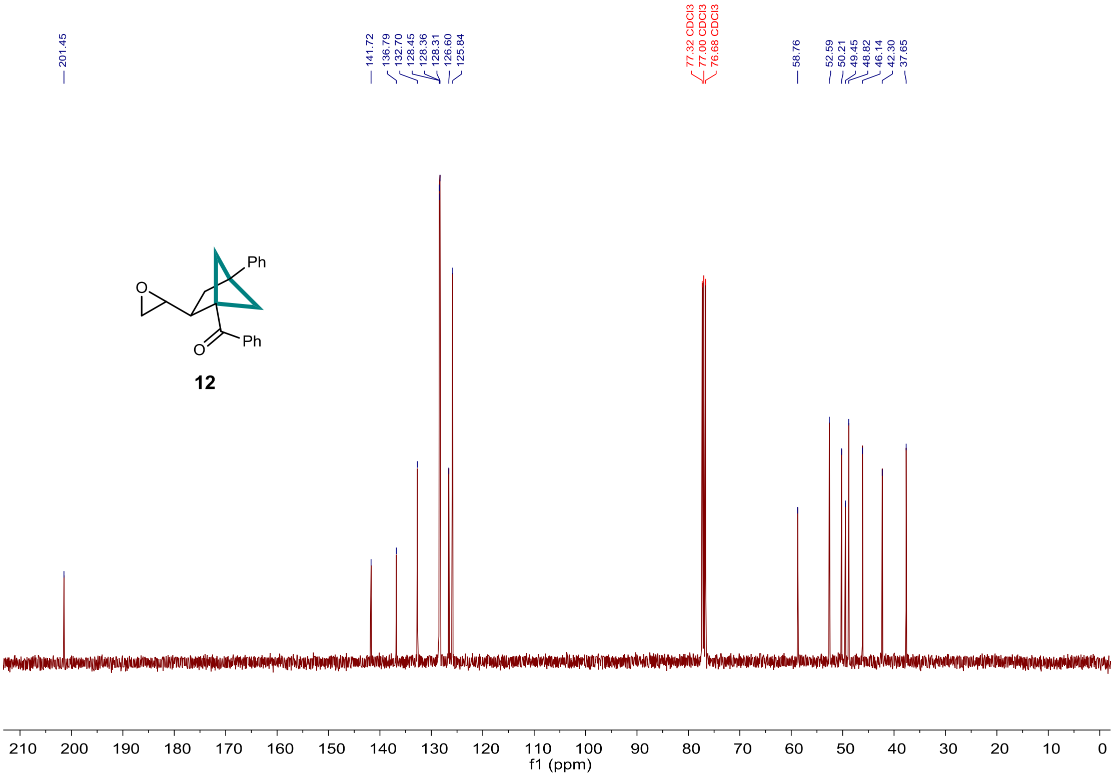

Supplementary Figure 160. **^{13}C NMR of the 12** (101 MHz, $CDCl_3$)

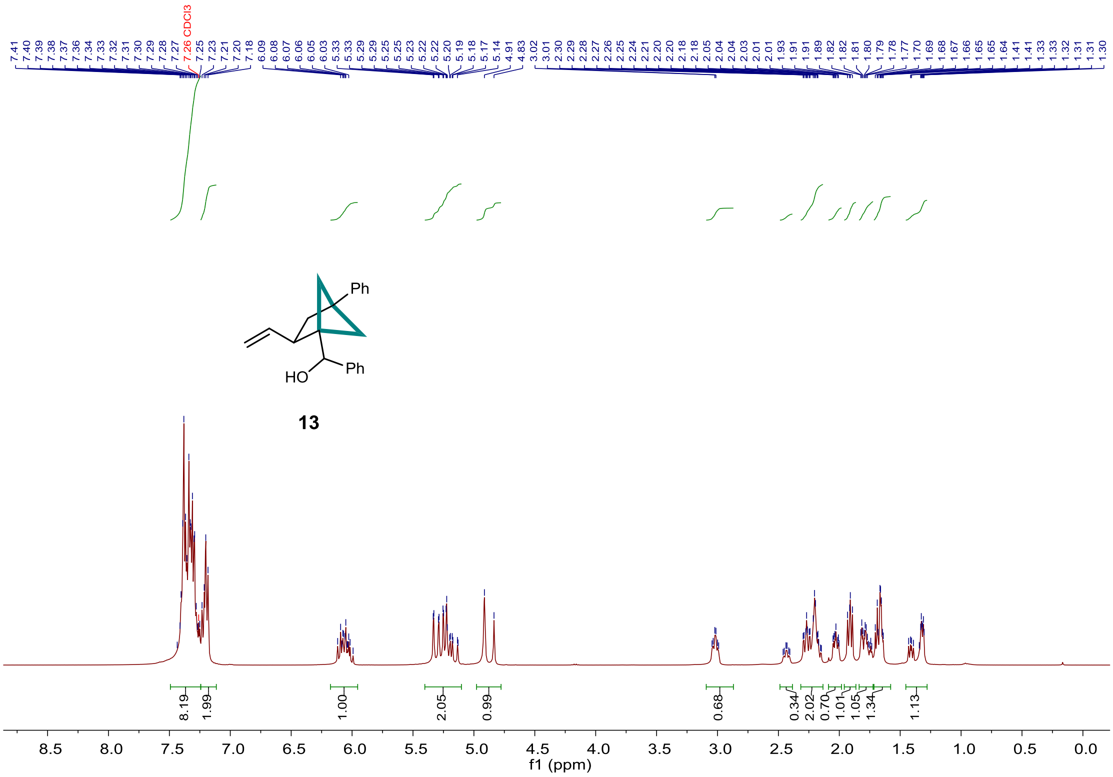

Supplementary Figure 161. **^{1}H NMR of the 13** (400 MHz, $CDCl_3$)

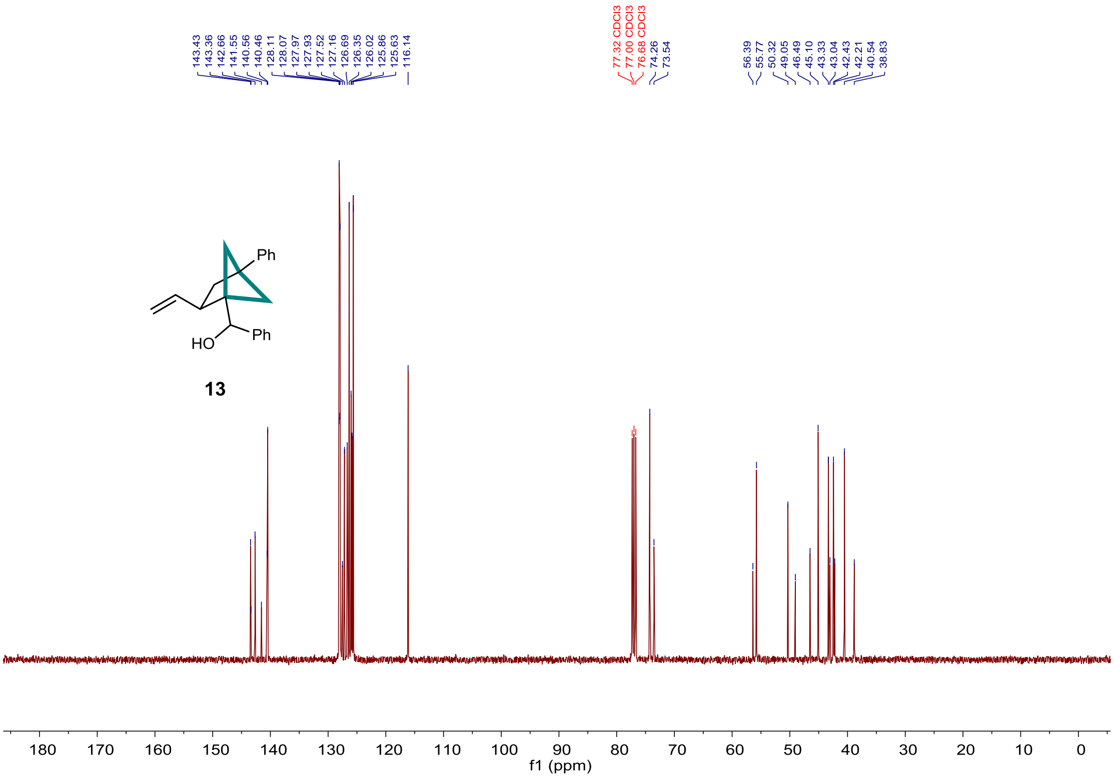

Supplementary Figure 162. **^{13}C NMR of the 13** (101 MHz, $CDCl_3$)

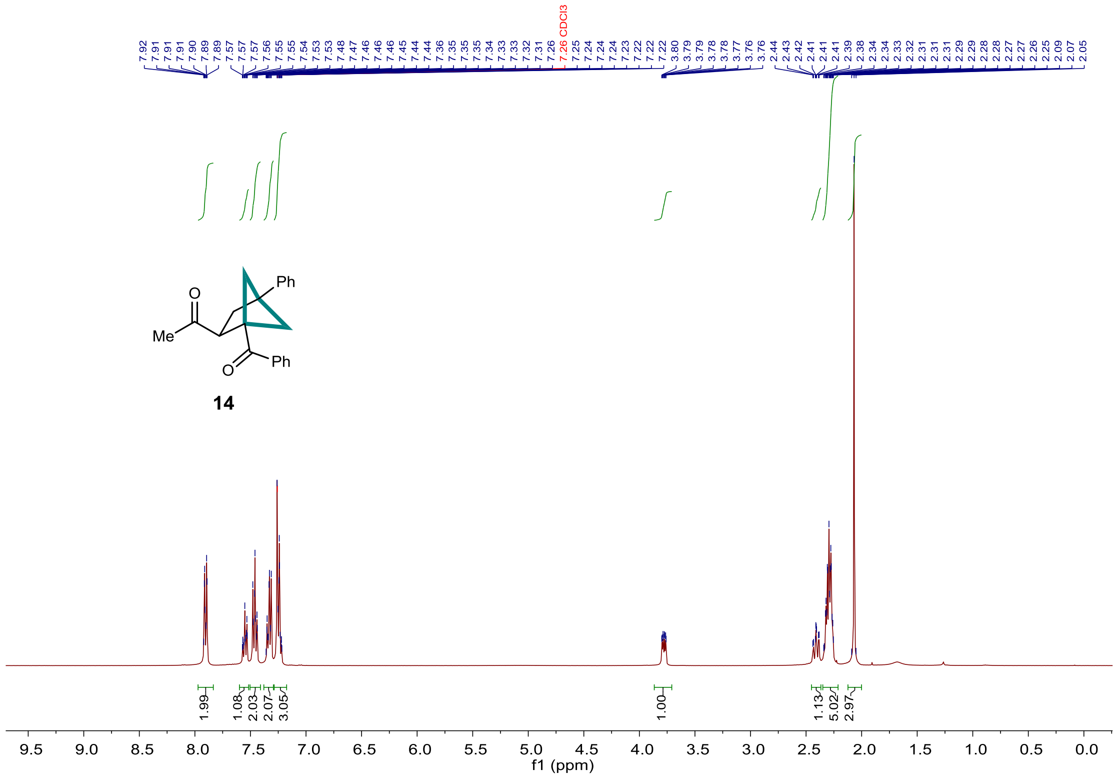

Supplementary Figure 163. **^{1}H NMR of the 14** (400 MHz, $CDCl_3$)

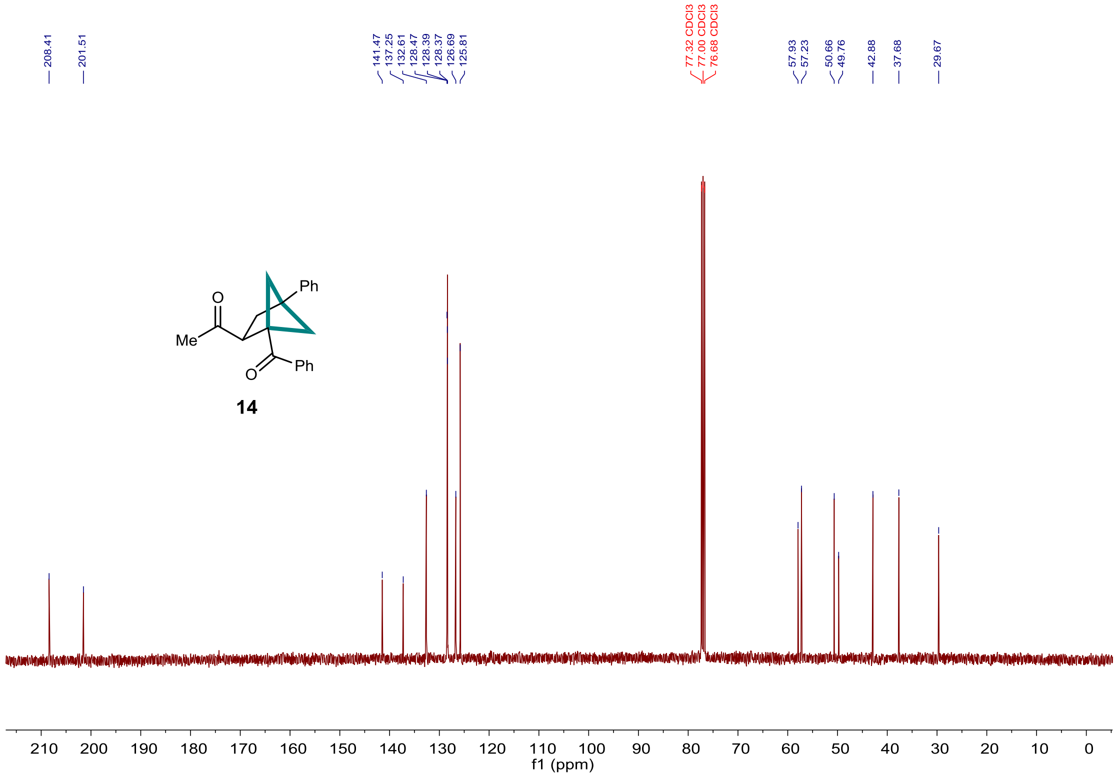

Supplementary Figure 164. **^{13}C NMR of the 14** (101 MHz, $CDCl_3$)

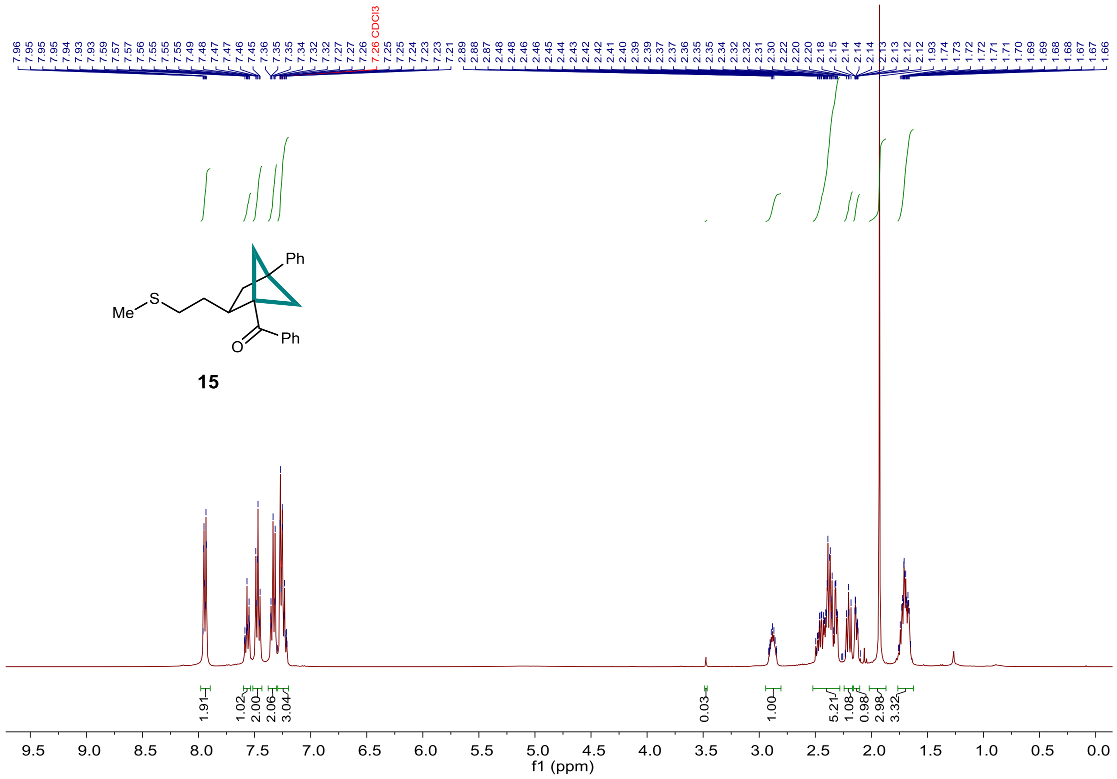

Supplementary Figure 165. **^{1}H NMR of the 15** (400 MHz, $CDCl_3$)

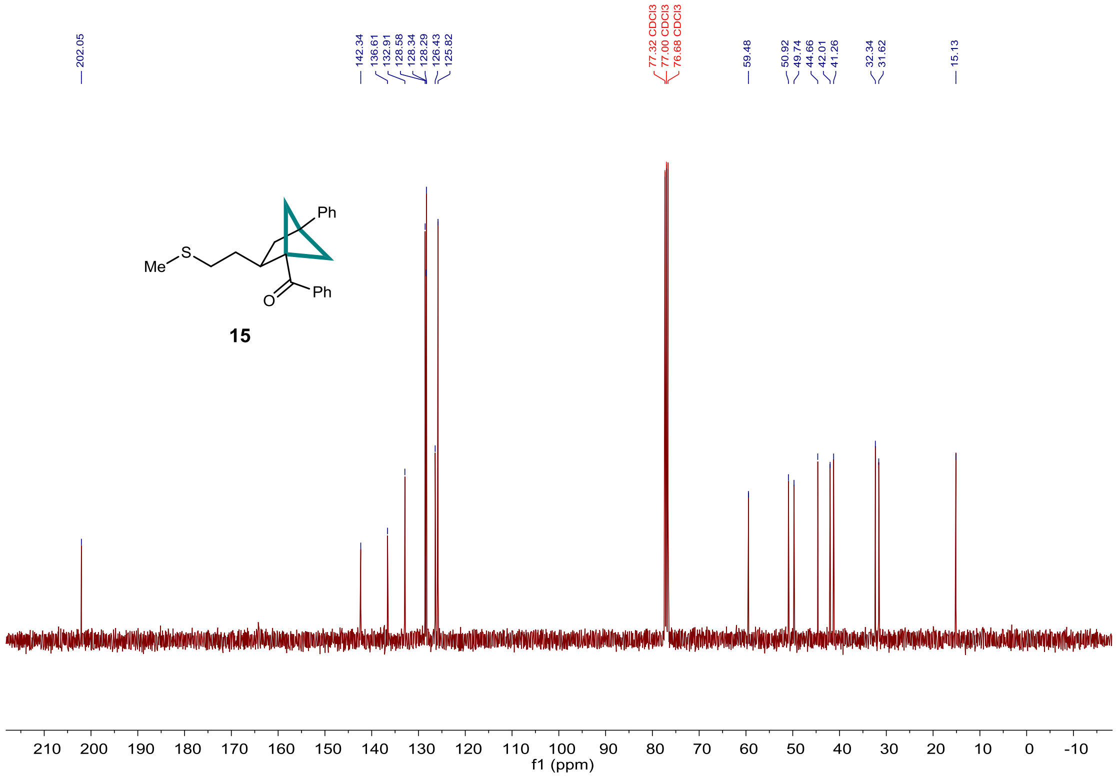

Supplementary Figure 166. **^{13}C NMR of the 14** (101 MHz, $CDCl_3$)

4 Supplementary References

1. Shen, J.; Xu, Z.; Yang, S.; Li, S.; Jiang, J.; Zhang, Y-Q. Quaternary Stereocenters via Catalytic Enantioconvergent Allylation of Epoxides. J. Am. Chem. Soc. 145, 21122-21131 (2023).
2. Guo, R.; Chang, Y-C.; Herter, L.; Salome, C.; Braley, S. E.; Fessard, T. C.; Brown, M. K. Strain-release [2π + 2σ] cycloadditions for the synthesis of bicyclo[2.1.1]hexanes initiated by energy transfer. *J. Am. Chem. Soc.* **144**, 7988-7994 (2022).
3. Fernandes, R. A.; Gangani, A. J.; Panja, A. Synthesis of 5-Vinyl-2-isoxazolines by Palladium-Catalyzed Intramolecular O-Allylation of Ketoximes. *Org. Lett.* **23**, 6227–6231 (2021).
4. Yang, Q-L.; Wang, X-Y.; Lu, J-Y.; Zhang, L-P.; Fang, P.; Mei, T-S.Copper-Catalyzed Electrochemical C–H Amination of Arenes with Secondary Amines. *J. Am. Chem. Soc.* **140**, 11487–11494 (2018).
5. Gaussian 16, Revision C.01, Frisch, M. J.; Trucks, G. W.; Schlegel, H. B.; Scuseria, G. E.; Robb, M. A.; Cheeseman, J. R.; Scalmani, G.; Barone, V.; Petersson, G. A.; Nakatsuji, H.; Li, X.; Caricato, M.; Marenich, A. V.; Bloino, J.; Janesko, B. G.; Gomperts, R.; Mennucci, B.; Hratchian, H. P.; Ortiz, J. V.; Izmaylov, A. F.; Sonnenberg, J. L.; Williams-Young, D.; Ding, F.; Lipparini, F.; Egidi, F.; Goings, J.; Peng, B.; Petrone, A.; Henderson, T.; Ranasinghe, D.; Zakrzewski, V. G.; Gao, J.; Rega, N.; Zheng, G.; Liang, W.; Hada, M.; Ehara, M.; Toyota, K.; Fukuda, R.; Hasegawa, J.; Ishida, M.; Nakajima, T.; Honda, Y.; Kitao, O.; Nakai, H.; Vreven, T.; Throssell, K.; Montgomery, J. A., Jr.; Peralta, J. E.; Ogliaro, F.; Bearpark, M. J.; Heyd, J. J.; Brothers, E. N.; Kudin, K. N.; Staroverov, V. N.; Keith, T. A.; Kobayashi, R.; Normand, J.; Raghavachari, K.; Rendell, A. P.; Burant, J. C.; Iyengar, S. S.; Tomasi, J.; Cossi, M.; Millam, J. M.; Klene, M.; Adamo, C.; Cammi, R.; Ochterski, J. W.; Martin, R. L.; Morokuma, K.; Farkas, O.; Foresman, J. B.; Fox, D. J. Gaussian, Inc., Wallingford CT, 2016.
6. Chai, J.-D.; Head-Gordon, M. Long-range Corrected Hybrid Density Functionals with Damped Atom-atom Dispersion Corrections. *Phys. Chem. Chem. Phys.* **10**, 6615−6620 (2008).
7. Weigend, F.; Ahlrichs, R. Balanced Basis Sets of Split Valence, Triple Zeta Valence and Quadruple Zeta Valence Quality for H to Rn: Design and Assessment of Accuracy. *Phys.Chem. Chem.Phys.* **7**, 3297−3305 (2005).
8. Marenich, A. V.; Cramer, C. J.; Truhlar, D. G. Universal Solvation Model Based on Solute Electron Density and on a Continuum Model of the Solvent Defined by the Bulk Dielectric Constant and Atomic Surface Tensions. *J. Phys. Chem. B* **113**, 6378−6396 (2009).
9. Grimme, S. Supramolecular Binding Thermodynamics by Dispersion-Corrected Density Functional Theory. *Chem. Eur. J.* **18**, 9955−9964 (2012).
10. Luchini, G.; Alegre-Requena, J. V.; Funes-Ardoiz, I.; Paton, R. S. F1000Research, 2020, 9, 291. GoodVibes version 3.2 DOI: 10.12688/f1000research.22758.1
